# Fatty acid traits mediate the effects of uric acid on cancers: a Mendelian randomization study

**DOI:** 10.3389/fgene.2024.1449205

**Published:** 2024-12-02

**Authors:** Jianing Li, Yongsheng Zhang, Tong Fu, Songyan Wang, Hongbo Cai, Fenghua Xu, Guoli Xing, Ying Tong

**Affiliations:** ^1^ Heilongjiang University of Chinese Medicine, Harbin, China; ^2^ Harbin Institute of Technology, Harbin, China; ^3^ Brandeis University, Waltham, MA, United States; ^4^ First Affiliated Hospital, Heilongjiang University of Chinese Medicine, Harbin, China

**Keywords:** Mendelian randomization, uric acid, fatty acids, cancer, mediate

## Abstract

**Introduction:**

Previous findings on the association between uric acid (UA) levels and cancer risk are conflicting. Moreover, the mechanisms underlying the interactions between UA levels, fatty acid traits, and cancer outcomes remain complex; it is still unclear whether elevated UA levels influence fatty acid traits and, thereby, contribute to an increased cancer risk. Therefore, we aimed to investigate the association between UA levels and cancer risk, with a specific focus on the potential mediating role of fatty acid traits.

**Methods:**

We employed a Mendelian randomization (MR) analysis utilizing genetic data from large-scale genome-wide association studies to assess the causal relationships among UA levels, fatty acid traits, and cancer risk. The primary method used was the inverse variance-weighted approach alongside Bayesian-weighted Mendelian randomization. Other MR models were also applied for comparison. Sensitivity analyses, based on various statistical assumptions, were also performed to evaluate the robustness of the findings. A two-step MR analysis was conducted to explore the mediating effects of fatty acid traits on the relationship between UA levels and cancer risk.

**Results and Discussion:**

Elevated UA levels were associated with an increased risk of *in situ* neoplasms, cervical cancer, and invasive mucinous ovarian cancer, while they were linked to a decreased risk of cancers of the eye and adnexa, small cell lung cancer, bronchus and lung cancer, respiratory system and intrathoracic organ cancers, as well as lung cancer. Mediation analysis revealed that fatty acid traits, particularly the docosahexaenoic acid/trans fatty acid ratio, mediated the relationship between UA levels and lung cancer risk. These findings underscore the potential of fatty acid traits to mediate the association between UA levels and cancer risk, offering new insights for targeted interventions and potentially improving clinical outcomes.

## 1 Introduction

Cancer remains a significant global health burden, with an estimated 19.3 million new cases and 10 million cancer-related deaths worldwide in 2020 (Danpanichkul et al., 2024), despite advances in cancer treatment. These figures underscore the persistent challenges of reducing cancer incidence and mortality. Continued research is essential to broaden our understanding of the mechanisms driving cancer onset and progression, which will facilitate the development of novel preventive strategies and early detection methods. Through these research efforts, identifying new therapeutic targets has become possible, leading to the creation of more effective treatments.

Uric acid (UA), the end product of purine metabolism, may regulate cancer development by promoting oxidative stress, inflammation, immune responses, and cellular metabolism (Zhou et al., 2022). UA influences cancer progression by impacting critical cell signaling pathways (Wang et al., 2024). Notably, UA regulates pathways involved in cancer cell behaviors, including proliferation, metastasis, and apoptosis (Crişan et al., 2017; Sanchez-Garrido and Shenoy, 2021). Elevated serum UA levels have been linked to an increased risk of certain cancers. For instance, UA crystals activate the inflammasome, which induces the expression of pro-carcinogenic factors such as the nuclear factor kappa-light-chain-enhancer of activated B cells (NF-κB) and interleukin 1 beta (IL-1β), promoting cancer cell proliferation and invasion (Fini et al., 2012). Studies have also found a correlation between high UA levels and the incidence of colorectal and hepatobiliary cancers (Yiu et al., 2017). In contrast, UA can exhibit protective effects under certain conditions by neutralizing reactive oxygen species (ROS) and mitigating oxidative stress due to its antioxidant properties (Szkandera et al., 2015). This dual role highlights the complex nature of UA in cancer development and progression, warranting further research to unravel the mechanisms through which UA influences tumorigenesis and provide critical new insights for cancer prevention and treatment.

Recent studies have also revealed that UA affects fatty acid metabolism via multiple pathways. First, UA inhibits pyruvate dehydrogenase activity, disrupting the tricarboxylic acid cycle. Consequently, cells are unable to efficiently oxidize fatty acids for energy, prompting a shift to alternative metabolic pathways such as glycolysis, which fosters cancer cell growth and proliferation (Nakagawa et al., 2020). Second, UA disrupts the normal oxidative metabolism of fatty acids, leading to their intracellular accumulation, which impairs cellular functions (Sánchez-Lozada et al., 2007). Consequently, fatty acid traits may serve as mediators between UA levels and cancer. However, detailed investigations into the pathways through which UA influences cancer development remain limited.

Furthermore, previous research on the role of UA in cancer has primarily relied on observational studies, which are limited in their ability to infer causality due to confounding factors, sample selection bias, and reverse causality. To address these limitations, Mendelian randomization (MR) provides a robust approach. MR uses genetic variation as an instrumental variable (IV) to simulate randomized controlled trials, thereby strengthening causal inference and reducing biases (Allegrini et al., 2022). Although previous studies have identified associations between UA levels, fatty acid traits, and cancer risk, none have quantitatively assessed these relationships using MR. Thus, by employing an MR framework, the effects of UA levels and fatty acid traits on cancer can be precisely quantified, advancing our understanding of how these factors contribute to cancer progression and guiding targeted prevention strategies.

In this study, we employed a bidirectional and comprehensive MR approach to assess the causal relationship between UA and cancer with a particular focus on the intermediary role of fatty acids based on genetic variants. Our aim was to investigate how UA influences cancer development and to examine the role of fatty acids in mediating this relationship. By uncovering novel causal relationships, our research provides valuable insights into cancer pathogenesis, with broad clinical implications for therapeutic development and prevention strategies.

## 2 Methods

### 2.1 Study design

**FIGURE 1:**
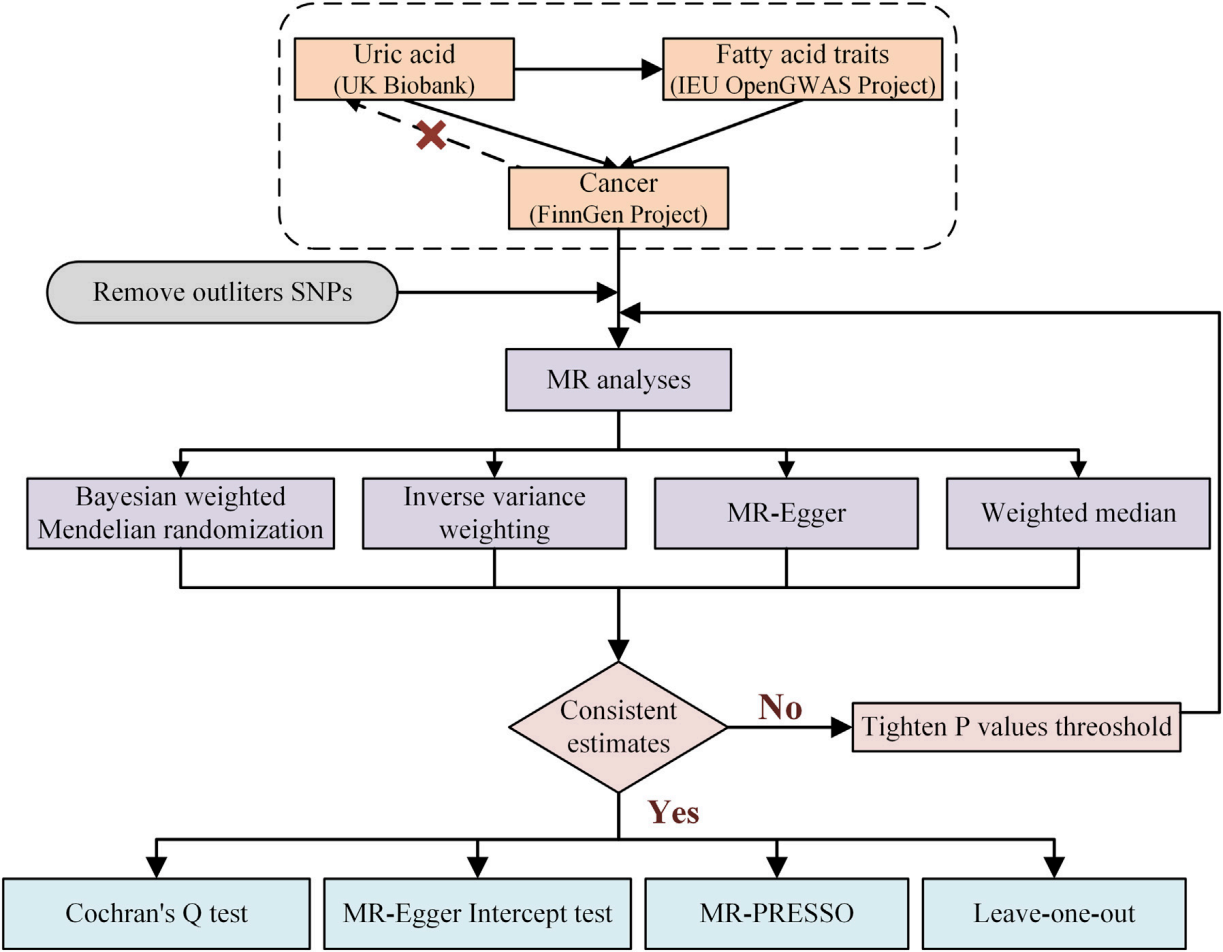
Research flow chart of the Mendelian randomization study.

Data preprocessing involved the following steps: (1) downloading summary statistics for publicly available genome-wide association studies (GWAS) from the UK Biobank, IEU Open GWAS project, and FinnGen project; and (2) harmonizing the datasets to remove ambiguous single-nucleotide polymorphisms (SNPs) and ensure consistency across exposure and outcome datasets. The overall study design, as illustrated in Figure 1, involved two distinct phases. In the first phase, the causal relationship between UA and cancer was evaluated using a two-way MR approach. In the second phase, a two-step MR analysis was used to delineate a causal pathway from UA to cancer, incorporating fatty acids as mediators. Subsequently, MR was employed to identify those fatty acids causally linked to cancer, and the effects of these fatty acids in mediating the link between UA and cancer were quantified. We utilized the TwoSampleMR (version 0.4.25) and MR-PRESSO (version 1.0) packages available in R (version 4.3.2) for the MR analysis. Key code snippets included MR implementation scripts, which are provided in the Supplementary Material S1.

### 2.2 Data sources

The UK Biobank is a large prospective resource that contains health and genomic data from approximately 5,00,000 participants and is designed to support research across a wide range of diseases. From this cohort, data from 49,960 participants aged 18 years and above were included in the UA panel. All participants had their hyperuricemia status confirmed through biomarkers such as serum UA levels. To ensure the accuracy and reliability of the results, individuals with a recent history of major or acute illness, as well as those with missing data, were excluded from the analysis (Barton et al., 2021). The IEU Open GWAS project serves as a platform that consolidates summary statistics from numerous GWAS with the goal of enhancing transparency and reproducibility in biomedical research. For the fatty acid group, 1,15,006 participants were ultimately enrolled, all of whom met diagnostic criteria for abnormalities in fatty acid metabolism. These criteria involved the measurement of various biomarkers including but not limited to free fatty acids and fatty acid esters. The participants were adults aged 18 years or older who provided informed consent to ensure voluntary participation and compliance with ethical standards. Similar to the UA panel, exclusion criteria included a recent history of major or acute illness that might affect fatty acid levels, as well as incomplete or unvalidated data to ensure the validity and reliability of the dataset (Richardson et al., 2022). The FinnGen project is a large-scale initiative encompassing the entire population of Finland, aiming to uncover the genetic basis of common diseases by integrating genomic data with comprehensive health registry information. The cancer cohort data used in this study obtained from the FinnGen project and included a total of 4,12,000 participants diagnosed with cancers in accordance with the International Classification of Diseases criteria. All of the participants were adults, and exclusion criteria included individuals with missing data or significant comorbidities to minimize potential confounding factors in the analysis (Kurki et al., 2023). No sample overlap was detected between the GWAS datasets pertaining to exposure, outcome, or mediators (Supplementary Material S2: Supplementary Table S1).

### 2.3 IV selection

Selecting IVs is a pivotal component of MR studies. By choosing IVs that are strongly linked to an exposure of interest but not to potential confounders, the causal effect of an exposure on an outcome can be precisely estimated. IVs typically comprise genetic variants that adhere to the following three fundamental assumptions: IVs should be significantly associated with the exposure, affect the outcome solely via exposure variables, and be independent of potential confounders (Li et al., 2024). We identified SNPs associated with fatty acids at the genome-wide significance threshold of *p* < 5.0 × 10^−8^ as potential IVs. Linkage disequilibrium (LD), which refers to genetic associations between different loci, can introduce bias or inaccuracies in MR analyses. To address this aspect, clumping procedures are frequently used to assess LD between IVs and reduce biased effect estimates resulting from genetic linkages. The standard LD threshold for clumping is set at *r* ^*2*^ < 0.001 within a window size of 10,000 kb (Long et al., 2024). The MR-PRESSO outlier test and global test were iteratively conducted until the *P*-value was no longer significant. Furthermore, the *F*-statistic serves as a common metric for evaluating the strength of the association between IVs and exposures, with a high *F*-statistic indicating a robust instrument. Specifically, IVs are considered strong when the *F*-statistic exceeds 10 (Long et al., 2024). The remaining SNPs were aggregated into the GWAS database for outcome evaluation.

### 2.4 MR analyses and mediation analysis

Various MR methods are utilized when exposure features comprise multiple IVs to estimate the overall effect. Among the different MR methods, the inverse variance-weighted (IVW) method was employed as the primary analytical approach because of its high statistical power. Bayesian weighted Mendelian randomization (BWMR) is a causal inference method based on GWAS data developed to overcome challenges such as weak genetic effects in polygenic traits and genetic pleiotropy, which may bias traditional MR results. BWMR improves causal inference by applying Bayesian weighting to reduce the impact of pleiotropic outliers and utilizes a variational expectation–maximization algorithm for more stable and efficient computation, thus effectively managing uncertainty in causal estimates (Zhao et al., 2020). Additionally, MR-Egger and weighted median approaches enhance IVW estimates, indicating their capacity to provide more robust estimates across a broader range of scenarios despite being less computationally efficient (Chen et al., 2021). In the mediation analysis, the association between UA and cancer was initially evaluated without considering potential mediating variables. Subsequently, the relationships among UA, fatty acids, and cancer were examined to evaluate mediating effects. Finally, the indirect and direct effects of UA on cancer via fatty acids were quantified, thereby elucidating the extent to which fatty acids mediate the UA–cancer relationship. The indirect effect as mediated by fatty acids was calculated as β1 × β2, where β1 represents the MR effect of UA on fatty acids and β2 represents the MR effect of fatty acids on cancer. The proportion of the mediating effect was determined as β1 × β2/β3, where β3 represents the MR effect of UA on cancer.

### 2.5 Sensitivity analyses

We conducted a thorough analysis of horizontal pleiotropy using the MR-Egger intercept test and leave-one-out analyses. Cochran’s Q test was employed to detect heterogeneity. A funnel plot was employed to evaluate the likely presence of directional pleiotropy. Ultimately, the MR-PRESSO technique was utilized to identify and eliminate outliers, effectively reducing the influence of pleiotropy (Greco et al., 2015; Emdin et al., 2017; Verbanck et al., 2018).

## 3 Results

### 3.1 IV selection

We incorporated data from 325 SNPs identified as IVs for UA-related traits (Supplementary Material 2: Supplementary Table S2) and 2,231 SNPs as IVs for fatty acid-related traits (Supplementary Material 2: Supplementary Table S3). Additionally, we utilized 3,609 SNPs as IVs for cancer traits (Supplementary Material 2: Supplementary Table S4). For each trait, the initial number of instrumental genes and the selection criteria are provided in the corresponding supplementary tables. Rigorous strength analyses were conducted on these IVs, revealing that all SNPs exhibited *F*-statistics exceeding 10. This confirmed that the selected IVs were strong and minimized potential confounding biases associated with weak IVs, indicating the robust predictive capabilities of our selected IVs.

### 3.2 Causal relationship between UA and cancer in the two-sample MR analysis

**FIGURE 2:**
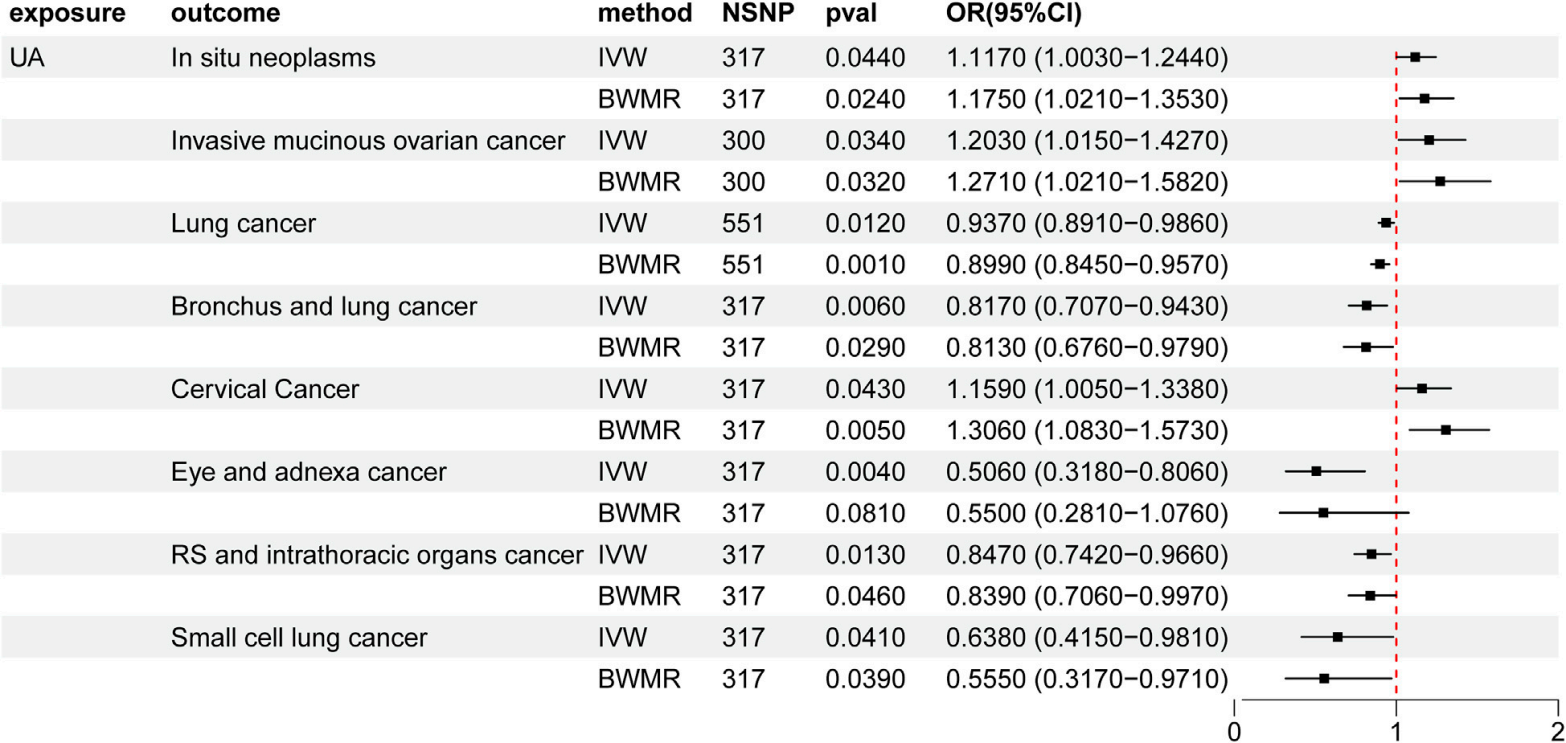
Mendelian randomization results of the causal effects of UA on cancer. UA: uric acid; IVW: inverse variance-weighted method; BWMR: Bayesian weighted Mendelian randomization; NSNP: number of single nucleotide polymorphism; OR: odds ratio; CI: confidence interval.

Heightened levels of UA were associated with an elevated risk of *in situ* neoplasms, cervical cancer, and invasive mucinous ovarian cancer. Conversely, higher UA levels were linked to a reduced risk of eye and adnexa cancer, small cell lung cancer, bronchus and lung cancer, respiratory system and intrathoracic organ cancer, and lung cancer (Supplementary Material 2, Table 1; Figure 2). However, the reverse MR analyses failed to uncover any evidence supporting a causal effect of UA on these cancers. The selected IVs for UA associated with the different cancers are detailed in the Supplementary Material (Supplementary Table S1).

**TABLE 1:** Causal relationship between UA and cancer in the two-sample MR analysis.

Exposure	Outcome	Method	*P*-value	OR (95% CI)
UA	*In situ* neoplasms	IVW	0.044	1.117 (1.003, 1.244)
UA	*In situ* neoplasms	BWMR	0.024	1.175 (1.021, 1.353)
UA	Invasive mucinous ovarian cancer	IVW	0.034	1.203 (1.015, 1.427)
UA	Invasive mucinous ovarian cancer	BWMR	0.032	1.271 (1.021, 1.582)
UA	Lung cancer	IVW	0.012	0.937 (0.891, 0.986)
UA	Lung cancer	BWMR	0.001	0.899 (0.845, 0.957)
UA	Bronchus and lung cancer	IVW	0.006	0.817 (0.707, 0.943)
UA	Bronchus and lung cancer	BWMR	0.029	0.813 (0.676, 0.979)
UA	Cervical Cancer	IVW	0.043	1.159 (1.005, 1.338)
UA	Cervical Cancer	BWMR	0.005	1.306 (1.083, 1.573)
UA	Eye and adnexa cancer	IVW	0.004	0.506 (0.318, 0.806)
UA	Eye and adnexa cancer	BWMR	0.081	0.55 (0.281, 1.076)
UA	RS and intrathoracic organs cancer	IVW	0.013	0.847 (0.742, 0.966)
UA	RS and intrathoracic organs cancer	BWMR	0.046	0.839 (0.706, 0.997)
UA	Small cell lung cancer	IVW	0.041	0.638 (0.415, 0.981)
UA	Small cell lung cancer	BWMR	0.039	0.555 (0.317, 0.971)

UA, uric acid; IVW, inverse variance-weighted method; BWMR, bayesian weighted mendelian randomization; OR, odds ratio; CI, confidence interval.

### 3.3 Causal relationship between UA and fatty acid traits in the two-sample MR analysis

**FIGURE 3:**
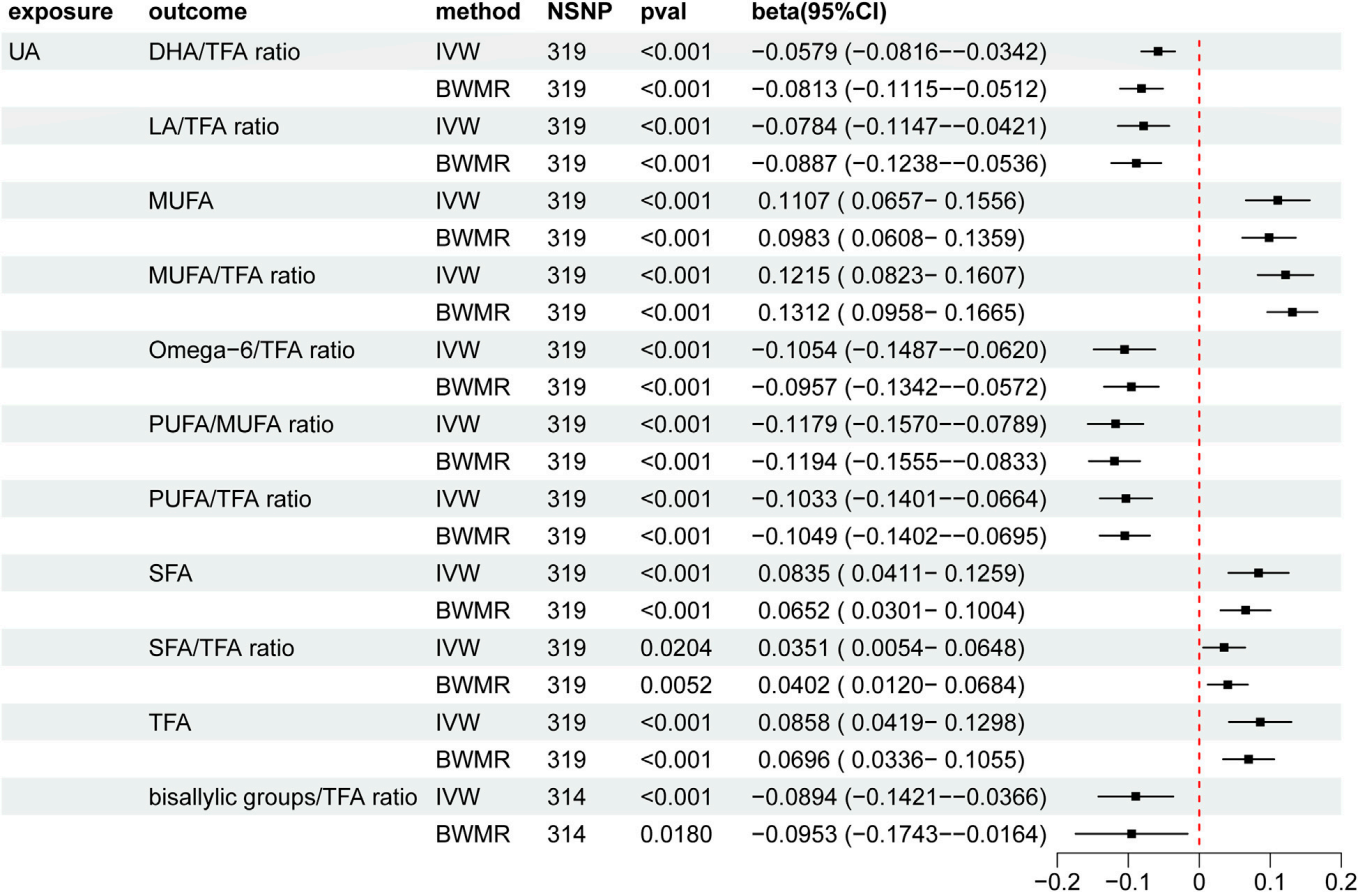
Mendelian randomization results of the causal effects of UA on fatty acid traits. UA: uric acid; IVW: inverse variance-weighted method; BWMR: Bayesian weighted Mendelian randomization; TFA: trans fatty acid; SFA: saturated fatty acid; PUFA: polyunsaturated fatty acid; MUFA: monounsaturated fatty acid; LA: linoleic acid; DHA: docosahexaenoic acid; NSNP: number of single nucleotide polymorphisms; CI: confidence interval.

Our analyses revealed significant changes in several fatty acid ratios with increasing levels of UA. Specifically, the polyunsaturated fatty acids (PUFAs)/monounsaturated fatty acids (MUFAs) ratio, omega-6/trans fatty acids (TFAs) ratio, PUFA/TFA ratio, bisallylic groups/TFA ratio, linoleic acid (LA)/TFA ratio, and docosahexaenoic acid (DHA)/TFA ratio gradually decreased, whereas the saturated fatty acids (SFAs)/TFA ratio, SFA, TFA, MUFA, MUFA, MUFA/TFA ratio, and LA/TFA ratio gradually increased (Supplementary Material 2, Table 2; Figure 3). The selected IVs for UA associated with fatty acid traits are detailed in the Supplementary Material (Supplementary Table S6).

**TABLE 2:** Causal relationship between UA and fatty acid traits in the two-sample MR analysis.

Exposure	Outcome	Method	*P*-value	Beta (95% CI)
UA	bisallylic groups/TFA ratio	BWMR	0.018	−0.095 (−0.174, −0.016)
UA	bisallylic groups/TFA	IVW	9.03 × 10^−4^	−0.089 (−0.142, −0.037)
UA	TFA	BWMR	1.50 × 10^−4^	0.07 (0.034, 0.106)
UA	TFA	IVW	1.31 × 10^−4^	0.086 (0.042, 0.13)
UA	SFA/TFA ratio	BWMR	0.005	0.04 (0.012, 0.068)
UA	SFA/TFA ratio	IVW	0.020	0.035 (0.005, 0.065)
UA	SFA	BWMR	2.77 × 10^−4^	0.065 (0.03, 0.1)
UA	SFA	IVW	1.12 × 10^−4^	0.084 (0.041, 0.126)
UA	PUFA/TFA ratio	BWMR	6.04 × 10^−9^	−0.105 (−0.14, −0.07)
UA	PUFA/TFA ratio	IVW	3.96 × 10^−8^	−0.103 (−0.14, −0.066)
UA	PUFA/MUFA ratio	BWMR	8.69 × 10^−11^	−0.119 (−0.155, −0.083)
UA	PUFA/MUFA ratio	IVW	3.27 × 10^−9^	−0.118 (−0.157, −0.079)
UA	Omega-6/TFA ratio	BWMR	1.11 × 10^−6^	−0.096 (−0.134, −0.057)
UA	Omega-6/TFA ratio	IVW	1.89 × 10^−6^	−0.105 (−0.149, −0.062)
UA	MUFA/TFA ratio	BWMR	3.46 × 10^−13^	0.131 (0.096, 0.167)
UA	MUFA/TFA ratio	IVW	1.26 × 10^−9^	0.122 (0.082, 0.161)
UA	MUFA	BWMR	2.85 × 10^−7^	0.098 (0.061, 0.136)
UA	MUFA	IVW	1.37 × 10^−6^	0.111 (0.066, 0.156)
UA	LA/TFA ratio	BWMR	7.31 × 10^−7^	−0.089 (−0.124, −0.054)
UA	LA/TFA ratio	IVW	2.30 × 10^−5^	−0.078 (−0.115, −0.042)
UA	DHA/TFA ratio	BWMR	1.28 × 10^−7^	−0.081 (−0.112, −0.051)
UA	DHA/TFA ratio	IVW	1.75 × 10^−6^	−0.058 (−0.082, −0.034)

UA, uric acid; IVW, inverse variance-weighted method; BWMR, bayesian weighted mendelian randomization; TFA, trans fatty acid; SFA, saturated fatty acid; PUFA, polyunsaturated fatty acid; MUFA, monounsaturated fatty acid; LA, linoleic acid; DHA, docosahexaenoic acid; CI, confidence interval.

### 3.4 Causal relationship between fatty acid traits and cancer in the two-sample MR analysis

**FIGURE 4:**
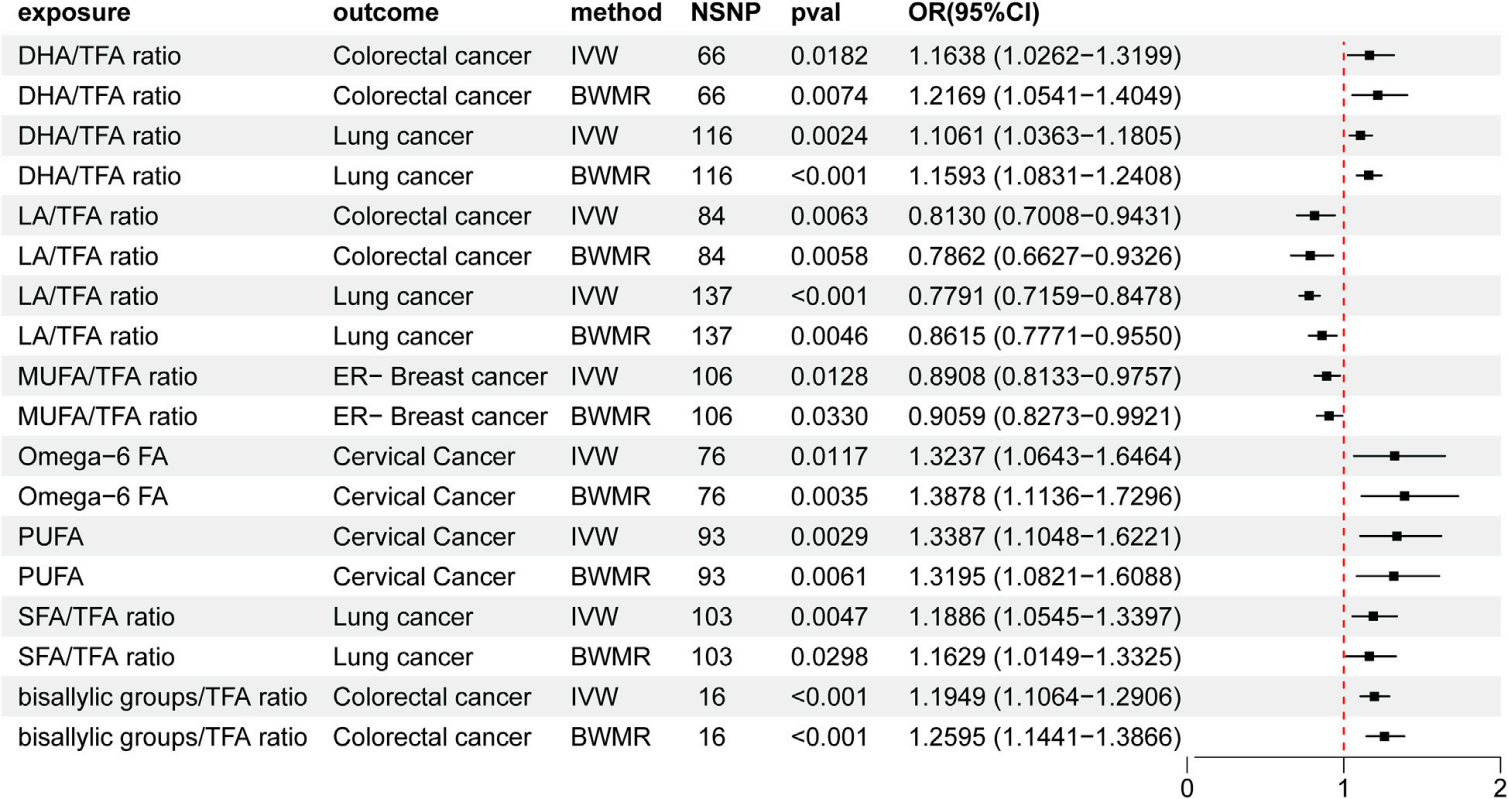
Mendelian randomization results of the causal effects of fatty acid traits and cancer. UA: uric acid; IVW: inverse variance-weighted method; BWMR: Bayesian weighted Mendelian randomization; TFA: trans fatty acid; SFA: saturated fatty acid; PUFA: polyunsaturated fatty acid; MUFA: monounsaturated fatty acid; LA: linoleic acid; DHA: docosahexaenoic acid; NSNP: number of single nucleotide polymorphisms; OR: odds ratio; CI: confidence interval.

Elevated DHA/TFA ratios increased the risk of colorectal cancer and lung cancer. Similarly, an increased LA/TFA ratio was associated with a decreased risk of colorectal cancer and lung cancer. Increased MUFA/TFA ratios were associated with a reduced risk of ER-breast cancer. Increased omega-6/TFA ratios and PUFA were associated with a higher risk of cervical cancer. In addition, increased SFA/TFA and bisallylic groups/TFA ratios were associated with an increased risk of lung cancer and colorectal cancer (Supplementary Material 2, Table 3; Figure 4).

**TABLE 3:** Causal relationship between fatty acid traits and cancer in the two-sample MR analysis.

Exposure	Outcome	Method	*P*-value	OR (95% CI)
DHA/TFA ratio	Colorectal cancer	IVW	0.018	1.164 (1.026, 1.32)
DHA/TFA ratio	Colorectal cancer	BWMR	0.007	1.217 (1.054, 1.405)
DHA/TFA ratio	Lung cancer	IVW	0.002	1.106 (1.036, 1.181)
DHA/TFA ratio	Lung cancer	BWMR	2.04 × 10^−5^	1.159 (1.083, 1.241)
LA/TFA ratio	Colorectal cancer	IVW	0.006	0.786 (0.663, 0.933)
LA/TFA ratio	Colorectal cancer	BWMR	0.006	0.813 (0.701, 0.943)
LA/TFA ratio	Lung cancer	IVW	7.22 × 10^−9^	0.779 (0.716, 0.848)
LA/TFA ratio	Lung cancer	BWMR	0.005	0.861 (0.777, 0.955)
MUFA/TFA ratio	ER- Breast cancer	IVW	0.033	0.906 (0.827, 0.992)
MUFA/TFA ratio	ER- Breast cancer	BWMR	0.013	0.891 (0.813, 0.976)
Omega-6/TFA ratio	Cervical Cancer	IVW	0.004	1.388 (1.114, 1.73)
Omega-6/TFA ratio	Cervical Cancer	BWMR	0.012	1.324 (1.064, 1.646)
PUFA	Cervical Cancer	IVW	0.006	1.319 (1.082, 1.609)
PUFA	Cervical Cancer	BWMR	0.003	1.339 (1.105, 1.622)
SFA/TFA ratio	Lung cancer	IVW	0.03	1.163 (1.015, 1.333)
SFA/TFA ratio	Lung cancer	BWMR	0.005	1.189 (1.055, 1.34)
bisallylic groups/TFA ratio	Colorectal cancer	IVW	2.54 × 10^−6^	1.26 (1.144, 1.387)
bisallylic groups/TFA ratio	Colorectal cancer	BWMR	5.81 × 10^−6^	1.195 (1.106, 1.291)

UA, uric acid; IVW, inverse variance-weighted method; BWMR, Bayesian weighted Mendelian randomization; TFA, trans fatty acid; SFA, saturated fatty acid; PUFA, polyunsaturated fatty acid; MUFA, monounsaturated fatty acid; LA, linoleic acid; DHA, docosahexaenoic acid; CI, confidence interval.

### 3.5 Mediation analysis

**FIGURE 5:**
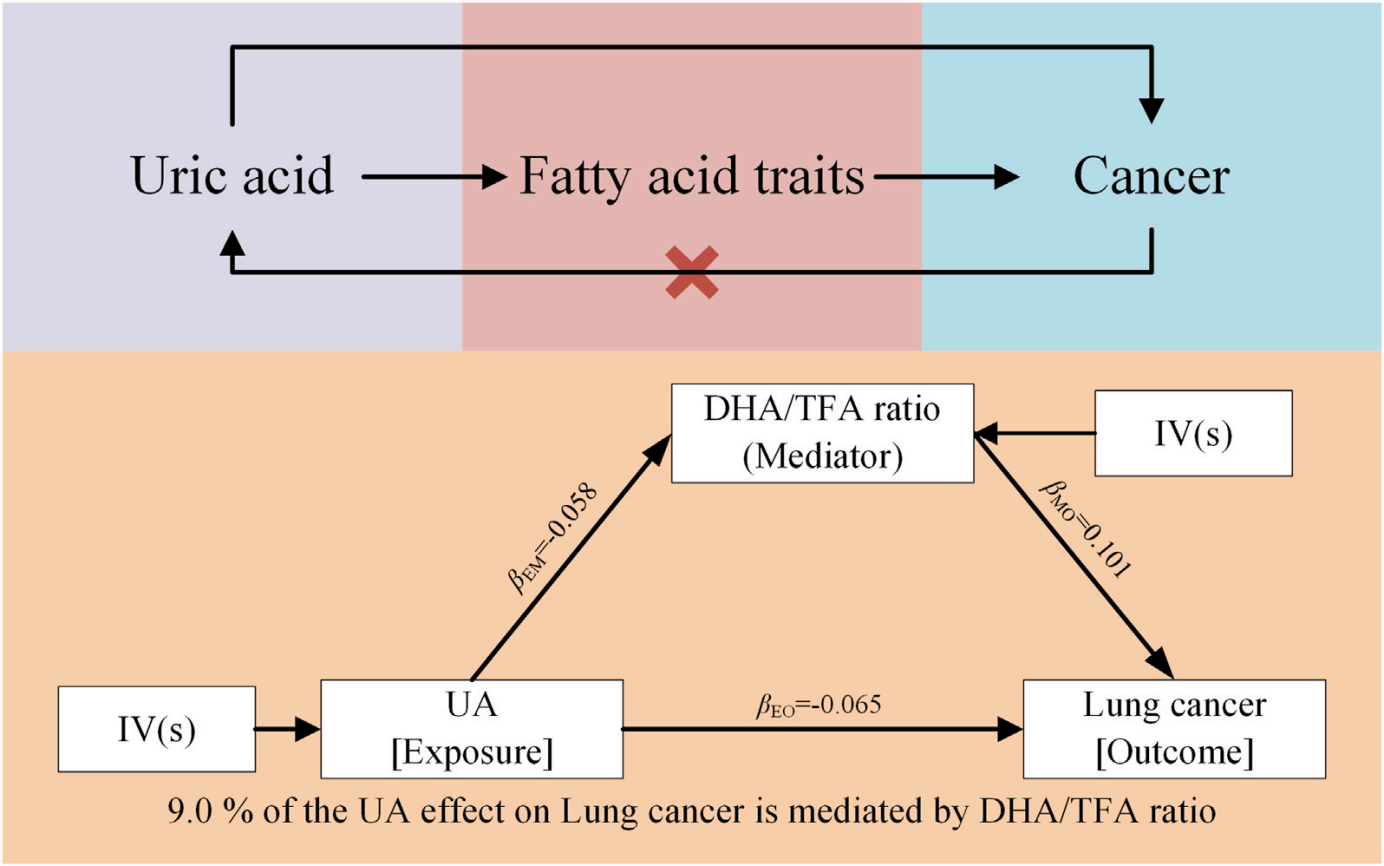
Mendelian randomization results of UA on cancer via fatty acid traits, specifically the DHA/TFA ratio.

Our findings indicate that UA plays a significant role in mitigating lung cancer progression by reducing the DHA/TFA ratio. Specifically, the reduction in this ratio mediates the relationship between elevated UA levels and decreased lung cancer progression. This suggests that higher UA levels result in a lower DHA/TFA ratio, which, in turn, reduces the risk of lung cancer progression. The mediating effect of UA on the DHA/TFA ratio accounted for 9% of the overall reduction in lung cancer progression (Figure 5). Although other factors may also influence lung cancer progression, the UA-induced reduction in the DHA/TFA ratio represents a substantial mediating factor.

### 3.6 Sensitivity analysis

The MR-Egger intercept test showed that all *P*-values were greater than 0.05, indicating no horizontal pleiotropy. The leave-one-out analysis and funnel plots are provided in the Supplementary Materials S3, S4. The estimations were not biased by a single SNP, suggesting that the estimates were not violated. All *P*-values generated in the Cochran’s Q test exceeded 0.05. Additionally, MR-PRESSO analysis revealed no evidence of horizontal pleiotropy in the MR study.

## 4 Discussion

Previous research has shown that UA is not only an important marker for metabolic diseases, such as gout, but may also be closely associated with the development of various tumors (Fini et al., 2012). As the end product of purine metabolism, UA accumulation can trigger pathological processes, including oxidative stress, inflammatory responses, and abnormal cellular metabolism (Maiuolo et al., 2016). Fatty acids, as key components of energy metabolism, also play a pivotal role in regulating inflammation and cellular metabolism (Carracedo et al., 2013). The complex interactions between fatty acids and UA in metabolic pathways may significantly influence tumorigenesis and cancer progression by affecting the tumor microenvironment. A deeper investigation into the molecular mechanisms linking UA, fatty acids, and cancer risk is, therefore, valuable for uncovering their specific roles in tumorigenesis and providing new directions for future therapeutic interventions.

UA accumulation in the body can contribute to tumorigenesis through multiple mechanisms. First, as a danger-signaling molecule, UA activates inflammasomes, inducing downstream inflammatory responses and promoting tumor microenvironment remodeling (Guo et al., 2015; Meyers and Zhu, 2020). Second, UA elevates cancer risk by inducing oxidative stress and disrupting the antioxidant balance within cells, leading to deoxyribonucleic acid (DNA) damage and gene mutations. Fatty acids further impact cancer cell survival and proliferation by regulating energy metabolism, lipid peroxidation, and signal transduction pathways within the tumor microenvironment (Carracedo et al., 2013). They also meet the heightened energy demands of cancer cells, promoting malignant growth through metabolic reprogramming (Ediriweera and Jayasena, 2023).

Our study suggests that the interaction between UA and fatty acids regulates tumor progression through various signaling pathways. For example, UA promotes cell proliferation and metabolic activity by activating the signaling pathway, driving tumor progression (Johnson et al., 2010). Disturbances in fatty acid metabolism, in turn, activate the AMP-activated protein kinase (AMPK) signaling pathway, which inhibits cancer cell apoptosis and enhances tolerance to external stress. These signaling pathway interactions are critical in the tumor microenvironment, indirectly promoting tumor growth and metastasis by affecting immune cell function and inflammatory responses (Meyers and Zhu, 2020).

Considering the complex metabolic pathways involved in tumor progression, it is crucial to explore how external factors, such as circadian rhythms, influence fatty acid and UA metabolism, potentially affecting cancer development (Gnocchi et al., 2015). Circadian rhythms modulate UA levels by regulating fatty acid metabolism, which may mediate tumorigenesis and progression. For example, fatty acid metabolism follows circadian fluctuations, with lipid synthesis, transport, and catabolism regulated by the biological clock. Thus, disruption of circadian rhythms triggers lipid metabolism abnormalities, leading to fatty acid accumulation (Gnocchi and Bruscalupi, 2017). This accumulation promotes UA production as UA is also a byproduct of lipid and purine metabolism (Kanemitsu et al., 2017). High UA levels trigger oxidative stress and chronic inflammatory responses, which are important triggers of tumorigenesis. In addition, circadian rhythms directly modulate the immune response and affect the ability of the body to fight tumors. When the circadian rhythm is disturbed, fatty acid and UA metabolism is imbalanced and the immune-monitoring function of the body may also be suppressed, providing a favorable environment for the proliferation and metastasis of cancer cells (Kanabrocki et al., 2000). Therefore, circadian rhythm disruption indirectly contributes to tumorigenesis by affecting fatty acid metabolism and UA levels, especially in cancers driven by metabolic disorders.

Our study revealed a complex relationship between elevated UA levels and the risk of various cancers. While UA promotes certain cancers, it exhibits protective effects in others. A substantial body of preclinical and clinical evidence supports this dual role (Fan et al., 2023). Elevated UA levels have been associated with *in situ* tumors in several studies (Wu et al., 2023). For example, higher serum UA levels in cervical cancer patients are significantly linked to tumor malignancy and poor prognosis (De et al., 2022). UA, serving as a promoter of oxidative stress, may contribute to carcinogenesis by impairing cellular DNA repair mechanisms. In invasive mucinous ovarian cancer, UA plays a key role in tumor growth and metastasis, with high UA levels enhancing the invasiveness of cancer cells by activating redox pathways (Zhao et al., 2024). These studies have suggested that the pro-inflammatory and pro-oxidative properties of UA enable tumor progression in these specific cancers.

Our study further revealed a remarkable duality in the role of UA in various cancers, potentially attributable to its complex *in vivo* metabolism and varying functional expression under different physiological conditions. The antioxidant properties of UA provide protective effects in certain cancers. For instance, one clinical study on small-cell lung cancer reported that elevated UA levels were linked to increased patient survival, possibly due to the capacity of UA to scavenge free radicals and, thereby, reduce oxidative stress, minimize cellular damage, and inhibit cancer cell spread and proliferation (Mi et al., 2020). Similarly, in ocular appendage and respiratory-related cancers, UA functions as an endogenous antioxidant, potentially mitigating oxidative damage and slowing cancer progression (Lin et al., 2024). However, the role of UA is not always so simple. Consistent with our study and its findings, previous studies have shown that under specific conditions, UA promotes tumorigenesis through oxidative stress and inflammatory pathways in metabolically disturbed states (Mi et al., 2020). For example, UA may alter the cellular microenvironment and promote the growth and invasion of cancer cells through its oxidative stress and inflammatory response properties in metabolically disturbed states (Allegrini et al., 2022). These effects may be related to the contribution of UA to cancer development by inducing elevated intracellular ROS levels and causing DNA damage and gene mutations (Fini et al., 2012). Moreover, under specific metabolic conditions, elevated UA levels may synergize with obesity, hypertension, and other metabolic disorders to accelerate cancer cell proliferation and angiogenesis, ultimately increasing cancer risk (Allegrini et al., 2022). Therefore, the role of UA in various cancers may be influenced by multiple factors including a patient’s metabolic status, genetic background, and cancer type. This dual role indicates the need for more in-depth exploration of the molecular mechanisms underlying the role of UA in different tumor types as well as its interactions with other metabolic factors. Further research in these areas would help to clarify the specific role of UA in tumorigenesis and cancer development, providing a more comprehensive theoretical foundation for its potential use in cancer risk assessment and therapeutic intervention.

Considering the complexity in the mechanism of UA and tumorigenesis, we further investigated the effect of elevated UA levels on fatty acid metabolism and found significant changes in multiple fatty acid ratios. Our study revealed that elevated UA levels are associated with significant changes in the ratios of various fatty acids that may reflect the complex metabolic regulatory role of UA *in vivo* and could potentially serve as biomarkers in different tissues. Notably, the decreases in the ratios of PUFA to MUFA, omega-6 to TFA, and PUFA to TFA suggest that as UA levels increase, fatty acid metabolism may shift towards MUFAs and SFAs (Fini et al., 2021). This shift is consistent with a more aggressive tumor phenotype, as previous studies have indicated a significant association between elevated MUFA levels and increased cancer cell proliferation (Dovell and Boffetta, 2018).

Our observed changes in fatty acid profiles may contribute to cancer progression through multiple mechanisms. Elevated MUFA levels, for instance, may activate oncogenic signaling pathways, such as mTOR, promoting cancer cell survival and proliferation (Camargo et al., 2018). Increased omega-6/TFA ratios are also closely linked to inflammation, a well-known driver of cancer progression (Calder, 2017). Moreover, higher SFA and TFA levels are also correlated with enhanced oxidative stress, which may further promote tumor initiation and development (Comba et al., 2010). These findings are consistent with previous reports indicating that disruptions in fatty acid metabolism can lead to poor clinical outcomes in certain cancers. For instance, a high SFA/TFA ratio has been significantly linked to disease progression in specific gastrointestinal cancers, likely due to its role in altering lipid metabolism pathways that facilitate cancer cell proliferation and invasion (Carmody et al., 2015).

Exploring the role of fatty acids in different cancers is crucial given the observed relationship between UA levels and fatty acid metabolism. Our findings indicate that changes in fatty acid ratios are strongly associated with cancer risk, and several studies support this link (Mei et al., 2024; Farag and Gad, 2022). For example, we observed that elevated DHA/TFA ratios were associated with an increased risk of colorectal and lung cancer (Nagarajan et al., 2021). Although DHA is an omega-3 polyunsaturated fatty acid known for its anti-inflammatory effects, it may exhibit pro-carcinogenic effects in certain cellular environments, particularly in intestinal and lung cells. DHA from deep-sea fish, while potentially beneficial for some cancer prevention, may have specific metabolic pathways that promote cancer cell proliferation in these cancers. This dual role reflects the complex effects of fatty acid metabolism on different organs and cancer types (Johnson et al., 2010). In contrast, the association between elevated LA/TFA ratios and a reduced risk of colorectal and lung cancers suggests that LA intake may be protective. LA, an omega-6 fatty acid commonly found in vegetable oils, is generally regarded as pro-inflammatory, but its ability to inhibit cell proliferation and reduce inflammation is enhanced when combined with a lower TFA diet, thereby reducing cancer risk (Moral et al., 2022). These complex interactions between fatty acids may influence cancer pathogenesis by modulating inflammation.

We also found that an elevated MUFA/TFA ratio was linked to a reduced risk of ER-breast cancer, aligning with findings that MUFAs, particularly from olive oil and nuts, have protective effects against ER-breast cancer by modulating apoptotic and inflammatory pathways (Moral et al., 2022) and inhibiting cell proliferation and inducing apoptosis (MacLennan and Ma, 2010). This aligns with findings of previous studies suggesting that the anticancer potential of MUFAs may be due to their impact on cellular metabolic pathways. In contrast, epidemiological studies on cervical cancer have shown that an elevated intake of omega-6 fatty acids and PUFAs is strongly associated with increased cancer risk (Chen et al., 2021). Omega-6 fatty acids, primarily found in red meat and certain vegetable oils, promote the production of prostaglandin E2 (PGE2), a proinflammatory molecule. When omega-6 fatty acids accumulate excessively, PGE2 overexpression enhances cell proliferation and survival, contributing to cancer development under certain conditions (Chen et al., 2021). This evidence further supports our findings. Additionally, our research reveals that elevated SFA/TFA ratios are associated with increased lung and colorectal cancer risk. High SFA intake is closely linked to dyslipidemia and an enhanced inflammatory response—effects that may be amplified in by high TFA intake (Farag and Gad, 2022). This suggests that the interaction between SFA and TFA may accelerate cancer progression by exacerbating inflammation. Similarly, elevated dienophile/TFA ratios have been associated with a higher incidence of colorectal and lung cancer, indicating that specific fatty acid combinations may have a synergistic effect on cancer risk (Nagarajan et al., 2021).

By exploring the complex interrelationship between UA and cancer risk, our study showed a significant association between UA levels and DHA/TFA ratios. Further analysis revealed that lower UA levels were strongly correlated with a higher risk of lung cancer, mainly due to an increased DHA/TFA ratio. These findings suggest that UA influences tumorigenesis and progression not only through conventional mechanisms but also by altering fatty acid metabolism, particularly DHA metabolism (Azrad et al., 2013). The impact of UA on DHA metabolism and function is multifaceted. First, the structural similarity between UA and DHA may influence DHA solubility, with UA being more soluble than DHA (Jiang et al., 2021). Consequently, UA might regulate the deposition and accumulation of DHA. Additionally, UA and DHA undergo metabolic changes through similar pathways in the body, and the metabolic and excretory processes of UA may compete with those of DHA, potentially affecting DHA production and metabolic dynamics (Wilkinson et al., 2004). Although DHA, an omega-3 fatty acid, is widely recognized for its health benefits, some studies have linked DHA to tumor promotion in specific conditions (Li et al., 2014). DHA interacts with tumor cell membrane phospholipids, altering membrane fluidity and function, which can influence cellular signaling critical to tumor growth and development. DHA also affects the immune system by regulating immune cell activity and cytokine secretion, which may influence the body’s immune response to tumors and, thereby, affect tumor progression (Li et al., 2014). UA plays a significant role in cancer development through the oxidative stress pathway. Elevated intracellular UA levels can induce oxidative stress, leading to the overproduction of ROS, which can damage cellular DNA and membranes, thus promoting cancer cell proliferation and metastasis (Mi et al., 2020).

Moreover, the role of UA in carcinogenesis involves its impact on the inflammatory response (Yu et al., 2022). High UA levels activate inflammatory cells and promote the release of inflammatory mediators, creating a chronic inflammatory environment that supports cancer development (Wang et al., 2022). Such chronic inflammation may explain the observed decrease in the DHA/TFA ratio in response to high UA levels, which may slow lung cancer progression. Zhang et al. (2019) provided evidence supporting this mechanism by showing that DHA inhibits the release of inflammatory factors in monosodium urate (MSU)-induced THP-1 cells. Using a murine model, these authors showed that oral intake of DHA-rich microalgae oil significantly reduced neutrophil influx and inflammatory cytokine production. Furthermore, DHA treatment alleviated MSU-induced intracellular ROS production and reversed mitochondrial membrane potential damage (Zhang et al., 2019). These findings highlight the role of DHA in mitigating UA-induced oxidative stress and inflammatory responses, reinforcing the idea that elevated UA levels lower the DHA/TFA ratio and reduce lung cancer progression. TFAs may also reduce the activation of pro-inflammatory genes by altering the fatty acid composition of phosphatidic acid in cell membranes, whereby disruption of lipid rafts inhibits the activation of pro-inflammatory transcription factors (Calder, 2017). These mechanisms help to explain how elevated UA levels may reduce the DHA/TFA ratio and slow lung cancer progression.

Overall, our study is the first to propose that UA may slow lung cancer progression by regulating fatty acid metabolism, particularly through its impact on the DHA/TFA ratio. These findings offer new avenues for cancer prevention and treatment, suggesting that further exploration of the interactions between UA and fatty acid metabolism could lead to metabolism-based therapeutic strategies. Notably, the causal relationship between UA levels and cancer risk was revealed using MR methods, and the mediating role of fatty acids in this association highlights important mechanisms in cancer development. Methodologically, MR reduces bias from confounding factors and reverse causality, improving the accuracy of causal inferences.

This study has few limitations. First, we used data from multiple GWAS databases, including the UK Biobank and FinnGen, to assess associations between genetic variants and disease risk. Methodological differences in phenotype definitions, measurement methods, and participant selection across these databases could introduce variability and bias, affecting comparability. Nonetheless, to mitigate these issues, we employed standardized analytic processes and conducted sensitivity analyses to ensure the consistency and robustness of the results across studies. These measures helped to minimize potential bias due to methodological differences and improved the reliability of our findings. Second, although the GWAS database is publicly available, certain data protections limited our access to detailed participant information, preventing the direct observation of individual-level effects, which may have introduced some uncertainty. Third, focusing solely on individuals of European ancestry—while helping to control for demographic variation—limits the generalizability of our findings to other populations. Finally, horizontal pleiotropy cannot be fully excluded, although we employed various techniques to minimize it. Future studies should explore the specific roles of UA and fatty acids in cancer pathogenesis, providing broader insights into cancer prevention and treatment strategies.

## 5 Conclusion

This study established a causal link between elevated UA levels and increased cancer risk based on MR analysis, emphasizing the mediating role of specific fatty acids. These findings provide new insights into cancer prevention and treatment strategies, particularly those based on targeting fatty acid metabolism. Future research should focus on elucidating the precise contributions of UA and fatty acids in cancer pathogenesis to identify potential avenues for targeted cancer interventions.

## Data Availability

The datasets presented in this study can be found in online repositories. The names of the repository/repositories and accession number(s) can be found in the article/Supplementary Material.
