# Supplementary material for: Fatty acid traits mediate the effects of uric acid on cancers: a Mendelian randomization study
Source: Front Genet. 2024 Dec 2;15:1449205. doi: 10.3389/fgene.2024.1449205 (PMC11646984; doi:10.3389/fgene.2024.1449205)
Supplement: Supplementary file 1 [file Image2.pdf]

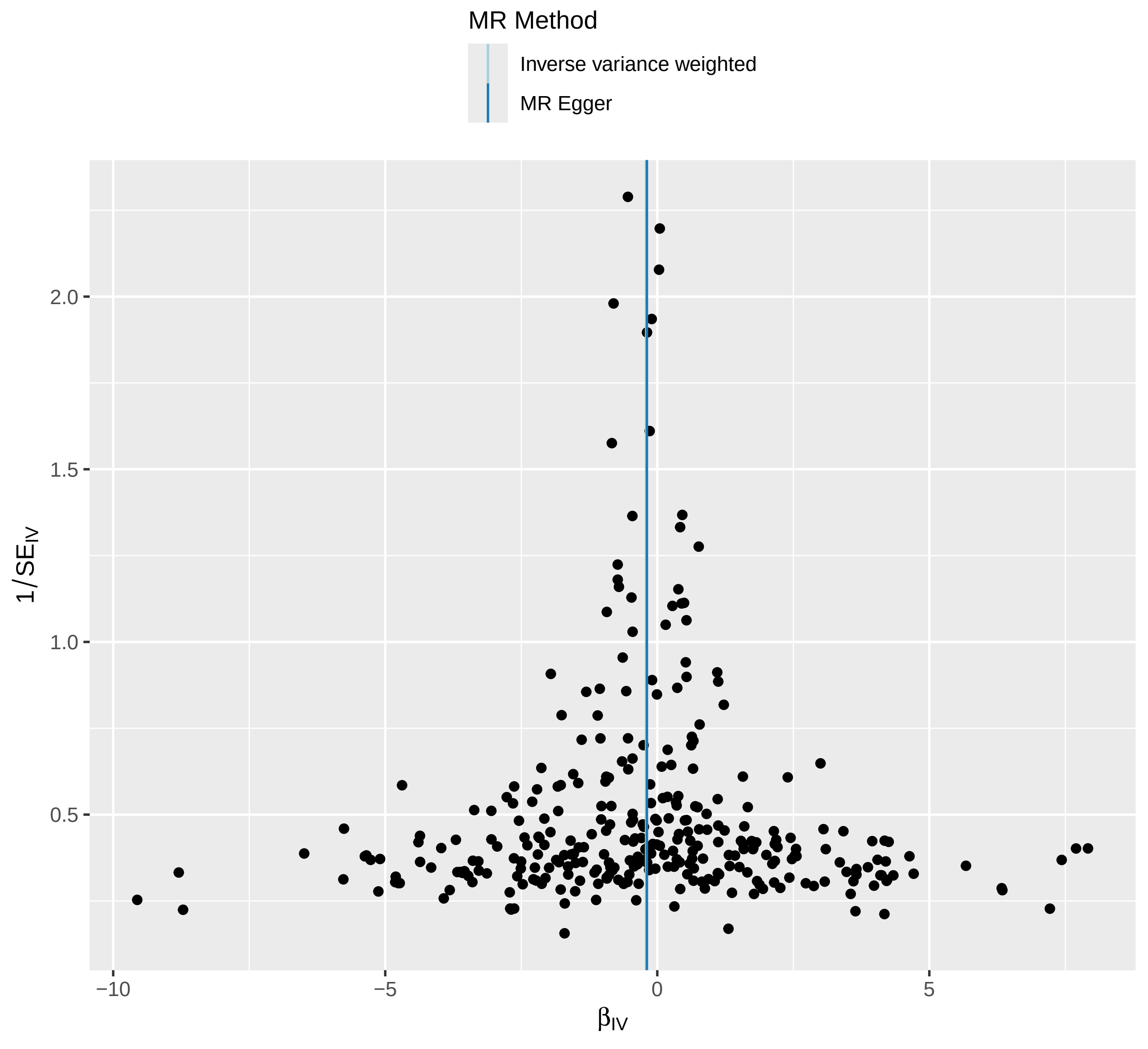
MR Method
Inverse variance weighted
MR Egger
1/SE_IV
2.0
1.5
1.0
0.5
−10
−5
0
5
β_IV

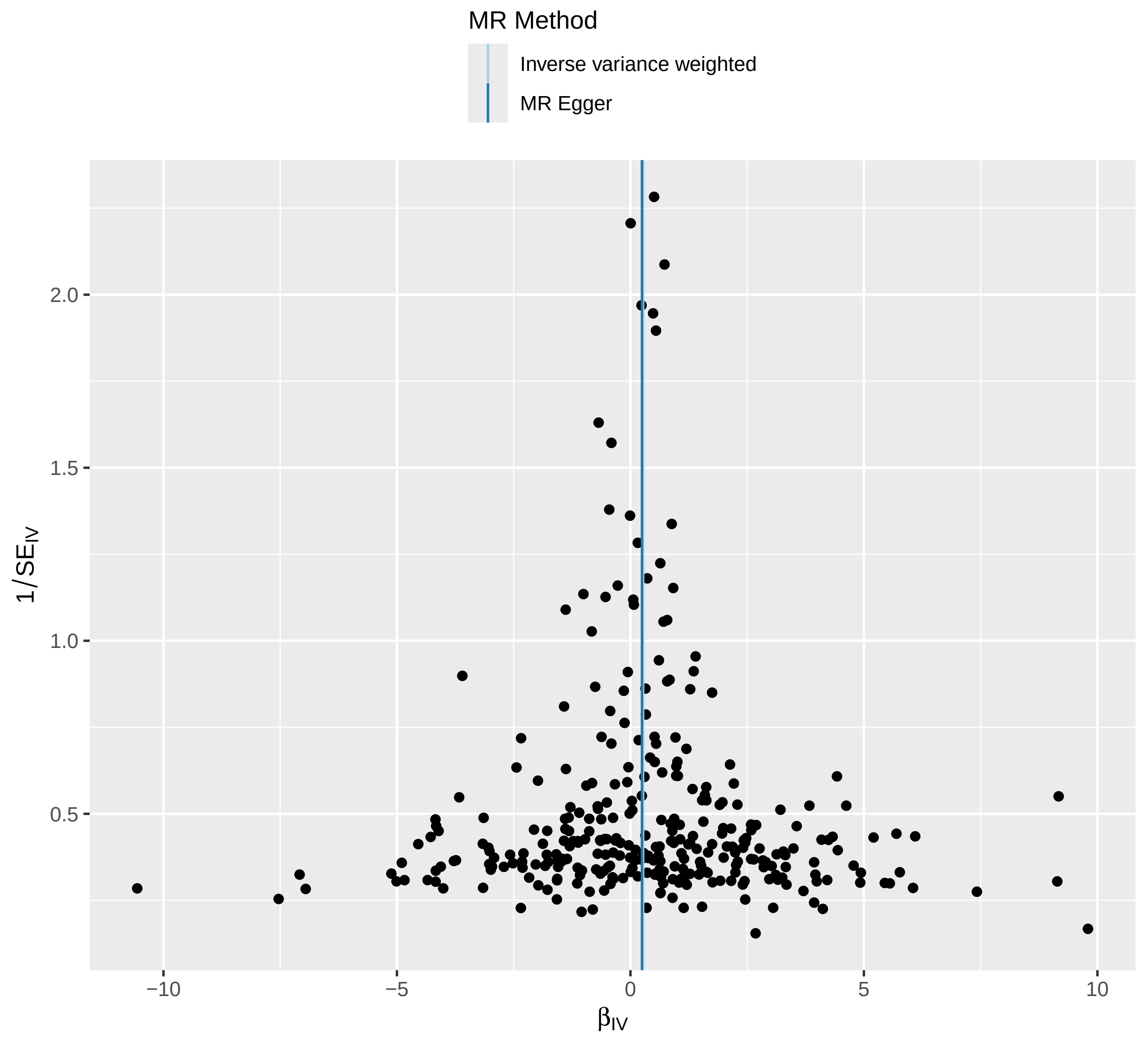
MR Method
Inverse variance weighted
MR Egger
2.0
1.5
1.0
0.5
−10
−5
0
5
10
$1/SE_{IV}$
β_{IV}

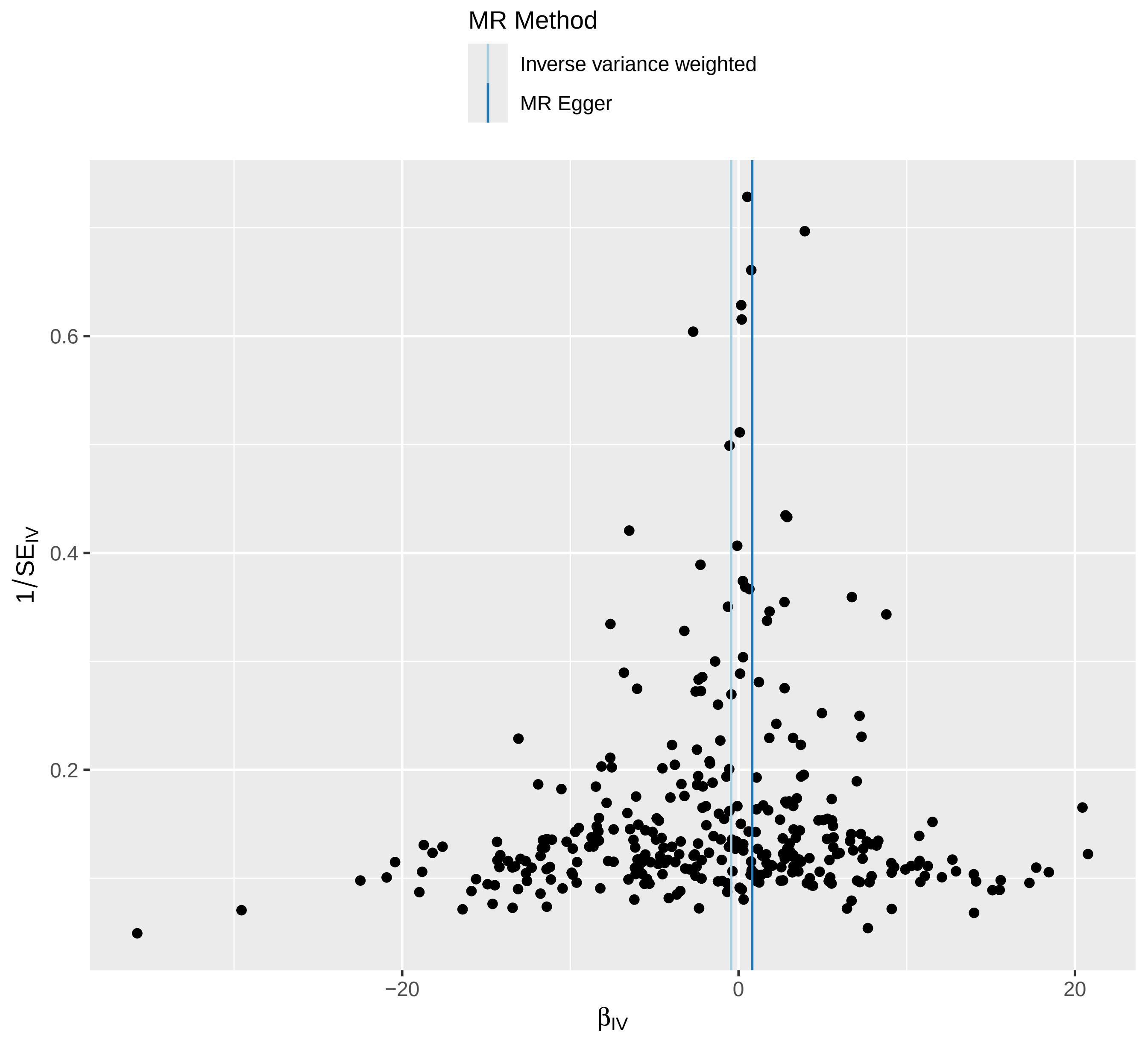

MR Method
Inverse variance weighted
MR Egger
1/SE_IV
0.2
0.4
0.6
−20
0
20
β_IV

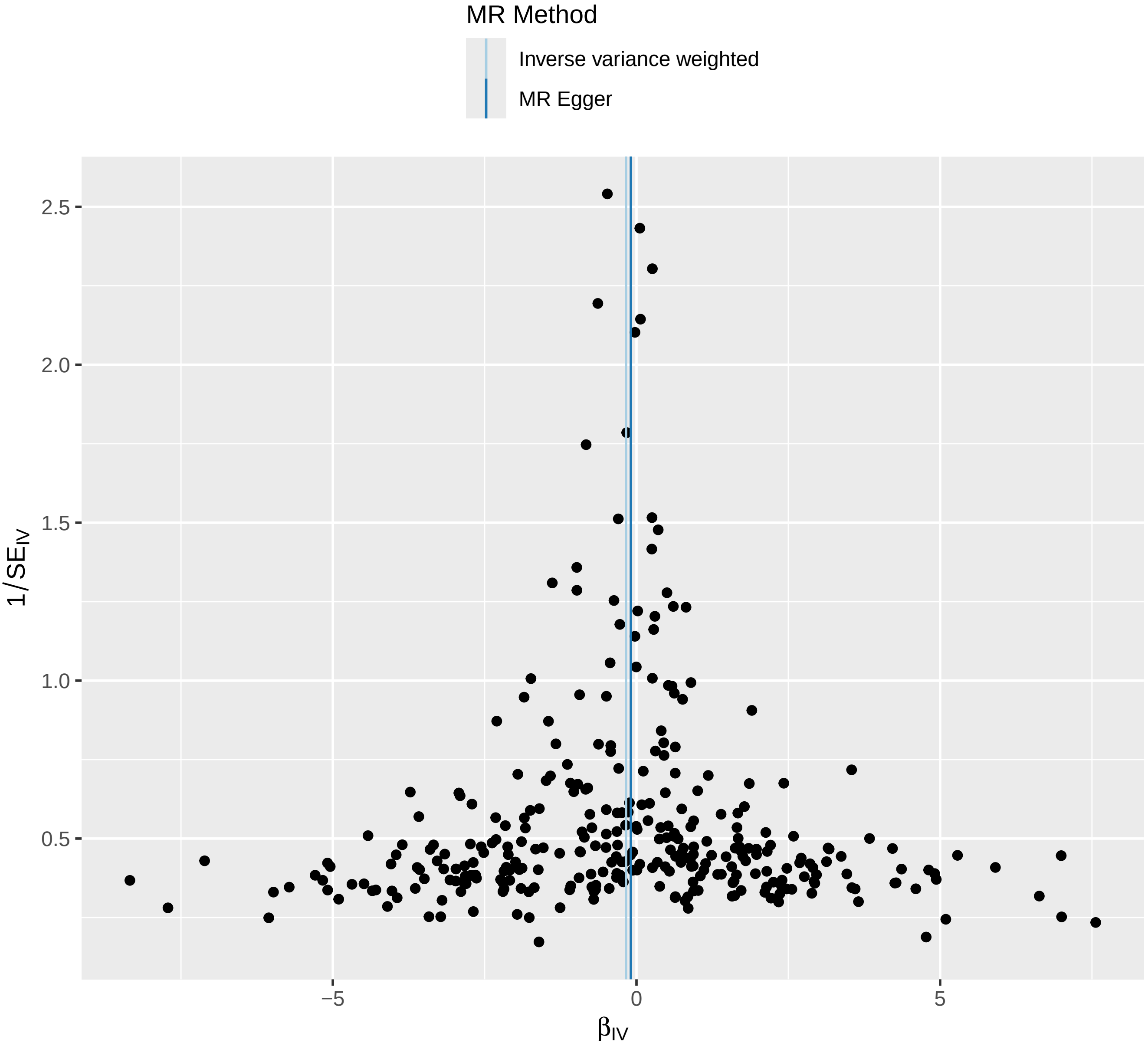

MR Method
Inverse variance weighted
MR Egger
1/SE_IV
2.5
2.0
1.5
1.0
0.5
−5
0
5
β_IV

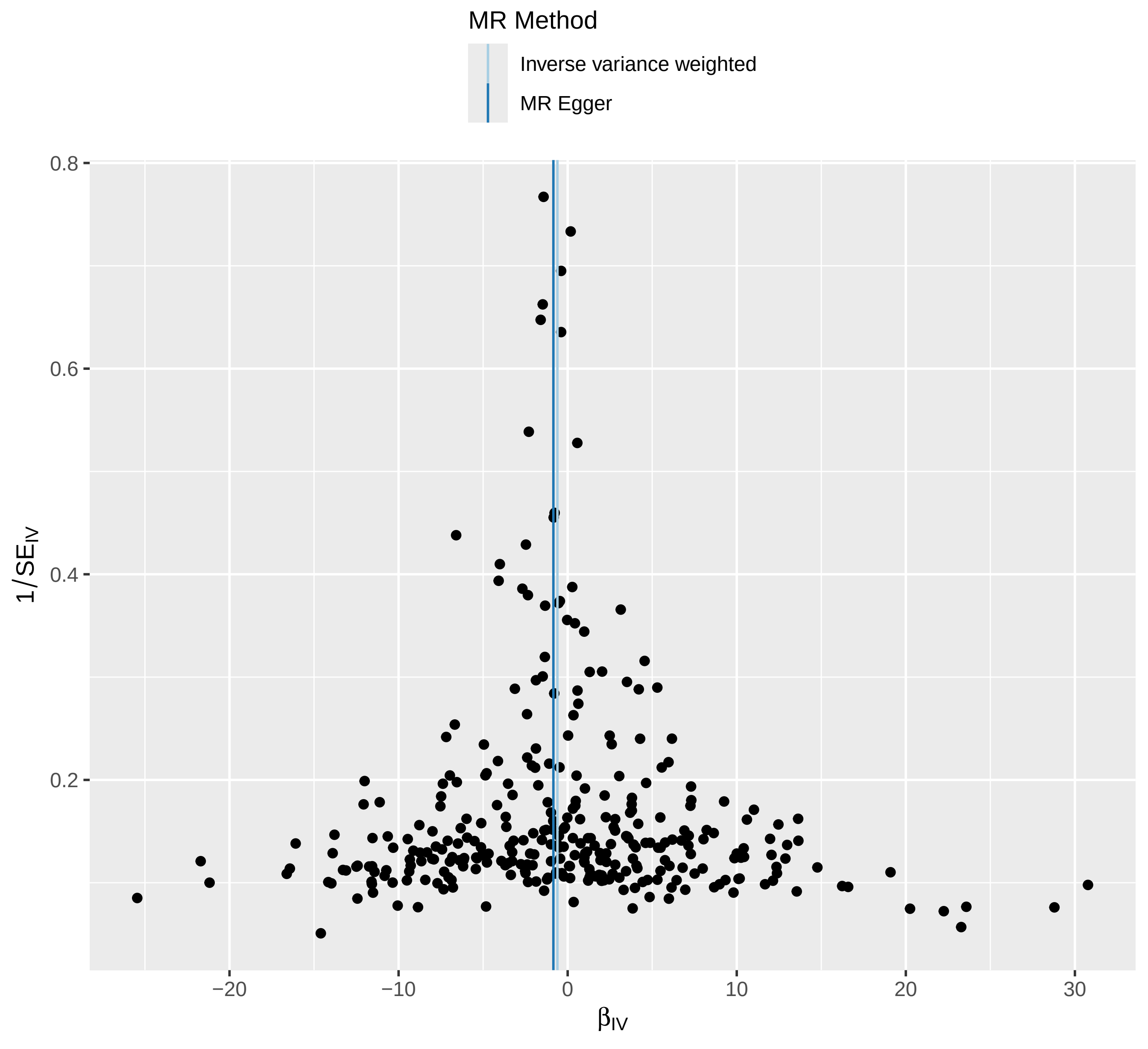

MR Method
Inverse variance weighted
MR Egger
0.8
0.6
0.4
0.2
1/SE_IV
−20
−10
0
10
20
30
β_IV

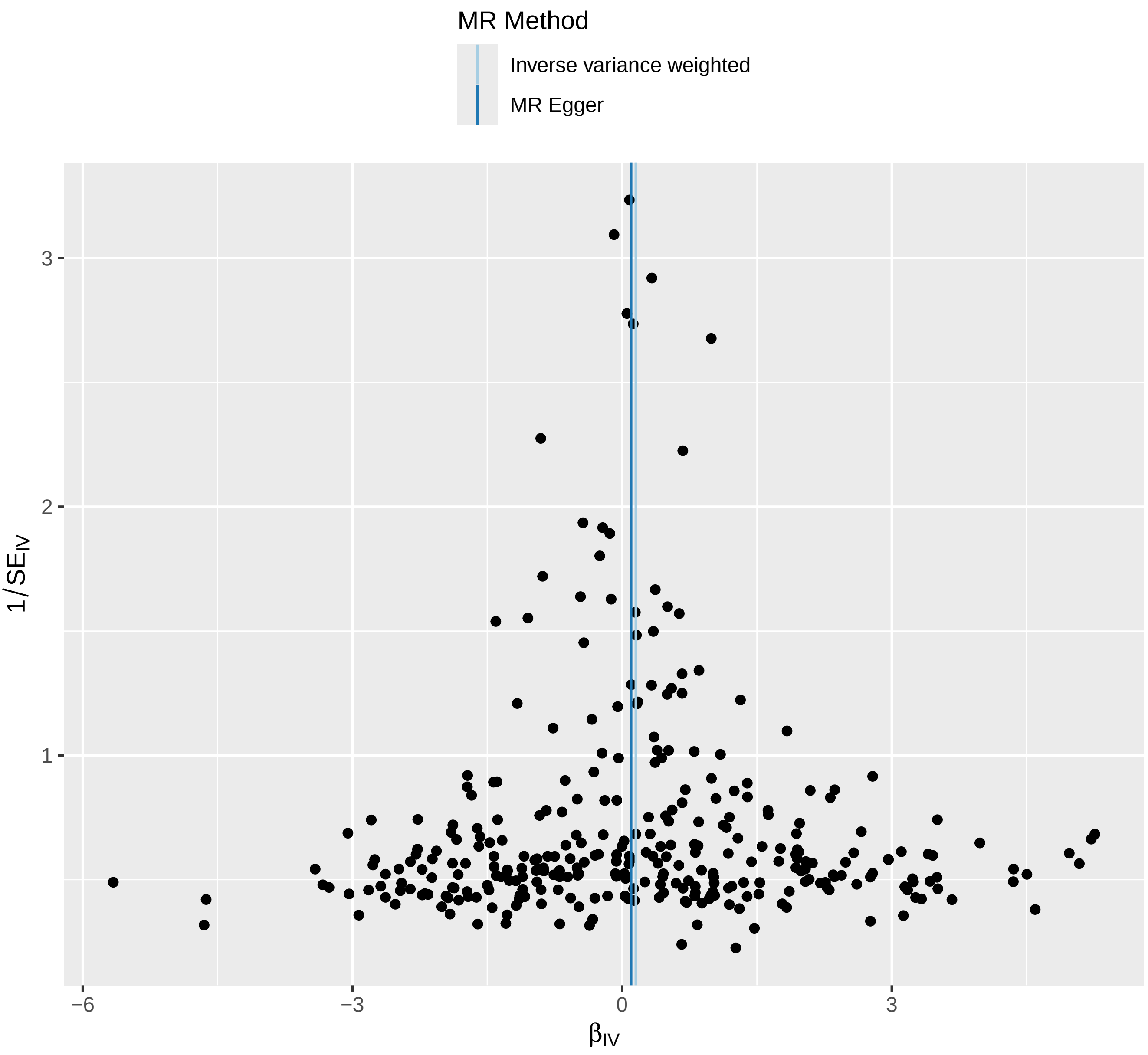

MR Method
Inverse variance weighted
MR Egger
1/SE_IV
3
2
1
−6
−3
0
3
β_IV

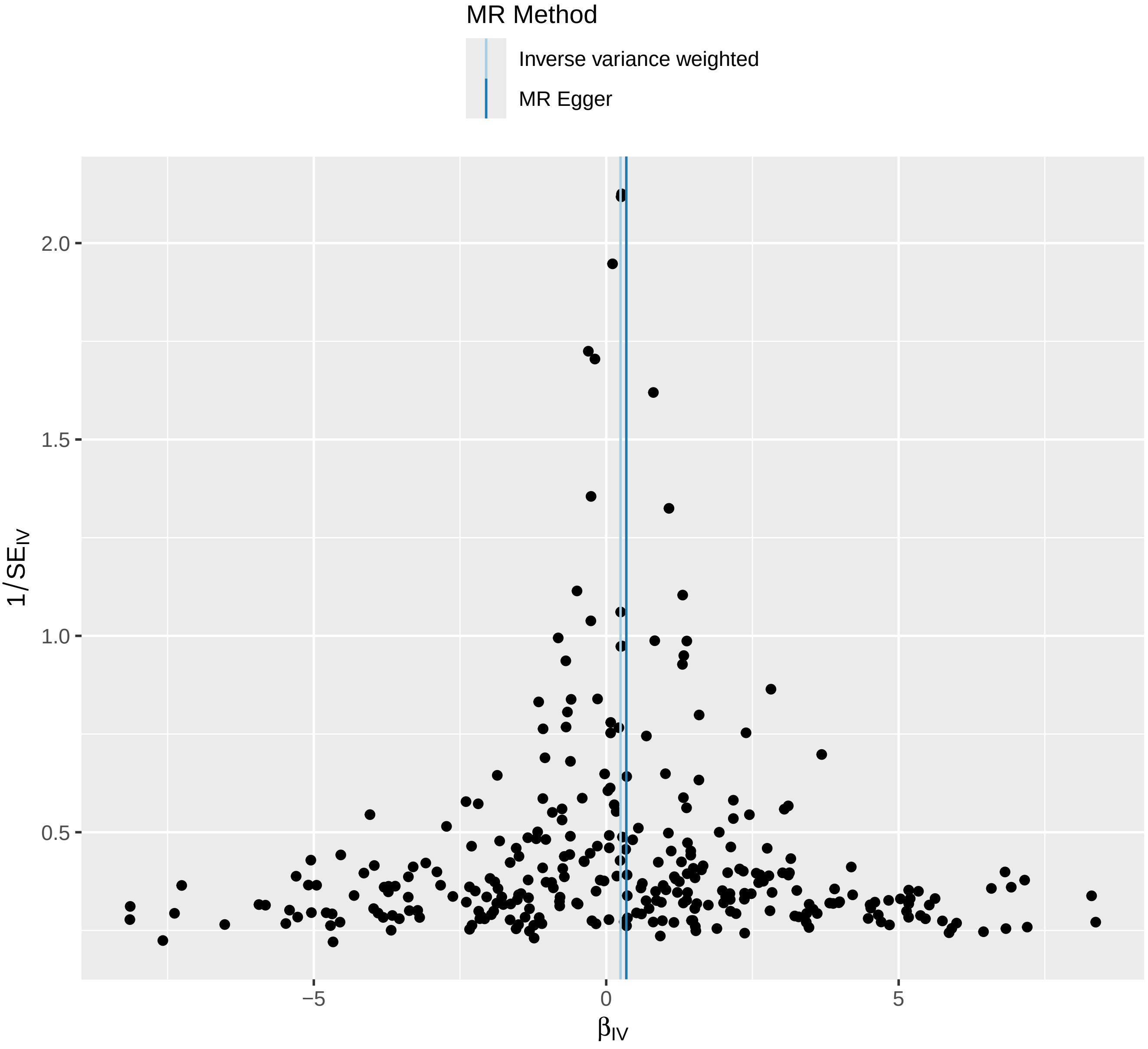
MR Method
Inverse variance weighted
MR Egger
2.0
1.5
1.0
0.5
$1/SE_{IV}$
−5
0
5
β_{IV}

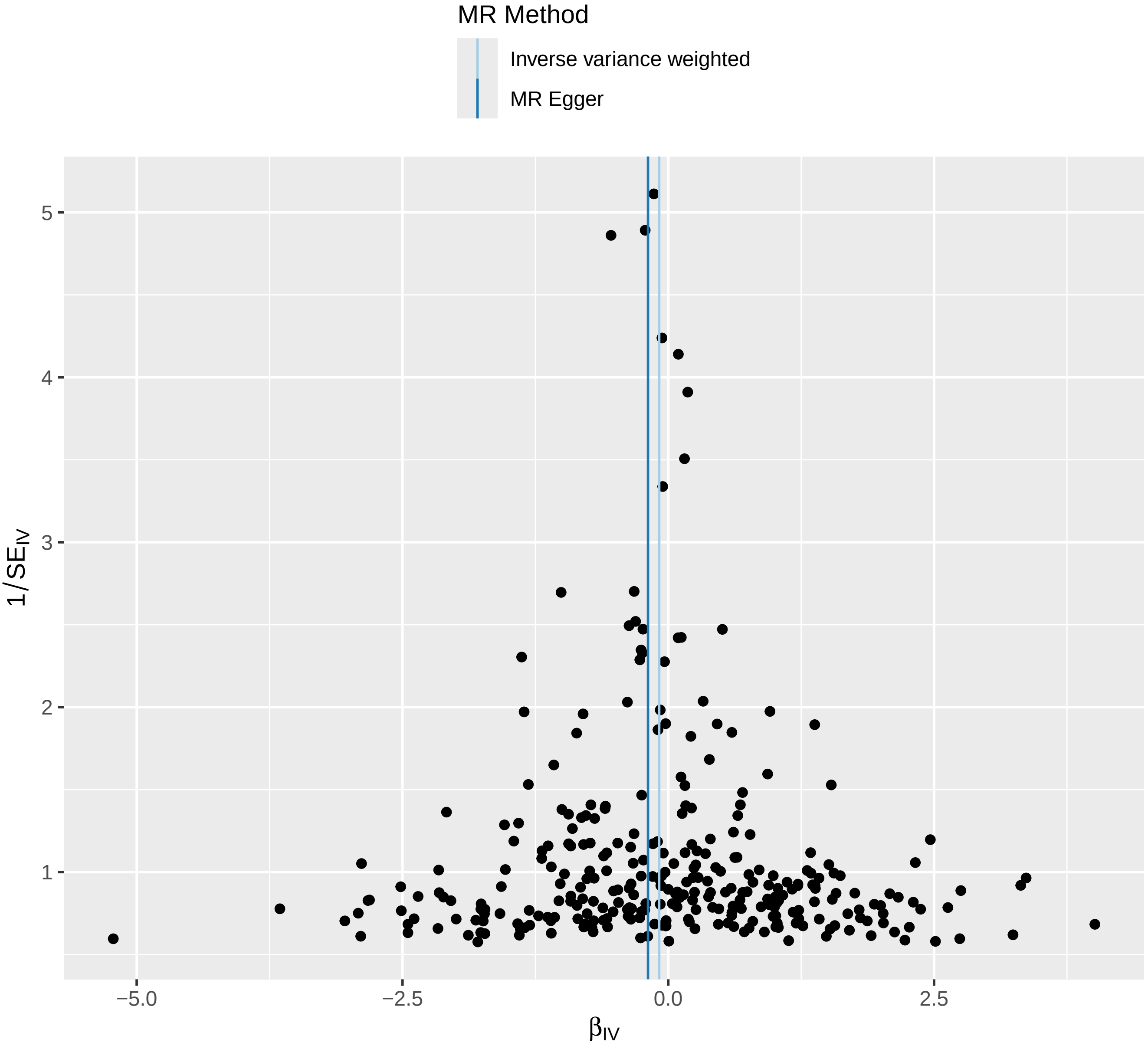
MR Method
Inverse variance weighted
MR Egger
1/SE_IV
β_IV
5
4
3
2
1
−5.0
−2.5
0.0
2.5

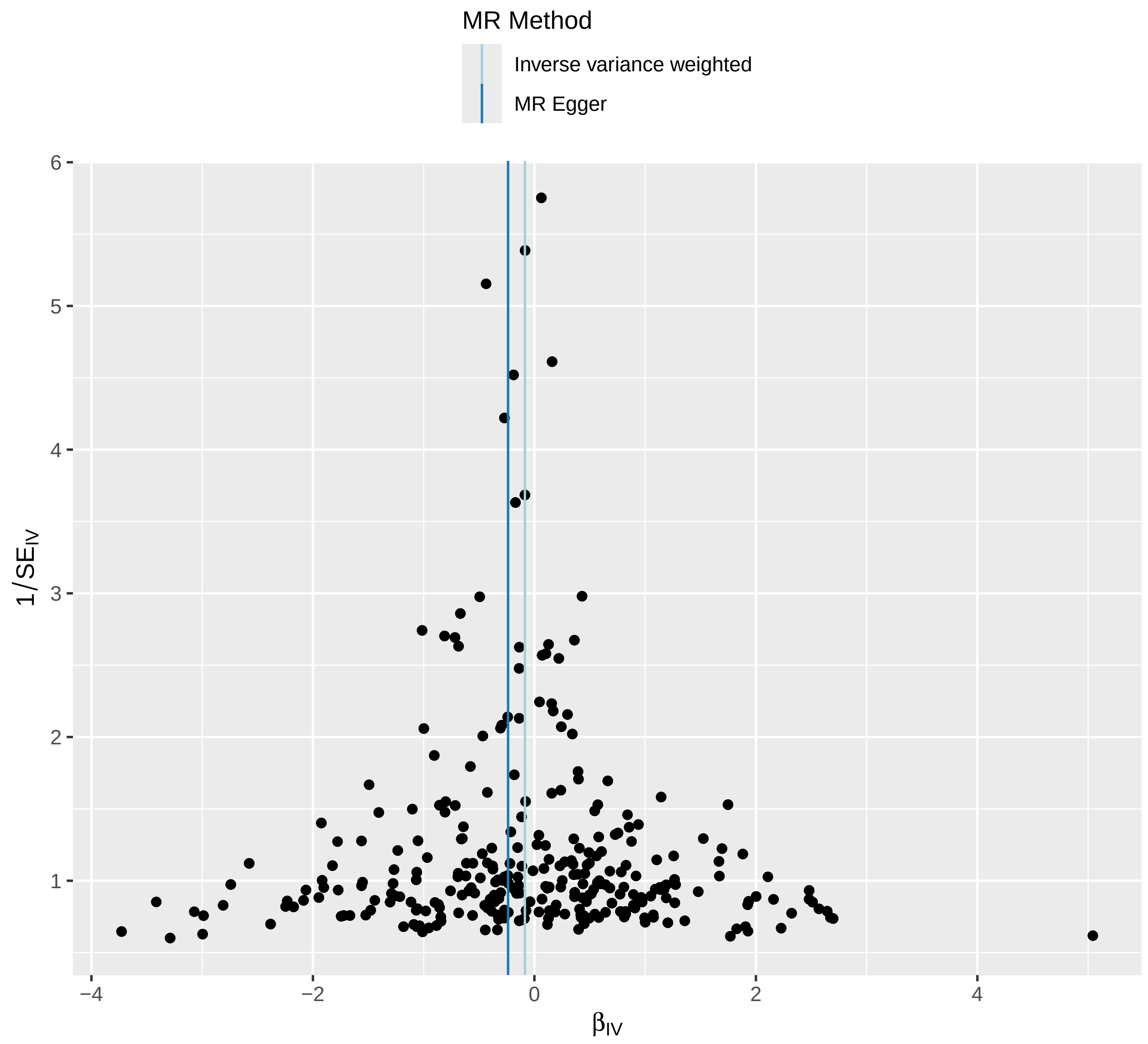
MR Method
Inverse variance weighted
MR Egger
6
5
4
3
2
1
1/SE_IV
−4
−2
0
2
4
β_IV

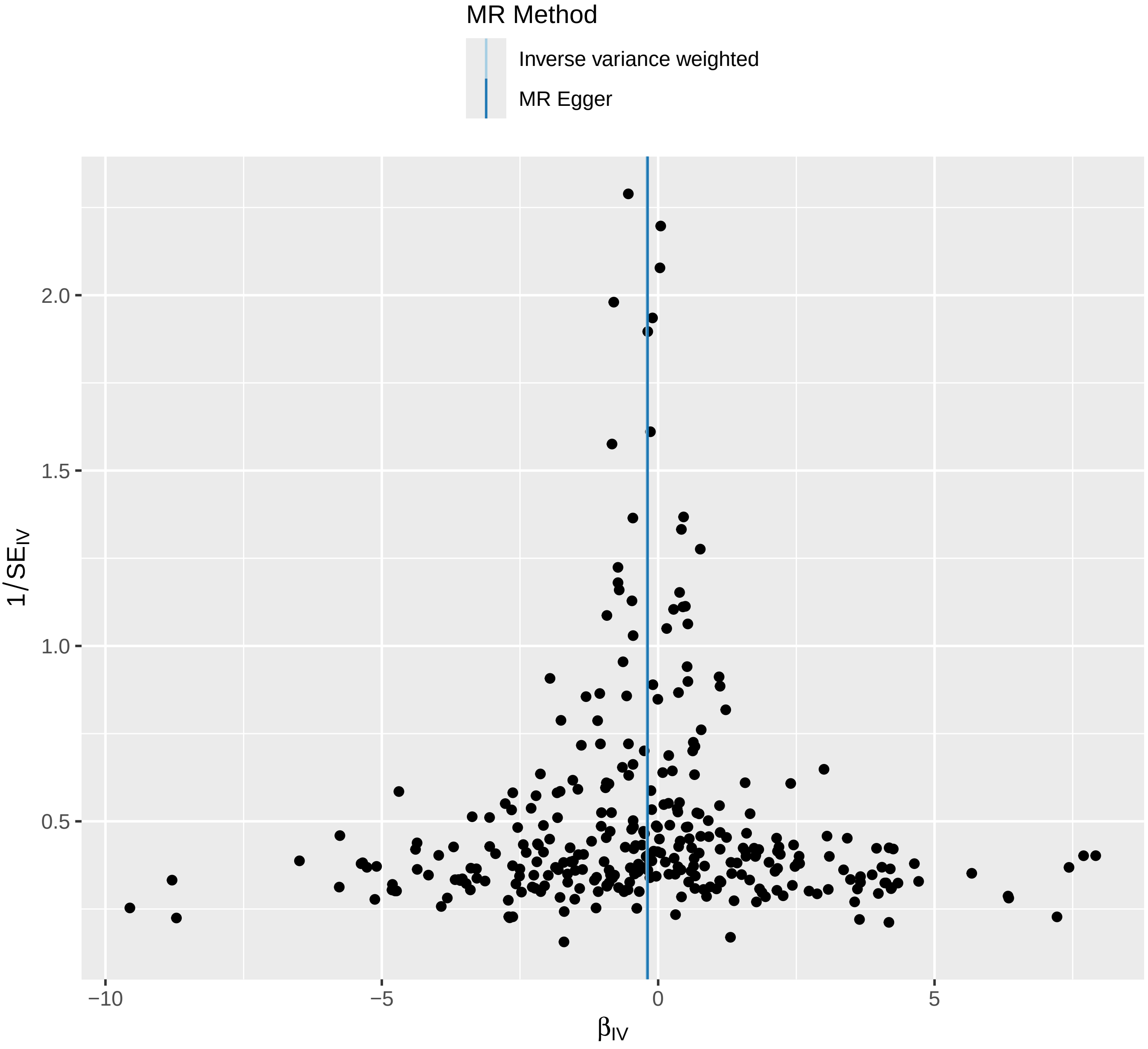
MR Method
Inverse variance weighted
MR Egger
1/SE_IV
β_IV
2.0
1.5
1.0
0.5
−10
−5
0
5

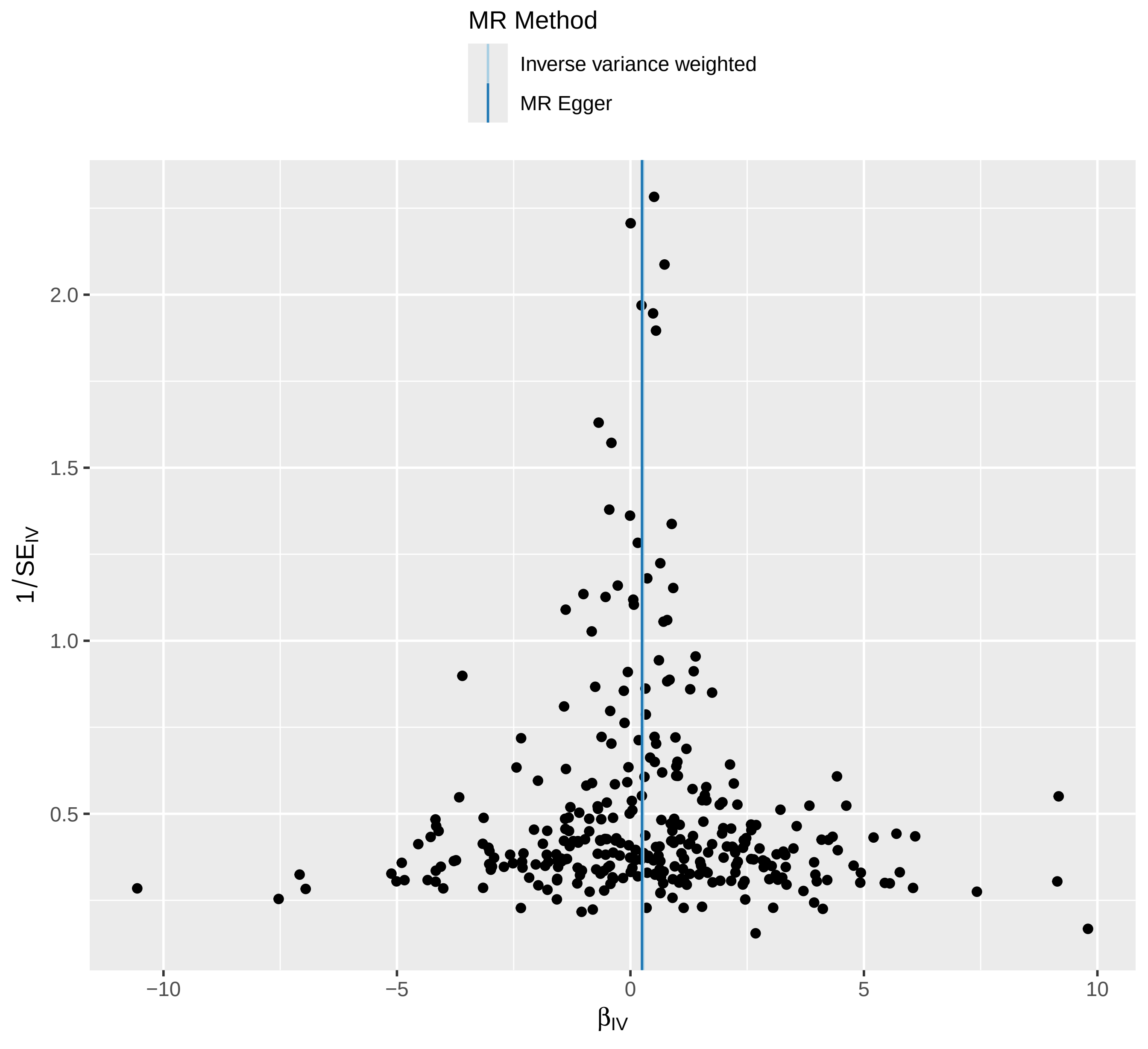
MR Method
Inverse variance weighted
MR Egger
2.0
1.5
1.0
0.5
$1/SE_{IV}$
−10
−5
0
5
10
β_{IV}

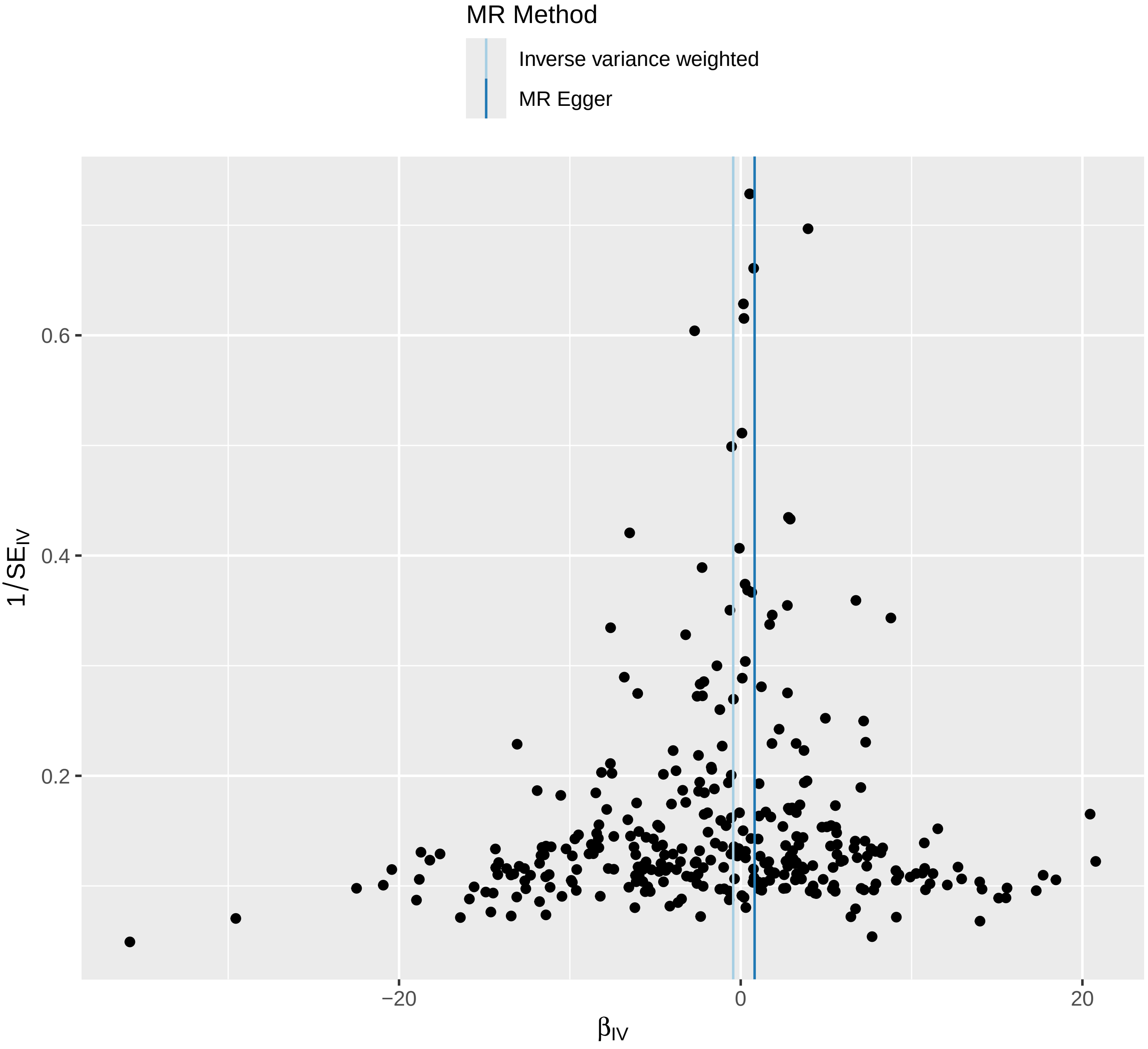
MR Method
Inverse variance weighted
MR Egger
0.6
0.4
0.2
1/SE_IV
−20
0
20
β_IV

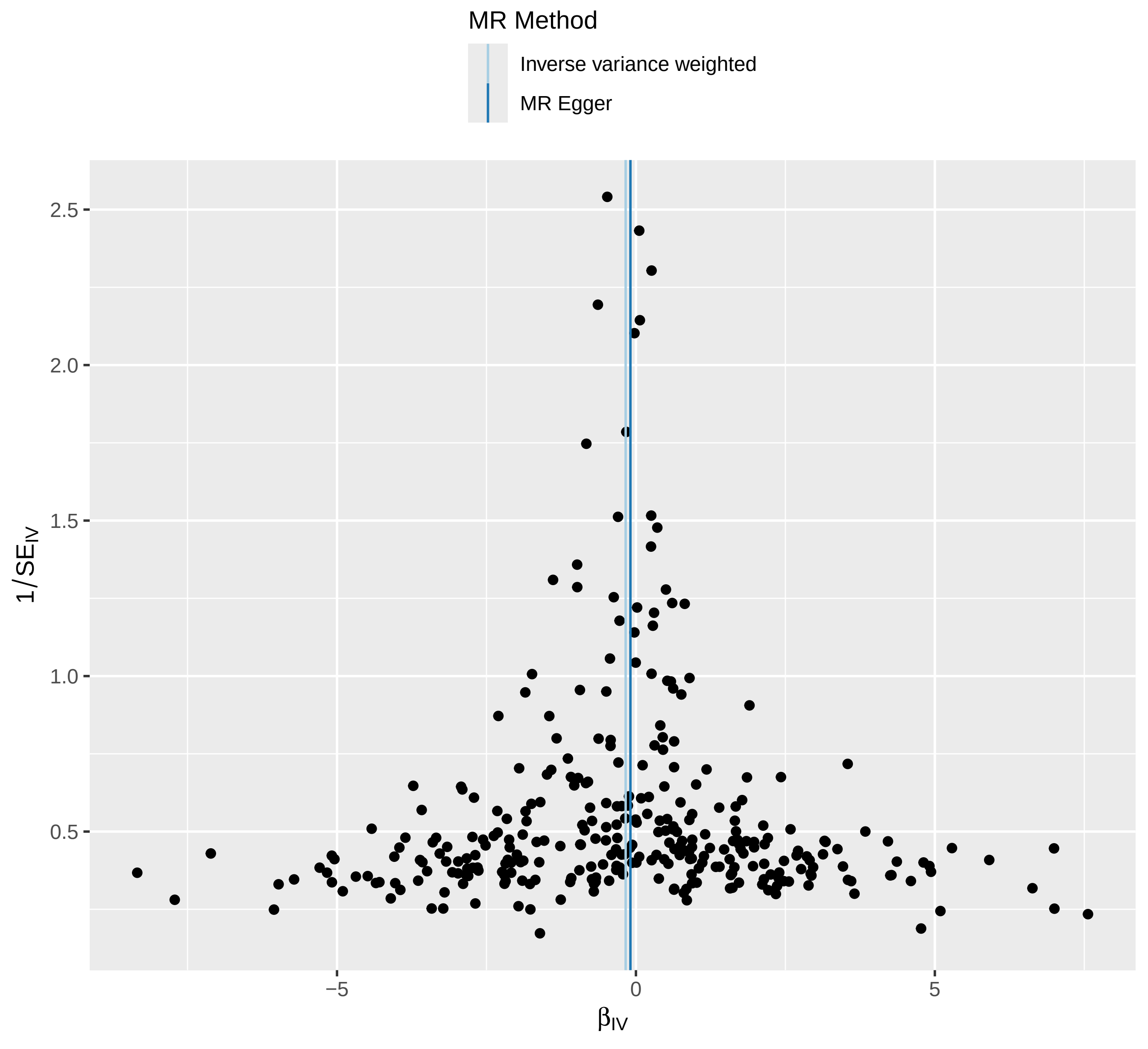
MR Method
Inverse variance weighted
MR Egger
1/SE$_{IV}$
2.5
2.0
1.5
1.0
0.5
−5
0
5
β$_{IV}$

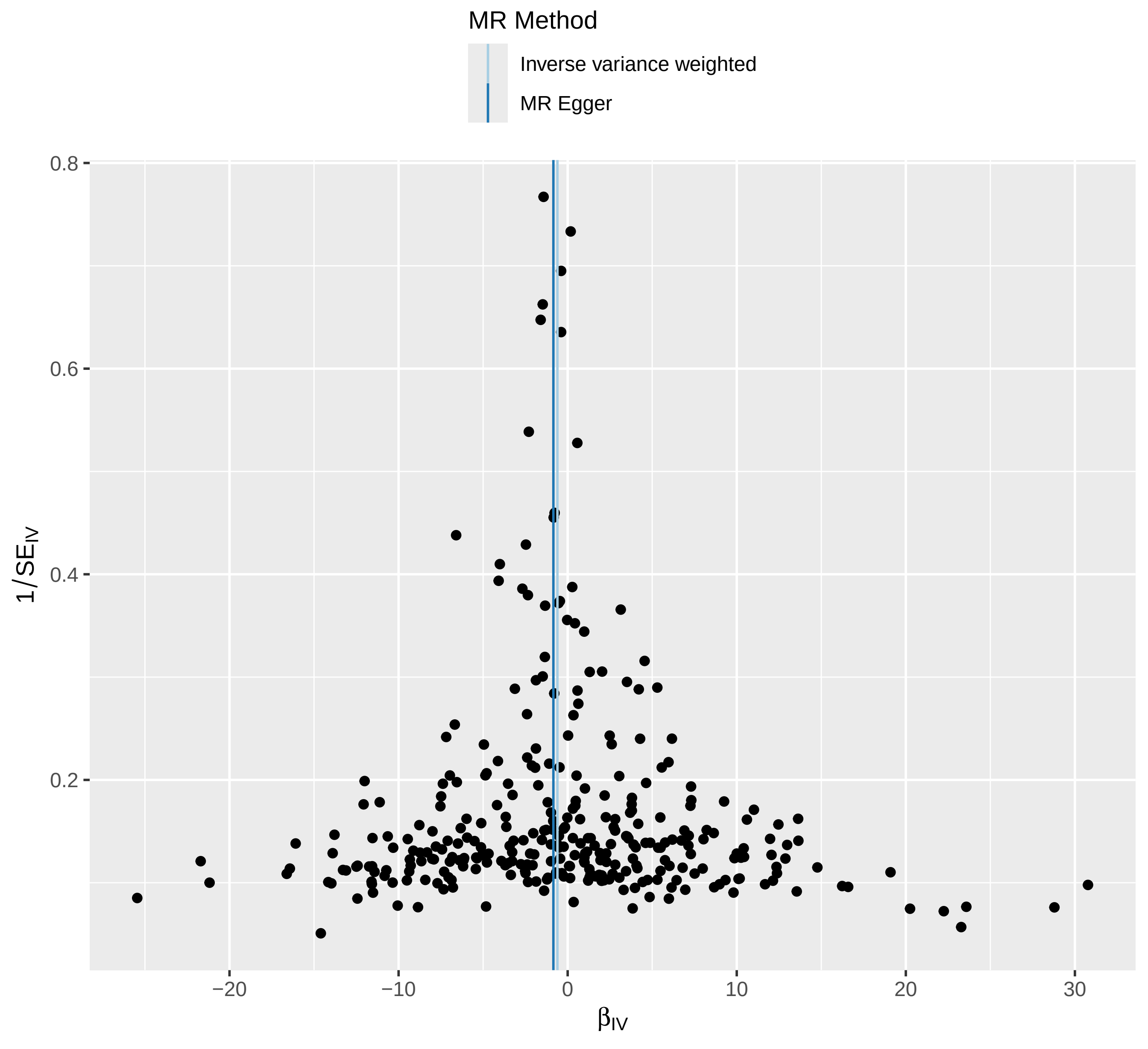

MR Method
Inverse variance weighted
MR Egger
0.8
0.6
0.4
0.2
1/SE_IV
−20
−10
0
10
20
30
β_IV

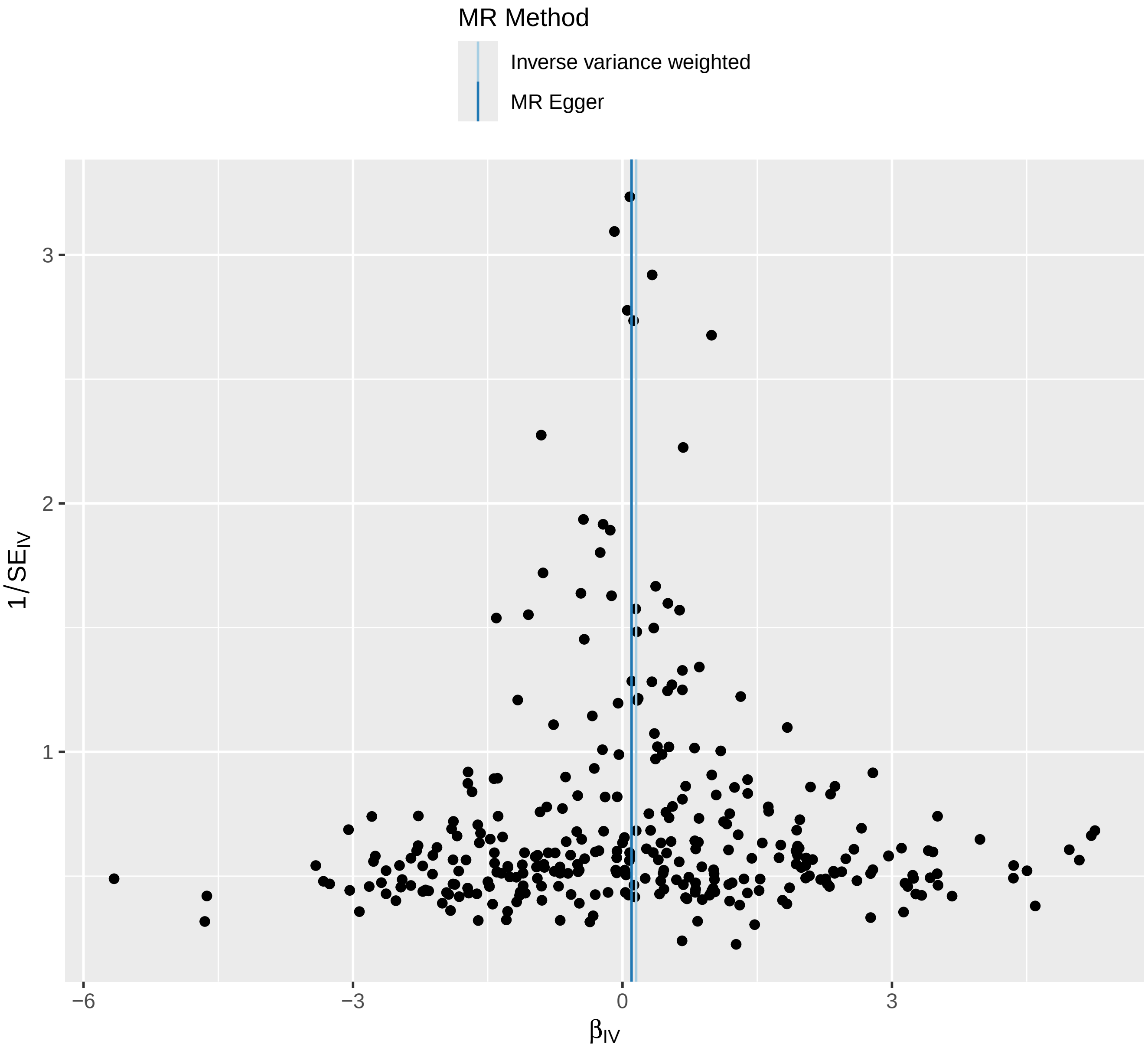

MR Method
Inverse variance weighted
MR Egger
1/SE_IV
β_IV
3
2
1
−6
−3
0
3

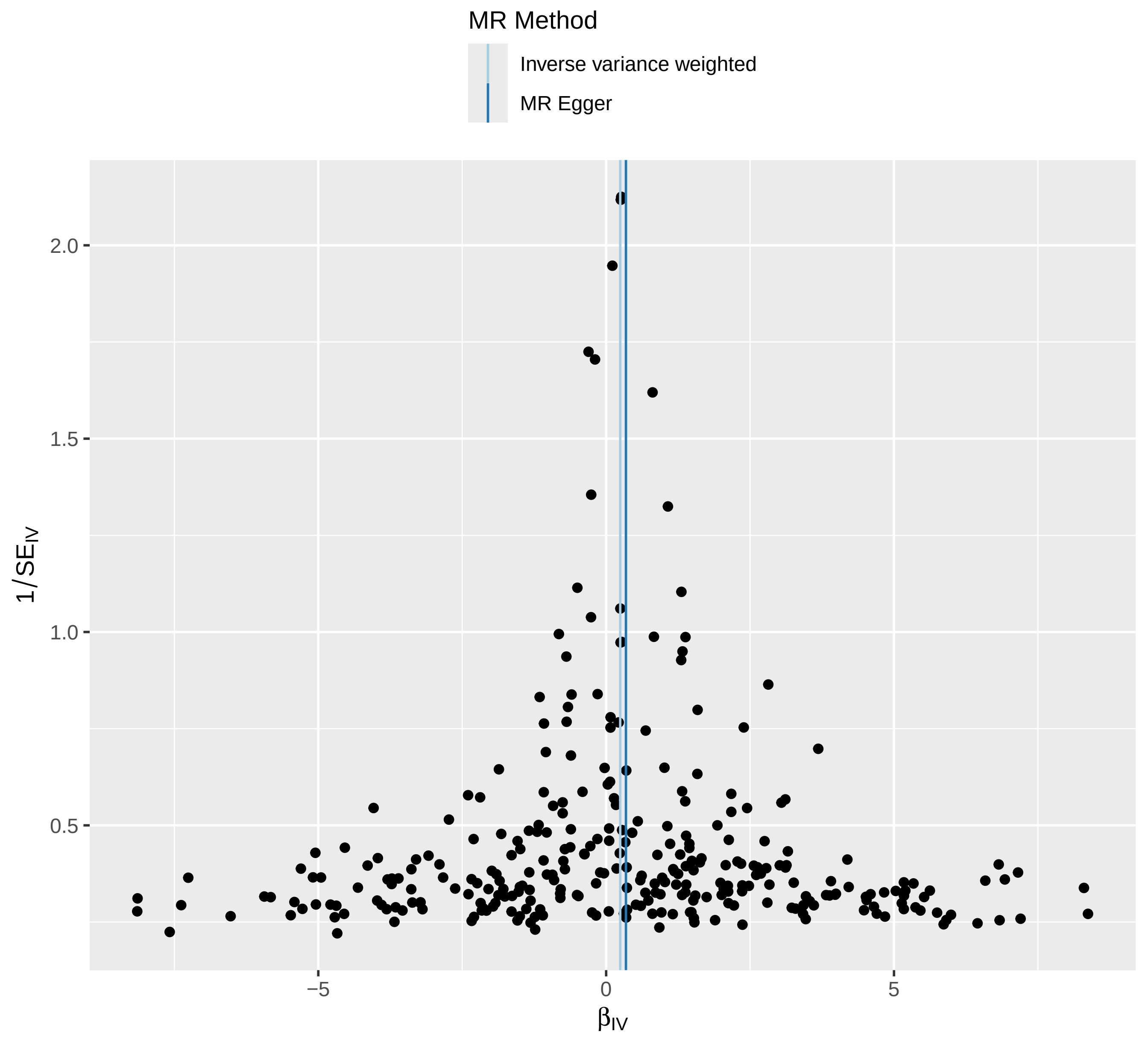
MR Method
Inverse variance weighted
MR Egger
1/SE_IV
2.0
1.5
1.0
0.5
−5
0
5
β_IV

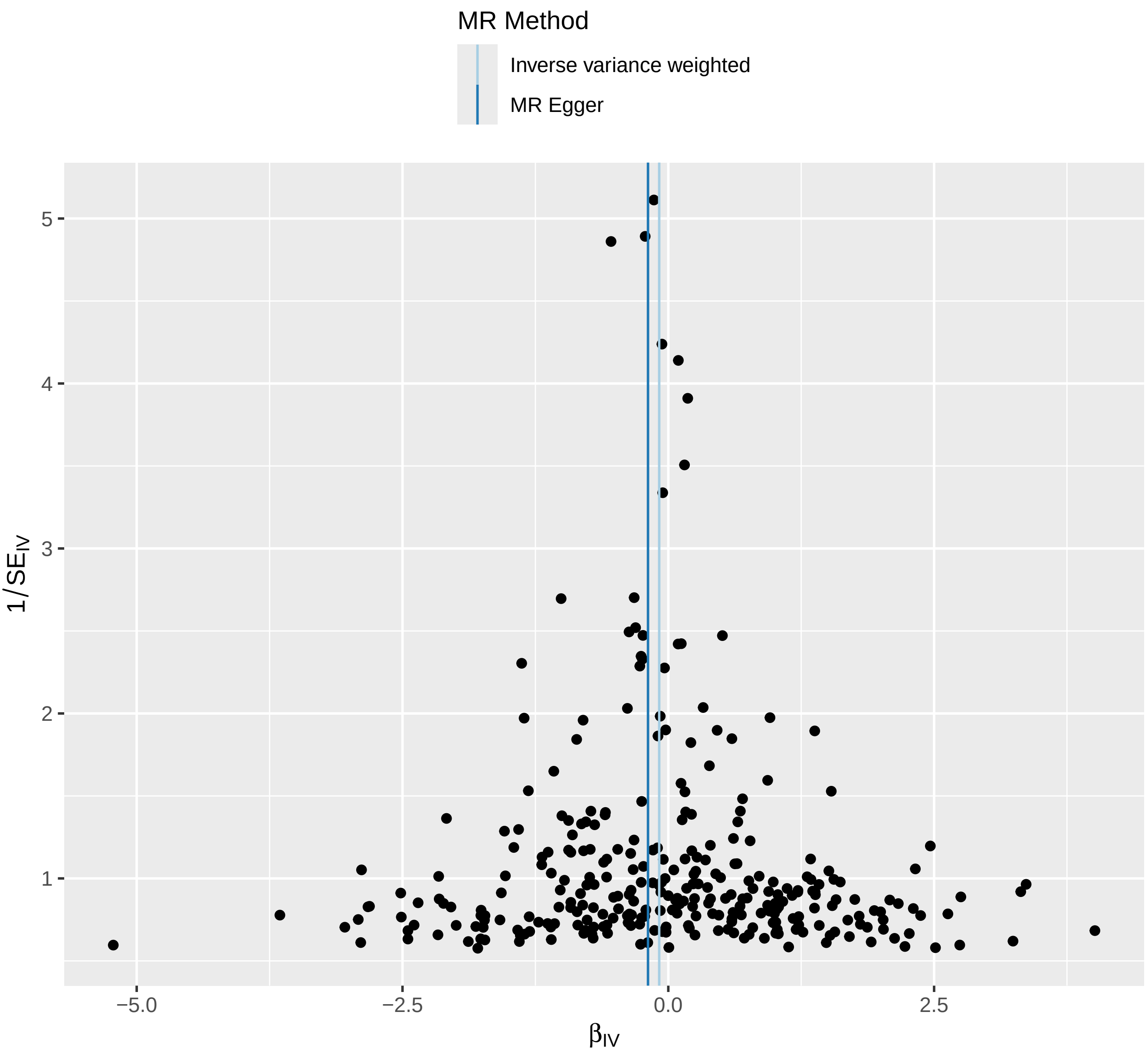

MR Method
Inverse variance weighted
MR Egger
5
4
3
2
1
$1/SE_{IV}$
−5.0
−2.5
0.0
2.5
β_{IV}

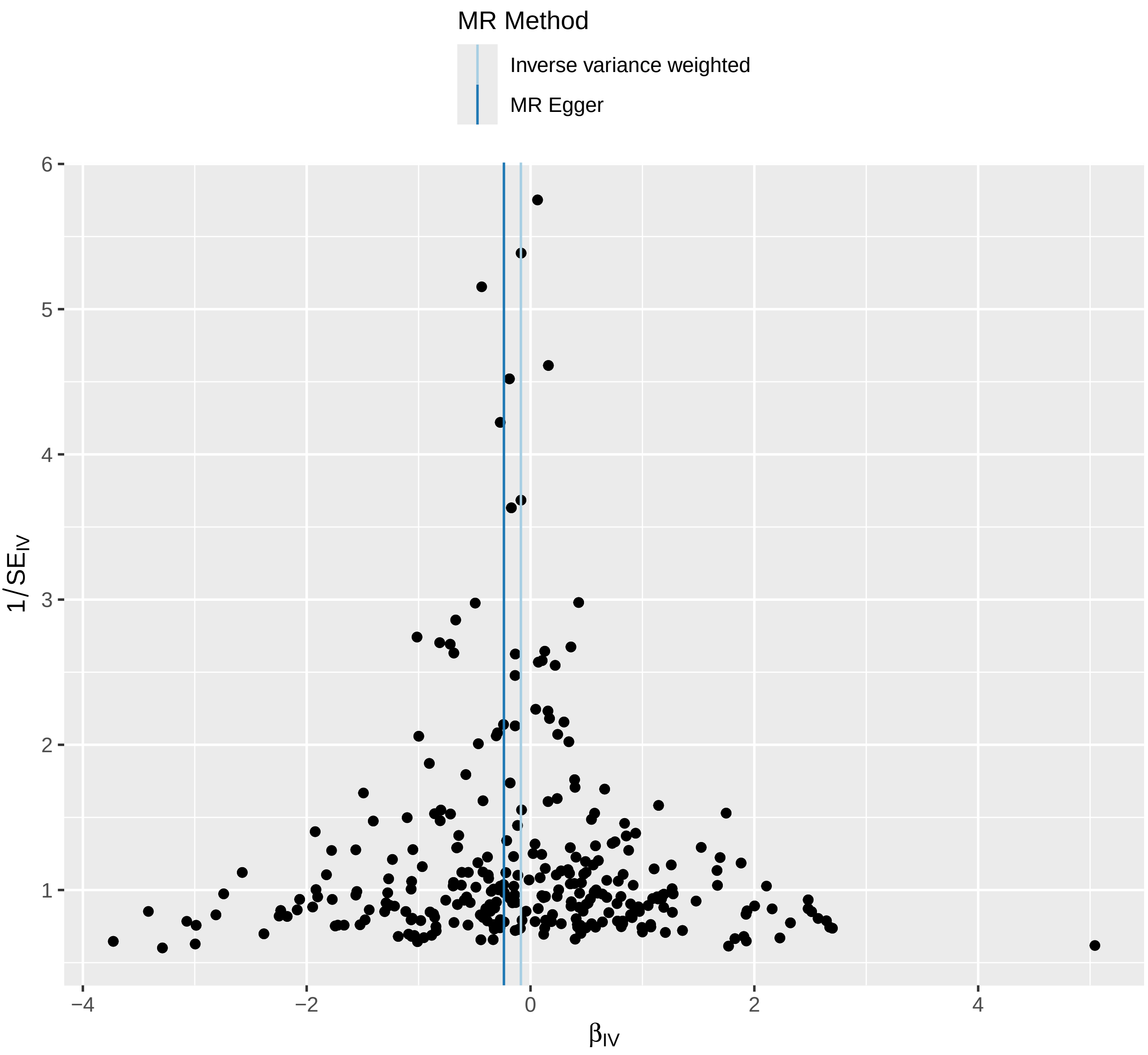
MR Method
Inverse variance weighted
MR Egger
1/SE_IV
β_IV
6
5
4
3
2
1
−4
−2
0
2
4

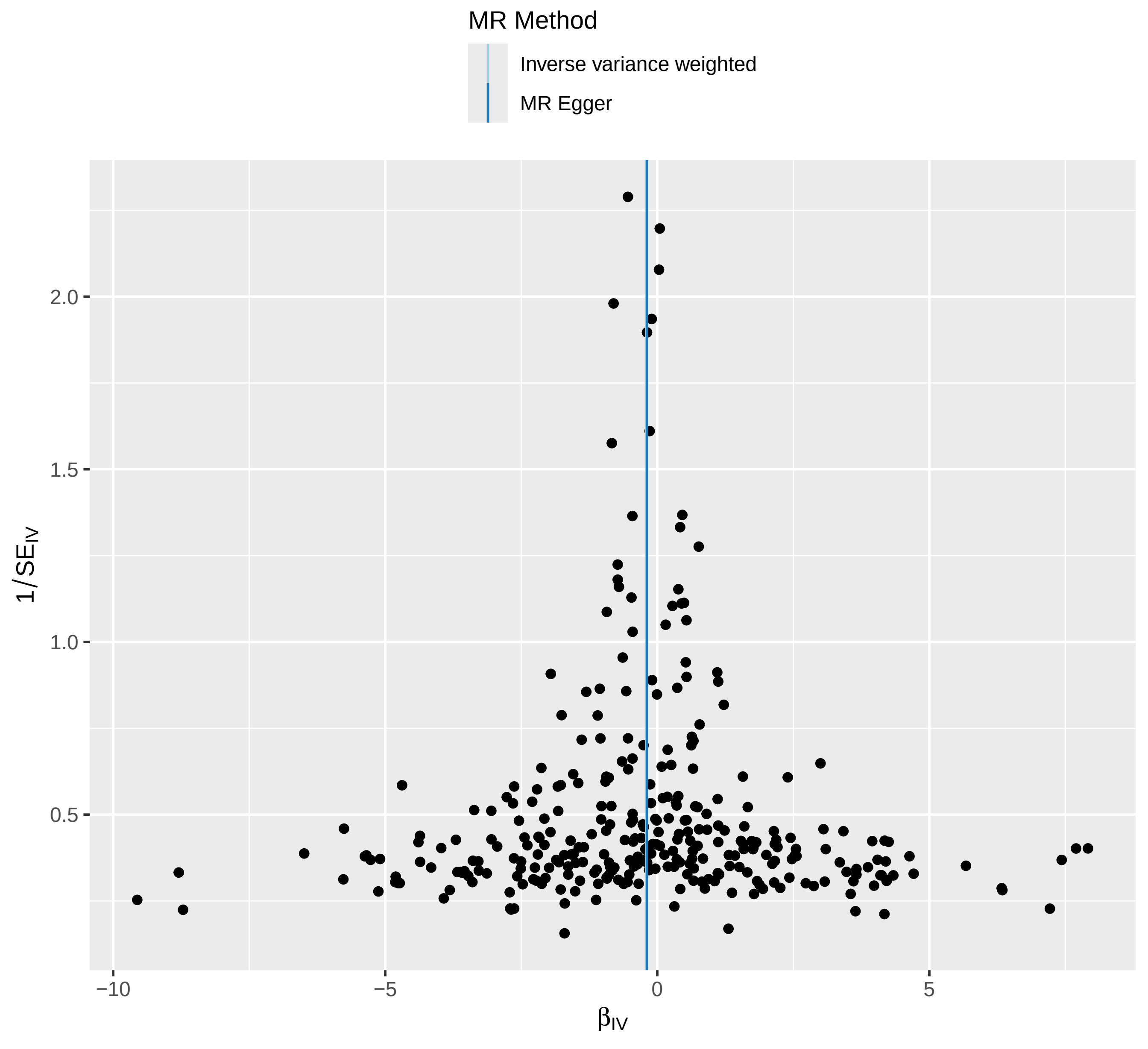
MR Method
Inverse variance weighted
MR Egger
1/SE_IV
2.0
1.5
1.0
0.5
−10
−5
0
5
β_IV

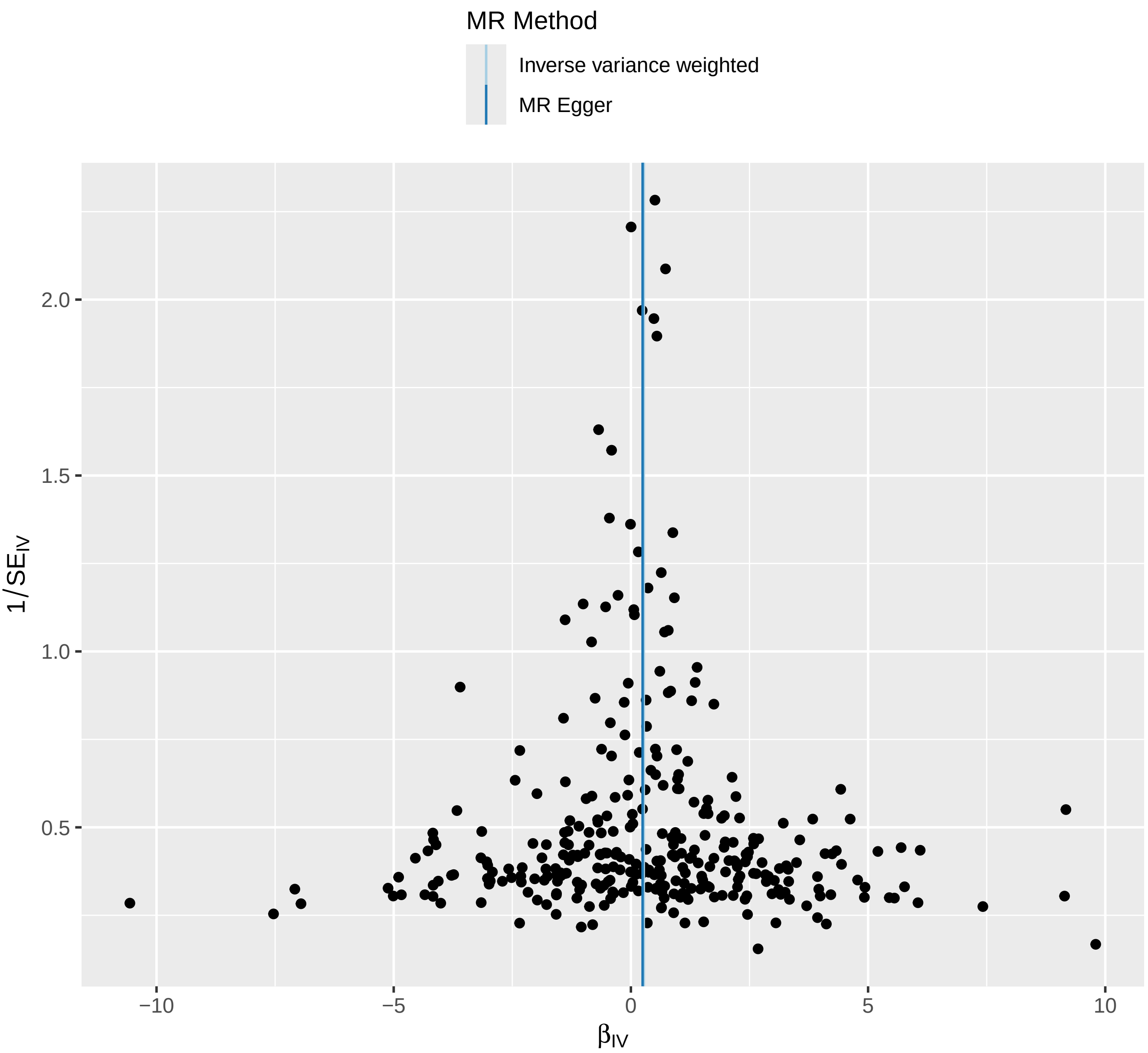

MR Method
Inverse variance weighted
MR Egger
1/SE$_{IV}$
2.0
1.5
1.0
0.5
−10
−5
0
5
10
β$_{IV}$

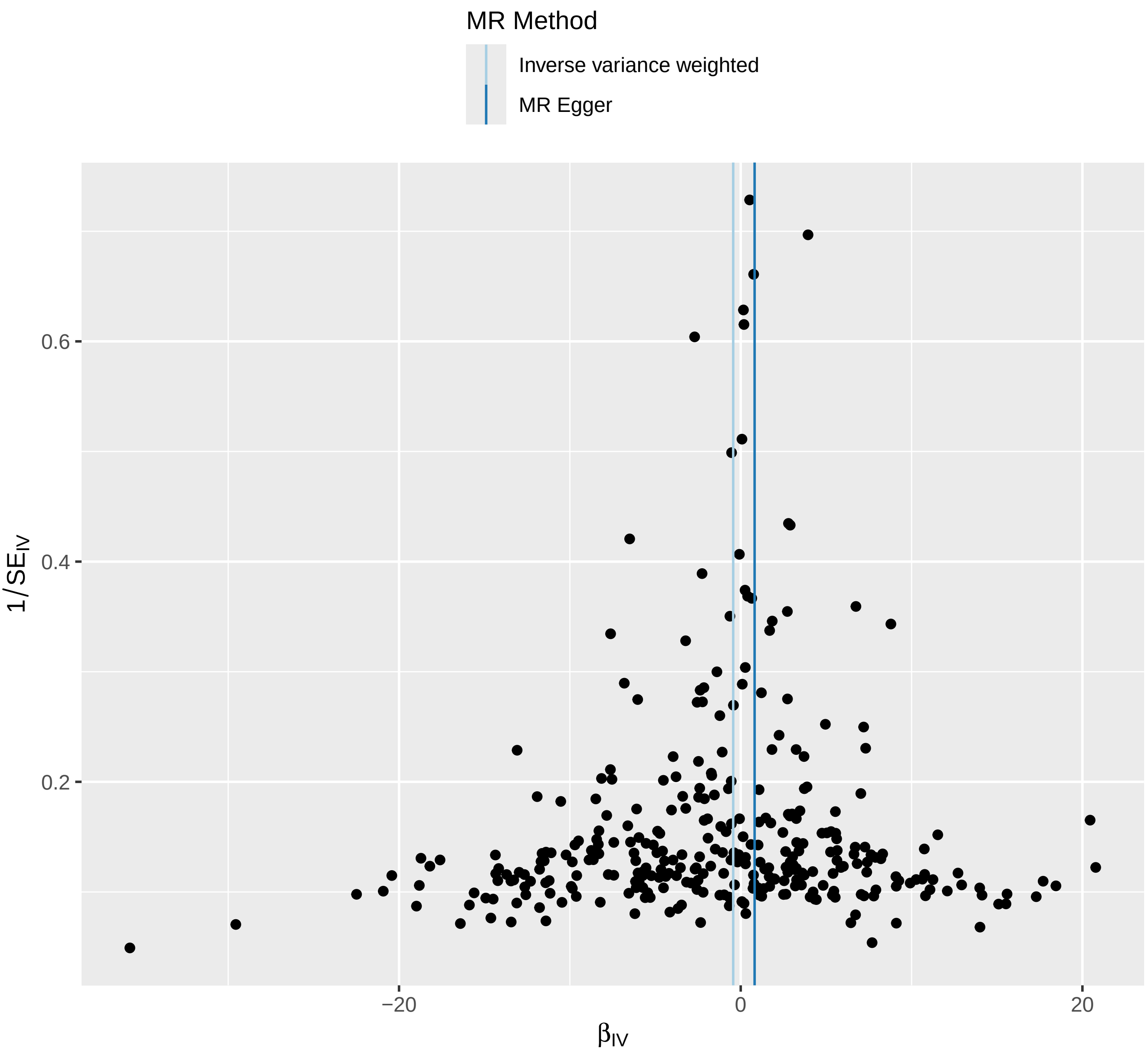
MR Method
Inverse variance weighted
MR Egger
1/SE_IV
0.2
0.4
0.6
β_IV
−20
0
20

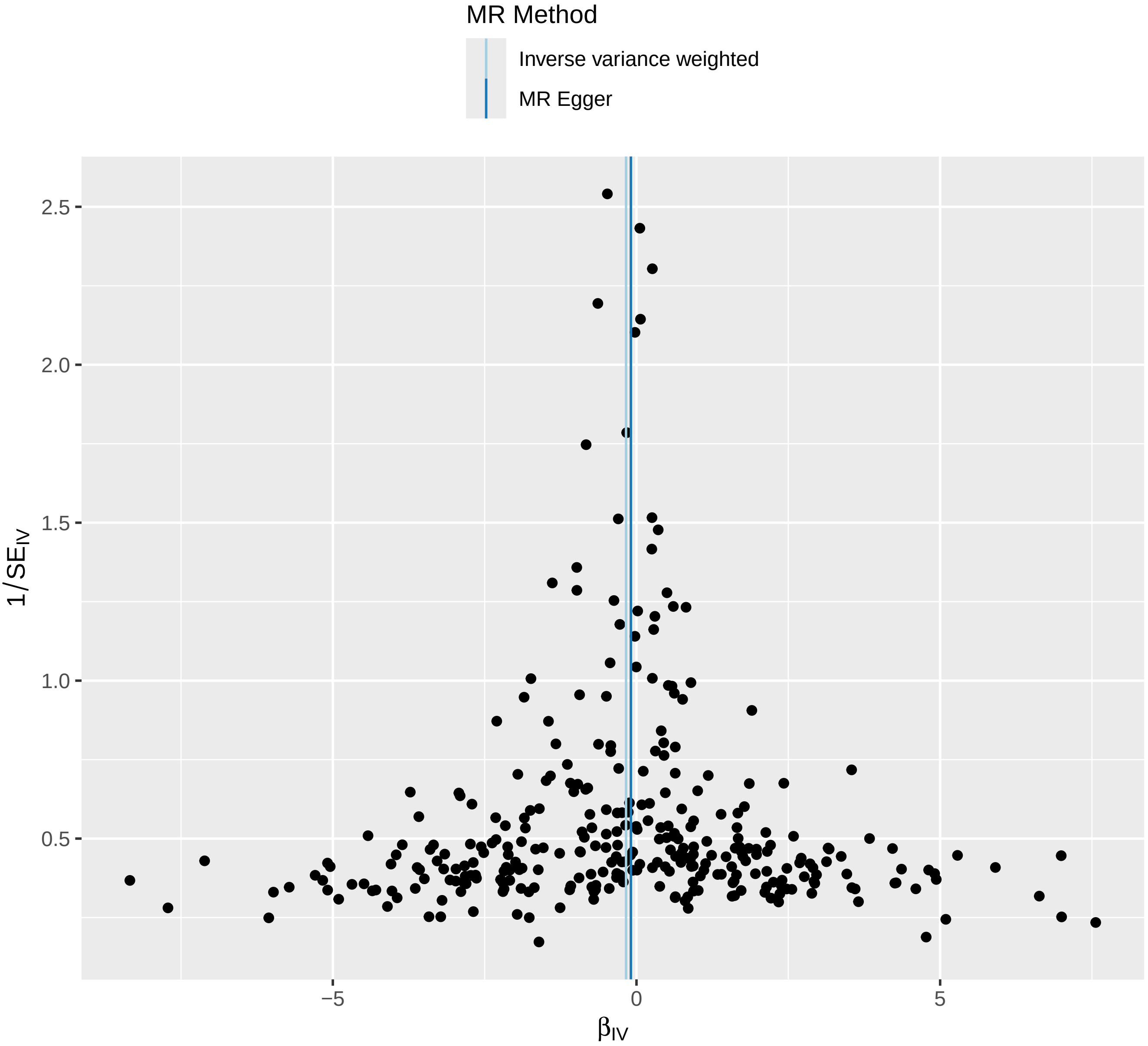
MR Method
Inverse variance weighted
MR Egger
1/SE$_{IV}$
2.5
2.0
1.5
1.0
0.5
−5
0
5
β$_{IV}$

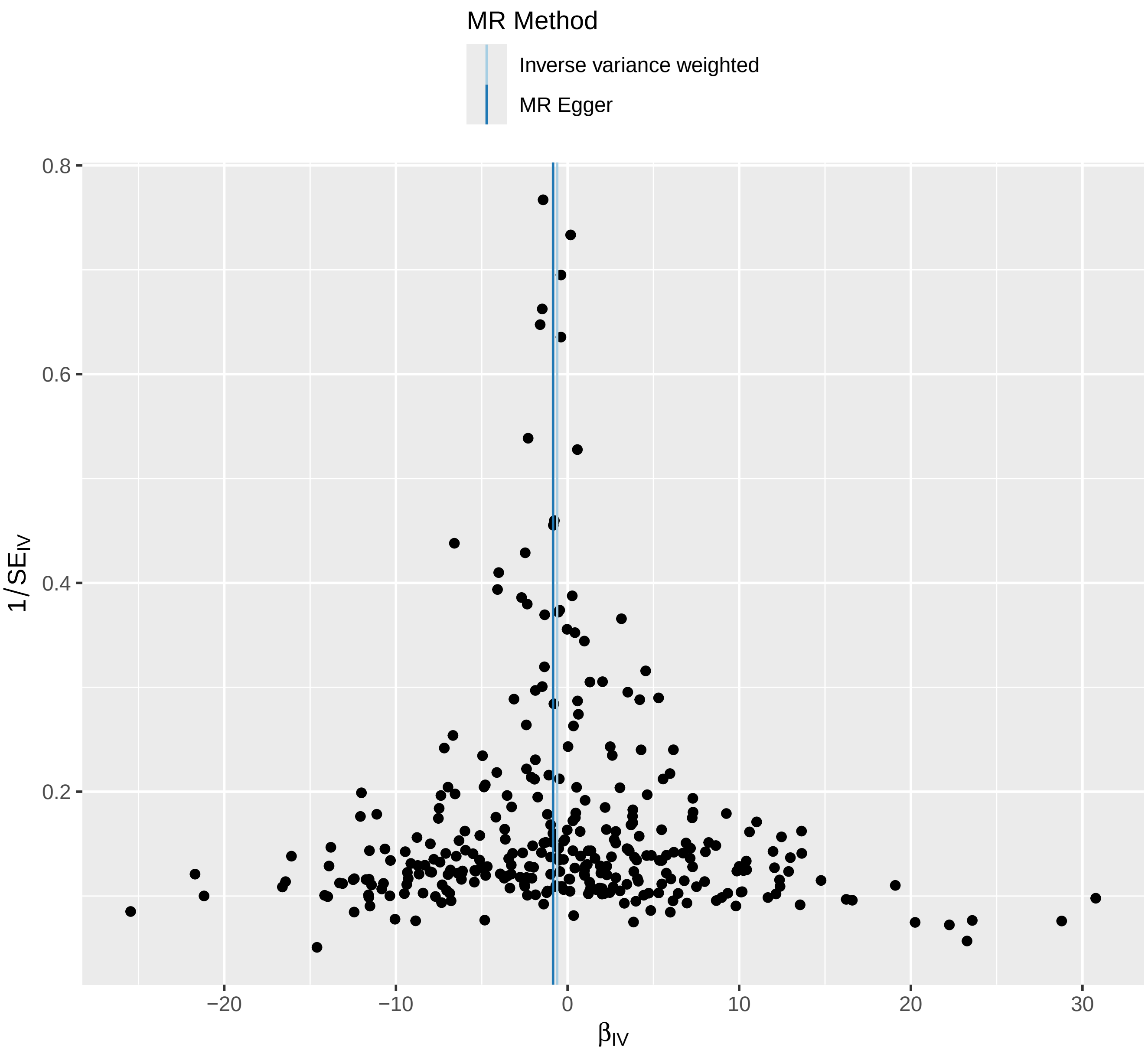

MR Method
Inverse variance weighted
MR Egger
0.8
0.6
0.4
0.2
1/SE_IV
−20
−10
0
10
20
30
β_IV

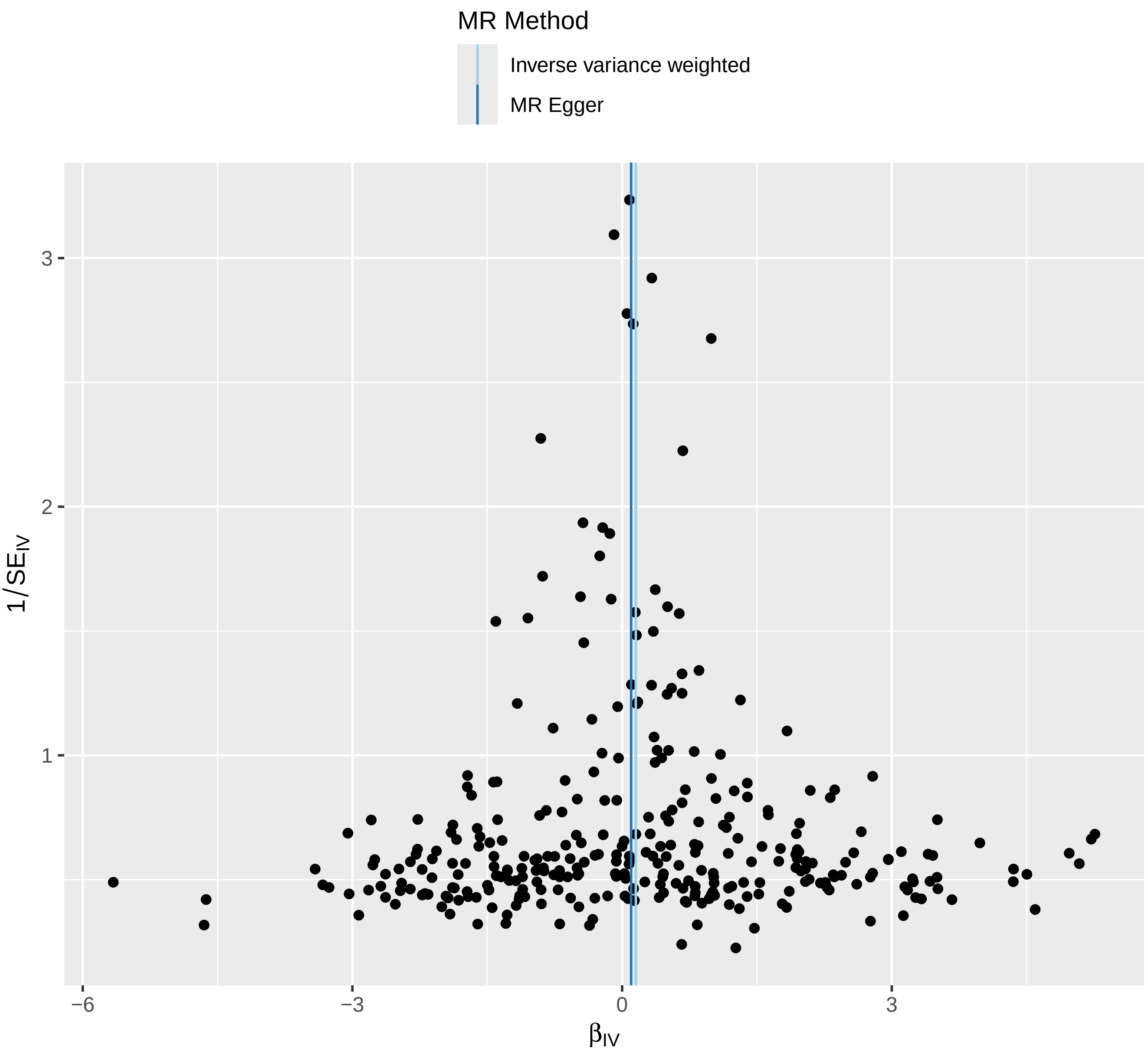
MR Method
Inverse variance weighted
MR Egger
1/SE$_{IV}$
3
2
1
−6
−3
0
3
β$_{IV}$

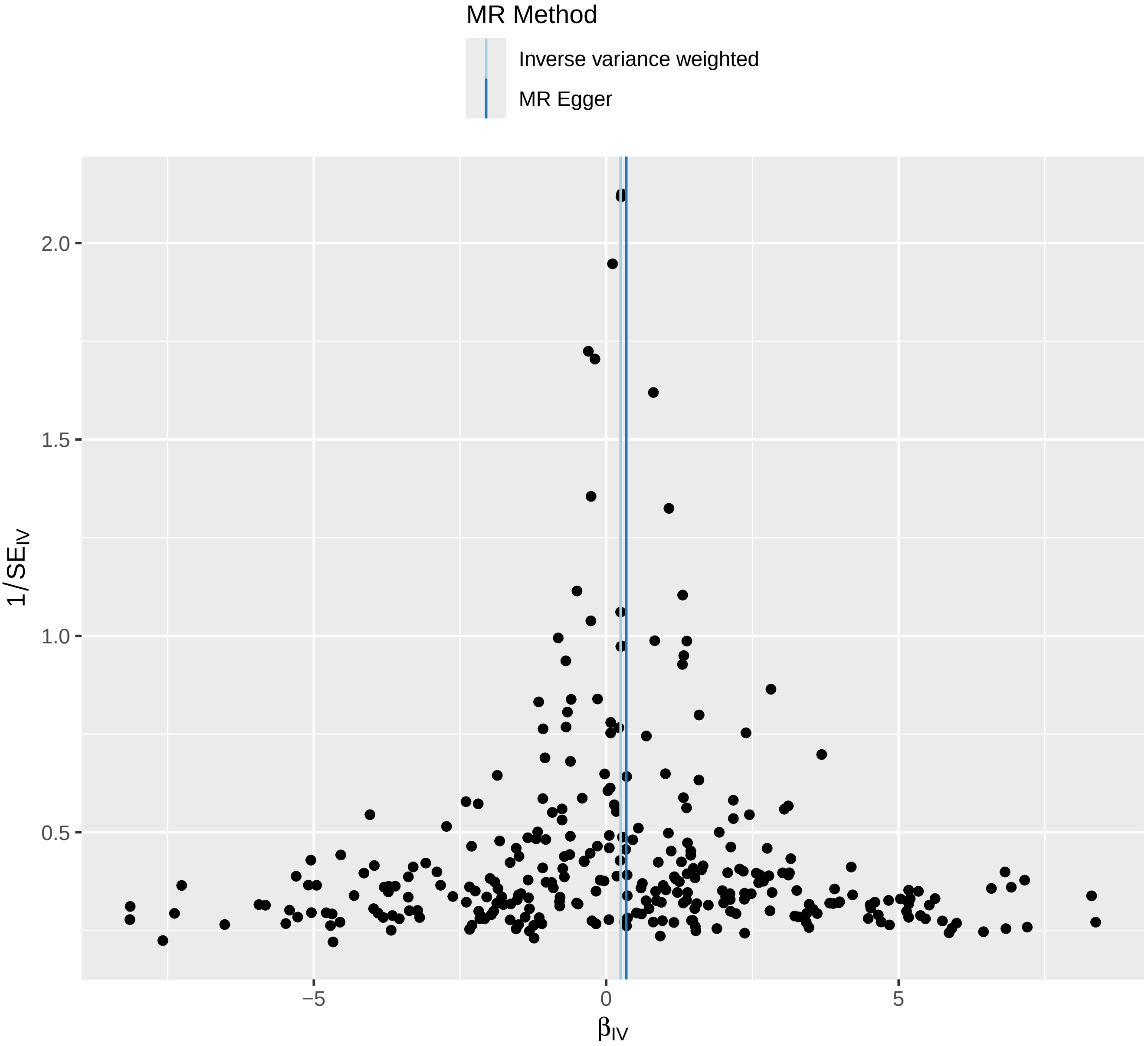

MR Method
Inverse variance weighted
MR Egger
2.0
1.5
1.0
0.5
$1/SE_{IV}$
−5
0
5
β_{IV}

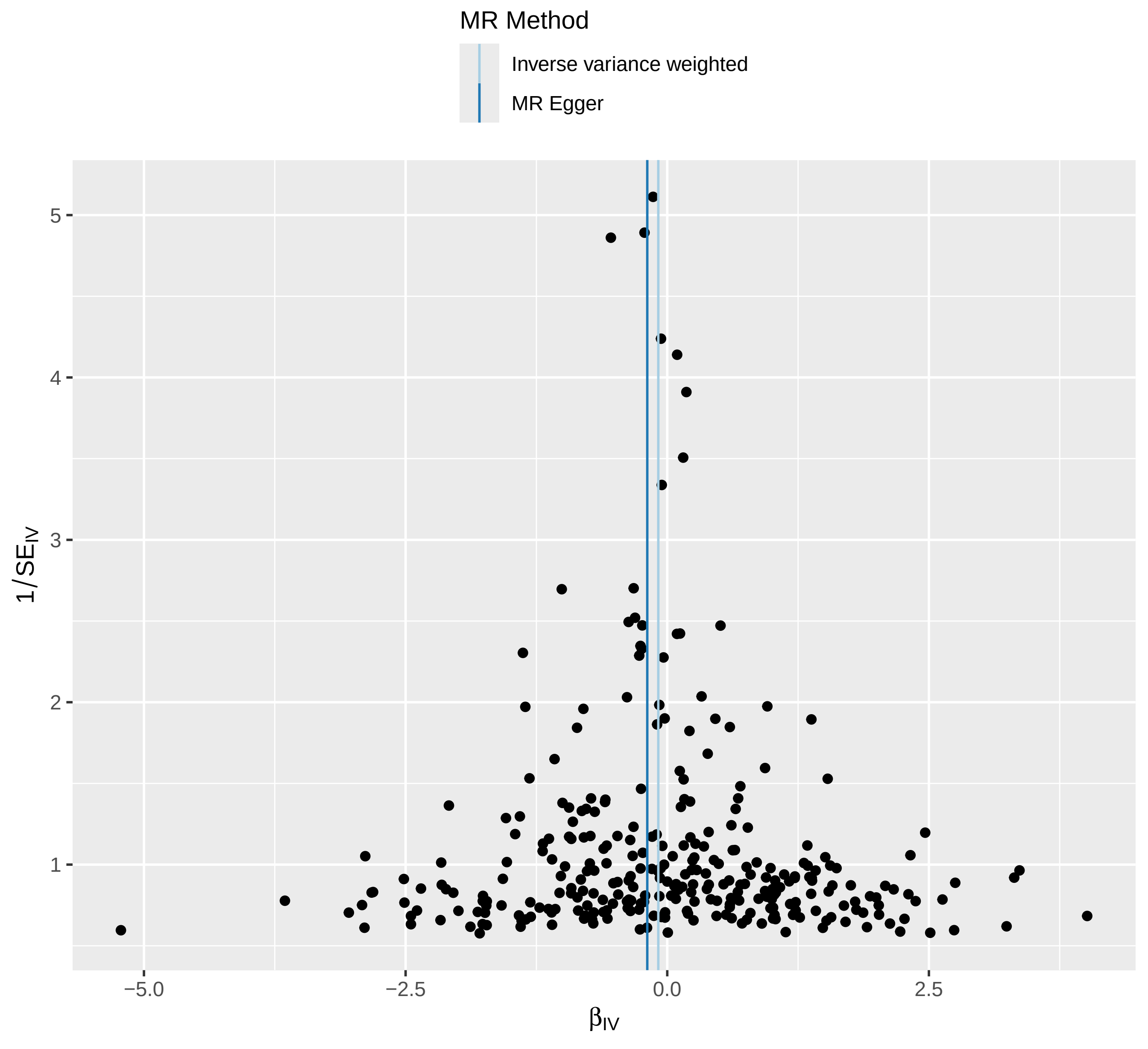
MR Method
Inverse variance weighted
MR Egger
1/SE_IV
β_IV
5
4
3
2
1
−5.0
−2.5
0.0
2.5

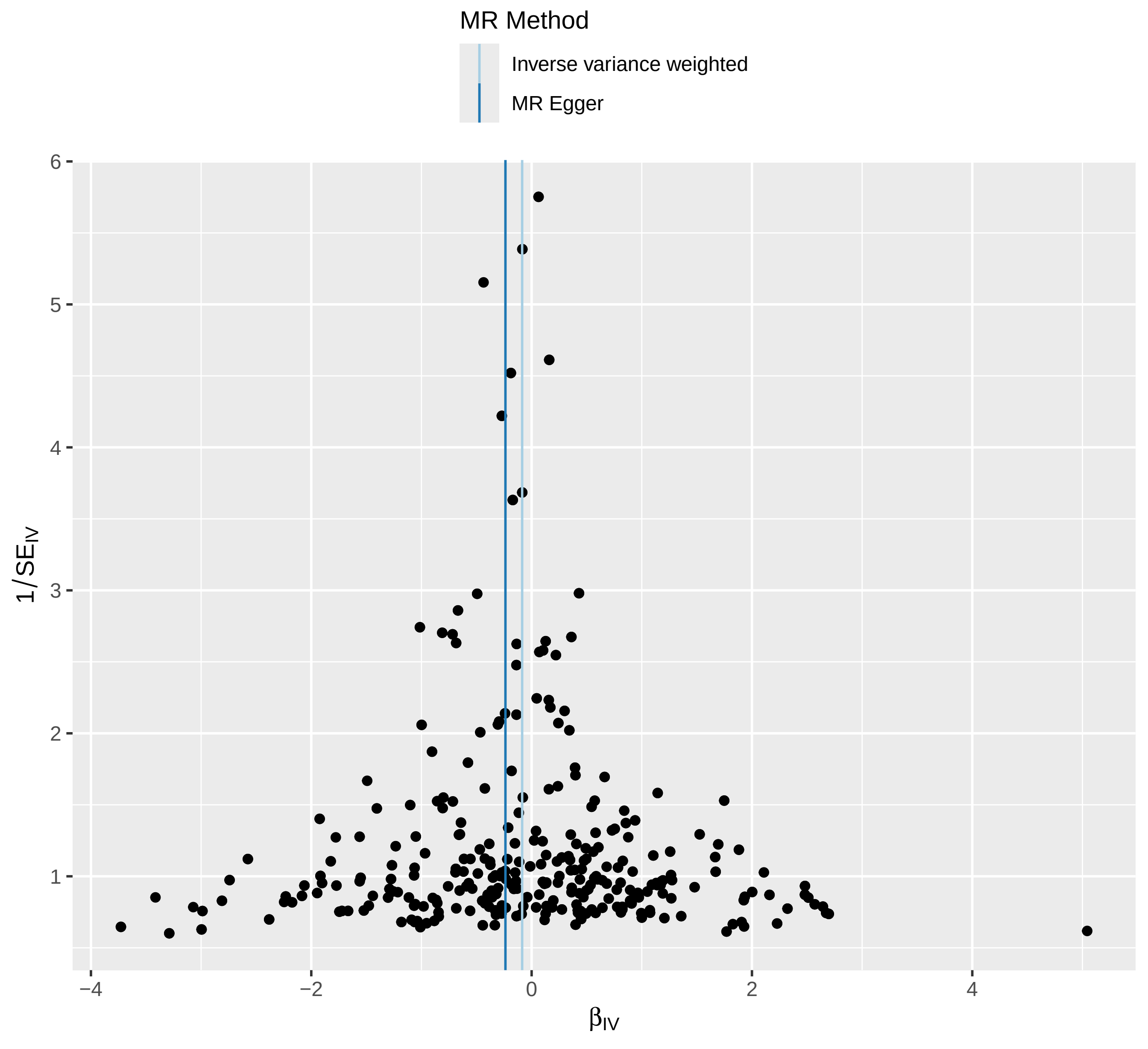
MR Method
Inverse variance weighted
MR Egger
6
5
4
3
2
1
$1/SE_{IV}$
−4
−2
0
2
4
β_{IV}

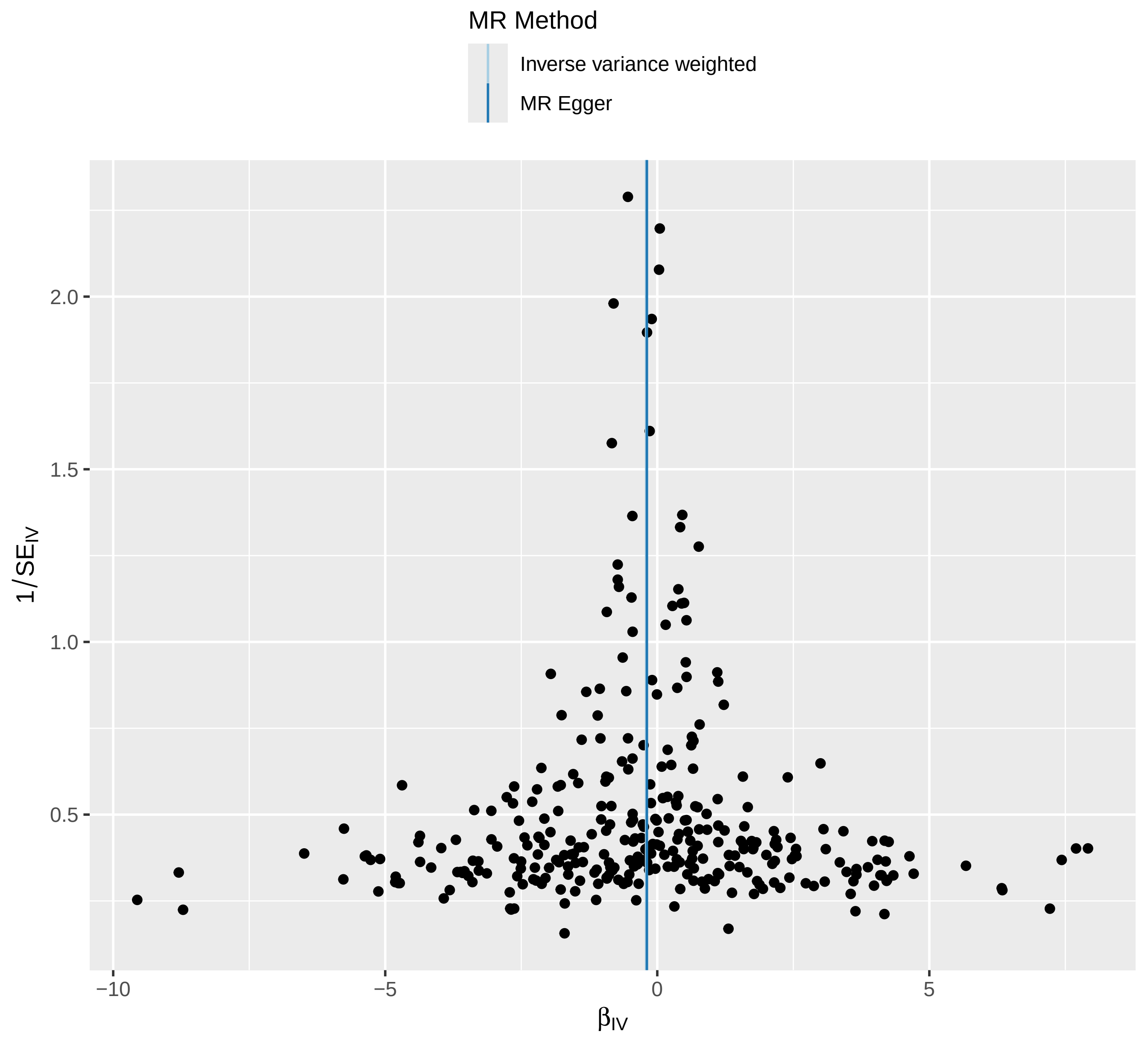
MR Method
Inverse variance weighted
MR Egger
1/SE$_{IV}$
2.0
1.5
1.0
0.5
−10
−5
0
5
β$_{IV}$

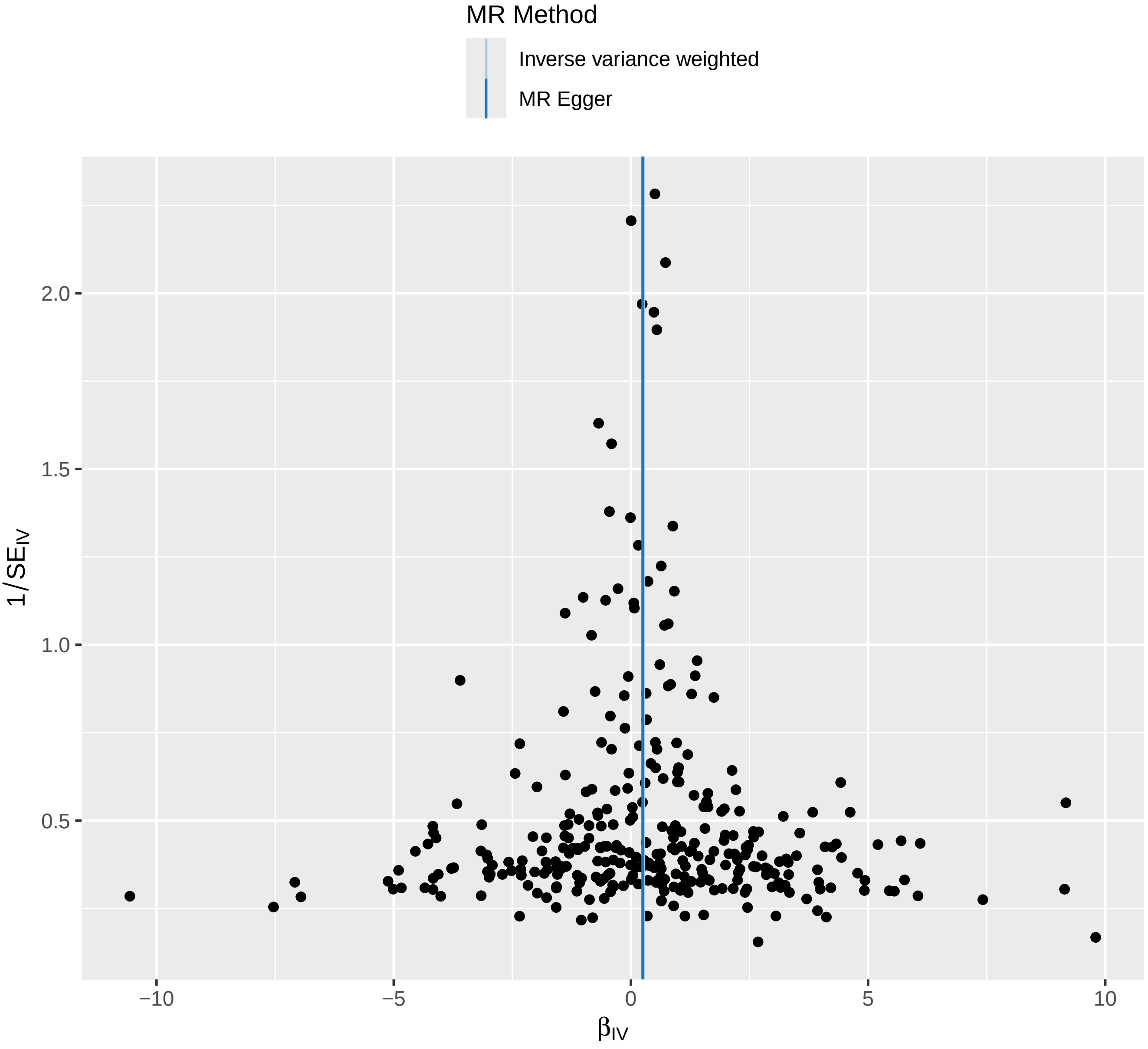

MR Method
Inverse variance weighted
MR Egger
1/SE_IV
2.0
1.5
1.0
0.5
−10
−5
0
5
10
β_IV

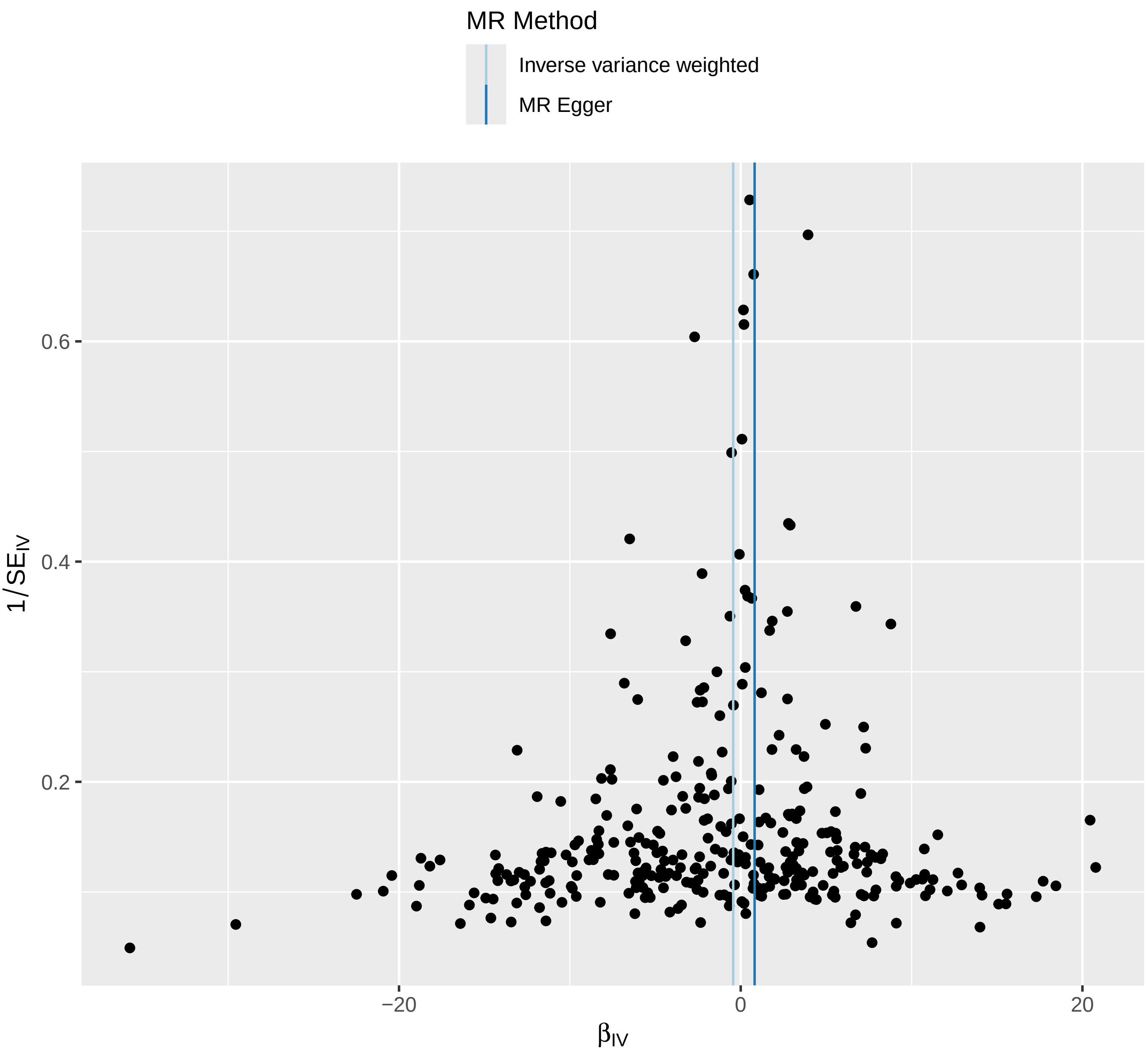
MR Method
Inverse variance weighted
MR Egger
1/SE_IV
0.6
0.4
0.2
−20
0
20
β_IV

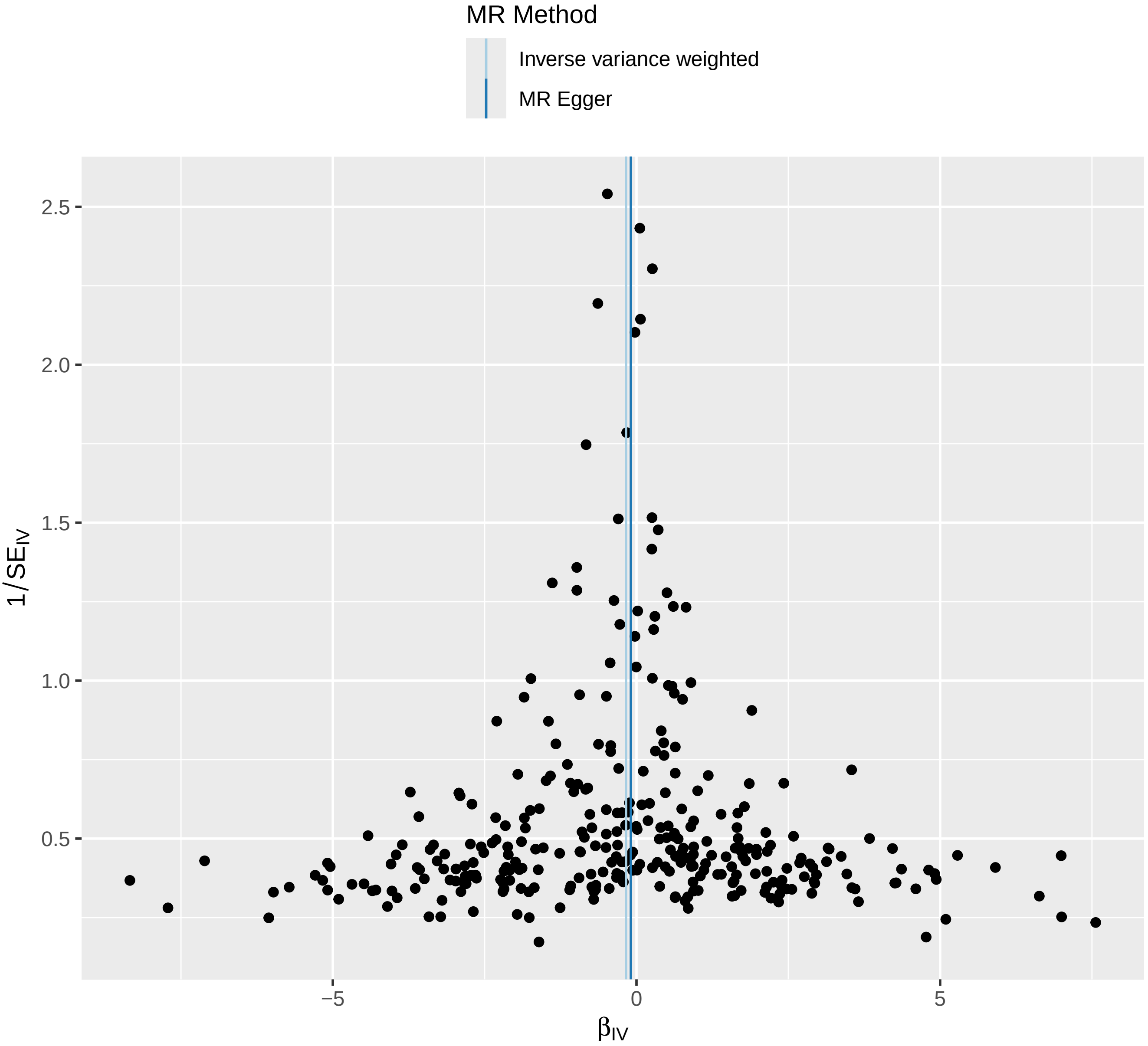
MR Method
Inverse variance weighted
MR Egger
1/SE$_{IV}$
2.5
2.0
1.5
1.0
0.5
−5
0
5
β$_{IV}$

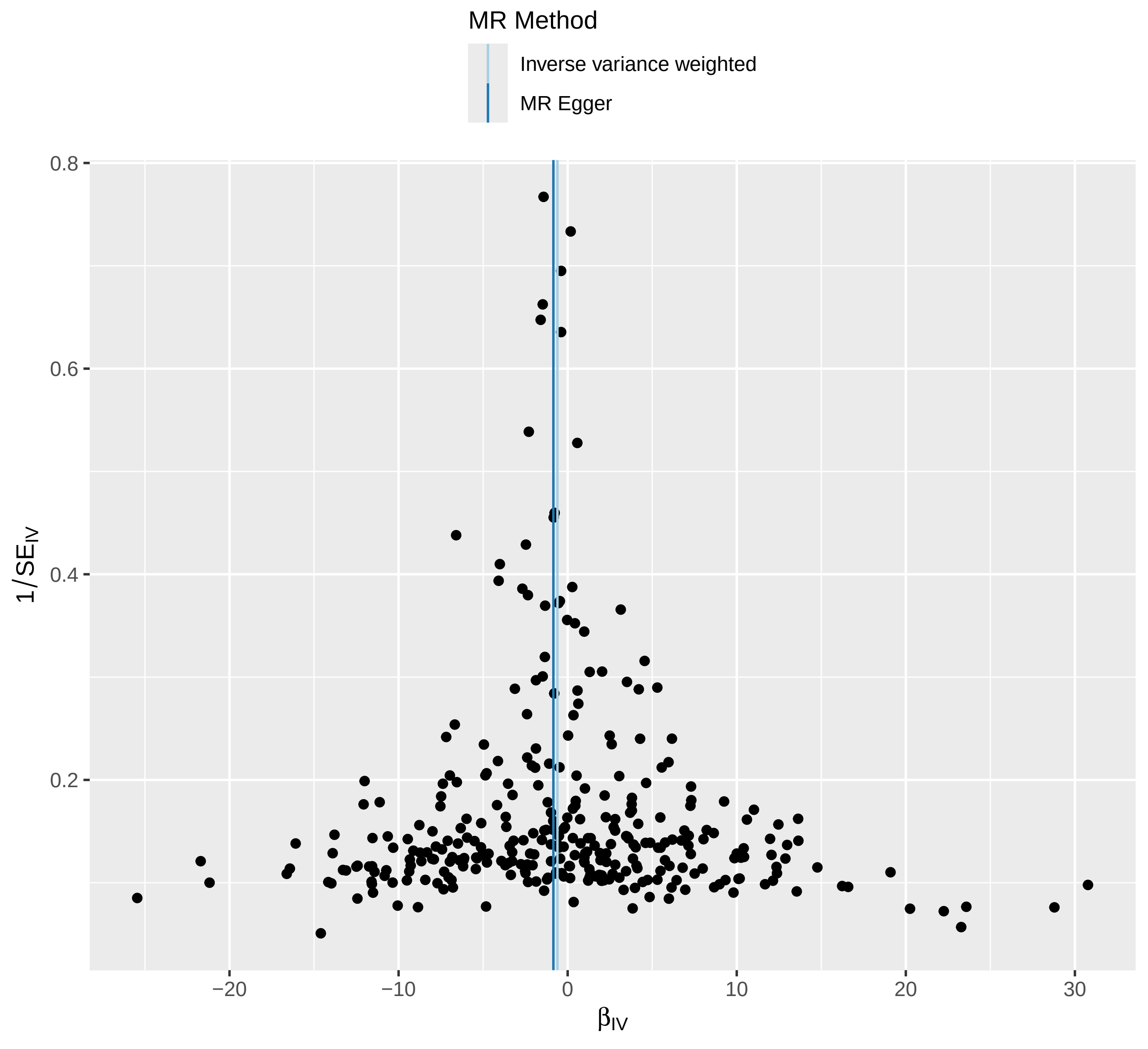
MR Method
Inverse variance weighted
MR Egger
1/SE_IV
0.8
0.6
0.4
0.2
−20
−10
0
10
20
30
β_IV

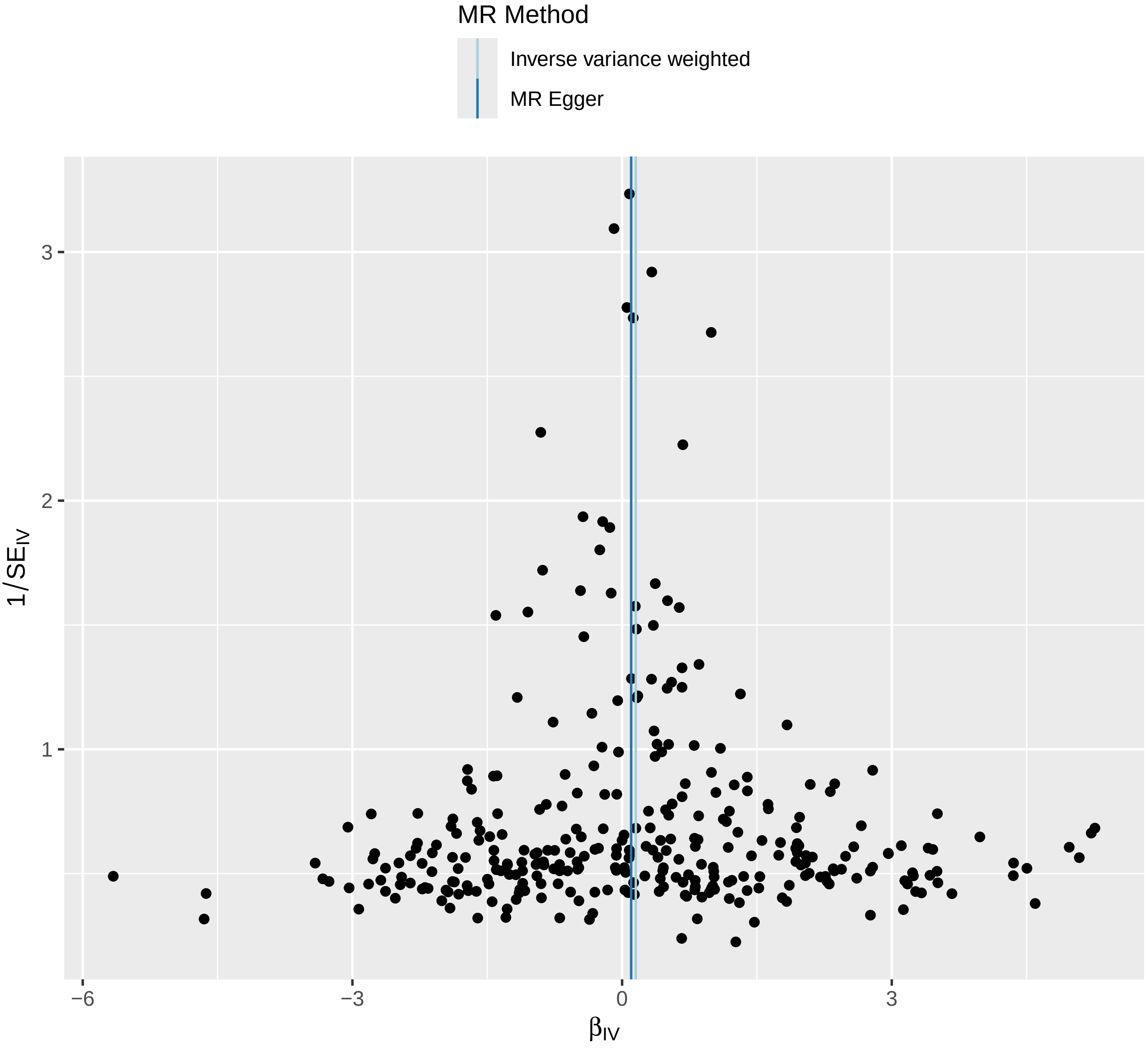

MR Method
Inverse variance weighted
MR Egger
3
2
1
1/SE_IV
−6
−3
0
3
β_IV

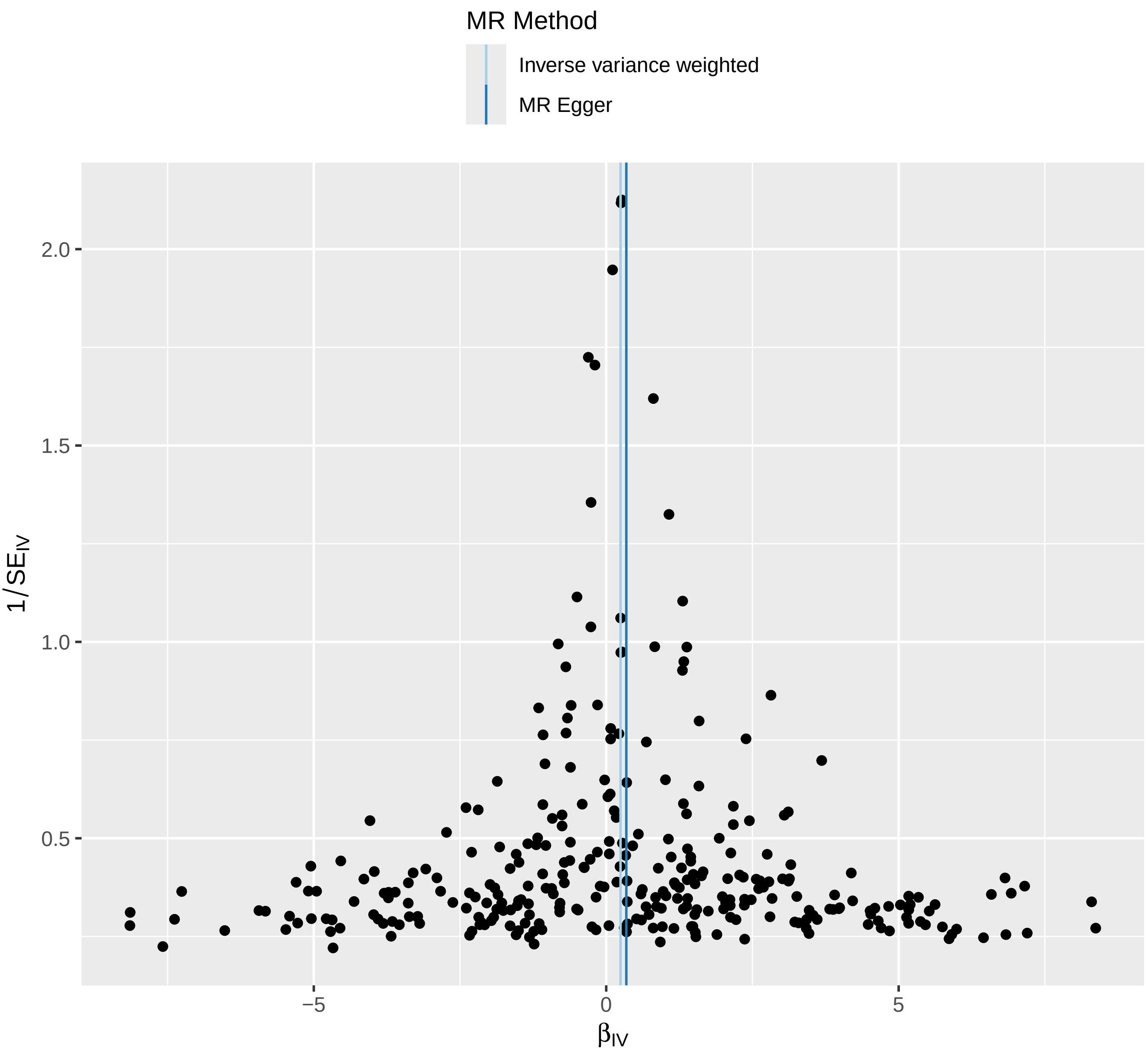
MR Method
Inverse variance weighted
MR Egger
2.0
1.5
1.0
0.5
−5
0
5
β_{IV}
$1/SE_{IV}$

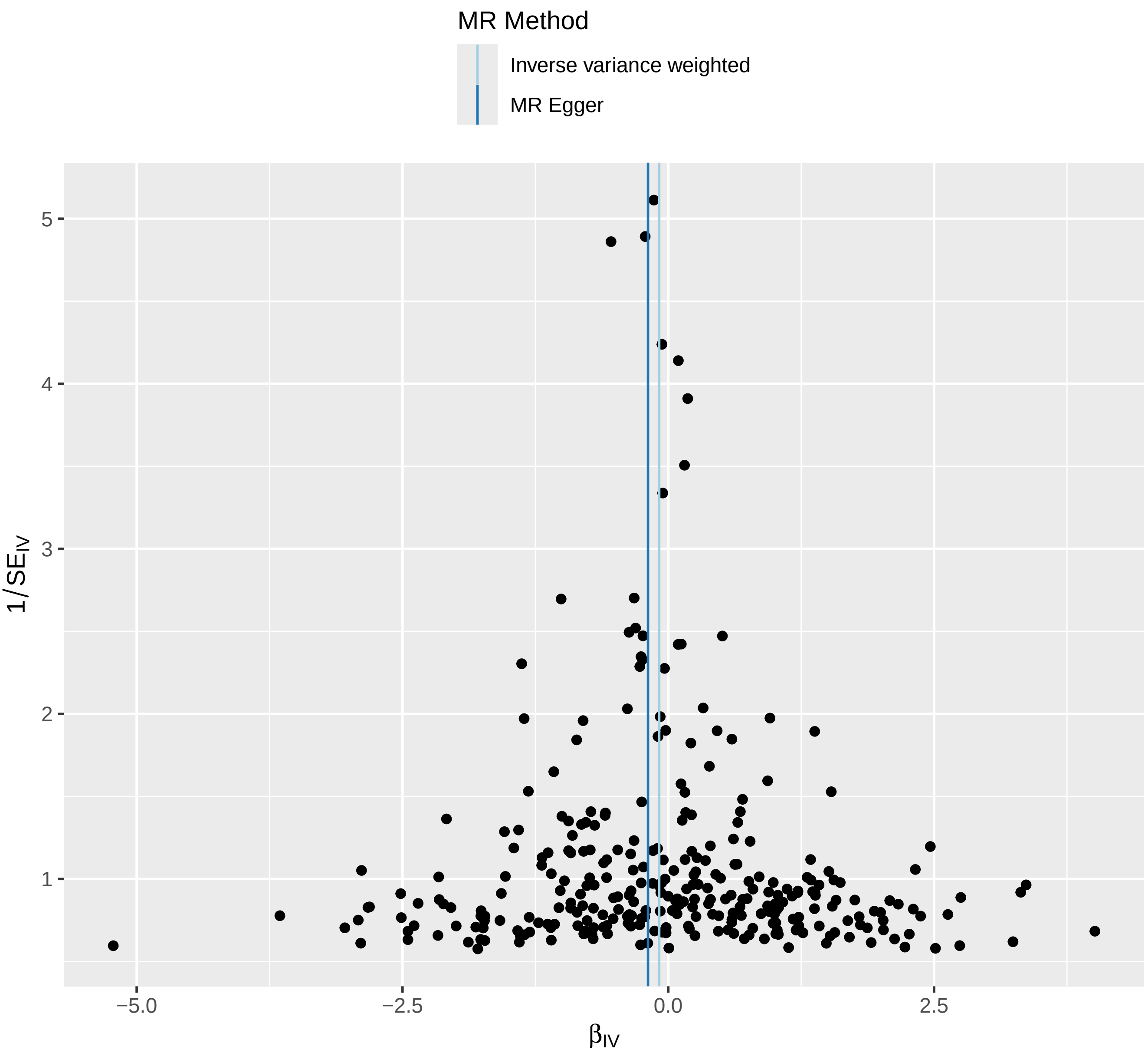

MR Method
Inverse variance weighted
MR Egger
5
4
3
2
1
$1/SE_{IV}$
−5.0
−2.5
0.0
2.5
β_{IV}

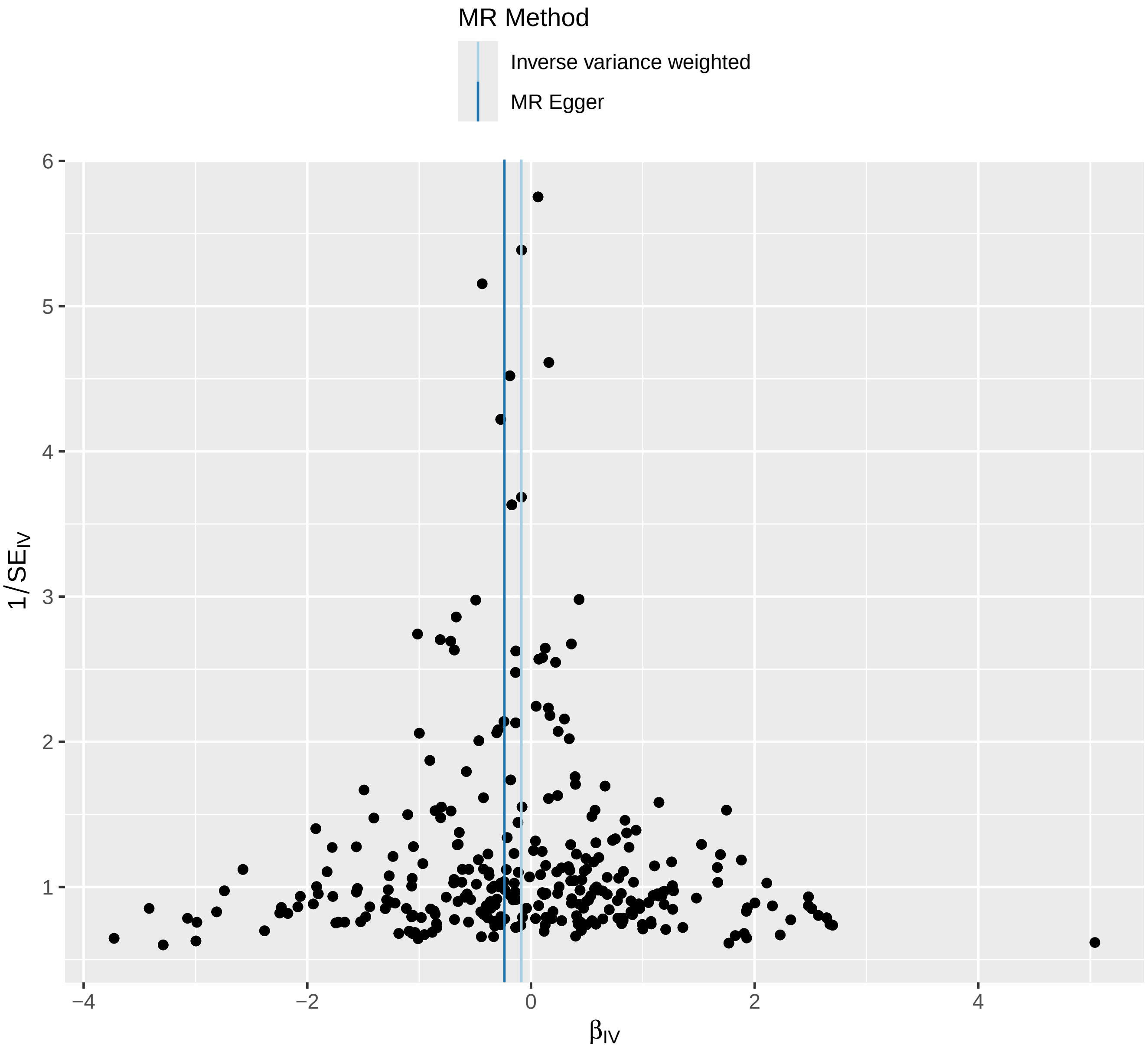
MR Method
Inverse variance weighted
MR Egger
6
5
4
3
2
1
1/SE$_{IV}$
−4
−2
0
2
4
β$_{IV}$

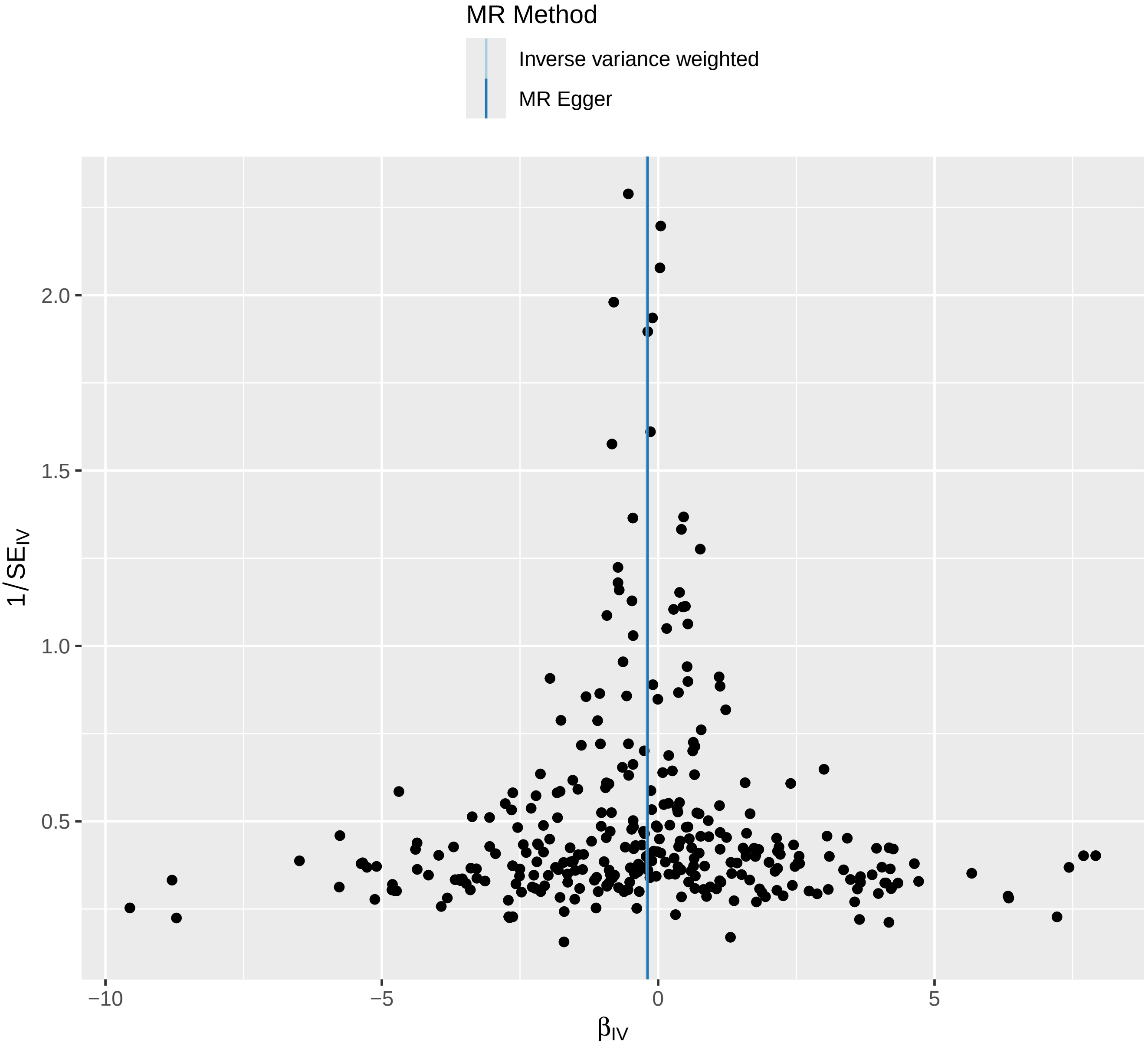
MR Method
Inverse variance weighted
MR Egger
1/SE_IV
2.0
1.5
1.0
0.5
−10
−5
0
5
β_IV

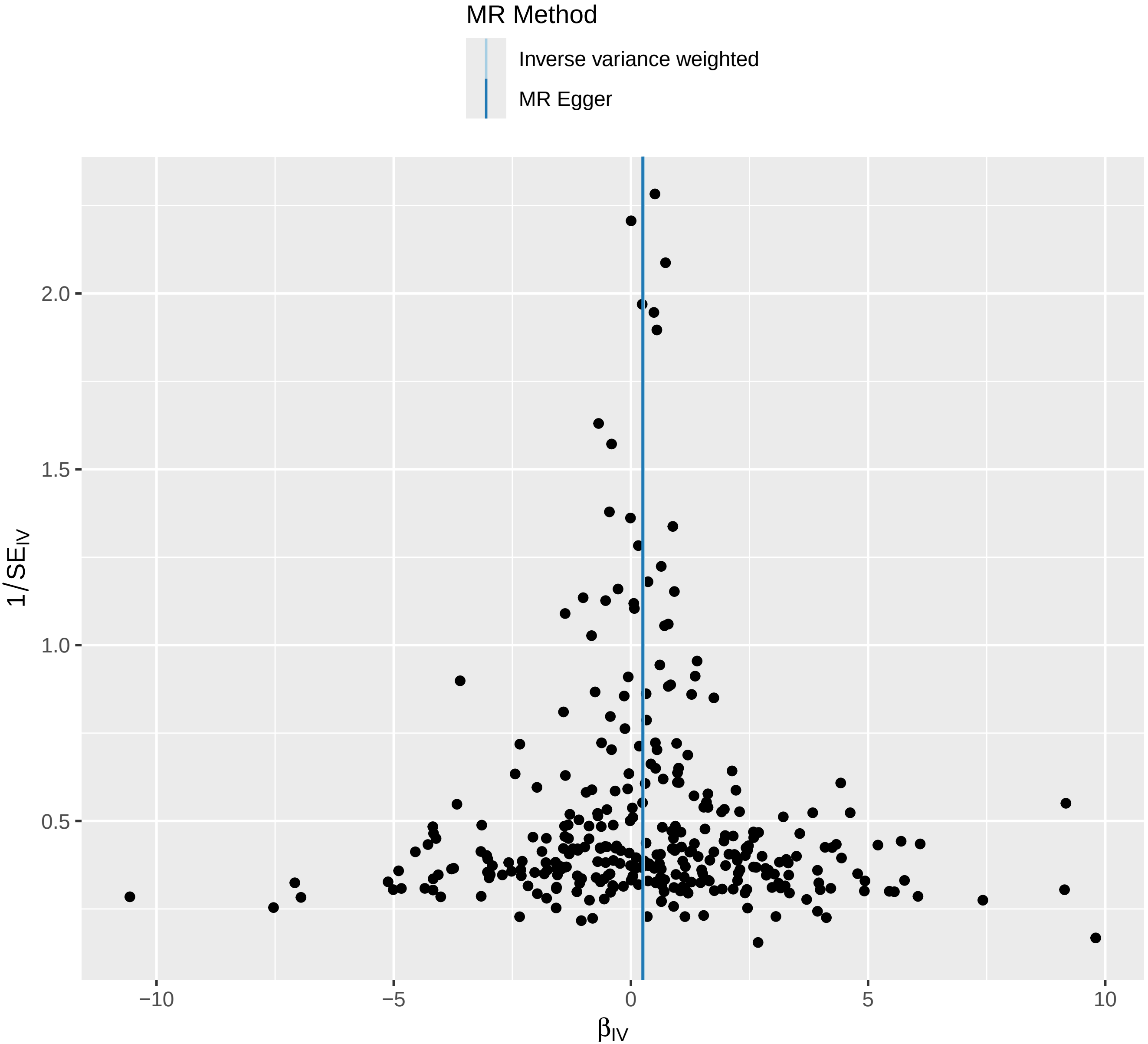
MR Method
Inverse variance weighted
MR Egger
1/SE_IV
2.0
1.5
1.0
0.5
−10
−5
0
5
10
β_IV

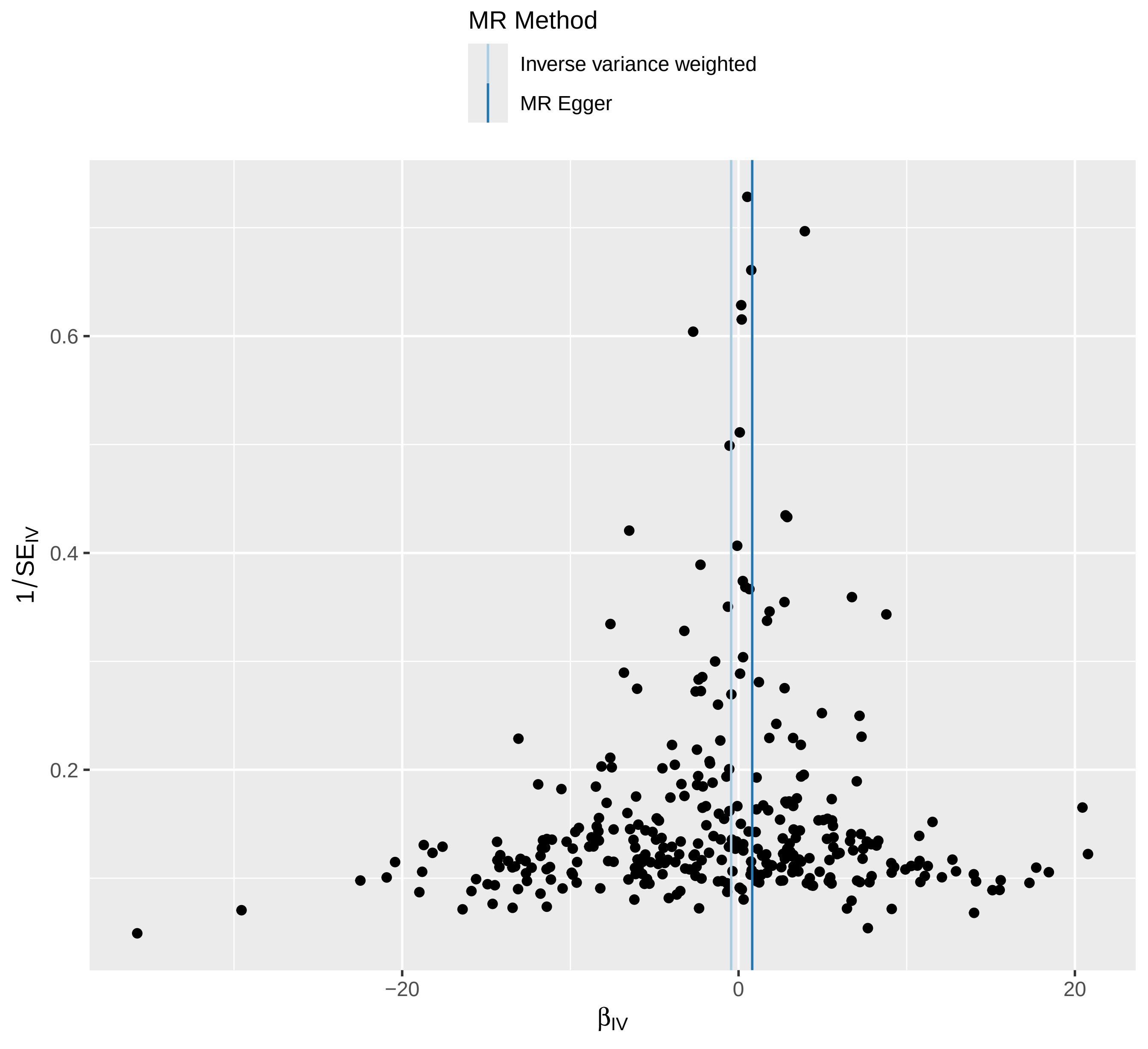

MR Method
Inverse variance weighted
MR Egger
0.6
0.4
0.2
$1/SE_{IV}$
−20
0
20
β_{IV}

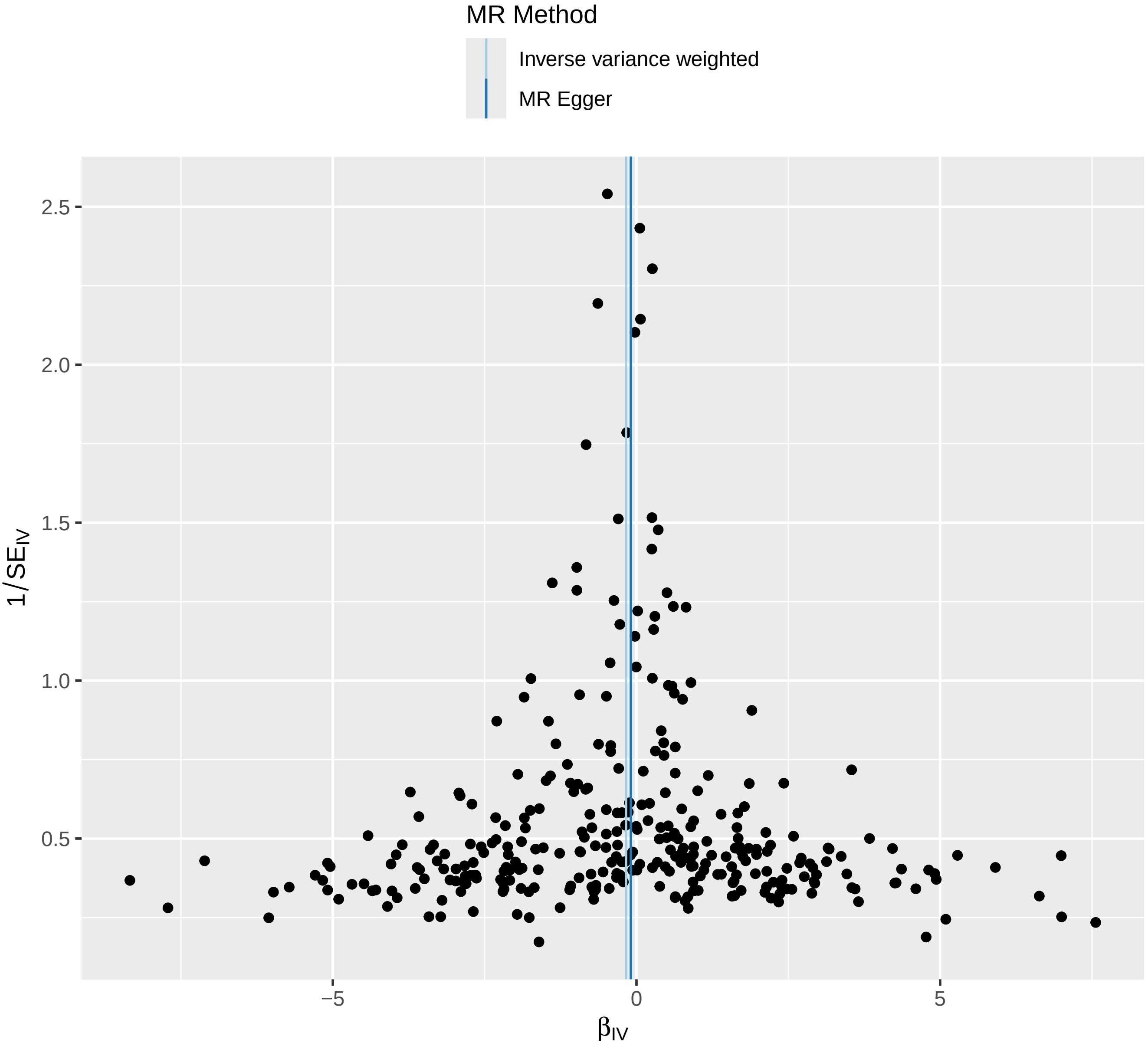

MR Method
Inverse variance weighted
MR Egger
2.5
2.0
1.5
1.0
0.5
$1/SE_{IV}$
−5
0
5
β_{IV}

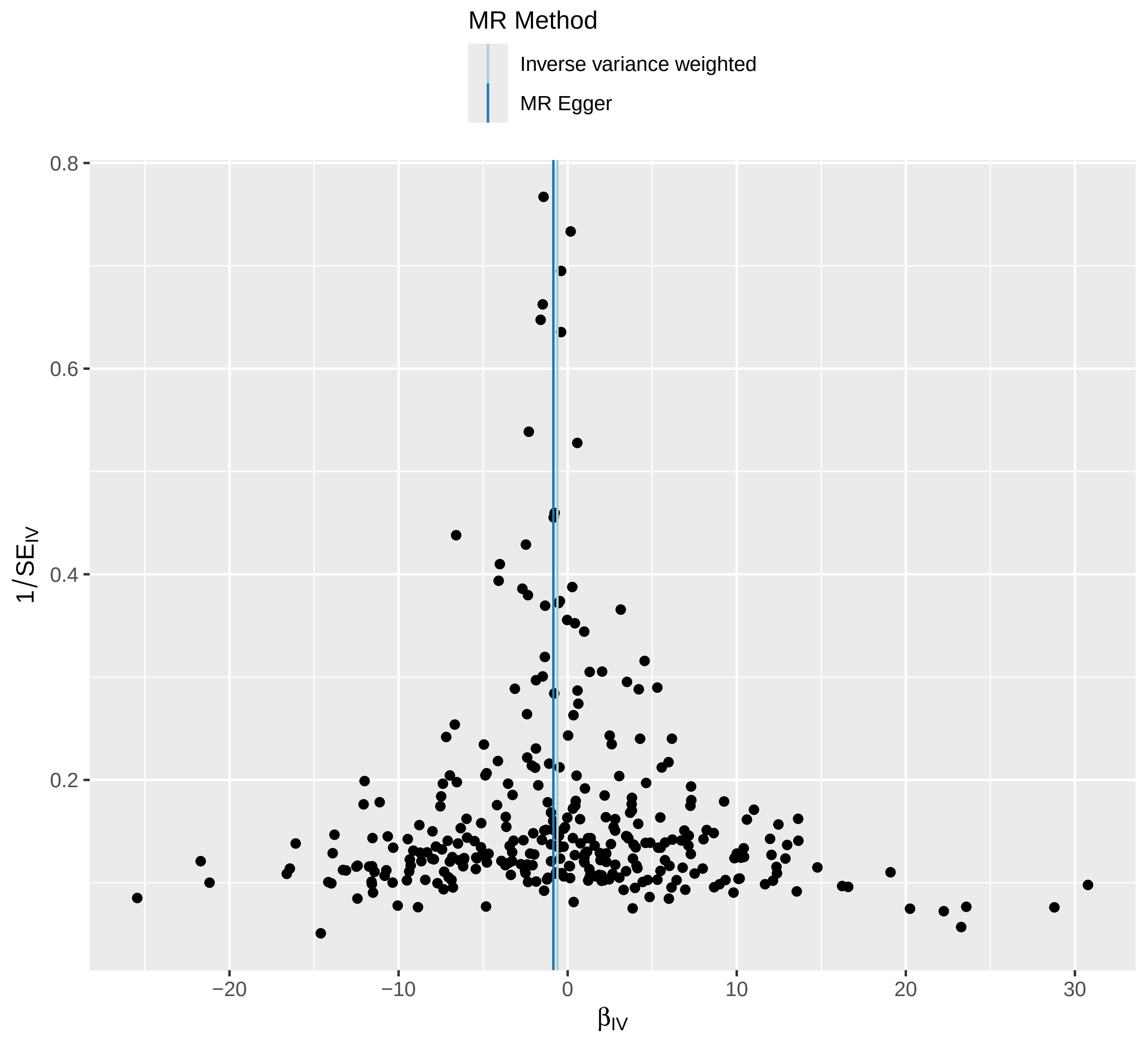
MR Method
Inverse variance weighted
MR Egger
0.8
0.6
0.4
0.2
1/SE_IV
−20
−10
0
10
20
30
β_IV

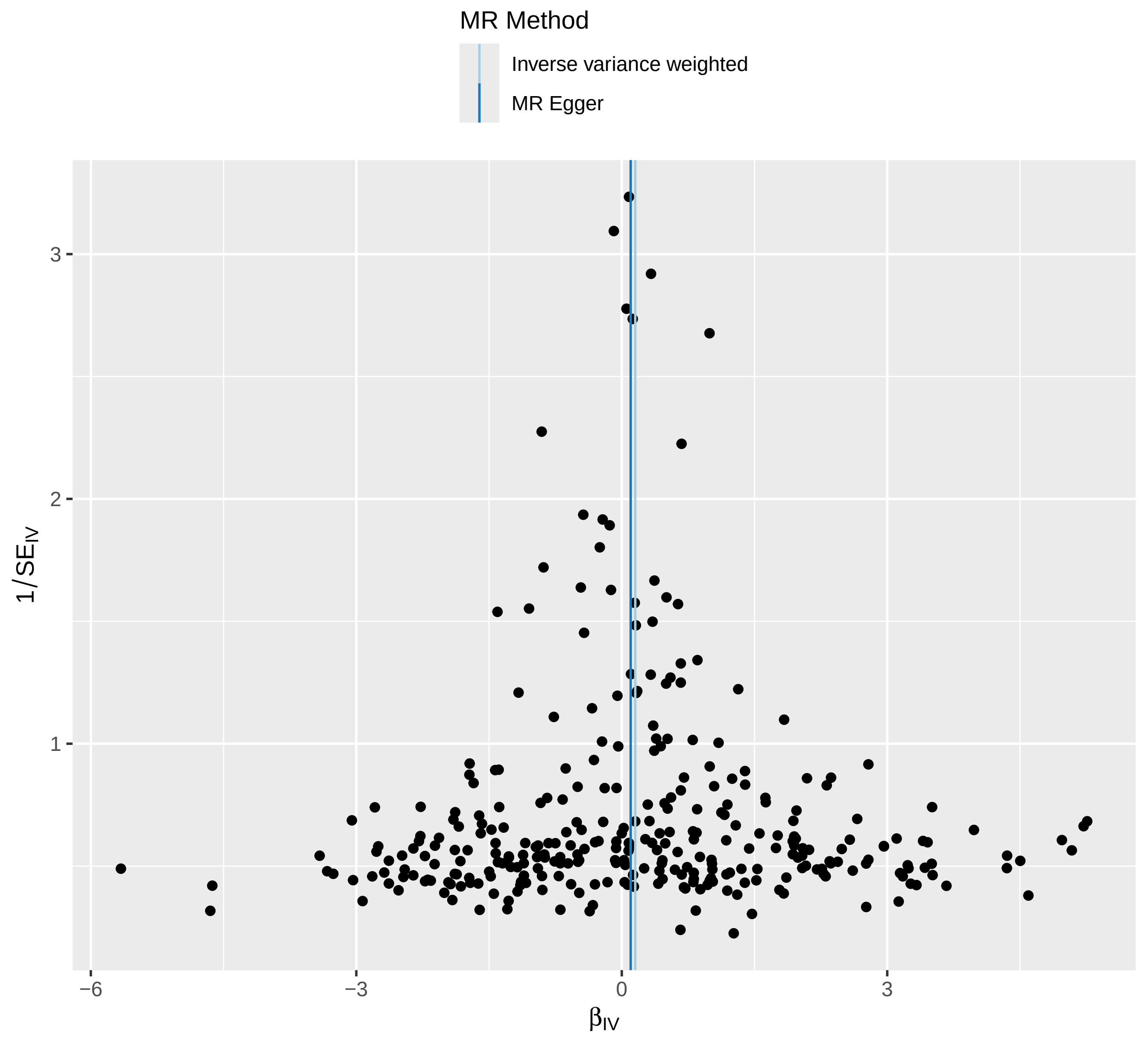
MR Method
Inverse variance weighted
MR Egger
3
2
1
1/SE_IV
−6
−3
0
3
β_IV

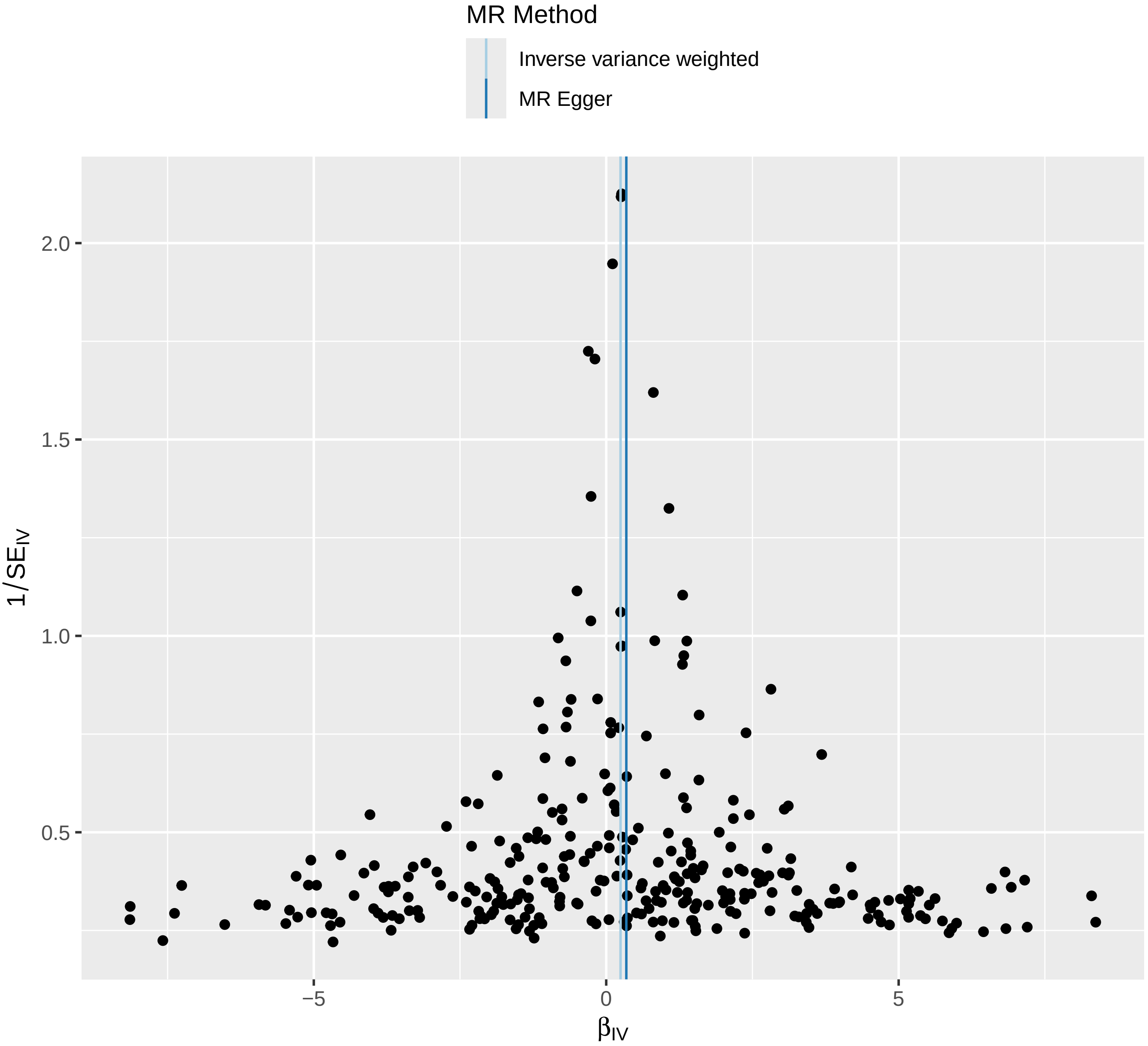
MR Method
Inverse variance weighted
MR Egger
2.0
1.5
1.0
0.5
1/SE_IV
−5
0
5
β_IV

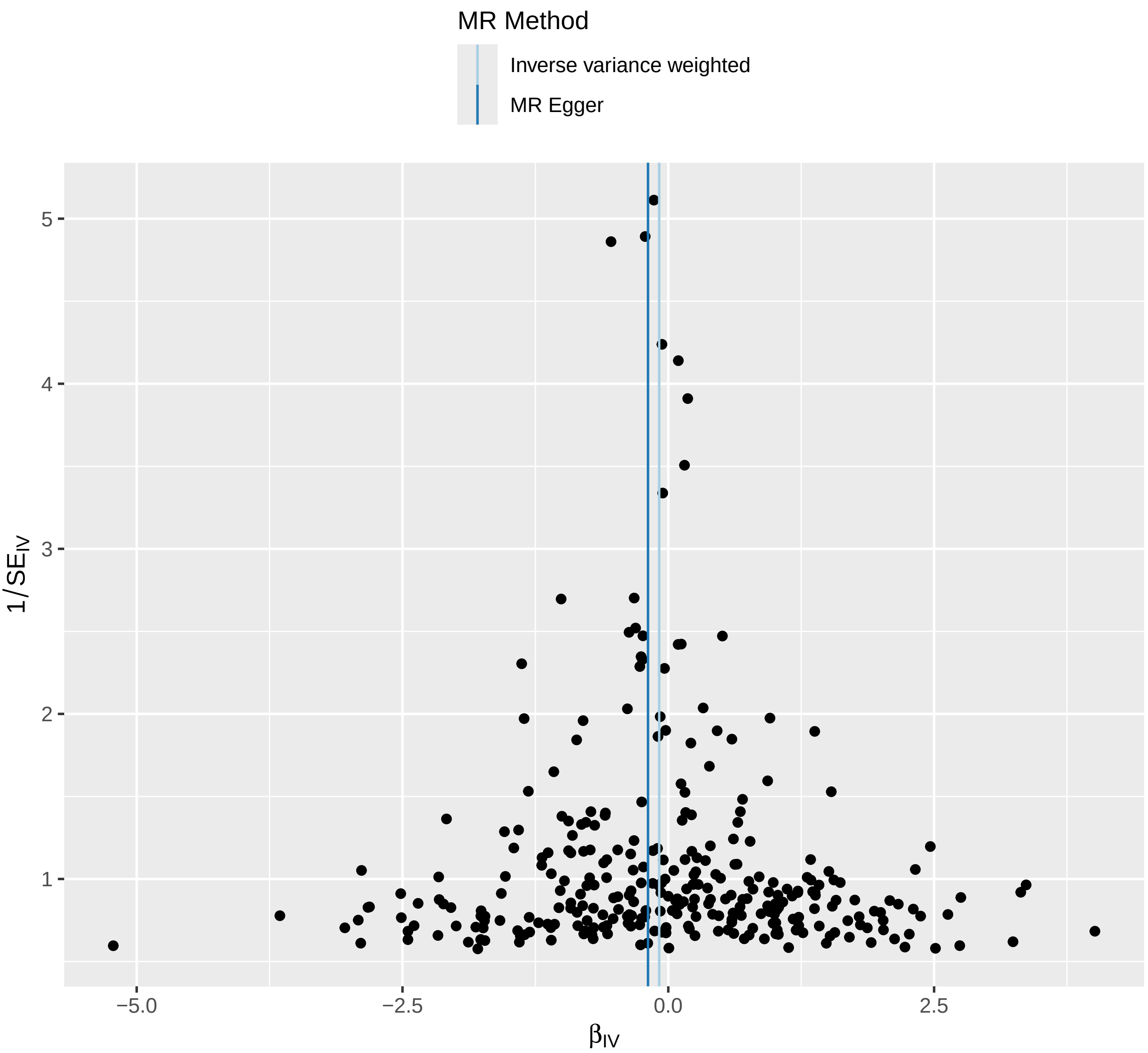
MR Method
Inverse variance weighted
MR Egger
5
4
3
2
1
1/SE_IV
−5.0
−2.5
0.0
2.5
β_IV

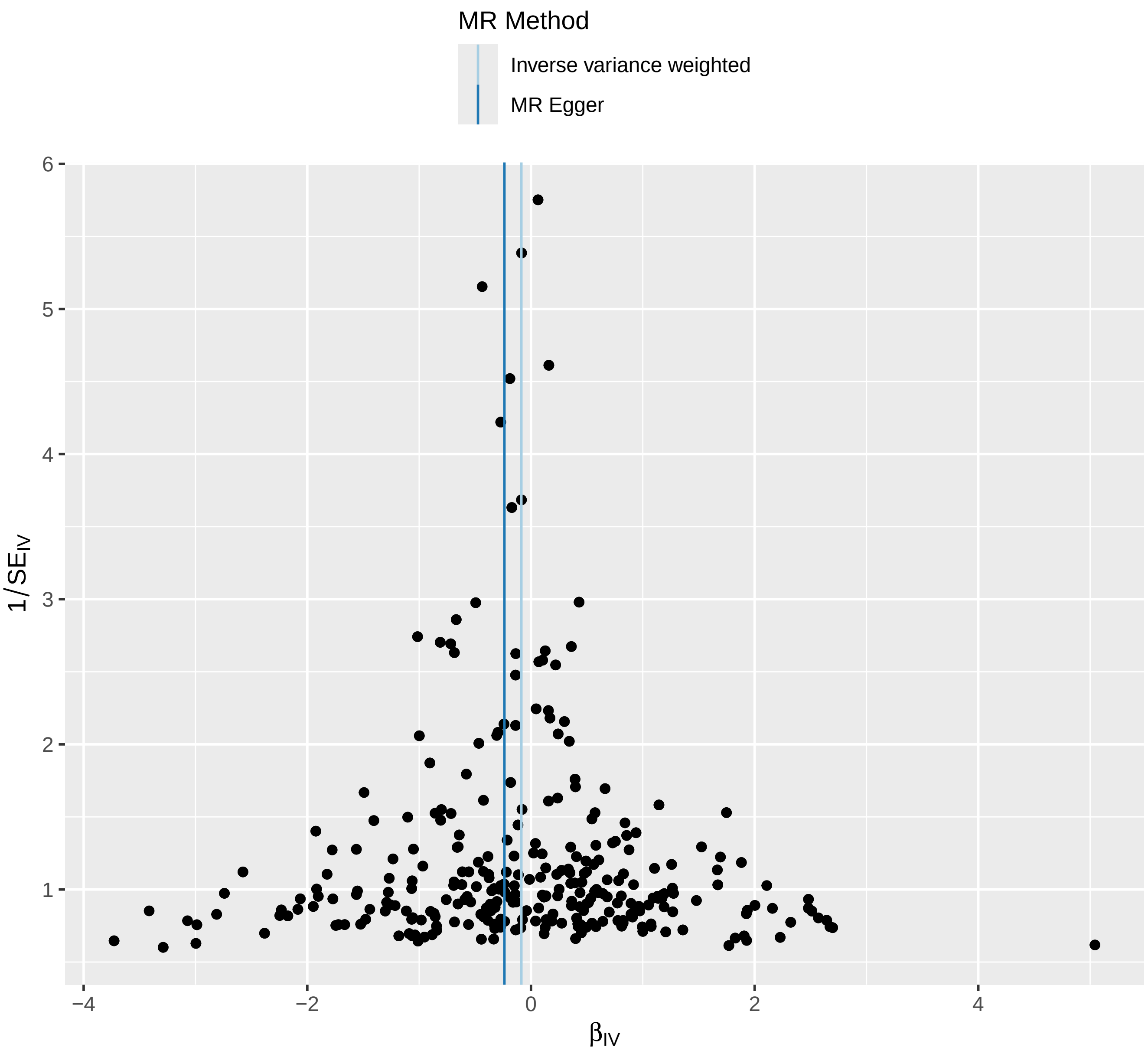

MR Method
Inverse variance weighted
MR Egger
6
5
4
3
2
1
1/SE$_{IV}$
−4
−2
0
2
4
β$_{IV}$

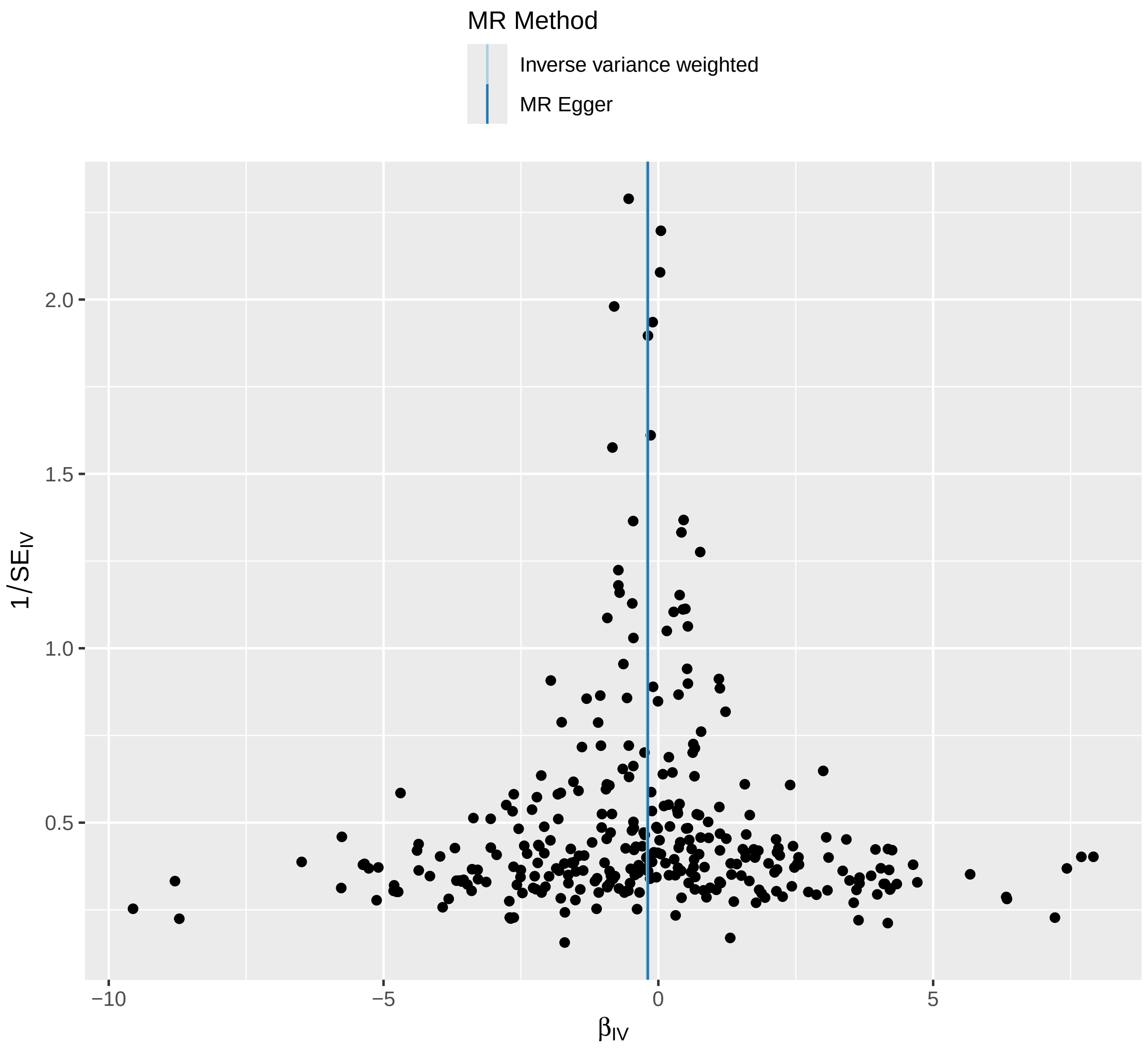

MR Method
Inverse variance weighted
MR Egger
2.0
1.5
1.0
0.5
1/SE_IV
−10
−5
0
5
β_IV

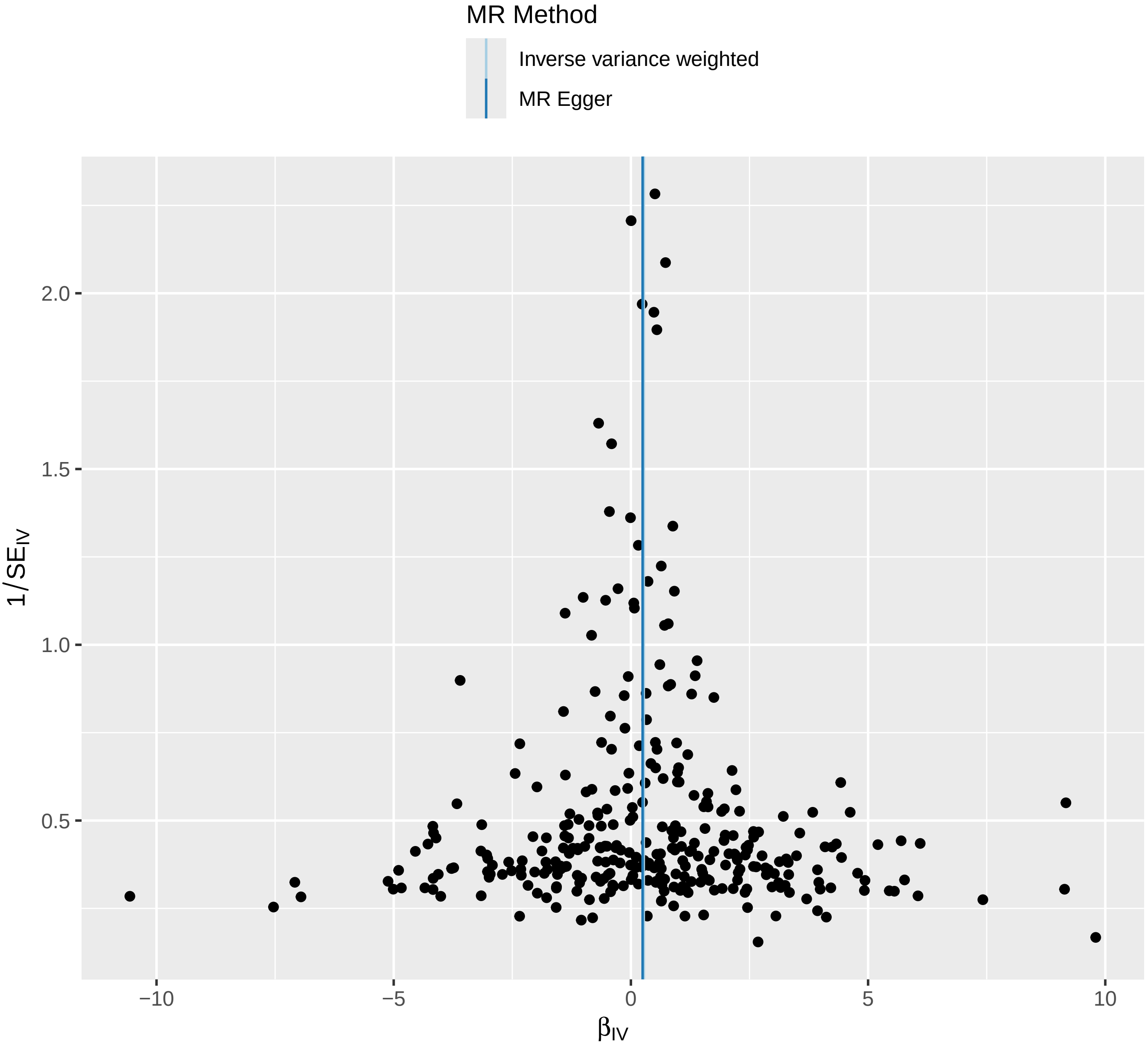
MR Method
Inverse variance weighted
MR Egger
2.0
1.5
1.0
0.5
1/SE_IV
−10
−5
0
5
10
β_IV

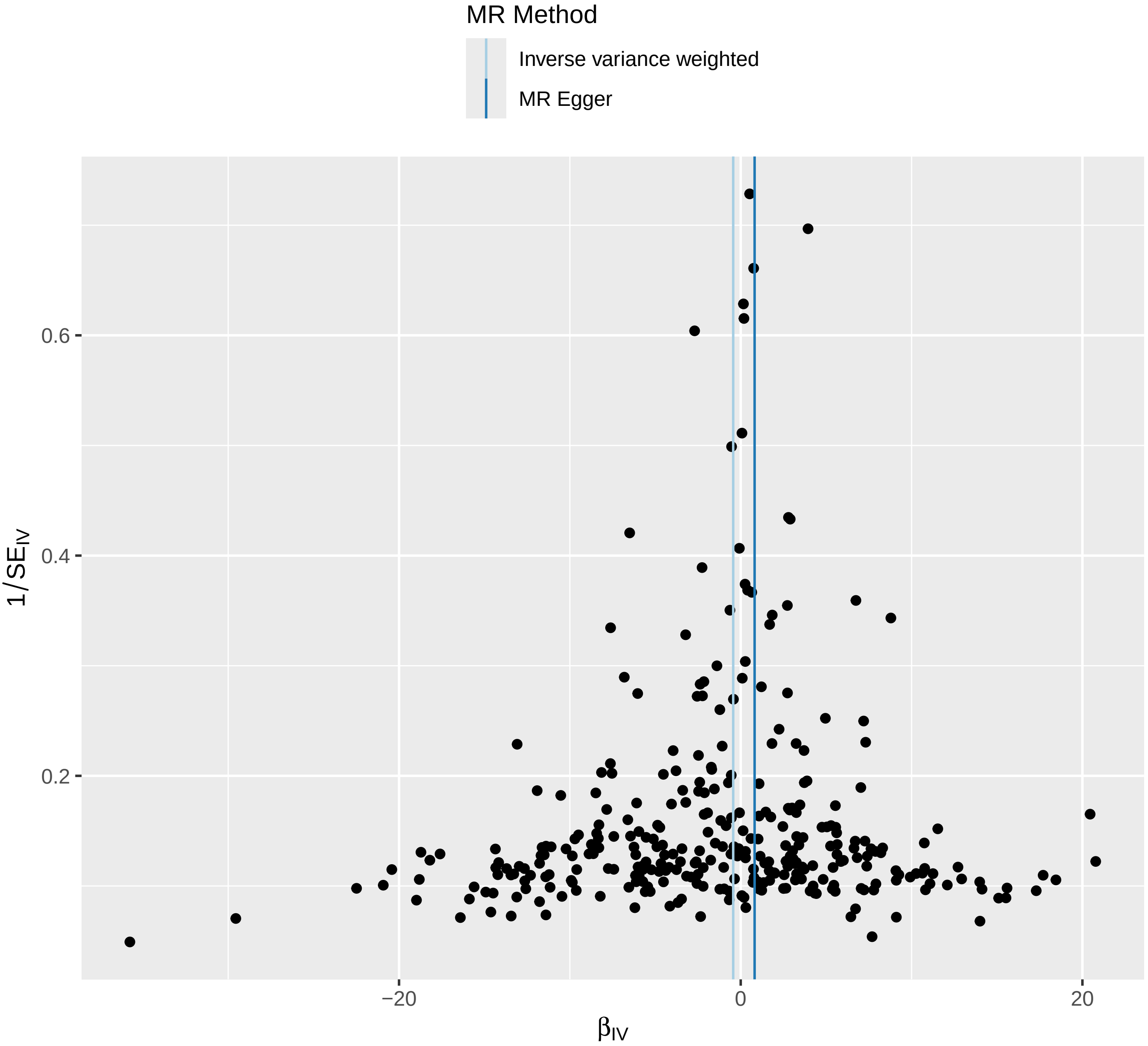
MR Method
Inverse variance weighted
MR Egger
1/SE$_{IV}$
0.6
0.4
0.2
−20
0
20
β$_{IV}$

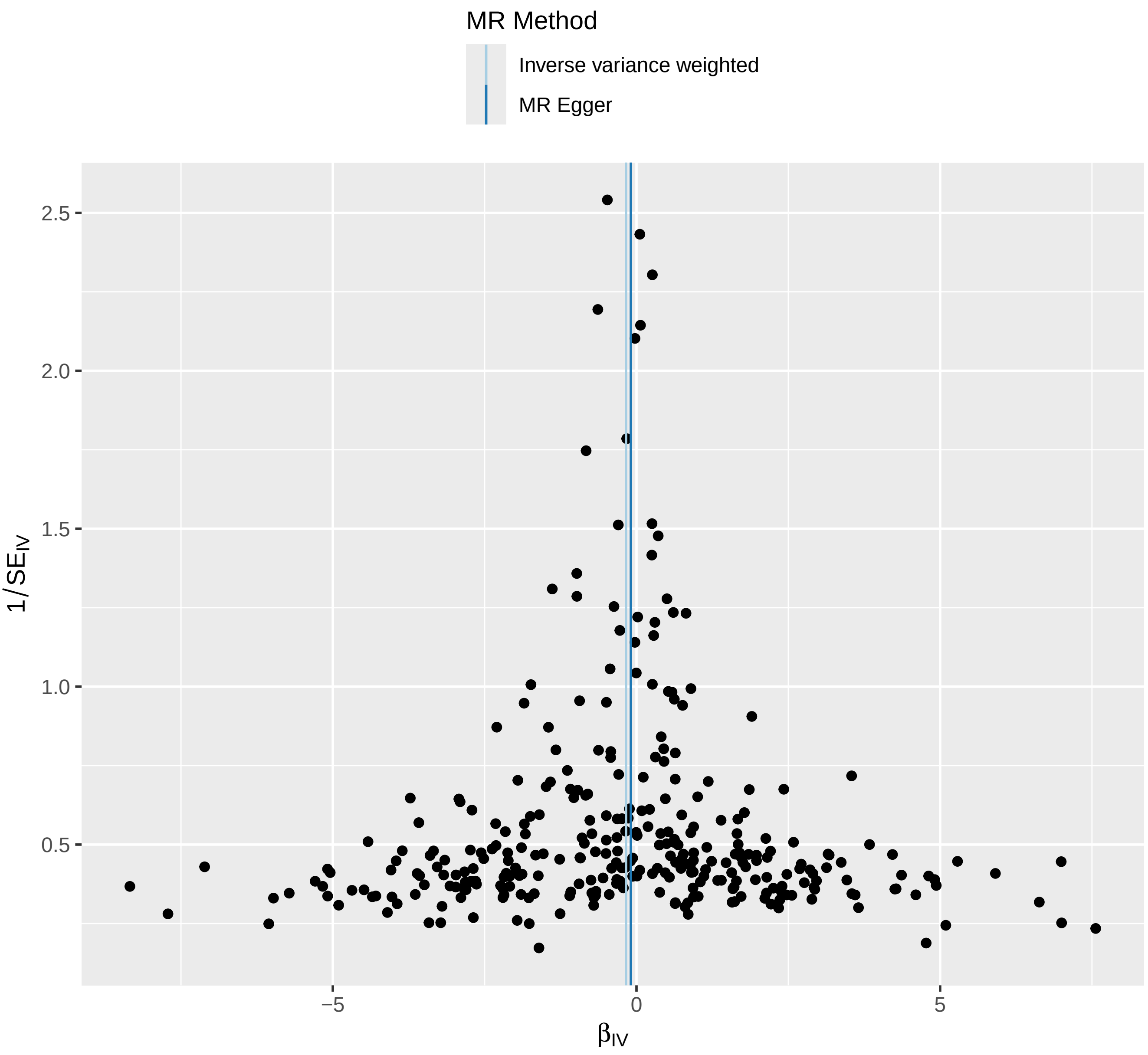
MR Method
Inverse variance weighted
MR Egger
2.5
2.0
1.5
1.0
0.5
$1/SE_{IV}$
−5
0
5
β_{IV}

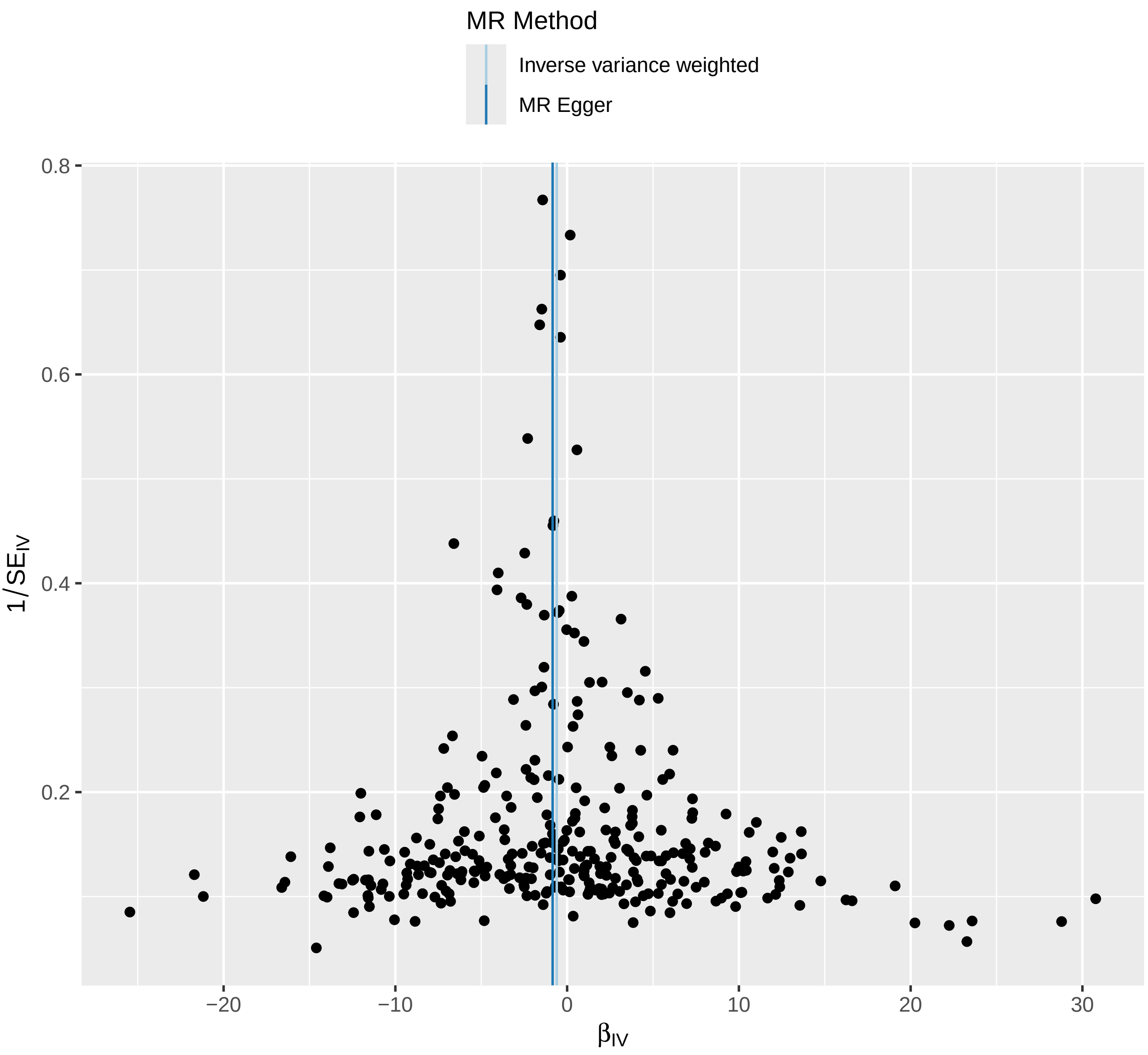
MR Method
Inverse variance weighted
MR Egger
0.8
0.6
0.4
0.2
1/SE_IV
−20
−10
0
10
20
30
β_IV

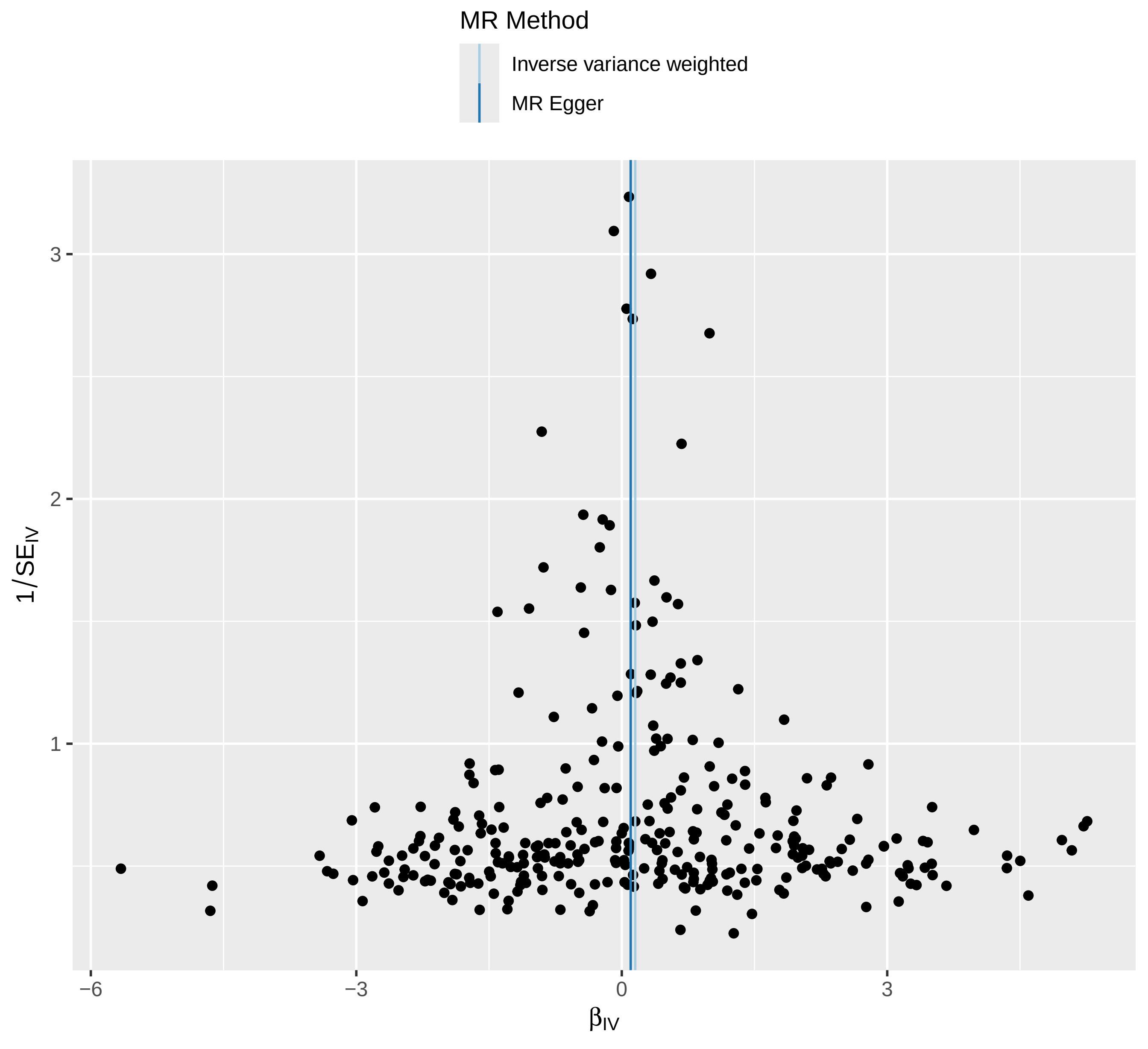
MR Method
Inverse variance weighted
MR Egger
1/SE_IV
3
2
1
−6
−3
0
3
β_IV

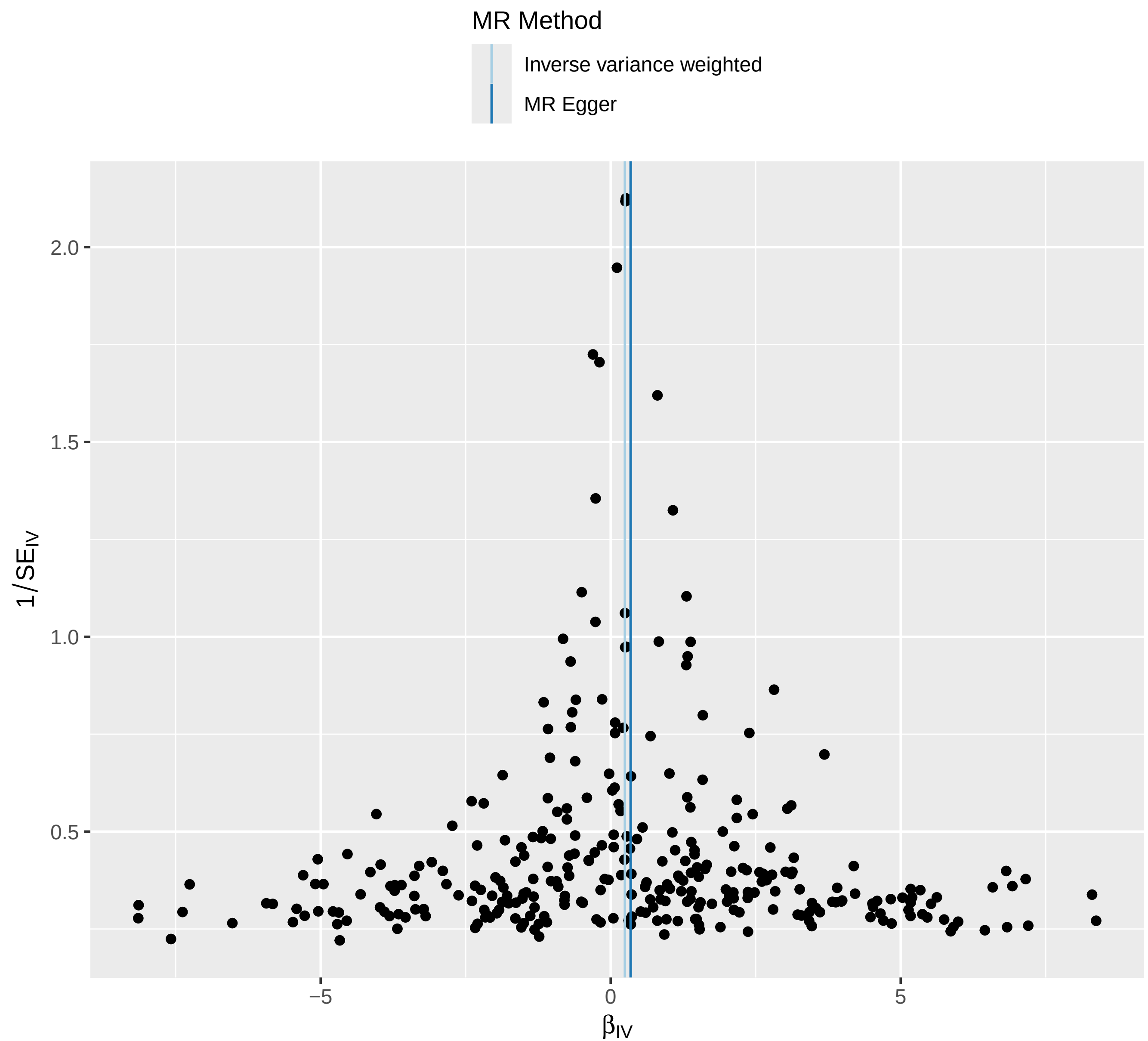
MR Method
Inverse variance weighted
MR Egger
2.0
1.5
1.0
0.5
1/SE_IV
−5
0
5
β_IV

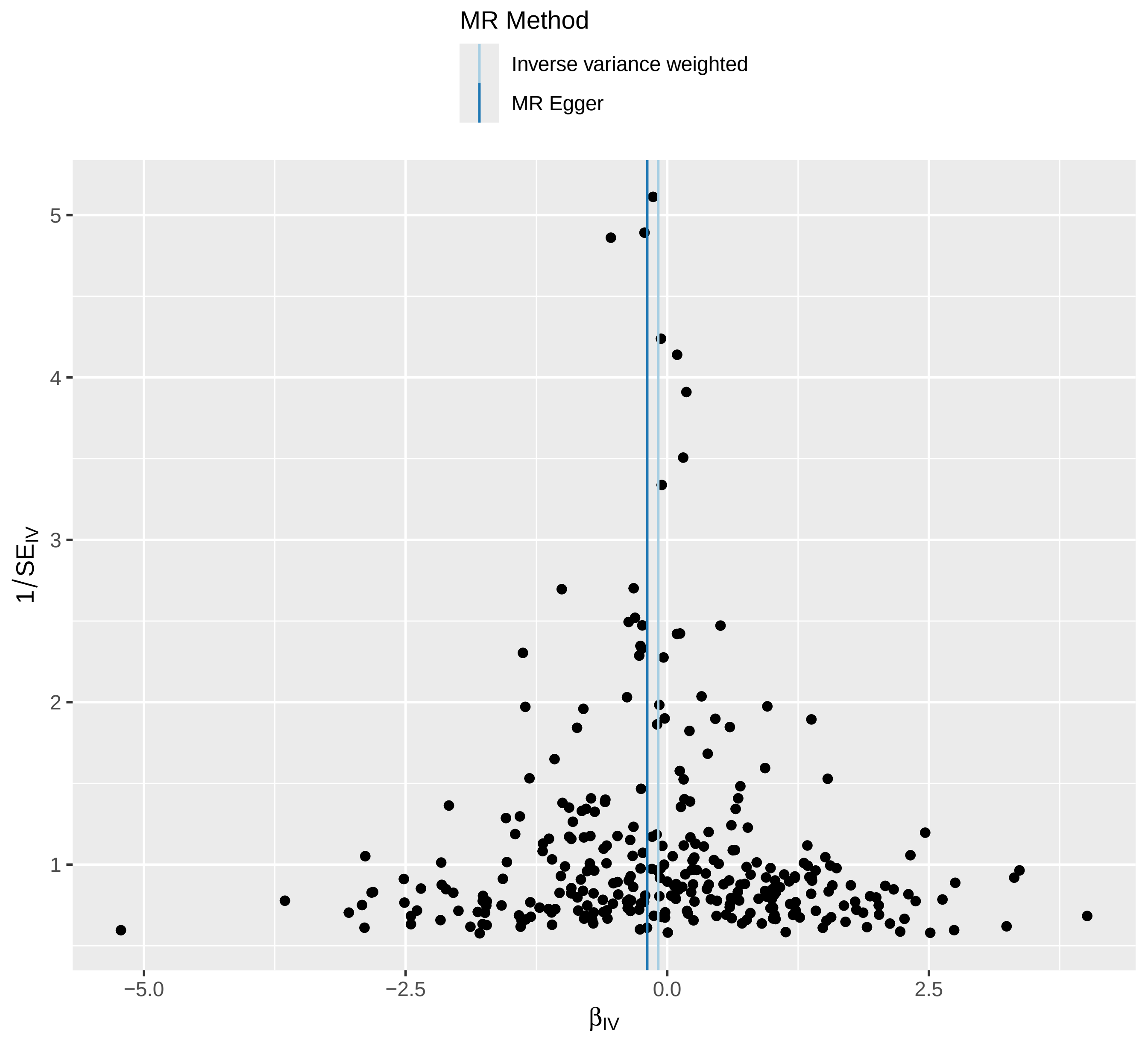

MR Method
Inverse variance weighted
MR Egger
5
4
3
2
1
$1/SE_{IV}$
−5.0
−2.5
0.0
2.5
β_{IV}

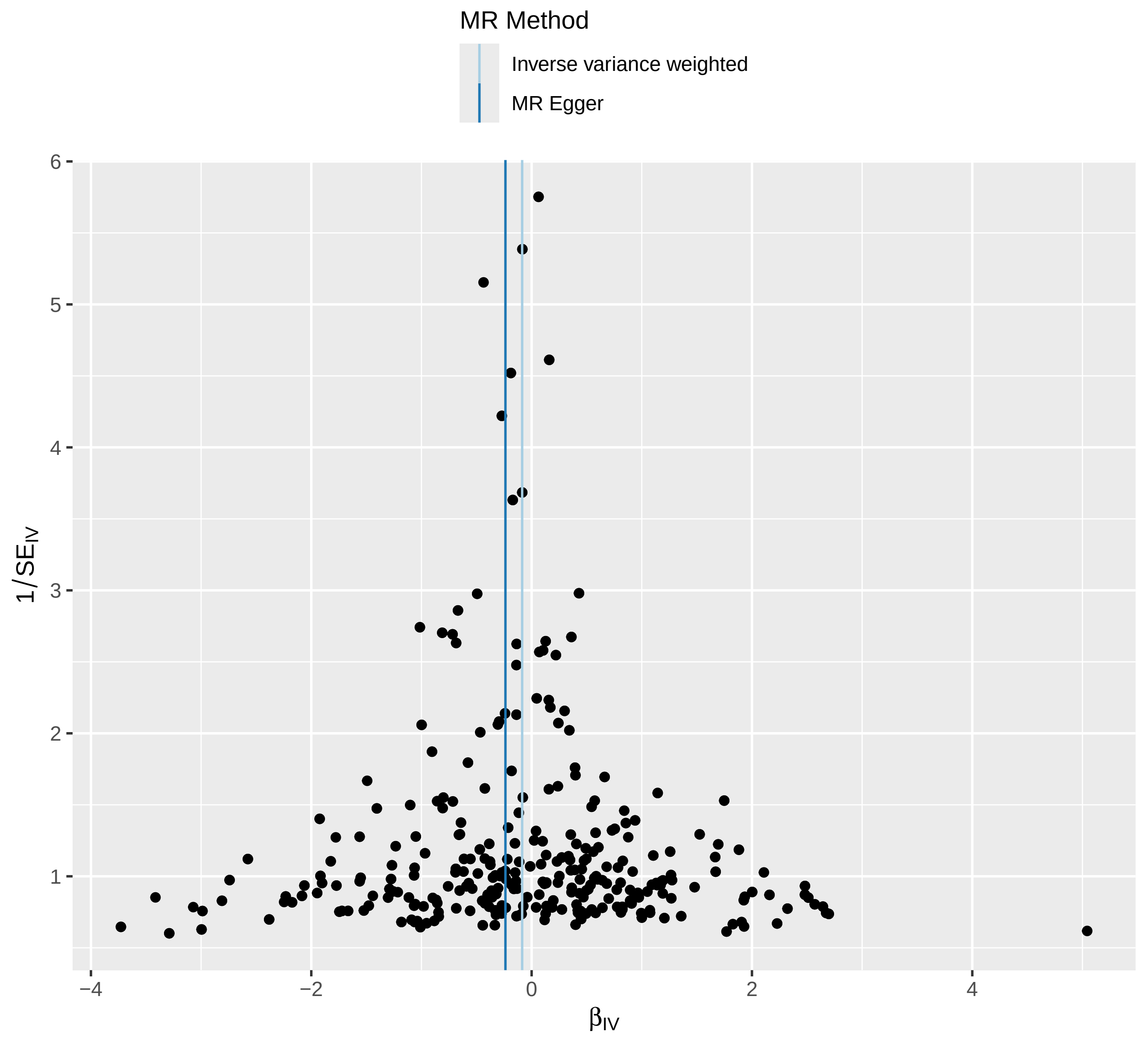

MR Method
Inverse variance weighted
MR Egger
6
5
4
3
2
1
1/SE_IV
−4
−2
0
2
4
β_IV

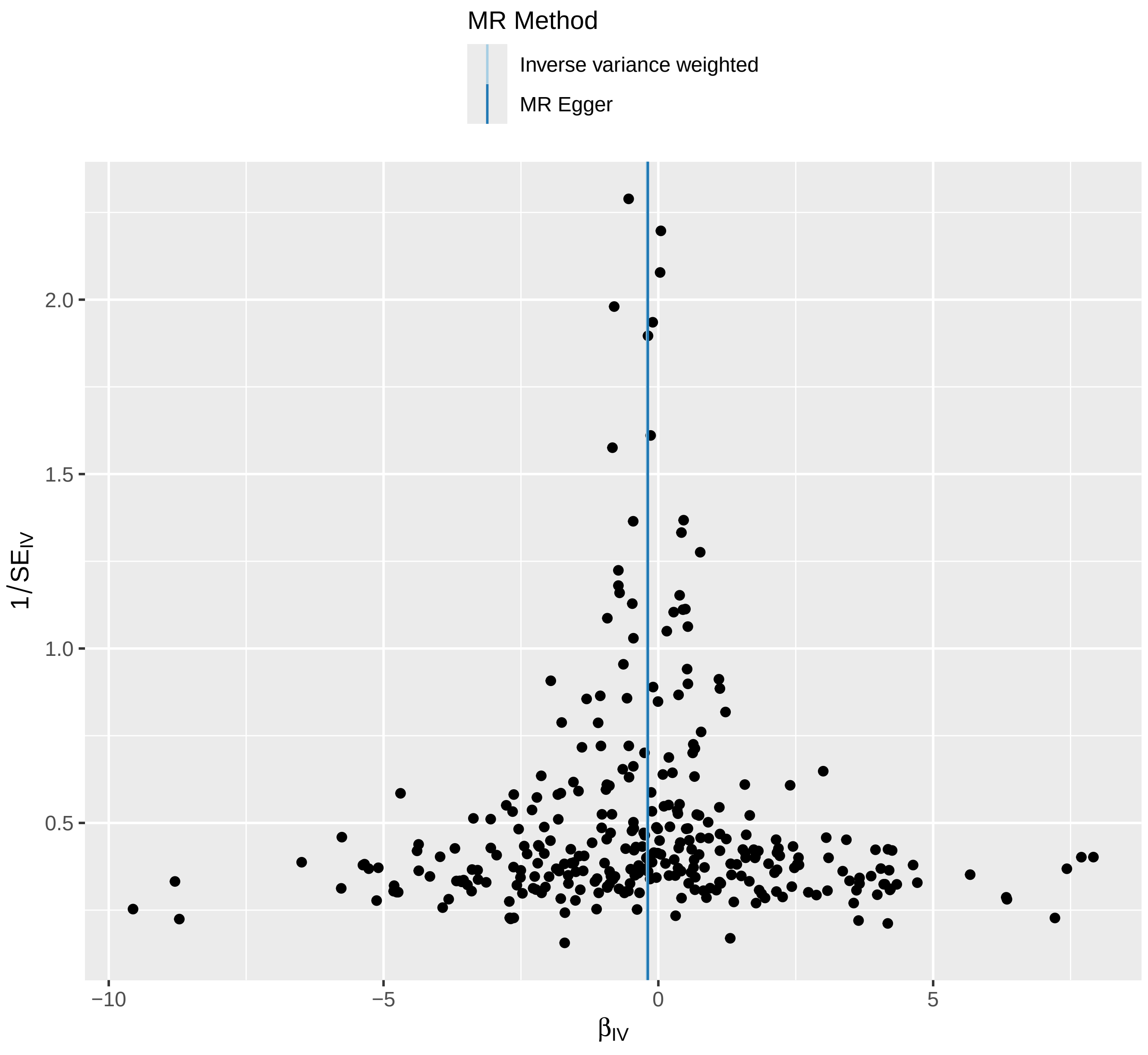

MR Method
Inverse variance weighted
MR Egger
2.0
1.5
1.0
0.5
1/SE_IV
−10
−5
0
5
β_IV

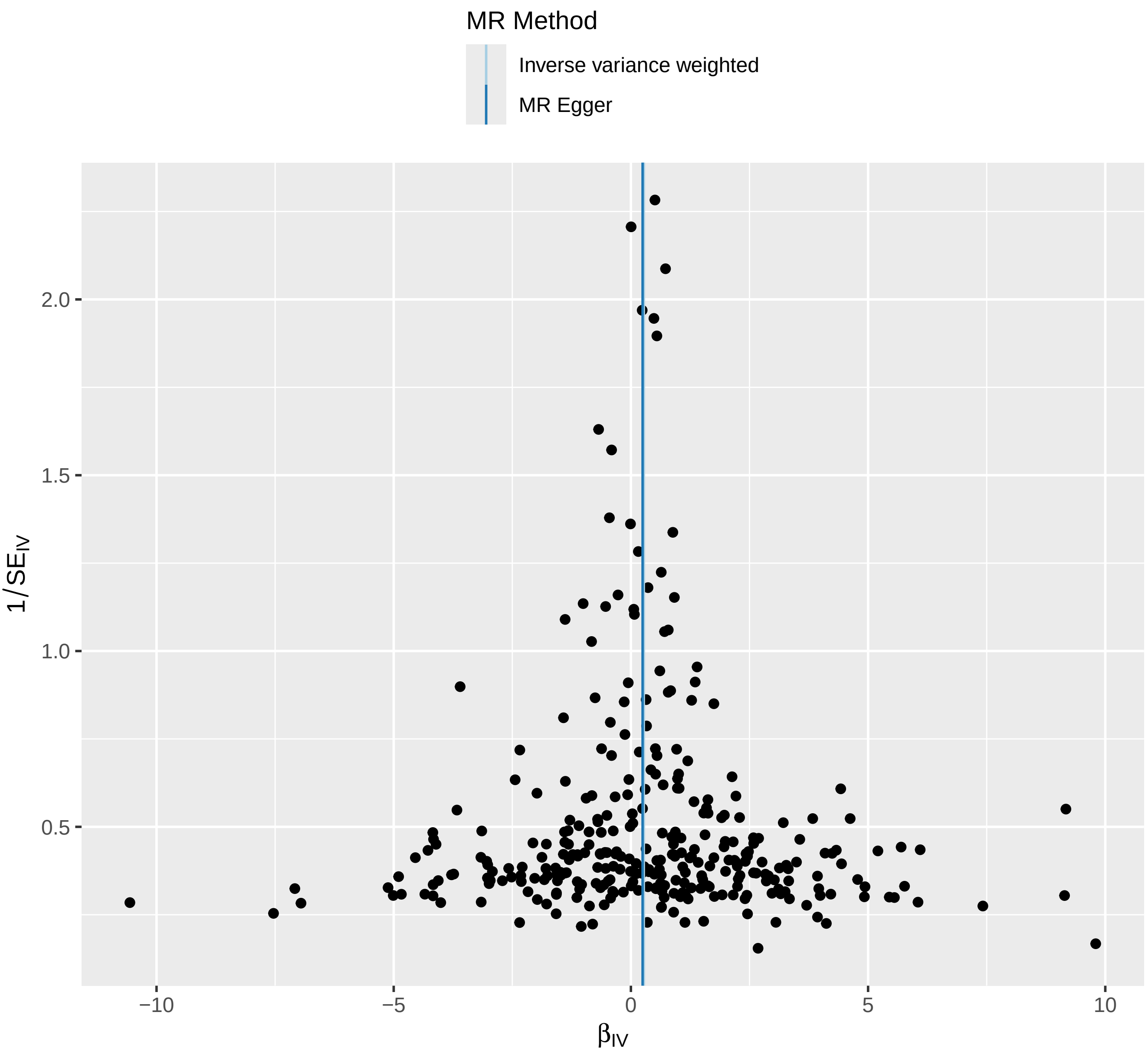
MR Method
Inverse variance weighted
MR Egger
1/SE_IV
2.0
1.5
1.0
0.5
−10
−5
0
5
10
β_IV

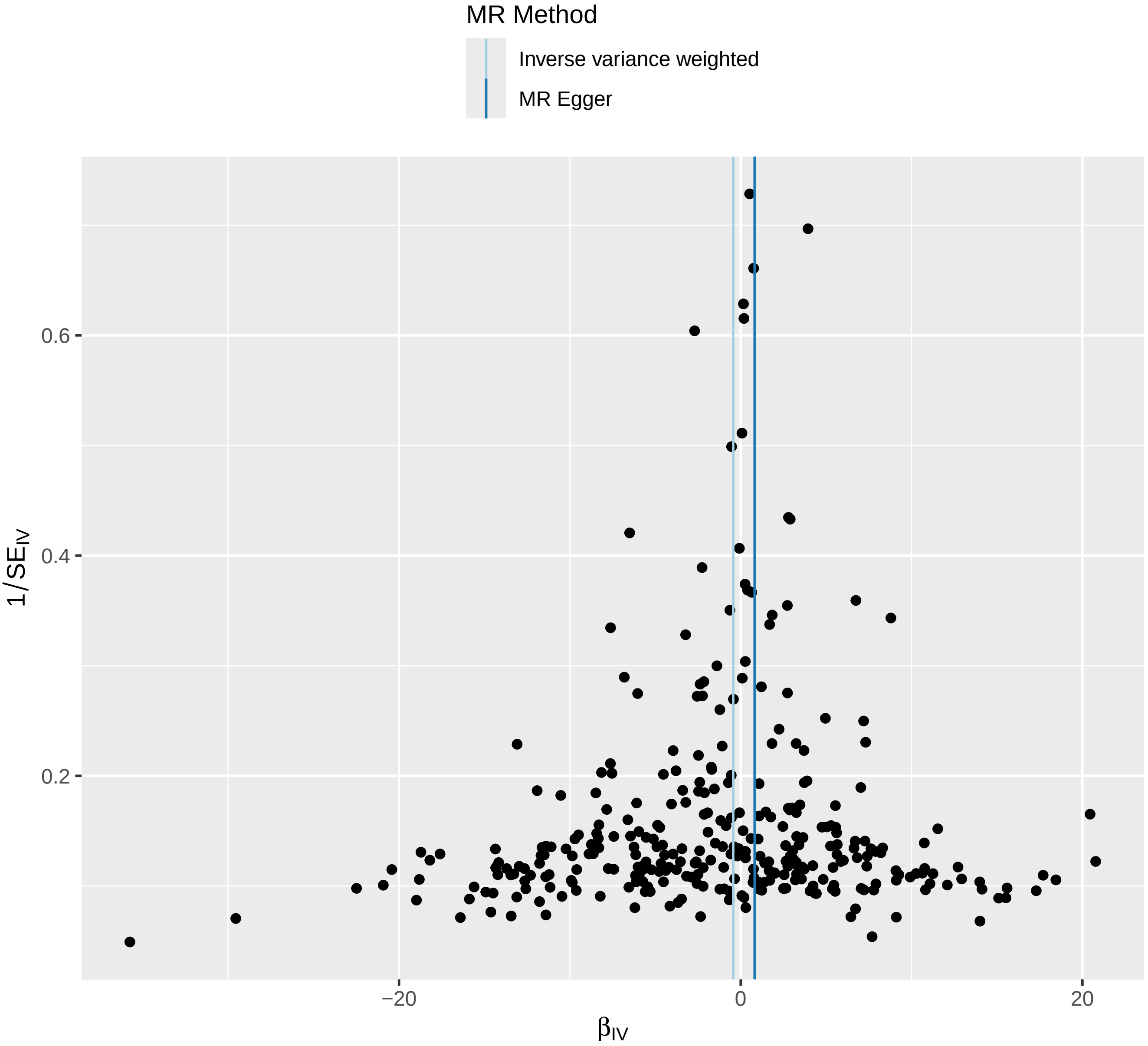

MR Method
Inverse variance weighted
MR Egger
0.6
0.4
0.2
1/SE_IV
−20
0
20
β_IV

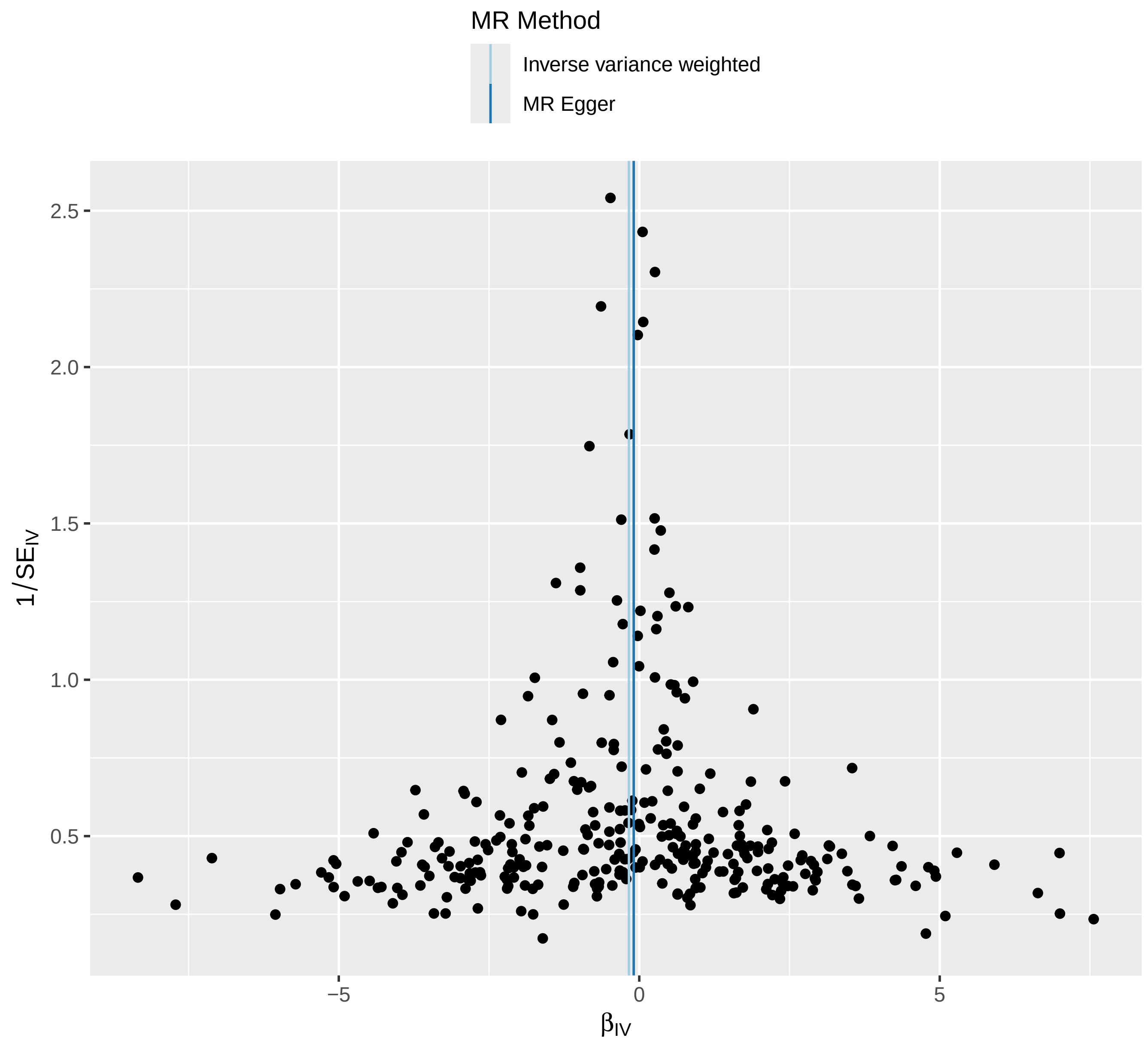

MR Method
Inverse variance weighted
MR Egger
2.5
2.0
1.5
1.0
0.5
1/SE_IV
−5
0
5
β_IV

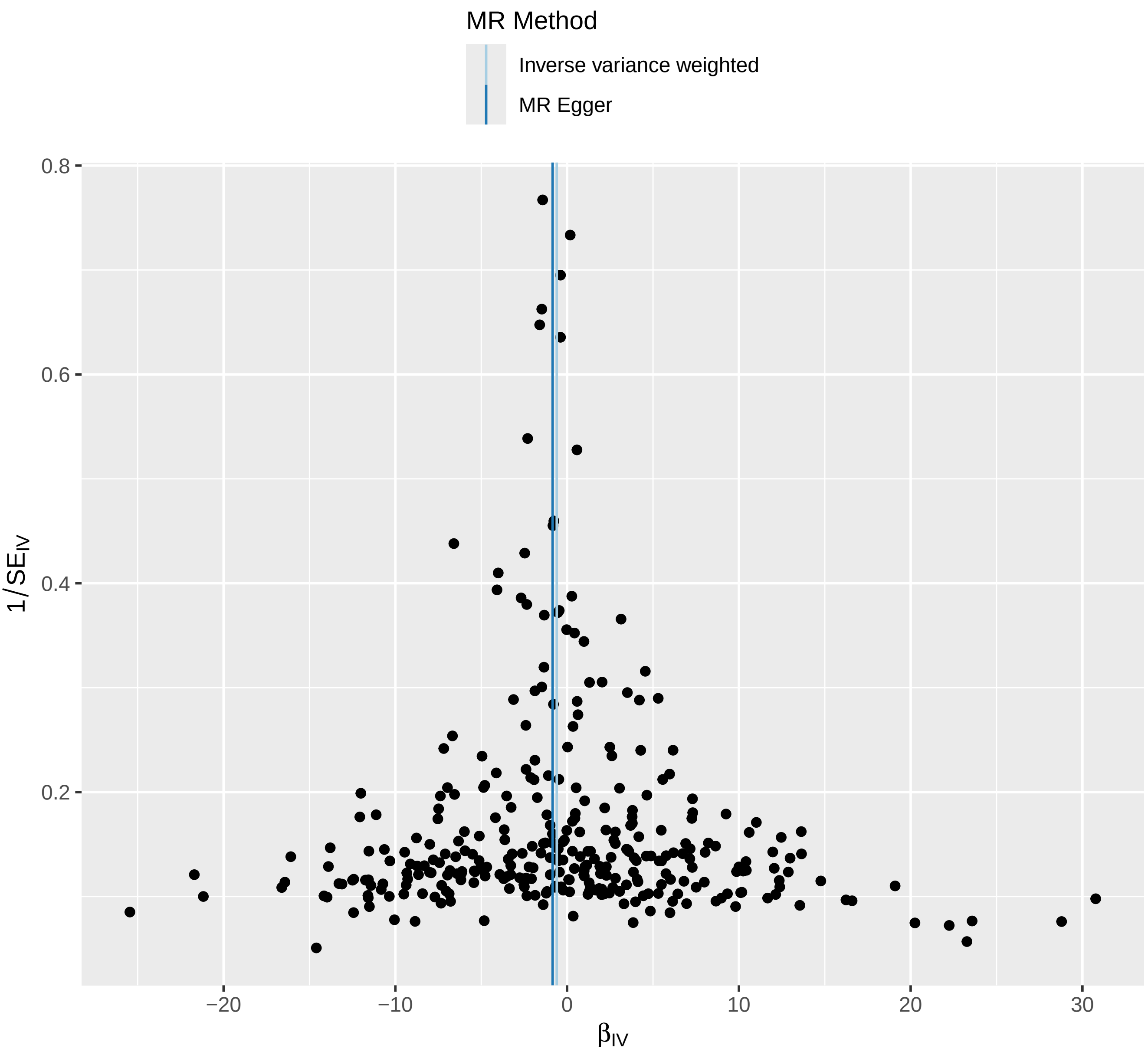
MR Method
Inverse variance weighted
MR Egger
0.8
0.6
0.4
0.2
$1/SE_{IV}$
−20
−10
0
10
20
30
β_{IV}

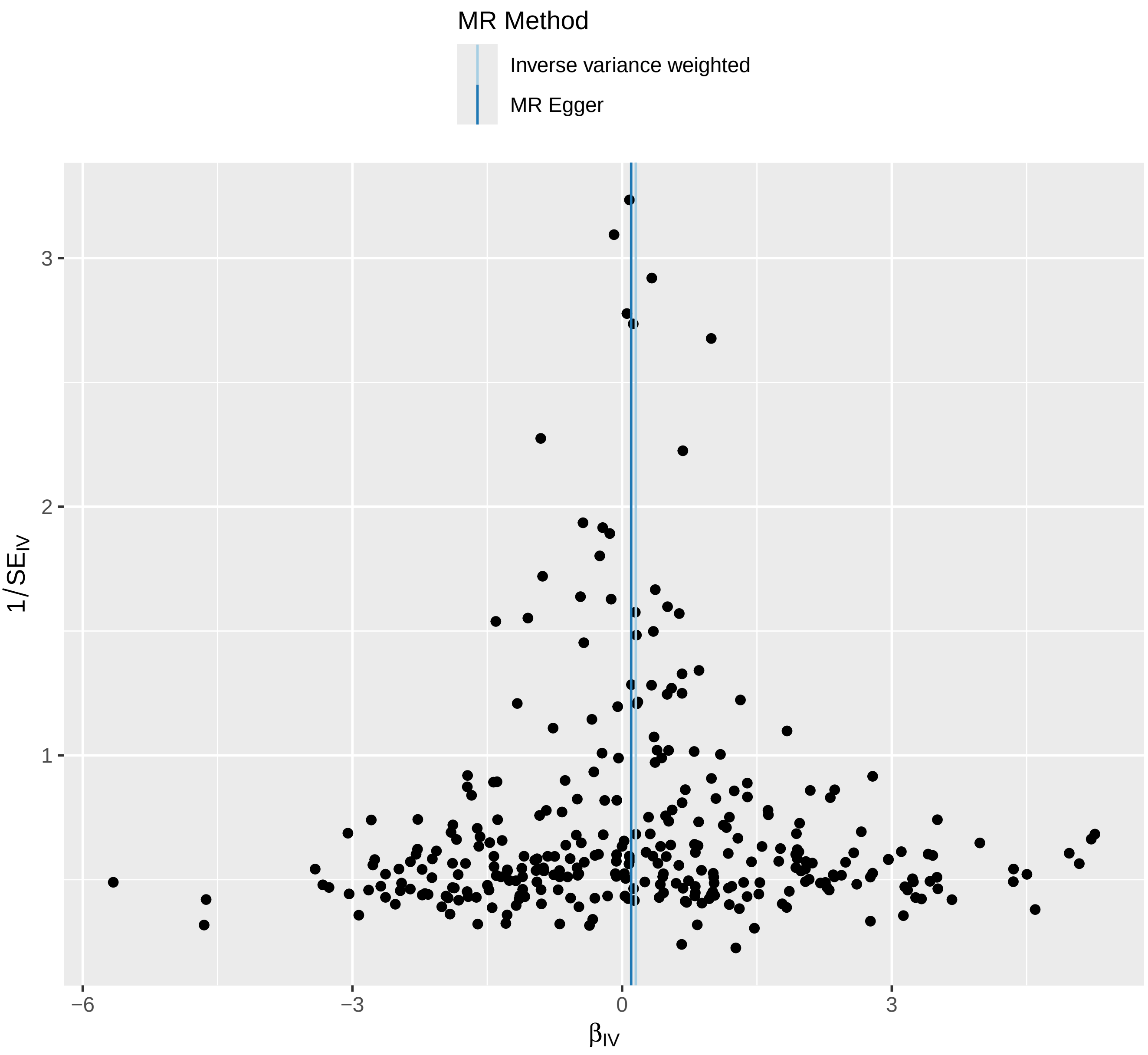
MR Method
Inverse variance weighted
MR Egger
1/SE_IV
3
2
1
−6
−3
0
3
β_IV

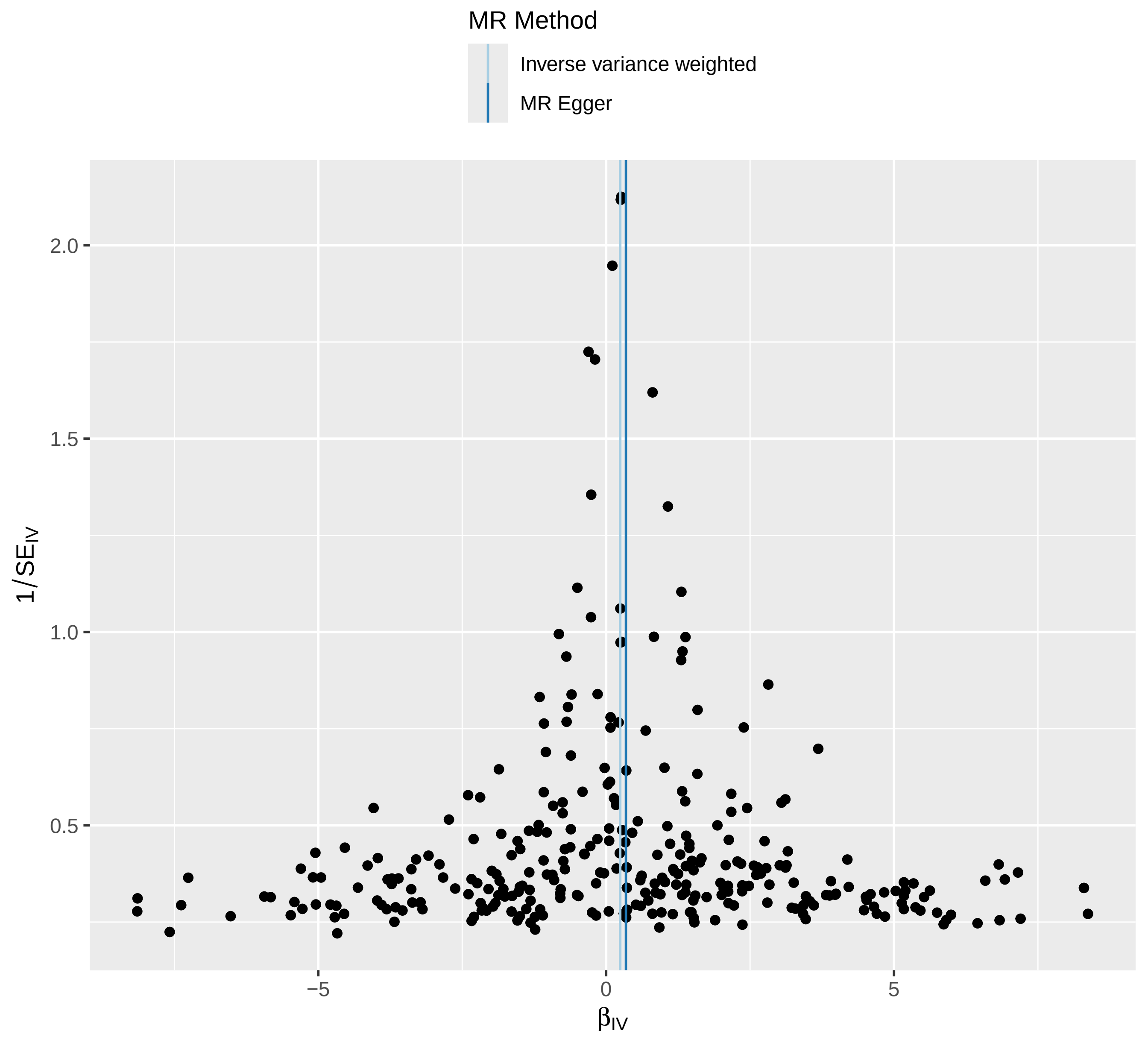

MR Method
Inverse variance weighted
MR Egger
2.0
1.5
1.0
0.5
−5
0
5
$1/SE_{IV}$
β_{IV}

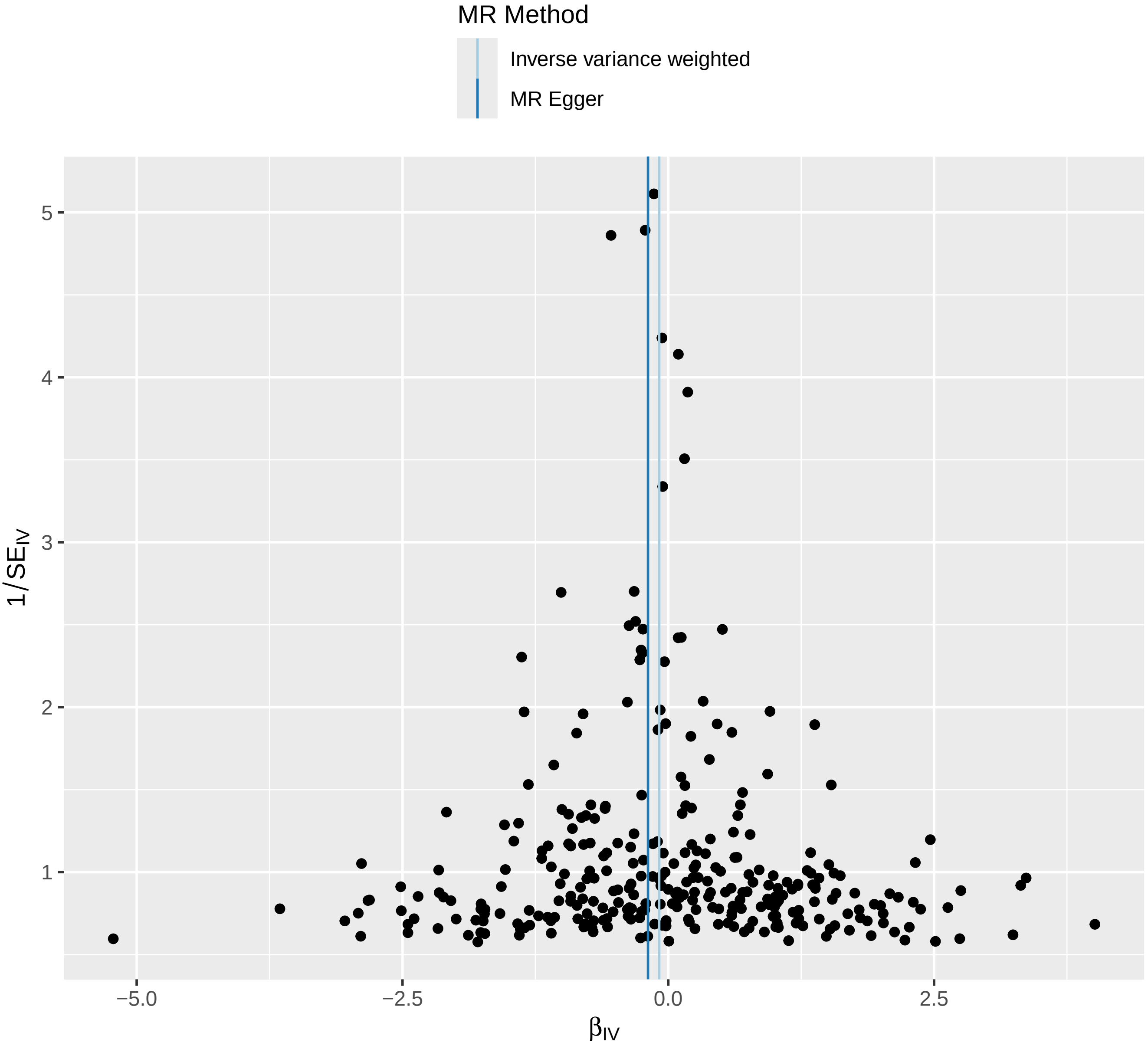

MR Method
Inverse variance weighted
MR Egger
1/SE$_{IV}$
5
4
3
2
1
−5.0
−2.5
0.0
2.5
β$_{IV}$

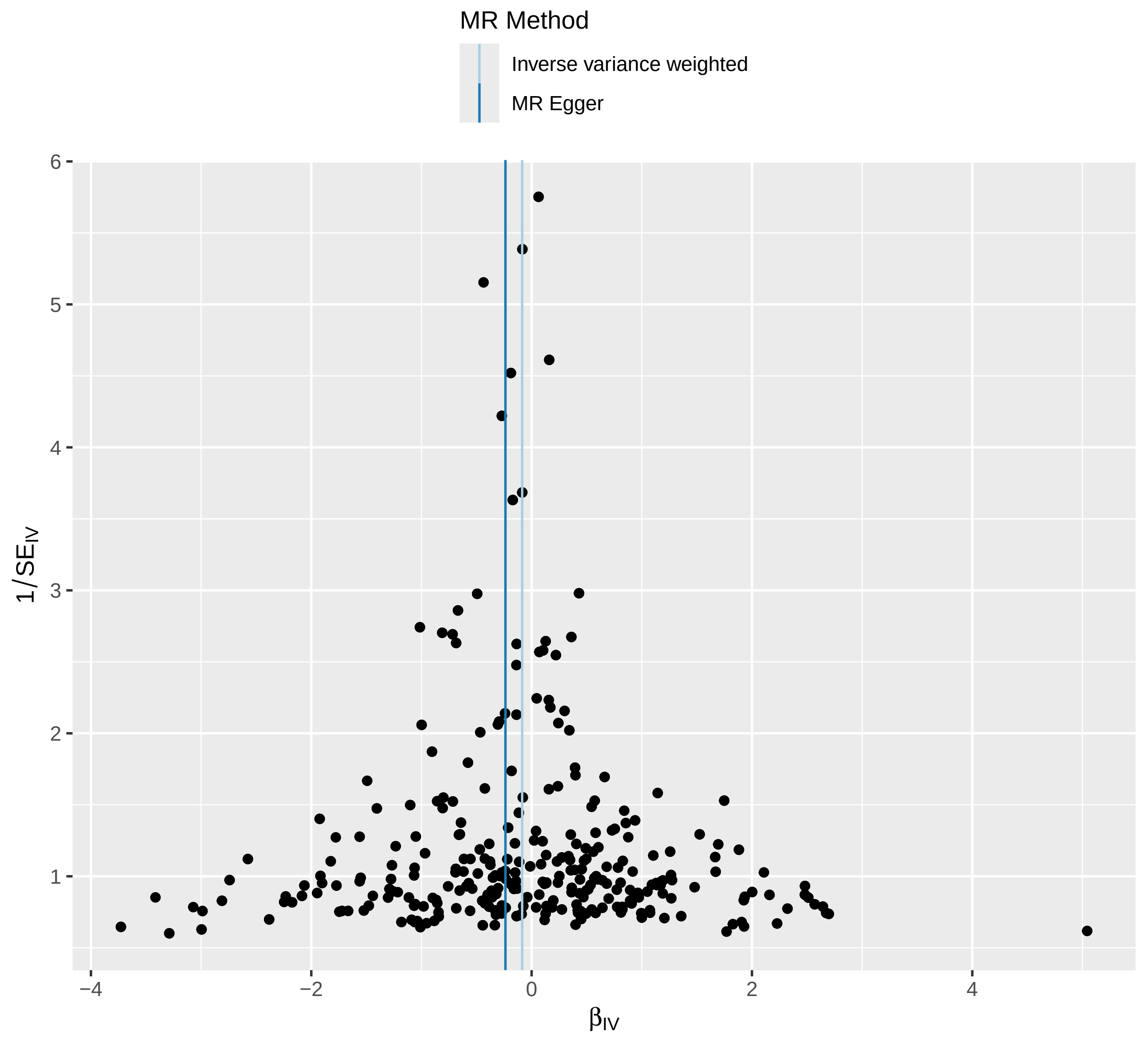
MR Method
Inverse variance weighted
MR Egger
$1/SE_{IV}$
β_{IV}
6
5
4
3
2
1
−4
−2
0
2
4

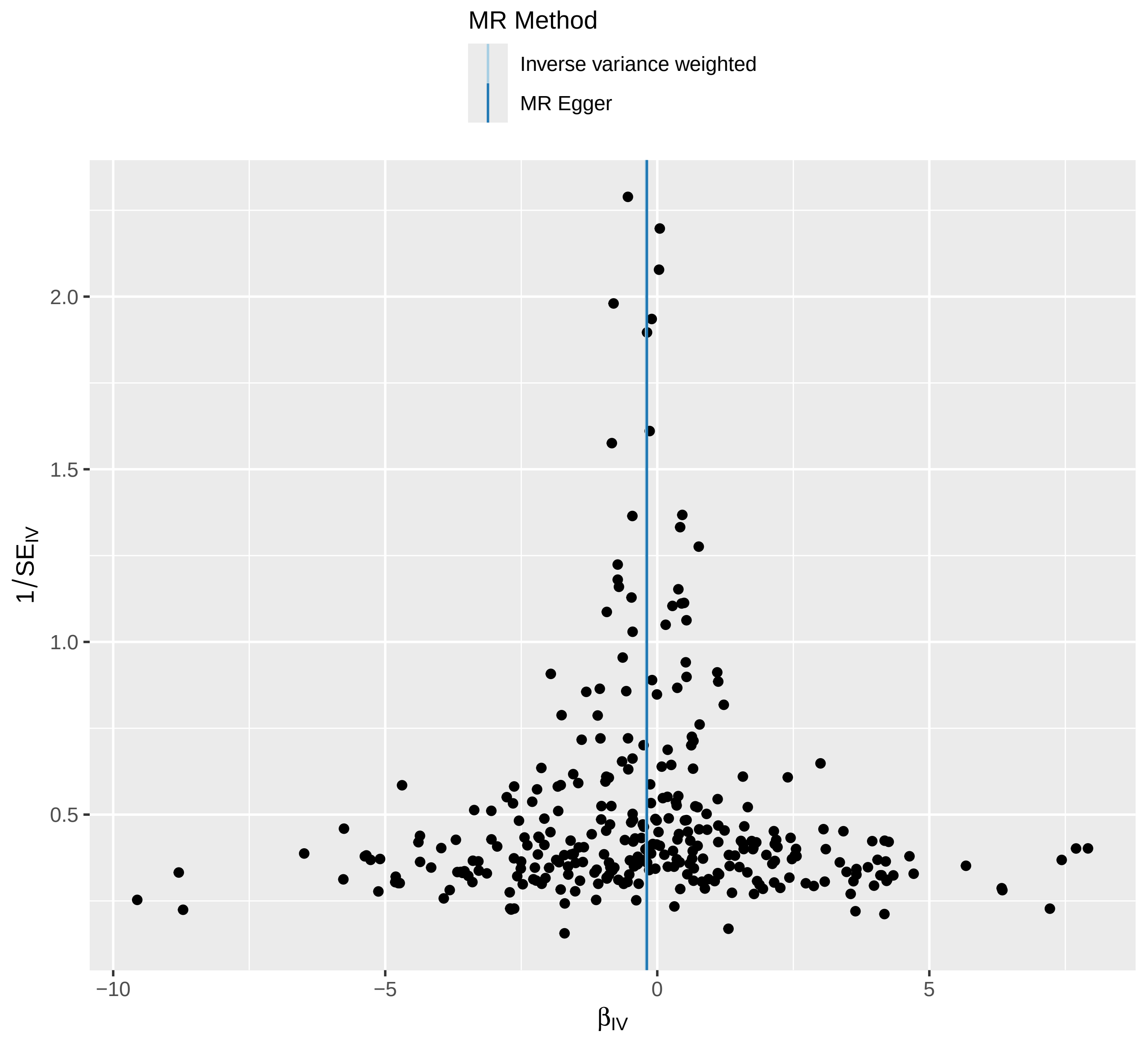
MR Method
Inverse variance weighted
MR Egger
1/SE_IV
2.0
1.5
1.0
0.5
−10
−5
0
5
β_IV

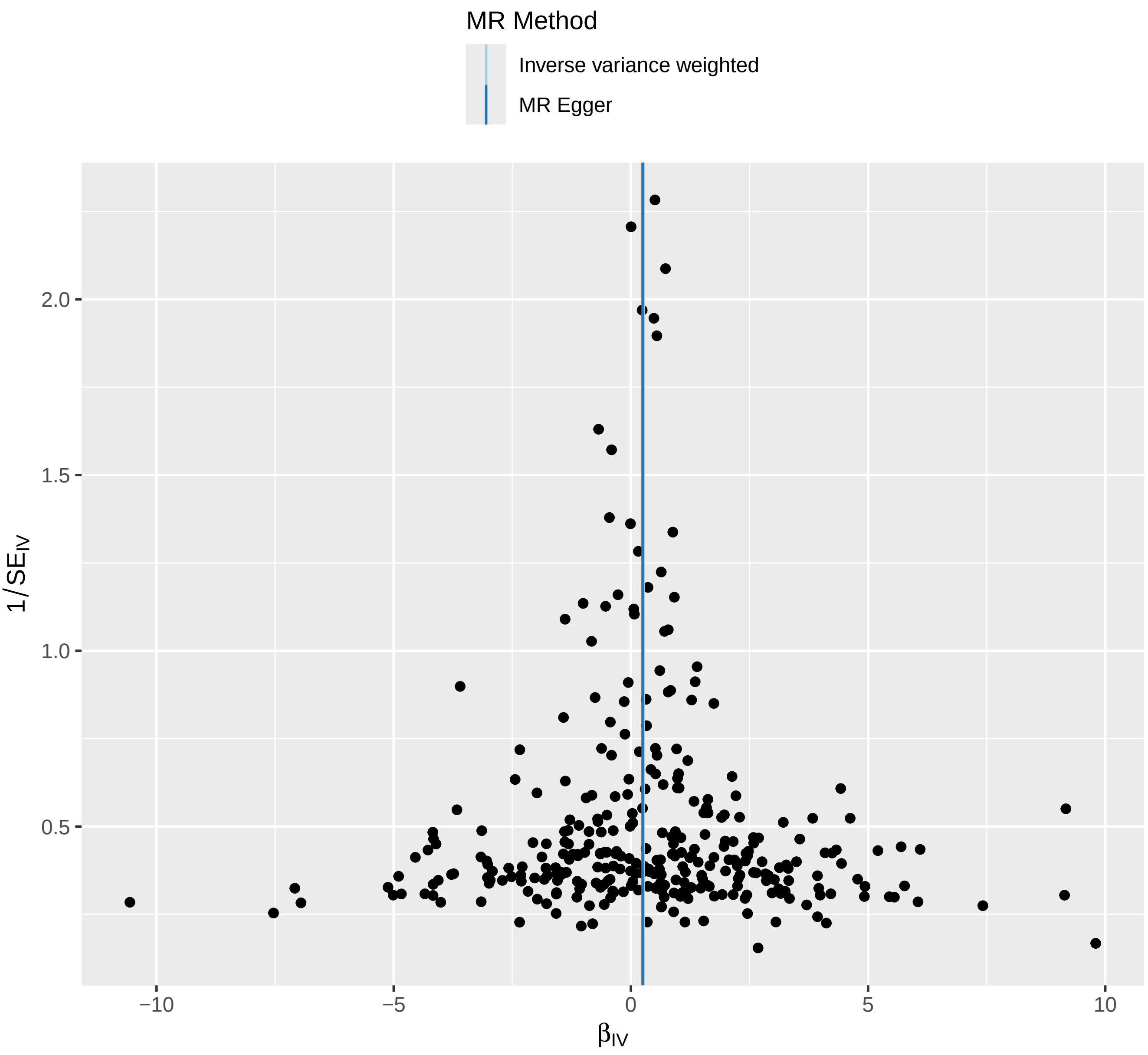

MR Method
Inverse variance weighted
MR Egger
2.0
1.5
1.0
0.5
1/SE_IV
−10
−5
0
5
10
β_IV

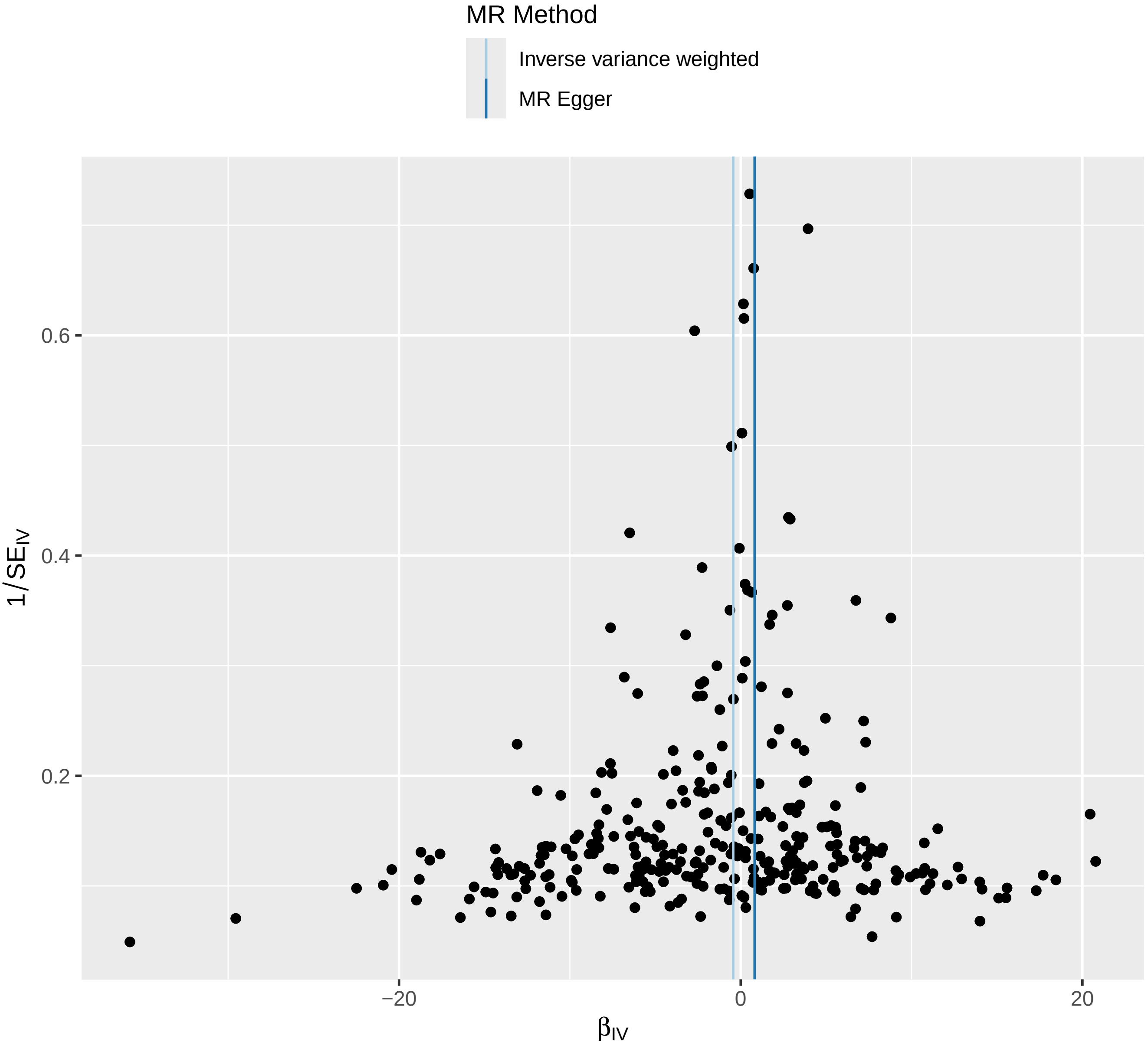

MR Method
Inverse variance weighted
MR Egger
1/SE_IV
0.6
0.4
0.2
−20
0
20
β_IV

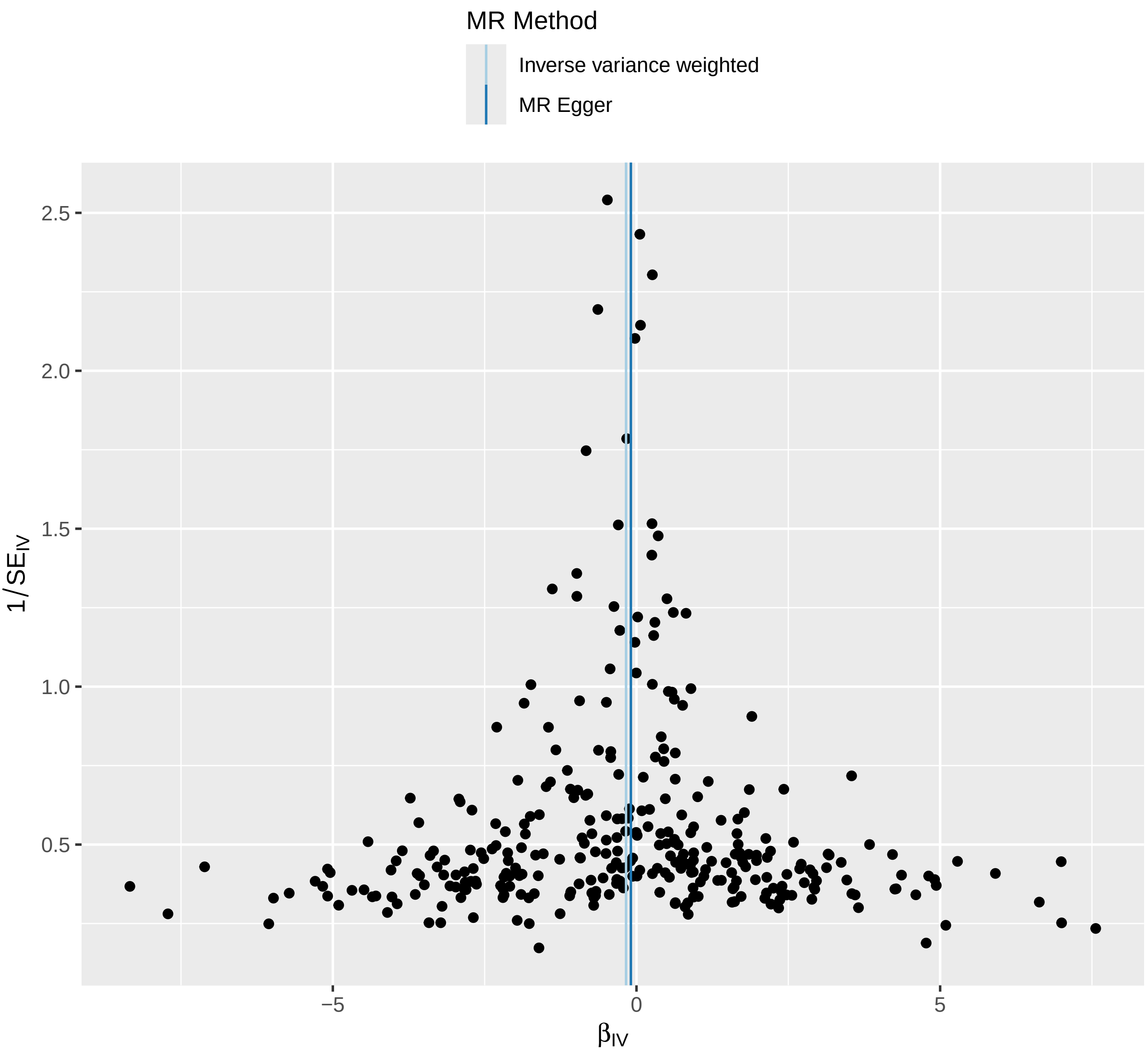
MR Method
Inverse variance weighted
MR Egger
2.5
2.0
1.5
1.0
0.5
$1/SE_{IV}$
−5
0
5
β_{IV}

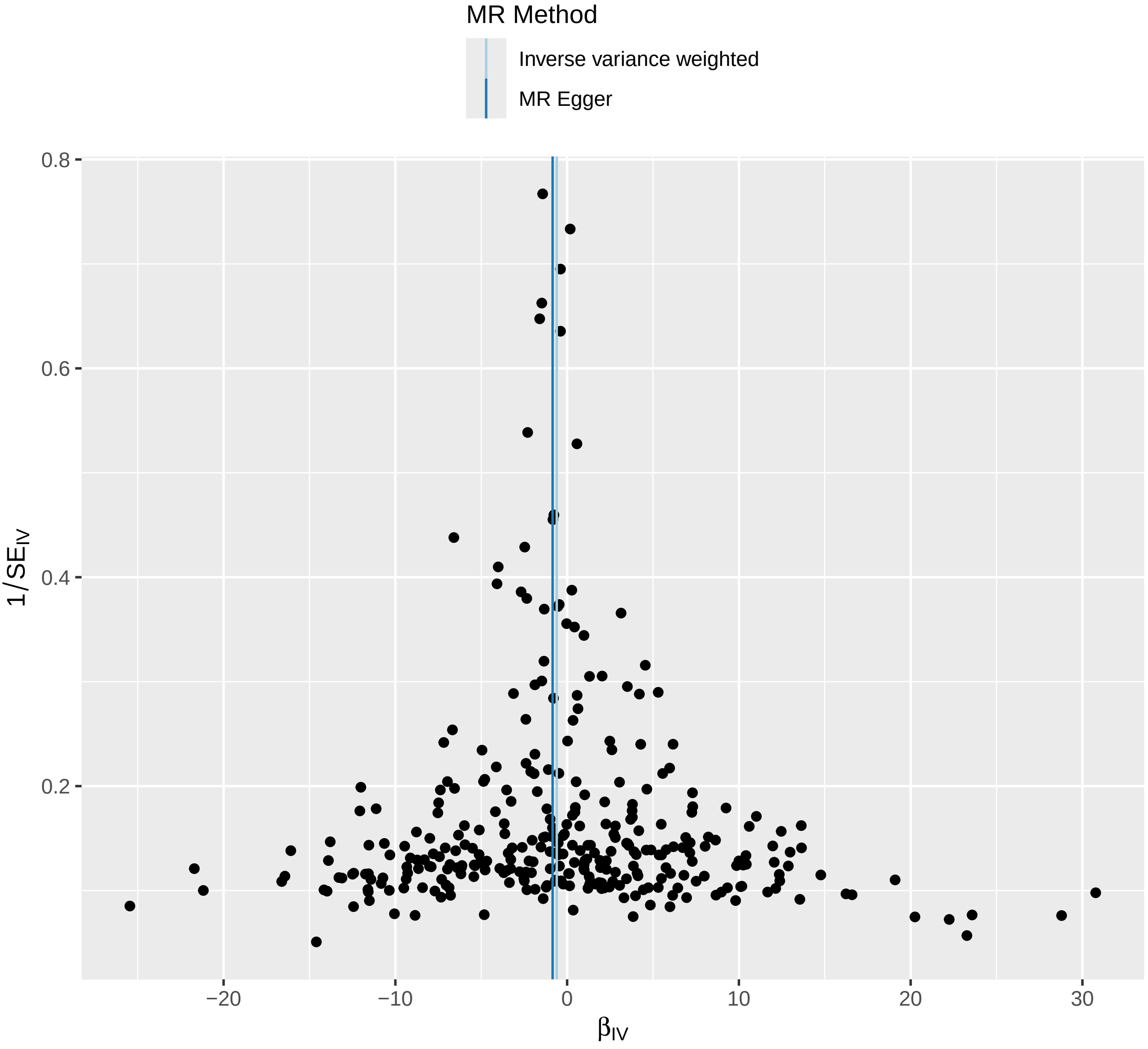
MR Method
Inverse variance weighted
MR Egger
0.8
0.6
0.4
0.2
1/SE_IV
−20
−10
0
10
20
30
β_IV

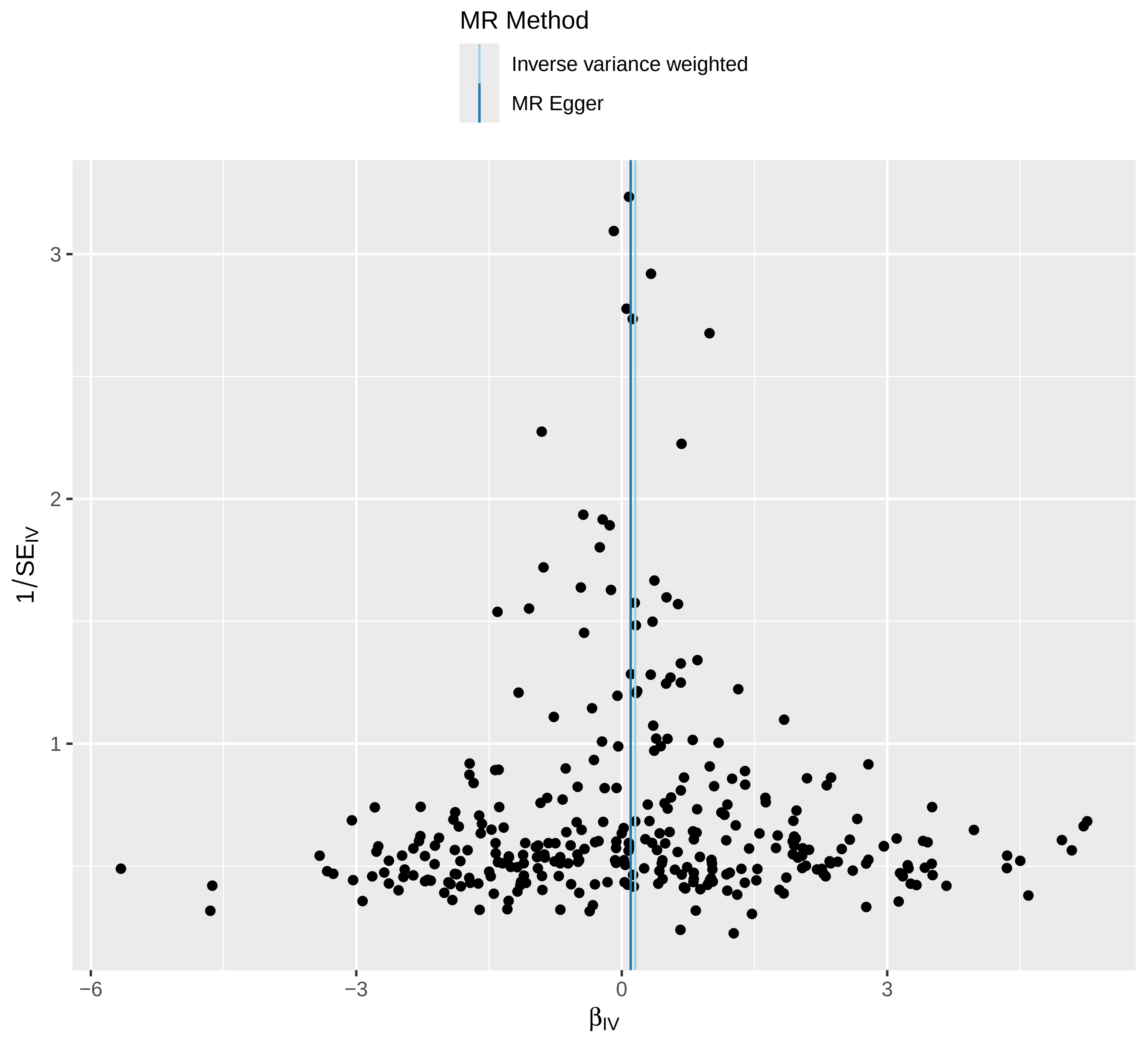
MR Method
Inverse variance weighted
MR Egger
1/SE_IV
3
2
1
−6
−3
0
3
β_IV

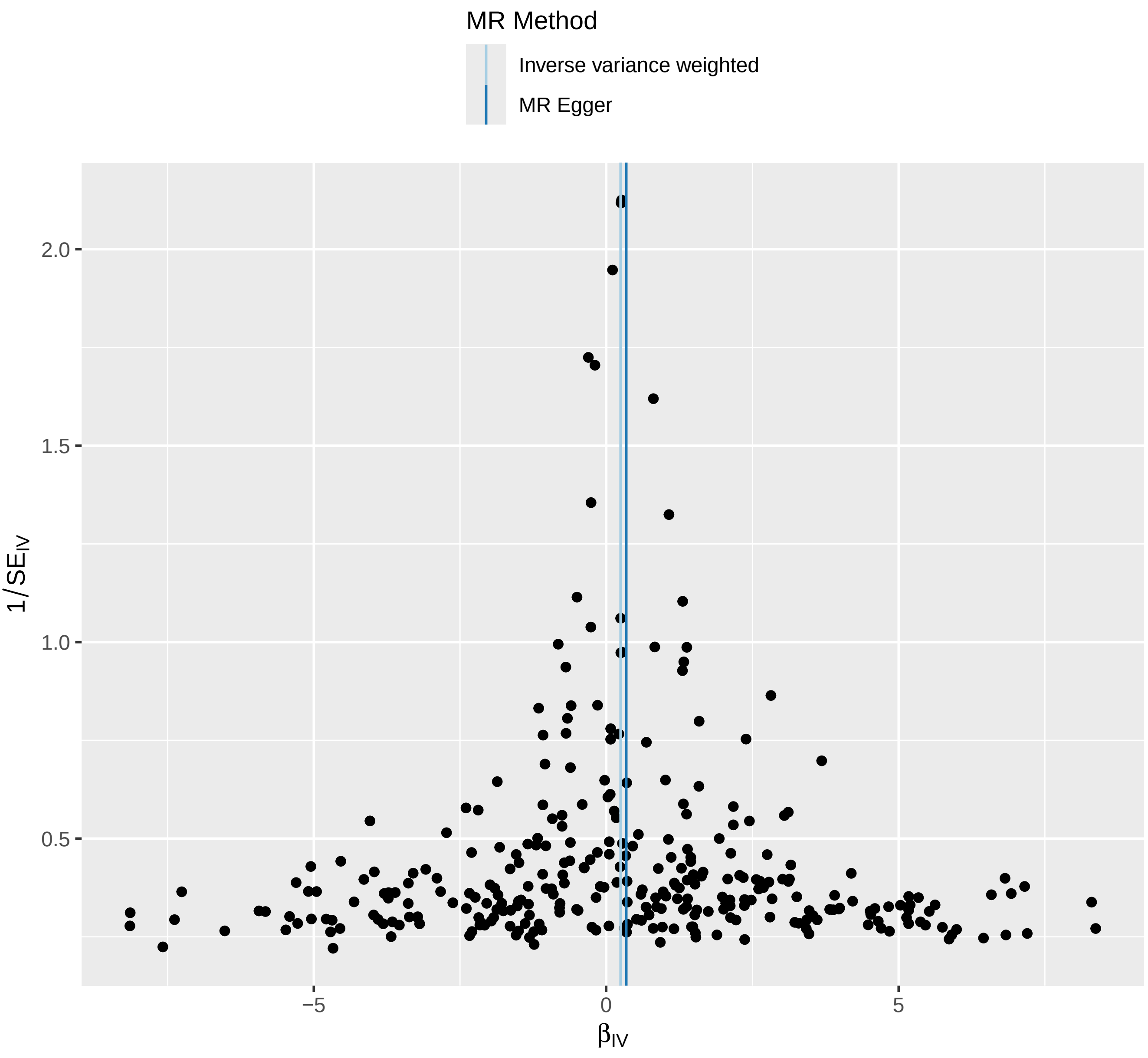
MR Method
Inverse variance weighted
MR Egger
2.0
1.5
1.0
0.5
$1/SE_{IV}$
−5
0
5
β_{IV}

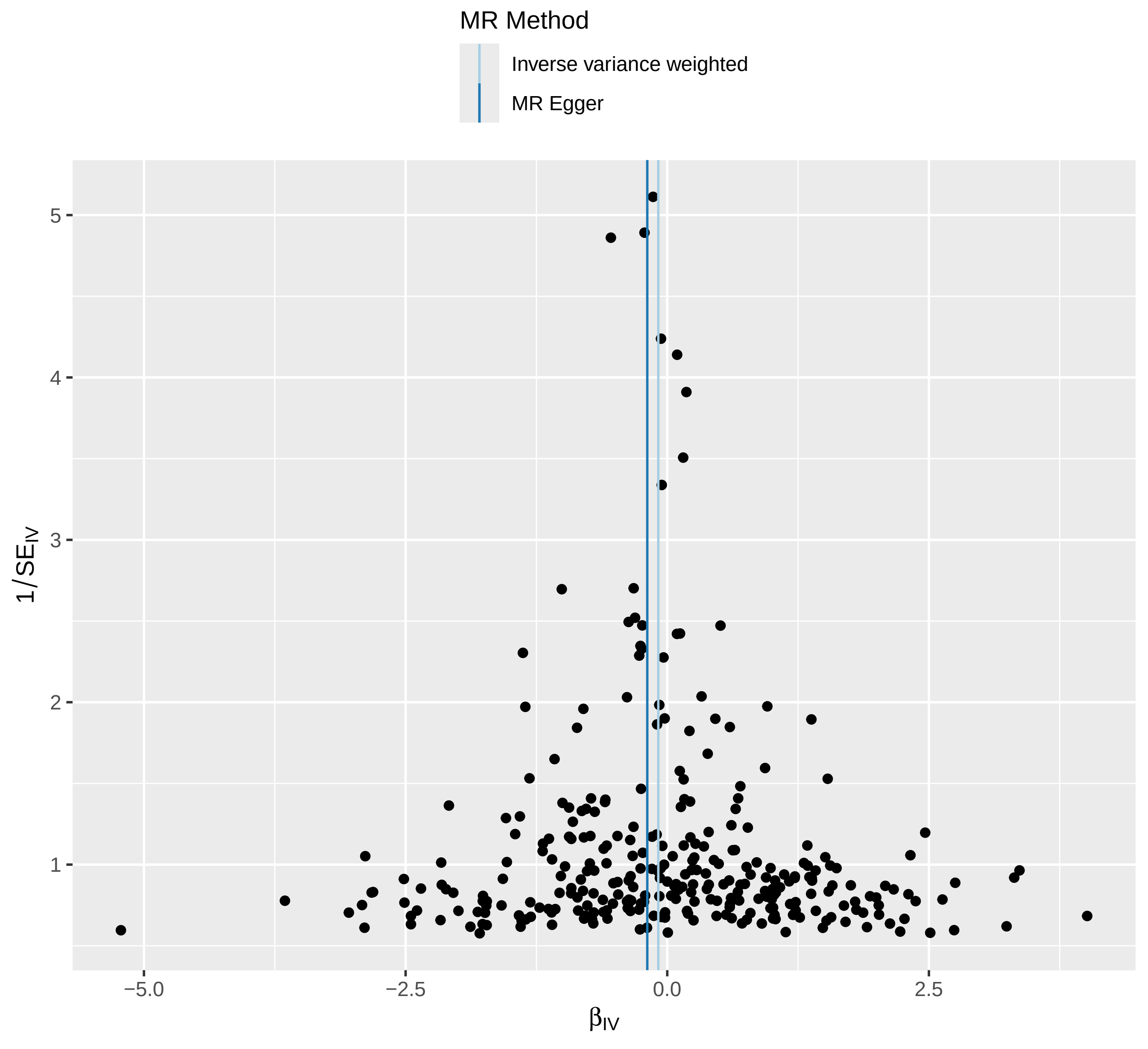

MR Method
Inverse variance weighted
MR Egger
5
4
3
2
1
1/SE$_{IV}$
−5.0
−2.5
0.0
2.5
β$_{IV}$

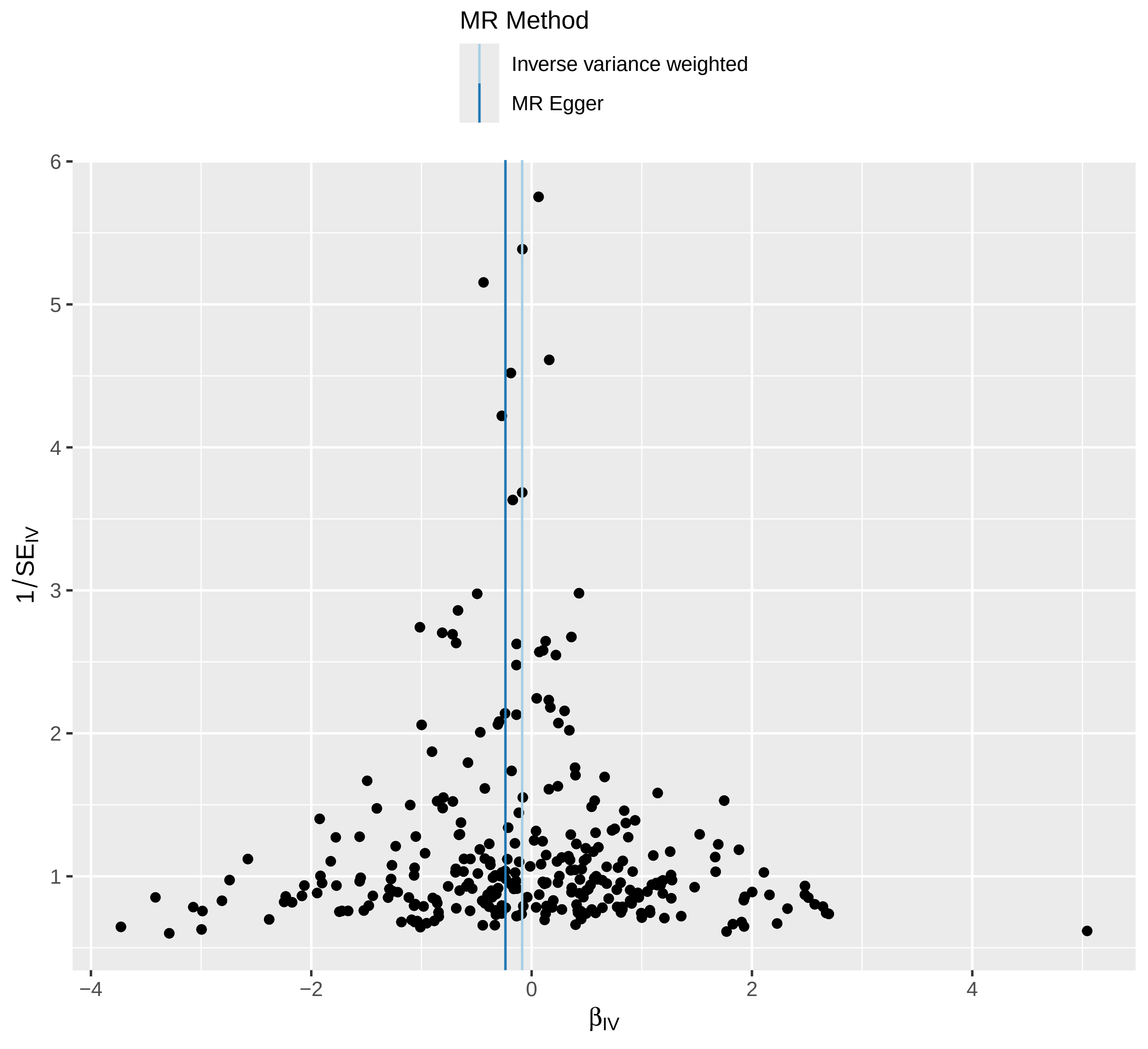

MR Method
Inverse variance weighted
MR Egger
$1/SE_{IV}$
β_{IV}
6
5
4
3
2
1
−4
−2
0
2
4

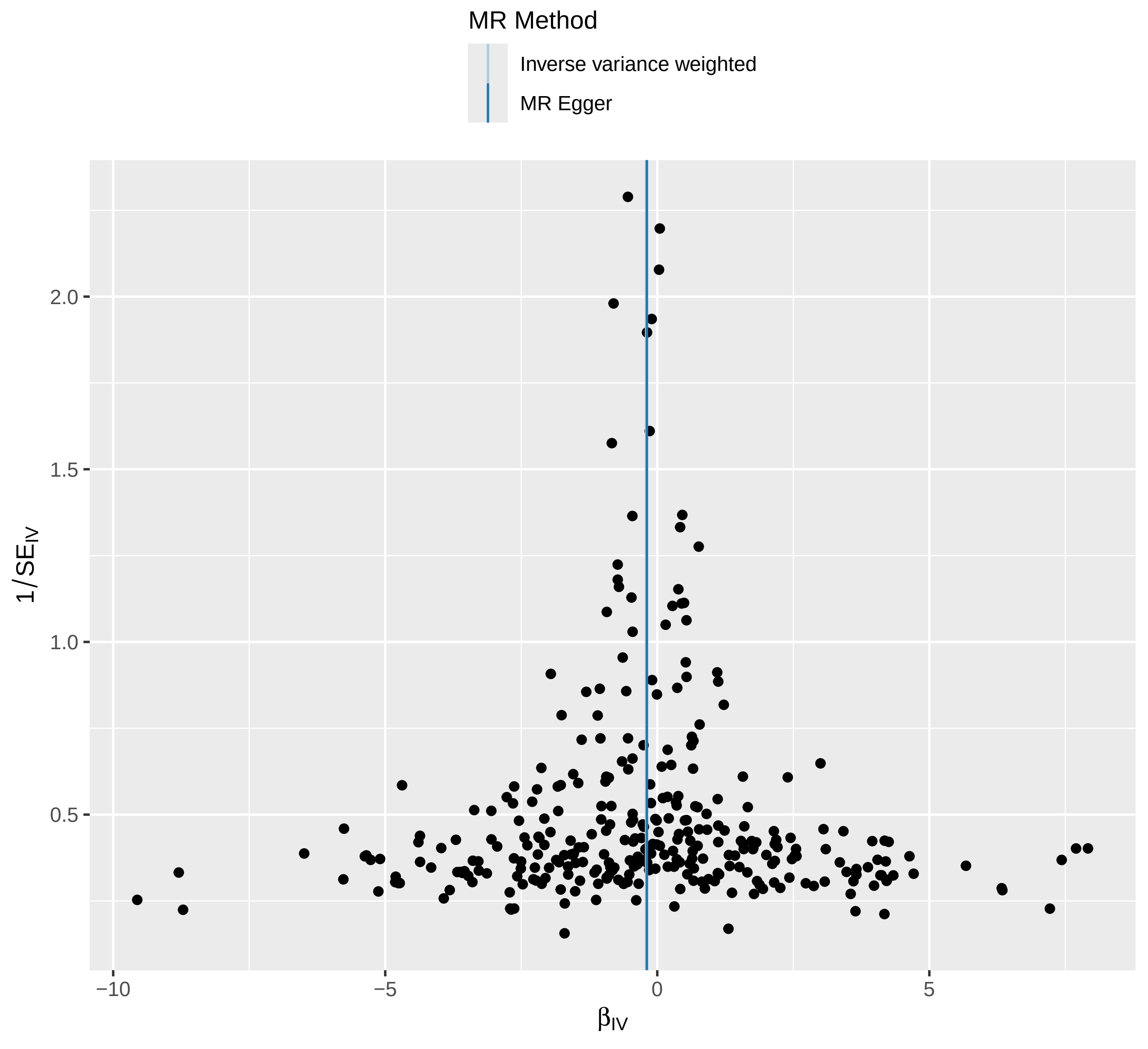
MR Method
Inverse variance weighted
MR Egger
1/SE_IV
2.0
1.5
1.0
0.5
−10
−5
0
5
β_IV

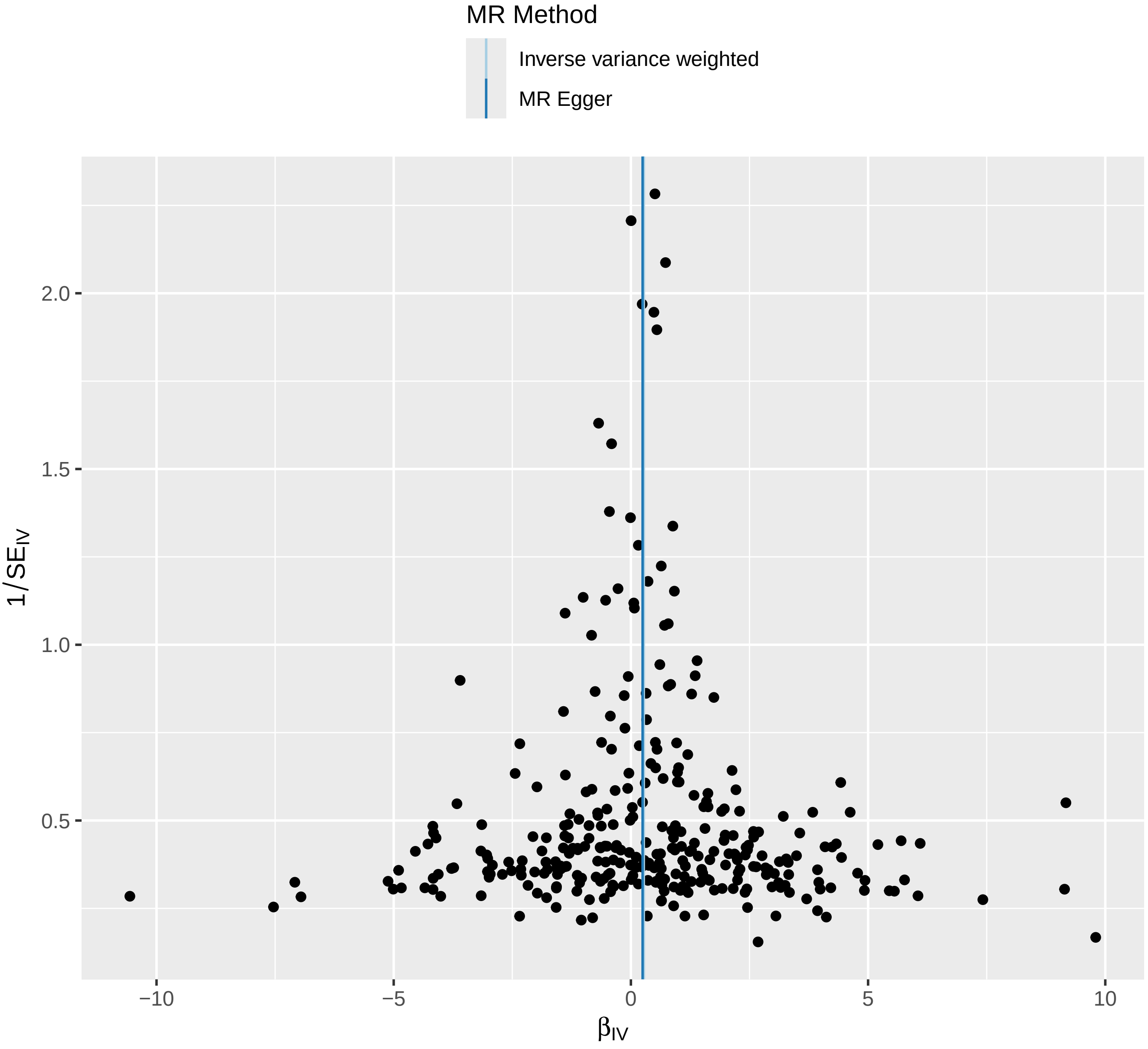
MR Method
Inverse variance weighted
MR Egger
2.0
1.5
1.0
0.5
1/SE_IV
−10
−5
0
5
10
β_IV

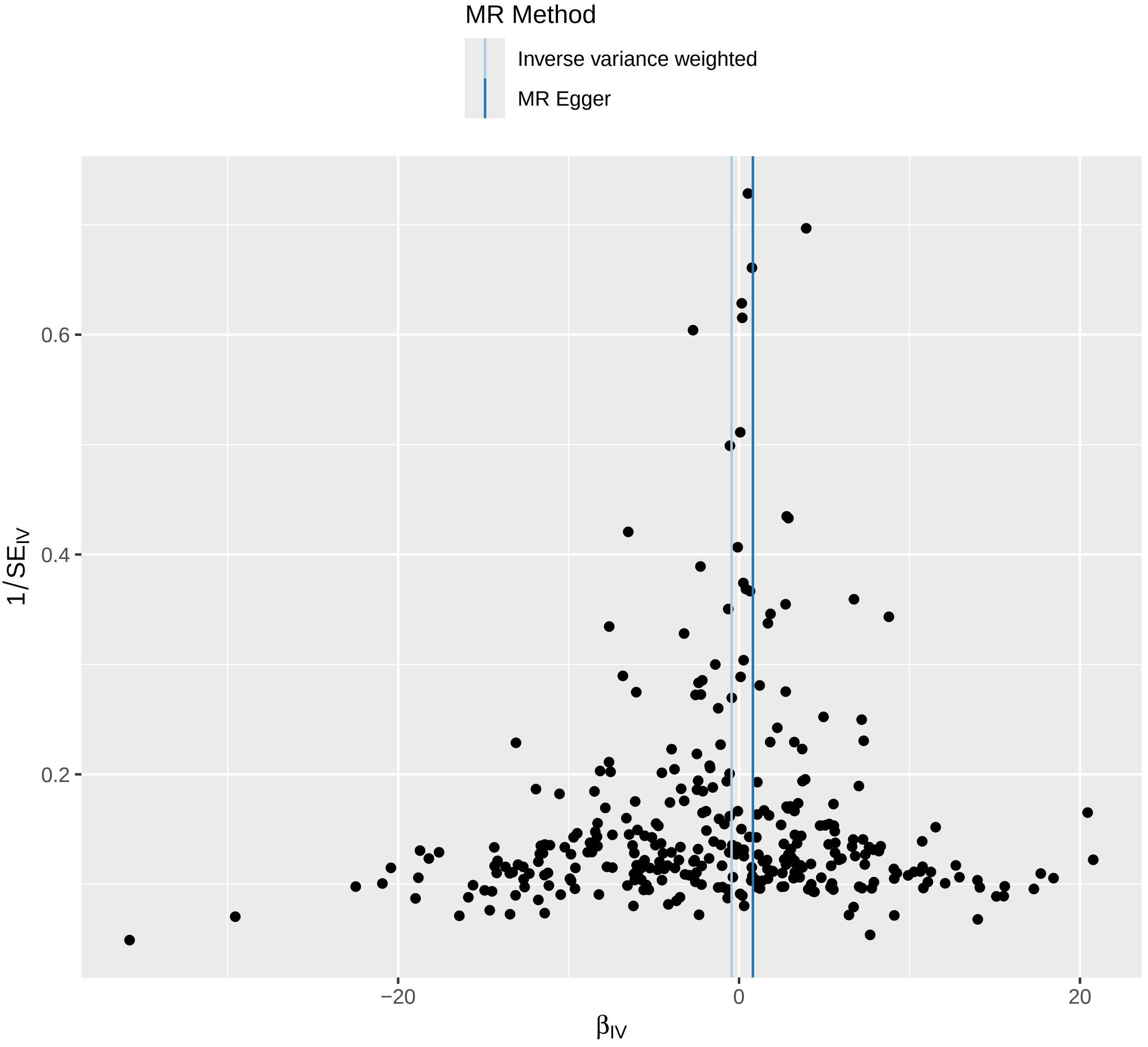

MR Method
Inverse variance weighted
MR Egger
1/SE_IV
0.6
0.4
0.2
−20
0
20
β_IV

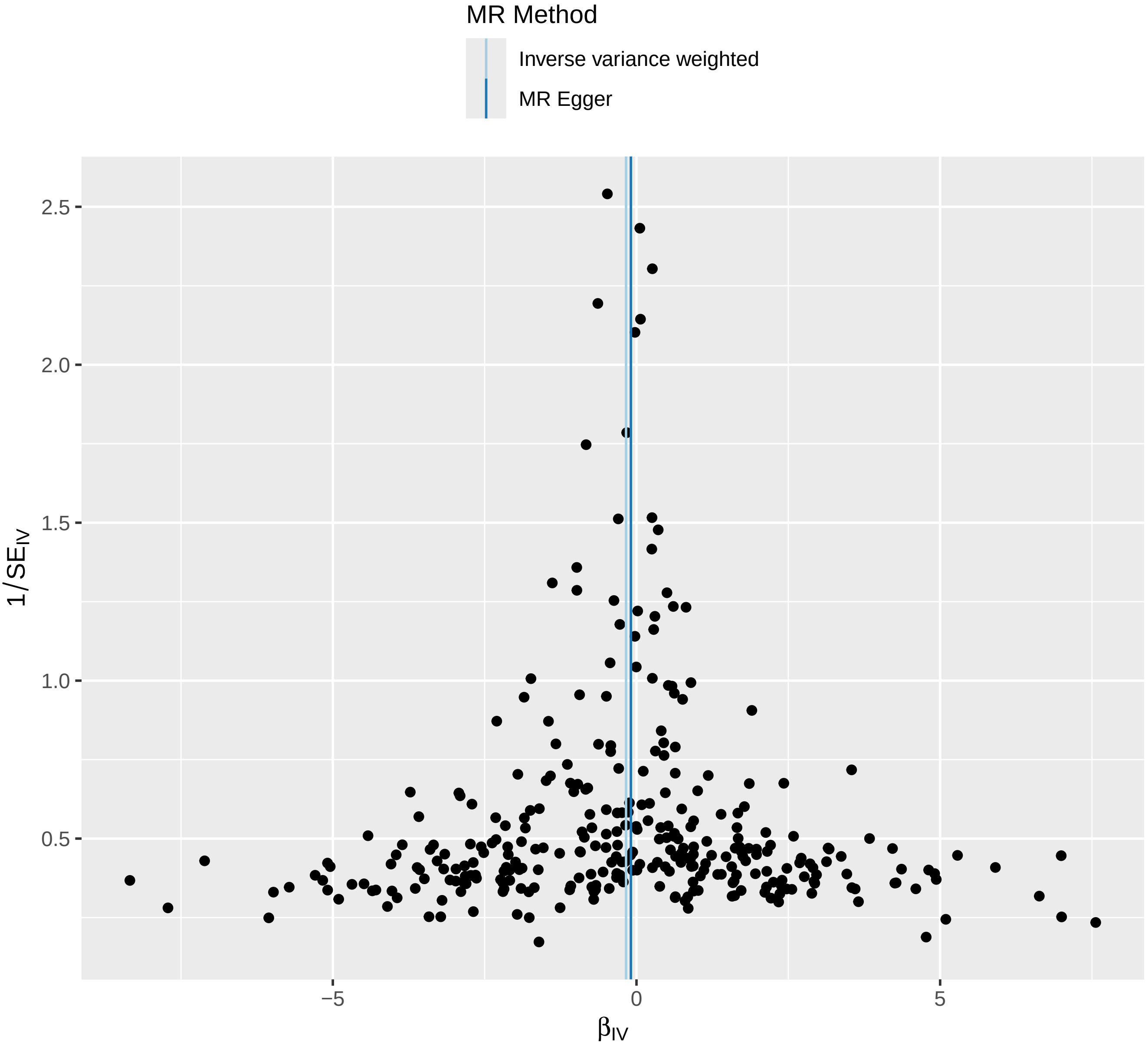

MR Method
Inverse variance weighted
MR Egger
2.5
2.0
1.5
1.0
0.5
1/SE_IV
−5
0
5
β_IV

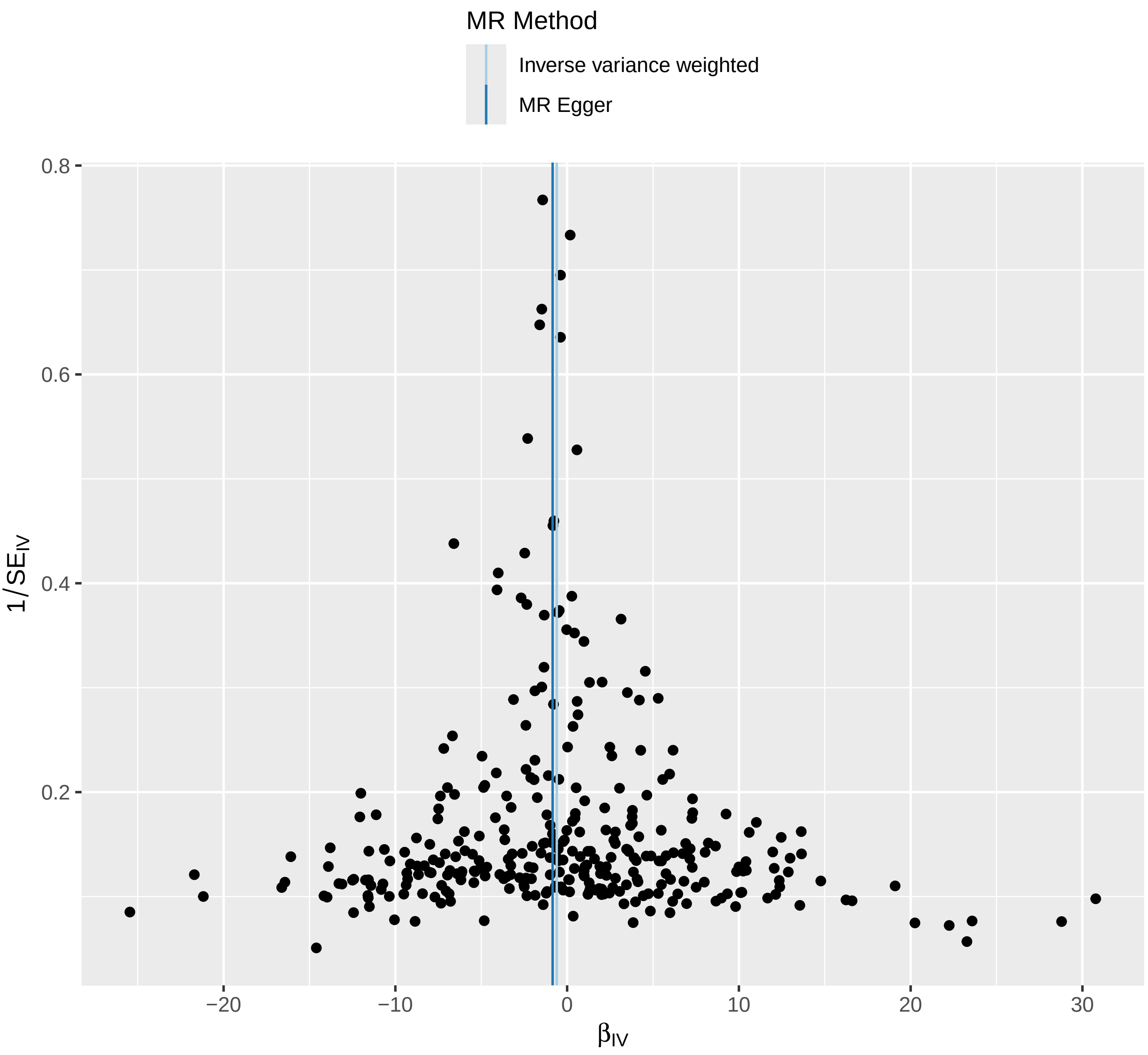
MR Method
Inverse variance weighted
MR Egger
0.8
0.6
0.4
0.2
1/SE_IV
−20
−10
0
10
20
30
β_IV

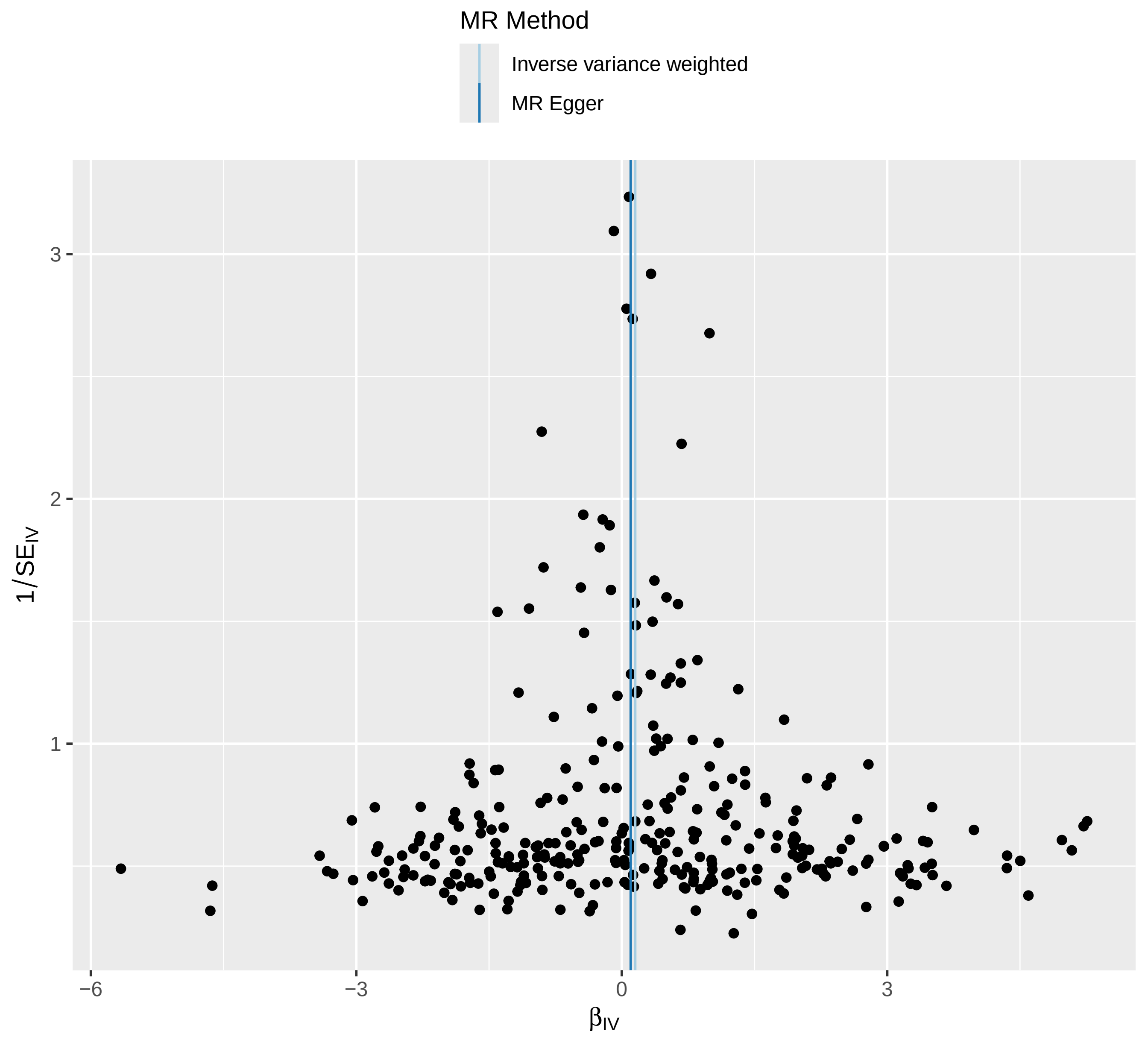
MR Method
Inverse variance weighted
MR Egger
1/SE_IV
3
2
1
β_IV
−6
−3
0
3

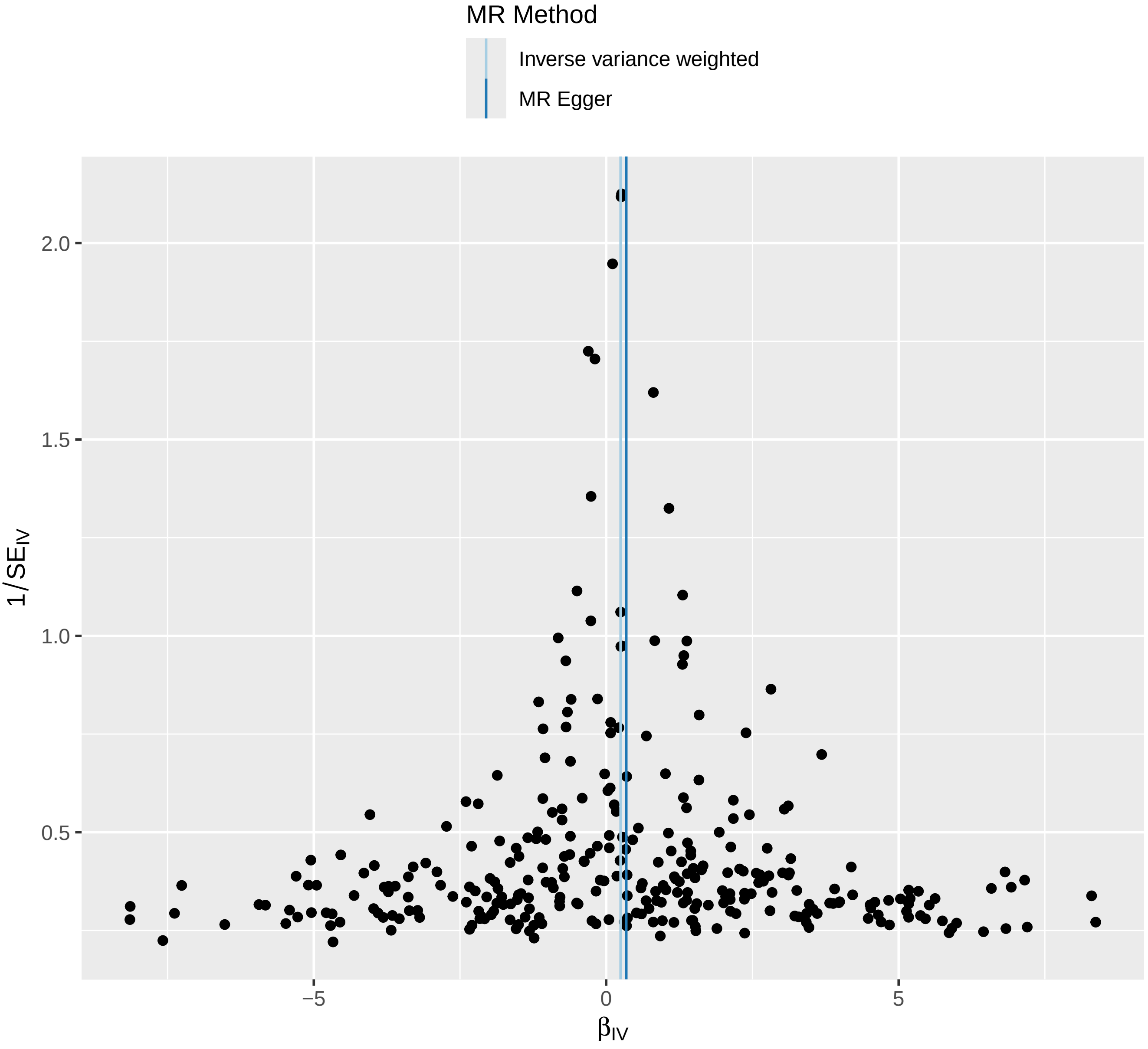
MR Method
Inverse variance weighted
MR Egger
$1/SE_{IV}$
2.0
1.5
1.0
0.5
−5
0
5
β_{IV}

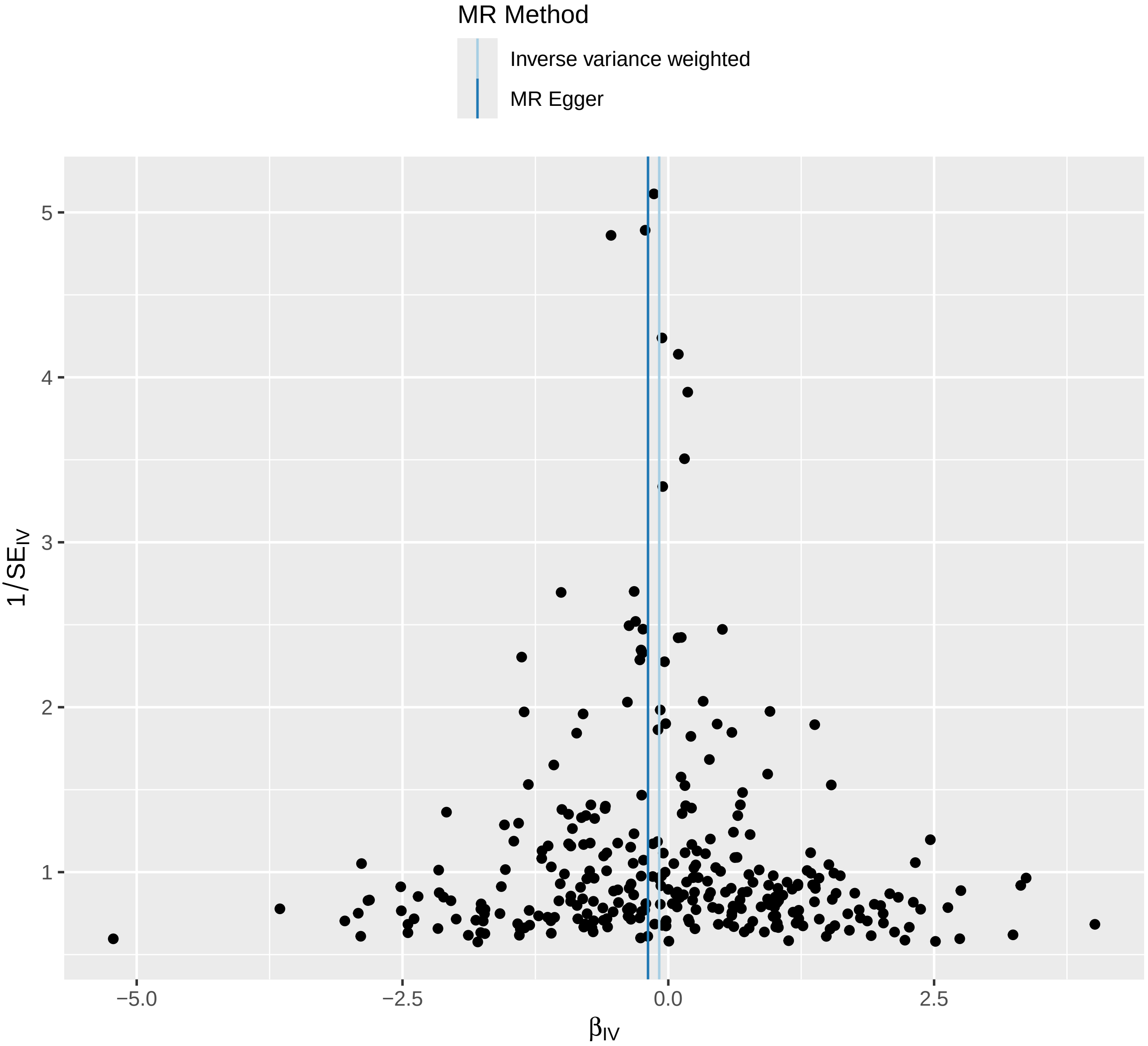
MR Method
Inverse variance weighted
MR Egger
5
4
3
2
1
1/SE_IV
−5.0
−2.5
0.0
2.5
β_IV

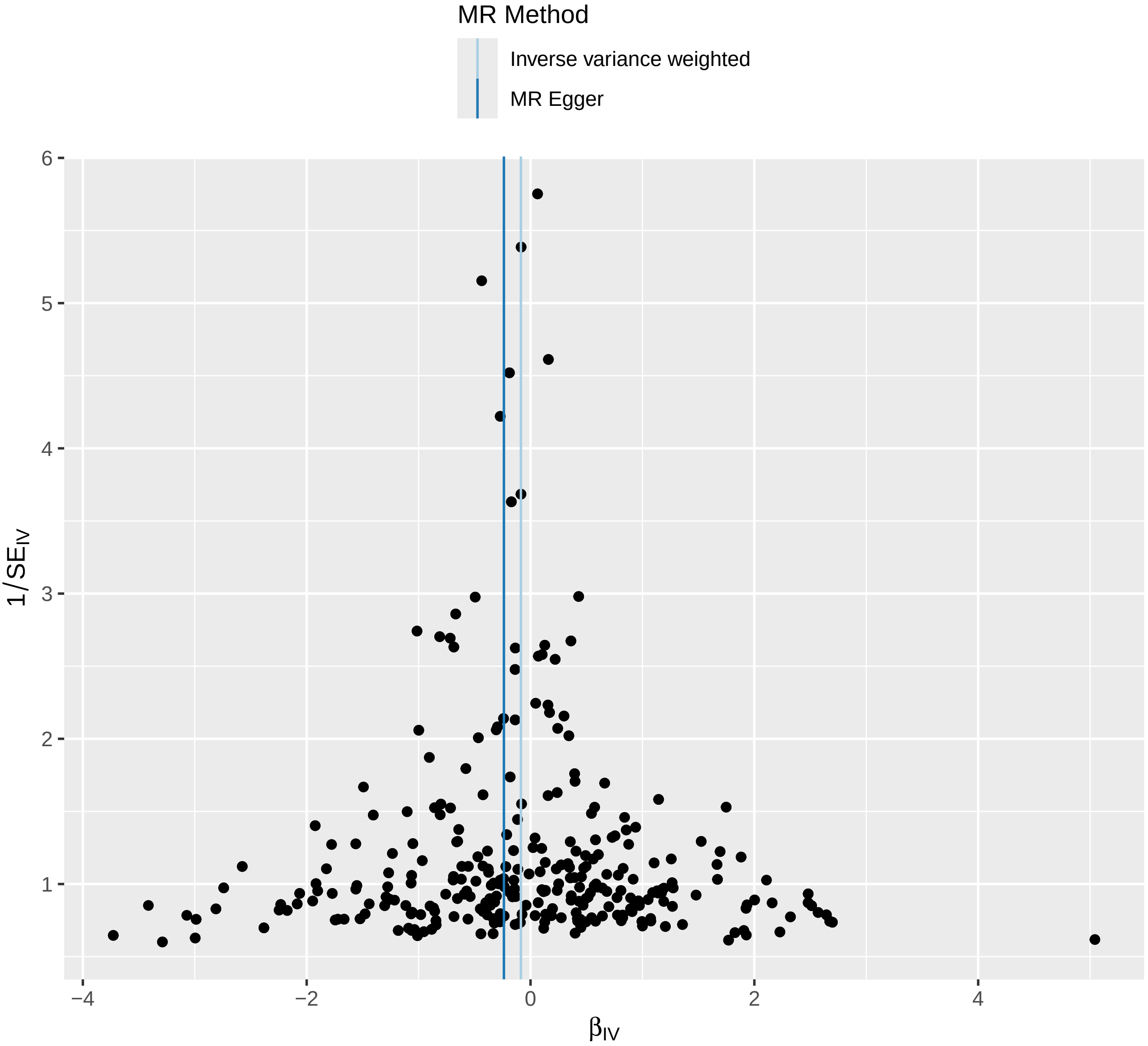

MR Method
Inverse variance weighted
MR Egger
6
5
4
3
2
1
1/SE_IV
−4
−2
0
2
4
β_IV
