# Supplementary figures and images for: Fatty acid traits mediate the effects of uric acid on cancers: a Mendelian randomization study
Source: Front Genet. 2024 Dec 2;15:1449205. doi: 10.3389/fgene.2024.1449205 (PMC11646984; doi:10.3389/fgene.2024.1449205)

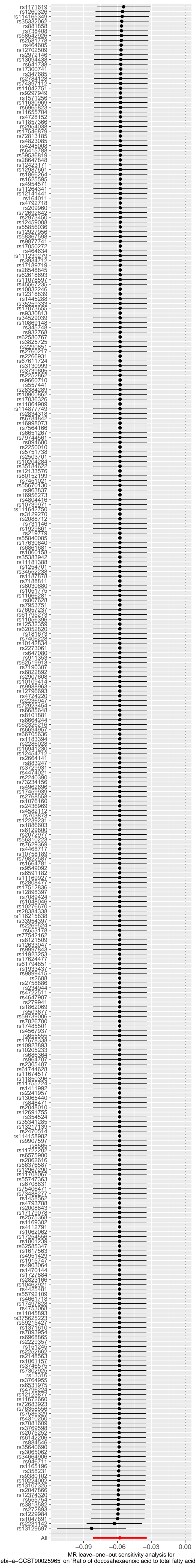

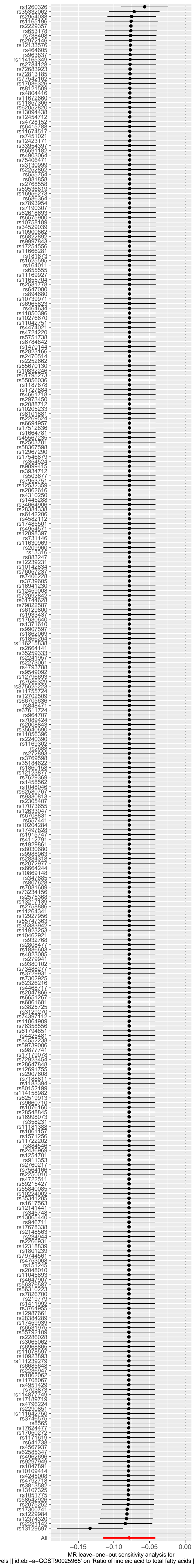

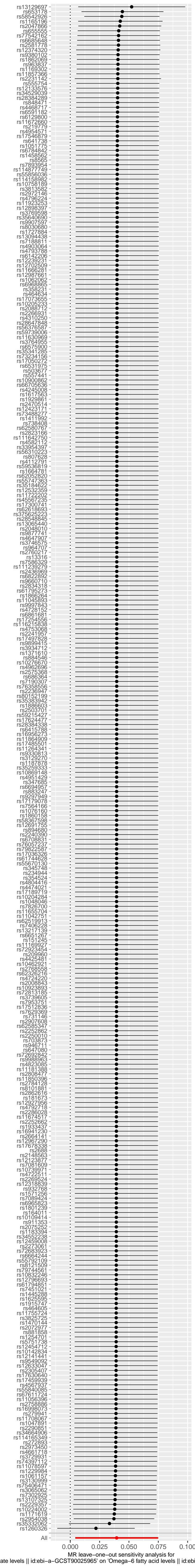

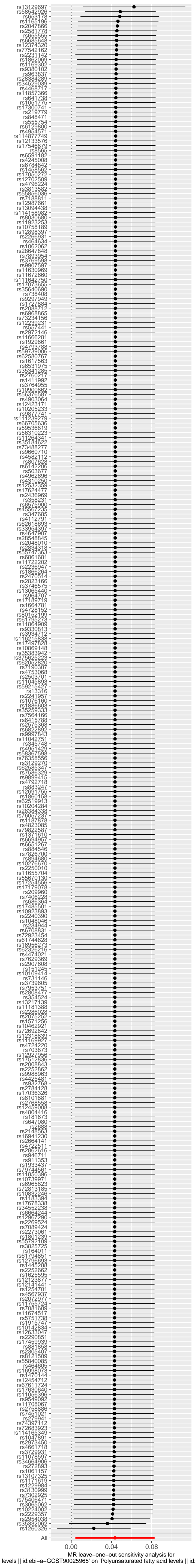

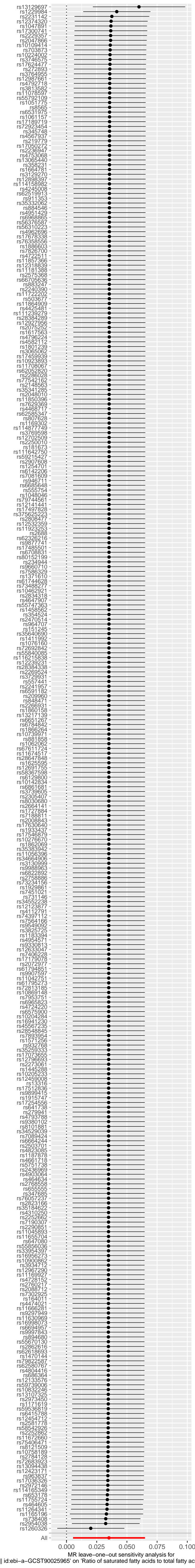

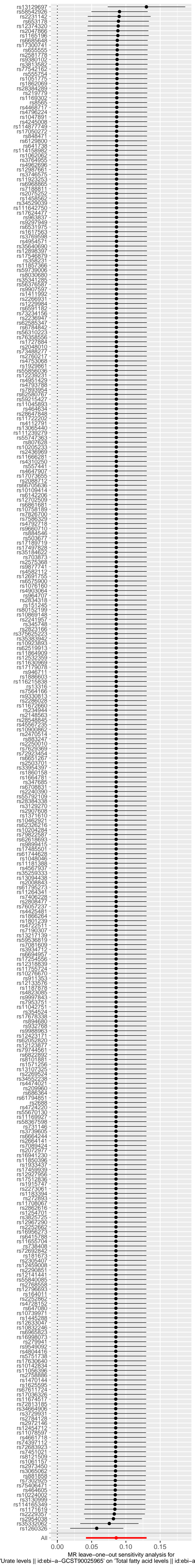

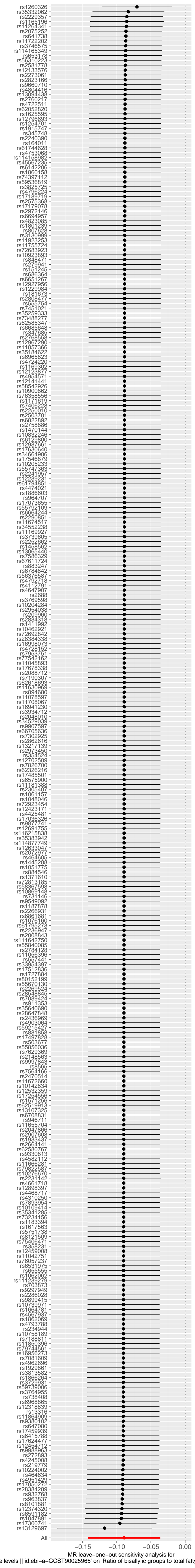

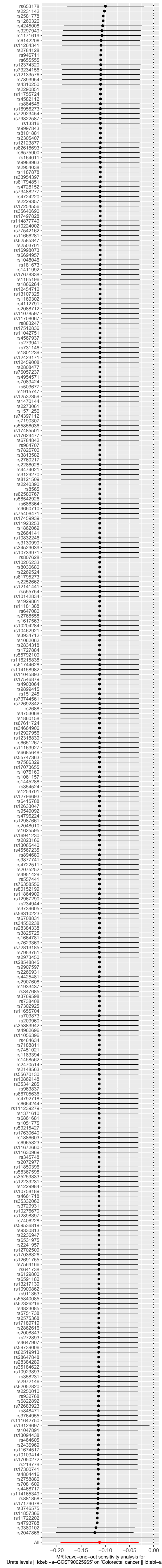

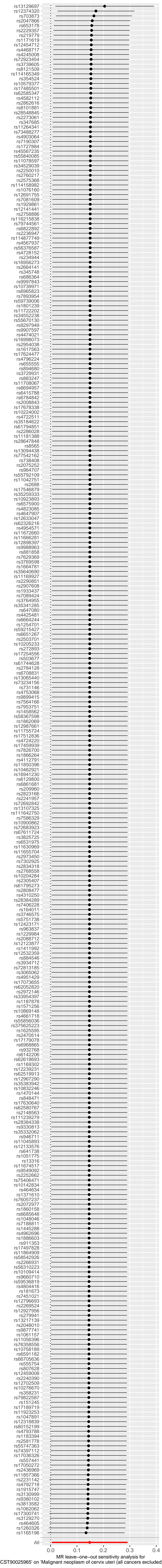

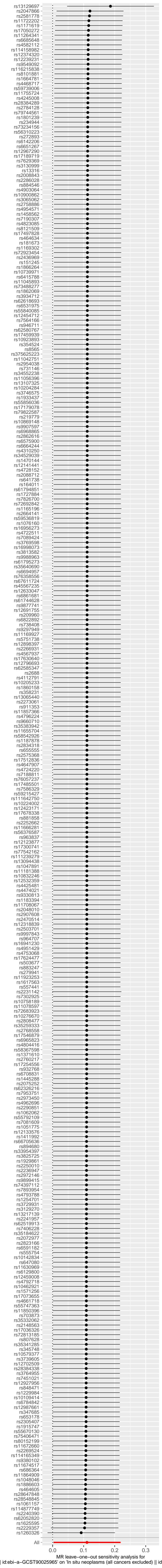

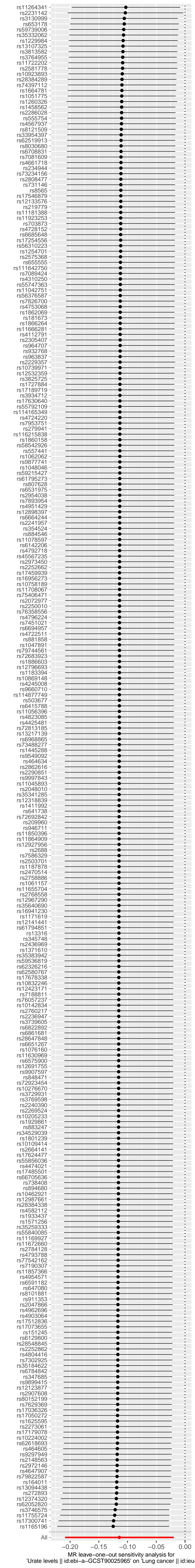

Supplement: Supplementary file 2 [file Image3.pdf]
