# Supplementary material for: Fatty acid traits mediate the effects of uric acid on cancers: a Mendelian randomization study
Source: Front Genet. 2024 Dec 2;15:1449205. doi: 10.3389/fgene.2024.1449205 (PMC11646984; doi:10.3389/fgene.2024.1449205)
Supplement: Supplementary file 3 [file DataSheet1.docx]

1. UA to cancers

library(TwoSampleMR)

library(ggplot2)

expo_rt<-extract_instruments(outcome="ebi-a-GCST90025965",clump = FALSE)

expo_rt<-clump_data(expo_rt,clump_kb = 10000,clump_r2 = 0.001,clump_p1 = 5e-08,clump_p2 = 5e-08,)

outc_rt <- extract_outcome_data(expo_rt$SNP, outcomes = c("finn-b-C3_EYE_ADNEXA_EXALLC","finn-b-C3_EYE_BRAIN_NEURO_EXALLC","finn-b-C3_BRONCHUS_LUNG_EXALLC","ebi-a-GCST012879","finn-b-CD2_NONHODGKIN_NAS_EXALLC","ieu-a-985","finn-b-C3_RESPIRATORY_INTRATHORACIC_EXALLC","ieu-a-1135","ieu-a-1123","finn-b-C3_SCLC_EXALLC","finn-b-C3_SCLC","finn-b-C3_CERVIX_UTERI_EXALLC","finn-b-CD2_INSITU_EXALLC","finn-b-CD2_INSITU_CERVIX_UTERI_EXALLC","finn-b-C3_HEART_MEDIASTINUM_PLEURA_EXALLC"))

dat<- harmonise_data(exposure_dat = expo_rt, outcome_dat = outc_rt)

outTab=dat[dat$mr_keep=="TRUE",]

write.csv(outTab, file="table.SNP.csv", row.names=F)

mrResult=mr(dat)

mrTab=generate_odds_ratios(dat)

write.csv(mrTab, file="table.MRresult.csv", row.names=F)

heterTab=mr_heterogeneity(dat)

write.csv(heterTab, file="table.heterogeneity.csv", row.names=F)

pleioTab=mr_pleiotropy_test(dat)

write.csv(pleioTab, file="table.pleiotropy.csv", row.names=F)

pdf(file="pic.scatter_plot.pdf", width=7.5, height=7)

mr_scatter_plot(mrResult, dat)

dev.off()

res_single=mr_singlesnp(dat)

pdf(file="pic.forest.pdf", width=7, height=5.5)

mr_forest_plot(res_single)

dev.off()

pdf(file="pic.funnel_plot.pdf", width=7, height=6.5)

mr_funnel_plot(singlesnp_results = res_single)

dev.off()

pdf(file="pic.leaveoneout.pdf", width=7, height=5.5)

mr_leaveoneout_plot(leaveoneout_results = mr_leaveoneout(dat))

dev.off()

1. UA to fatty acid

library(TwoSampleMR)

library(ggplot2)

expo_rt<-extract_instruments(outcome="ebi-a-GCST90025965",clump = FALSE)

expo_rt<-clump_data(expo_rt,clump_kb = 10000,clump_r2 = 0.001,clump_p1 = 5e-08,clump_p2 = 5e-08,)

outc_rt <- extract_outcome_data(expo_rt$SNP, outcomes = c("ebi-a-GCST90092929","ebi-a-GCST90092940","ebi-a-GCST90092941","ebi-a-GCST90092928","ebi-a-GCST90092817","ebi-a-GCST90092935","ebi-a-GCST90092881","ebi-a-GCST90092980","ebi-a-GCST90092987","met-c-845","ebi-a-GCST90092981","ebi-a-GCST90092933","ebi-a-GCST90092939","ebi-a-GCST90092880"))

dat<- harmonise_data(exposure_dat = expo_rt, outcome_dat = outc_rt)

outTab=dat[dat$mr_keep=="TRUE",]

write.csv(outTab, file="table.SNP.csv", row.names=F)

mrResult=mr(dat)

mrTab=generate_odds_ratios(dat)

write.csv(mrTab, file="table.MRresult.csv", row.names=F)

heterTab=mr_heterogeneity(dat)

write.csv(heterTab, file="table.heterogeneity.csv", row.names=F)

pleioTab=mr_pleiotropy_test(dat)

write.csv(pleioTab, file="table.pleiotropy.csv", row.names=F)

pdf(file="pic.scatter_plot.pdf", width=7.5, height=7)

mr_scatter_plot(mrResult, dat)

dev.off()

res_single=mr_singlesnp(dat)

pdf(file="pic.forest.pdf", width=7, height=5.5)

mr_forest_plot(res_single)

dev.off()

pdf(file="pic.funnel_plot.pdf", width=7, height=6.5)

mr_funnel_plot(singlesnp_results = res_single)

dev.off()

pdf(file="pic.leaveoneout.pdf", width=7, height=5.5)

mr_leaveoneout_plot(leaveoneout_results = mr_leaveoneout(dat))

dev.off()

1. fatty acid to cancers

library(TwoSampleMR)

library(ggplot2)

expo_rt<-extract_instruments(outcome="ebi-a-GCST90092817",clump = FALSE)

expo_rt<-clump_data(expo_rt,clump_kb = 10000,clump_r2 = 0.001,clump_p1 = 5e-08,clump_p2 = 5e-08,)

outc_rt <- extract_outcome_data(expo_rt$SNP, outcomes = c("ebi-a-GCST012879","ieu-a-1135","ieu-a-985"))

dat<- harmonise_data(exposure_dat = expo_rt, outcome_dat = outc_rt)

outTab=dat[dat$mr_keep=="TRUE",]

write.csv(outTab, file="table.SNP.csv", row.names=F)

mrResult=mr(dat)

mrTab=generate_odds_ratios(dat)

write.csv(mrTab, file="table.MRresult.csv", row.names=F)

heterTab=mr_heterogeneity(dat)

write.csv(heterTab, file="table.heterogeneity.csv", row.names=F)

pleioTab=mr_pleiotropy_test(dat)

write.csv(pleioTab, file="table.pleiotropy.csv", row.names=F)

pdf(file="pic.scatter_plot.pdf", width=7.5, height=7)

mr_scatter_plot(mrResult, dat)

dev.off()

res_single=mr_singlesnp(dat)

pdf(file="pic.forest.pdf", width=7, height=5.5)

mr_forest_plot(res_single)

dev.off()

pdf(file="pic.funnel_plot.pdf", width=7, height=6.5)

mr_funnel_plot(singlesnp_results = res_single)

dev.off()

pdf(file="pic.leaveoneout.pdf", width=7, height=5.5)

mr_leaveoneout_plot(leaveoneout_results = mr_leaveoneout(dat))

dev.off()

expo_rt<-extract_instruments(outcome="ebi-a-GCST90092881",clump = FALSE)

expo_rt<-clump_data(expo_rt,clump_kb = 10000,clump_r2 = 0.001,clump_p1 = 5e-08,clump_p2 = 5e-08,)

outc_rt <- extract_outcome_data(expo_rt$SNP, outcomes = c("ebi-a-GCST012879","finn-b-CD2_INSITU_EXALLC","ieu-a-985"))

dat<- harmonise_data(exposure_dat = expo_rt, outcome_dat = outc_rt)

outTab=dat[dat$mr_keep=="TRUE",]

write.csv(outTab, file="table.SNP.csv", row.names=F)

mrResult=mr(dat)

mrTab=generate_odds_ratios(dat)

write.csv(mrTab, file="table.MRresult.csv", row.names=F)

heterTab=mr_heterogeneity(dat)

write.csv(heterTab, file="table.heterogeneity.csv", row.names=F)

pleioTab=mr_pleiotropy_test(dat)

write.csv(pleioTab, file="table.pleiotropy.csv", row.names=F)

pdf(file="pic.scatter_plot.pdf", width=7.5, height=7)

mr_scatter_plot(mrResult, dat)

dev.off()

res_single=mr_singlesnp(dat)

pdf(file="pic.forest.pdf", width=7, height=5.5)

mr_forest_plot(res_single)

dev.off()

pdf(file="pic.funnel_plot.pdf", width=7, height=6.5)

mr_funnel_plot(singlesnp_results = res_single)

dev.off()

pdf(file="pic.leaveoneout.pdf", width=7, height=5.5)

mr_leaveoneout_plot(leaveoneout_results = mr_leaveoneout(dat))

dev.off()

expo_rt<-extract_instruments(outcome="ebi-a-GCST90092929",clump = FALSE)

expo_rt<-clump_data(expo_rt,clump_kb = 10000,clump_r2 = 0.001,clump_p1 = 5e-08,clump_p2 = 5e-08,)

outc_rt <- extract_outcome_data(expo_rt$SNP, outcomes ="ieu-a-1135")

dat<- harmonise_data(exposure_dat = expo_rt, outcome_dat = outc_rt)

outTab=dat[dat$mr_keep=="TRUE",]

write.csv(outTab, file="table.SNP.csv", row.names=F)

mrResult=mr(dat)

mrTab=generate_odds_ratios(dat)

write.csv(mrTab, file="table.MRresult.csv", row.names=F)

heterTab=mr_heterogeneity(dat)

write.csv(heterTab, file="table.heterogeneity.csv", row.names=F)

pleioTab=mr_pleiotropy_test(dat)

write.csv(pleioTab, file="table.pleiotropy.csv", row.names=F)

pdf(file="pic.scatter_plot.pdf", width=7.5, height=7)

mr_scatter_plot(mrResult, dat)

dev.off()

res_single=mr_singlesnp(dat)

pdf(file="pic.forest.pdf", width=7, height=5.5)

mr_forest_plot(res_single)

dev.off()

pdf(file="pic.funnel_plot.pdf", width=7, height=6.5)

mr_funnel_plot(singlesnp_results = res_single)

dev.off()

pdf(file="pic.leaveoneout.pdf", width=7, height=5.5)

mr_leaveoneout_plot(leaveoneout_results = mr_leaveoneout(dat))

dev.off()

expo_rt<-extract_instruments(outcome="ebi-a-GCST90092933",clump = FALSE)

expo_rt<-clump_data(expo_rt,clump_kb = 10000,clump_r2 = 0.001,clump_p1 = 5e-08,clump_p2 = 5e-08,)

outc_rt <- extract_outcome_data(expo_rt$SNP, outcomes ="finn-b-C3_CERVIX_UTERI_EXALLC")

dat<- harmonise_data(exposure_dat = expo_rt, outcome_dat = outc_rt)

outTab=dat[dat$mr_keep=="TRUE",]

write.csv(outTab, file="table.SNP.csv", row.names=F)

mrResult=mr(dat)

mrTab=generate_odds_ratios(dat)

write.csv(mrTab, file="table.MRresult.csv", row.names=F)

heterTab=mr_heterogeneity(dat)

write.csv(heterTab, file="table.heterogeneity.csv", row.names=F)

pleioTab=mr_pleiotropy_test(dat)

write.csv(pleioTab, file="table.pleiotropy.csv", row.names=F)

pdf(file="pic.scatter_plot.pdf", width=7.5, height=7)

mr_scatter_plot(mrResult, dat)

dev.off()

res_single=mr_singlesnp(dat)

pdf(file="pic.forest.pdf", width=7, height=5.5)

mr_forest_plot(res_single)

dev.off()

pdf(file="pic.funnel_plot.pdf", width=7, height=6.5)

mr_funnel_plot(singlesnp_results = res_single)

dev.off()

pdf(file="pic.leaveoneout.pdf", width=7, height=5.5)

mr_leaveoneout_plot(leaveoneout_results = mr_leaveoneout(dat))

dev.off()

expo_rt<-extract_instruments(outcome="ebi-a-GCST90092935",clump = FALSE)

expo_rt<-clump_data(expo_rt,clump_kb = 10000,clump_r2 = 0.001,clump_p1 = 5e-08,clump_p2 = 5e-08,)

outc_rt <- extract_outcome_data(expo_rt$SNP, outcomes ="ieu-a-985")

dat<- harmonise_data(exposure_dat = expo_rt, outcome_dat = outc_rt)

outTab=dat[dat$mr_keep=="TRUE",]

write.csv(outTab, file="table.SNP.csv", row.names=F)

mrResult=mr(dat)

mrTab=generate_odds_ratios(dat)

write.csv(mrTab, file="table.MRresult.csv", row.names=F)

heterTab=mr_heterogeneity(dat)

write.csv(heterTab, file="table.heterogeneity.csv", row.names=F)

pleioTab=mr_pleiotropy_test(dat)

write.csv(pleioTab, file="table.pleiotropy.csv", row.names=F)

pdf(file="pic.scatter_plot.pdf", width=7.5, height=7)

mr_scatter_plot(mrResult, dat)

dev.off()

res_single=mr_singlesnp(dat)

pdf(file="pic.forest.pdf", width=7, height=5.5)

mr_forest_plot(res_single)

dev.off()

pdf(file="pic.funnel_plot.pdf", width=7, height=6.5)

mr_funnel_plot(singlesnp_results = res_single)

dev.off()

pdf(file="pic.leaveoneout.pdf", width=7, height=5.5)

mr_leaveoneout_plot(leaveoneout_results = mr_leaveoneout(dat))

dev.off()

expo_rt<-extract_instruments(outcome="ebi-a-GCST90092939",clump = FALSE)

expo_rt<-clump_data(expo_rt,clump_kb = 10000,clump_r2 = 0.001,clump_p1 = 5e-08,clump_p2 = 5e-08,)

outc_rt <- extract_outcome_data(expo_rt$SNP, outcomes ="finn-b-C3_CERVIX_UTERI_EXALLC")

dat<- harmonise_data(exposure_dat = expo_rt, outcome_dat = outc_rt)

outTab=dat[dat$mr_keep=="TRUE",]

write.csv(outTab, file="table.SNP.csv", row.names=F)

mrResult=mr(dat)

mrTab=generate_odds_ratios(dat)

write.csv(mrTab, file="table.MRresult.csv", row.names=F)

heterTab=mr_heterogeneity(dat)

write.csv(heterTab, file="table.heterogeneity.csv", row.names=F)

pleioTab=mr_pleiotropy_test(dat)

write.csv(pleioTab, file="table.pleiotropy.csv", row.names=F)

pdf(file="pic.scatter_plot.pdf", width=7.5, height=7)

mr_scatter_plot(mrResult, dat)

dev.off()

res_single=mr_singlesnp(dat)

pdf(file="pic.forest.pdf", width=7, height=5.5)

mr_forest_plot(res_single)

dev.off()

pdf(file="pic.funnel_plot.pdf", width=7, height=6.5)

mr_funnel_plot(singlesnp_results = res_single)

dev.off()

pdf(file="pic.leaveoneout.pdf", width=7, height=5.5)

mr_leaveoneout_plot(leaveoneout_results = mr_leaveoneout(dat))

dev.off()

expo_rt<-extract_instruments(outcome="ebi-a-GCST90092940",clump = FALSE)

expo_rt<-clump_data(expo_rt,clump_kb = 10000,clump_r2 = 0.001,clump_p1 = 5e-08,clump_p2 = 5e-08,)

outc_rt <- extract_outcome_data(expo_rt$SNP, outcomes ="ieu-a-1135")

dat<- harmonise_data(exposure_dat = expo_rt, outcome_dat = outc_rt)

outTab=dat[dat$mr_keep=="TRUE",]

write.csv(outTab, file="table.SNP.csv", row.names=F)

mrResult=mr(dat)

mrTab=generate_odds_ratios(dat)

write.csv(mrTab, file="table.MRresult.csv", row.names=F)

heterTab=mr_heterogeneity(dat)

write.csv(heterTab, file="table.heterogeneity.csv", row.names=F)

pleioTab=mr_pleiotropy_test(dat)

write.csv(pleioTab, file="table.pleiotropy.csv", row.names=F)

pdf(file="pic.scatter_plot.pdf", width=7.5, height=7)

mr_scatter_plot(mrResult, dat)

dev.off()

res_single=mr_singlesnp(dat)

pdf(file="pic.forest.pdf", width=7, height=5.5)

mr_forest_plot(res_single)

dev.off()

pdf(file="pic.funnel_plot.pdf", width=7, height=6.5)

mr_funnel_plot(singlesnp_results = res_single)

dev.off()

pdf(file="pic.leaveoneout.pdf", width=7, height=5.5)

mr_leaveoneout_plot(leaveoneout_results = mr_leaveoneout(dat))

dev.off()

expo_rt<-extract_instruments(outcome="ebi-a-GCST90092981",clump = FALSE)

expo_rt<-clump_data(expo_rt,clump_kb = 10000,clump_r2 = 0.001,clump_p1 = 5e-08,clump_p2 = 5e-08,)

outc_rt <- extract_outcome_data(expo_rt$SNP, outcomes ="ieu-a-985")

dat<- harmonise_data(exposure_dat = expo_rt, outcome_dat = outc_rt)

outTab=dat[dat$mr_keep=="TRUE",]

write.csv(outTab, file="table.SNP.csv", row.names=F)

mrResult=mr(dat)

mrTab=generate_odds_ratios(dat)

write.csv(mrTab, file="table.MRresult.csv", row.names=F)

heterTab=mr_heterogeneity(dat)

write.csv(heterTab, file="table.heterogeneity.csv", row.names=F)

pleioTab=mr_pleiotropy_test(dat)

write.csv(pleioTab, file="table.pleiotropy.csv", row.names=F)

pdf(file="pic.scatter_plot.pdf", width=7.5, height=7)

mr_scatter_plot(mrResult, dat)

dev.off()

res_single=mr_singlesnp(dat)

pdf(file="pic.forest.pdf", width=7, height=5.5)

mr_forest_plot(res_single)

dev.off()

pdf(file="pic.funnel_plot.pdf", width=7, height=6.5)

mr_funnel_plot(singlesnp_results = res_single)

dev.off()

pdf(file="pic.leaveoneout.pdf", width=7, height=5.5)

mr_leaveoneout_plot(leaveoneout_results = mr_leaveoneout(dat))

dev.off()

expo_rt<-extract_instruments(outcome="ebi-a-GCST90092981",clump = FALSE)

expo_rt<-clump_data(expo_rt,clump_kb = 10000,clump_r2 = 0.001,clump_p1 = 5e-08,clump_p2 = 5e-08,)

outc_rt <- extract_outcome_data(expo_rt$SNP, outcomes ="finn-b-C3_CERVIX_UTERI_EXALLC")

dat<- harmonise_data(exposure_dat = expo_rt, outcome_dat = outc_rt)

outTab=dat[dat$mr_keep=="TRUE",]

write.csv(outTab, file="table.SNP.csv", row.names=F)

mrResult=mr(dat)

mrTab=generate_odds_ratios(dat)

write.csv(mrTab, file="table.MRresult.csv", row.names=F)

heterTab=mr_heterogeneity(dat)

write.csv(heterTab, file="table.heterogeneity.csv", row.names=F)

pleioTab=mr_pleiotropy_test(dat)

write.csv(pleioTab, file="table.pleiotropy.csv", row.names=F)

pdf(file="pic.scatter_plot.pdf", width=7.5, height=7)

mr_scatter_plot(mrResult, dat)

dev.off()

res_single=mr_singlesnp(dat)

pdf(file="pic.forest.pdf", width=7, height=5.5)

mr_forest_plot(res_single)

dev.off()

pdf(file="pic.funnel_plot.pdf", width=7, height=6.5)

mr_funnel_plot(singlesnp_results = res_single)

dev.off()

pdf(file="pic.leaveoneout.pdf", width=7, height=5.5)

mr_leaveoneout_plot(leaveoneout_results = mr_leaveoneout(dat))

dev.off()

expo_rt<-extract_instruments(outcome="ebi-a-GCST90092987",clump = FALSE)

expo_rt<-clump_data(expo_rt,clump_kb = 10000,clump_r2 = 0.001,clump_p1 = 5e-08,clump_p2 = 5e-08,)

outc_rt <- extract_outcome_data(expo_rt$SNP, outcomes ="finn-b-C3_CERVIX_UTERI_EXALLC")

dat<- harmonise_data(exposure_dat = expo_rt, outcome_dat = outc_rt)

outTab=dat[dat$mr_keep=="TRUE",]

write.csv(outTab, file="table.SNP.csv", row.names=F)

mrResult=mr(dat)

mrTab=generate_odds_ratios(dat)

write.csv(mrTab, file="table.MRresult.csv", row.names=F)

heterTab=mr_heterogeneity(dat)

write.csv(heterTab, file="table.heterogeneity.csv", row.names=F)

pleioTab=mr_pleiotropy_test(dat)

write.csv(pleioTab, file="table.pleiotropy.csv", row.names=F)

pdf(file="pic.scatter_plot.pdf", width=7.5, height=7)

mr_scatter_plot(mrResult, dat)

dev.off()

res_single=mr_singlesnp(dat)

pdf(file="pic.forest.pdf", width=7, height=5.5)

mr_forest_plot(res_single)

dev.off()

pdf(file="pic.funnel_plot.pdf", width=7, height=6.5)

mr_funnel_plot(singlesnp_results = res_single)

dev.off()

pdf(file="pic.leaveoneout.pdf", width=7, height=5.5)

mr_leaveoneout_plot(leaveoneout_results = mr_leaveoneout(dat))

dev.off()

expo_rt<-extract_instruments(outcome="met-c-845",clump = FALSE)

expo_rt<-clump_data(expo_rt,clump_kb = 10000,clump_r2 = 0.001,clump_p1 = 5e-08,clump_p2 = 5e-08,)

outc_rt <- extract_outcome_data(expo_rt$SNP, outcomes ="ebi-a-GCST012879")

dat<- harmonise_data(exposure_dat = expo_rt, outcome_dat = outc_rt)

outTab=dat[dat$mr_keep=="TRUE",]

write.csv(outTab, file="table.SNP.csv", row.names=F)

mrResult=mr(dat)

mrTab=generate_odds_ratios(dat)

write.csv(mrTab, file="table.MRresult.csv", row.names=F)

heterTab=mr_heterogeneity(dat)

write.csv(heterTab, file="table.heterogeneity.csv", row.names=F)

pleioTab=mr_pleiotropy_test(dat)

write.csv(pleioTab, file="table.pleiotropy.csv", row.names=F)

pdf(file="pic.scatter_plot.pdf", width=7.5, height=7)

mr_scatter_plot(mrResult, dat)

dev.off()

res_single=mr_singlesnp(dat)

pdf(file="pic.forest.pdf", width=7, height=5.5)

mr_forest_plot(res_single)

dev.off()

pdf(file="pic.funnel_plot.pdf", width=7, height=6.5)

mr_funnel_plot(singlesnp_results = res_single)

dev.off()

pdf(file="pic.leaveoneout.pdf", width=7, height=5.5)

mr_leaveoneout_plot(leaveoneout_results = mr_leaveoneout(dat))

dev.off()
