# Supplementary material for: Fatty acid traits mediate the effects of uric acid on cancers: a Mendelian randomization study
Source: Front Genet. 2024 Dec 2;15:1449205. doi: 10.3389/fgene.2024.1449205 (PMC11646984; doi:10.3389/fgene.2024.1449205)
Supplement: Supplementary file 4 [file Image1.pdf]

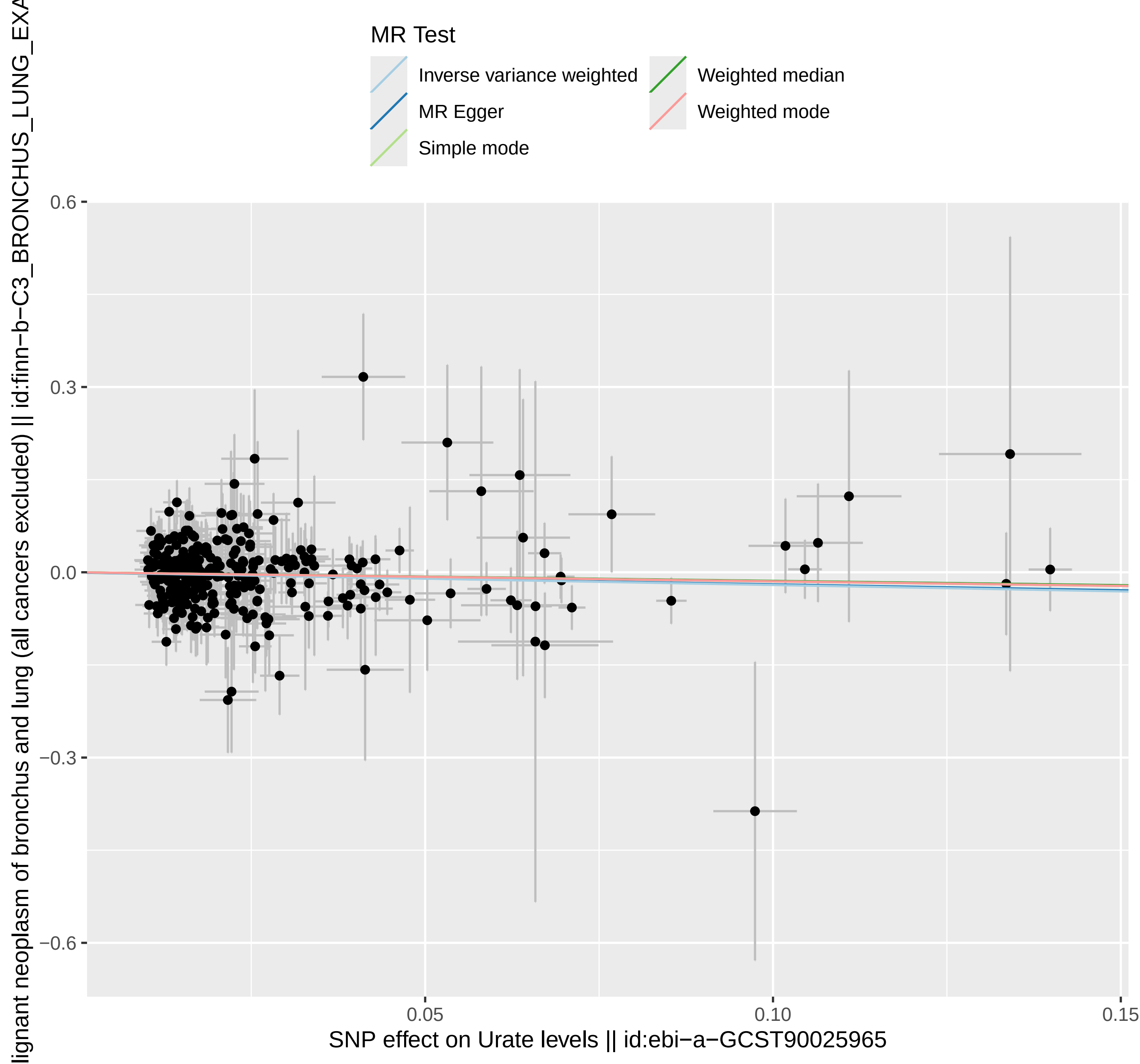
MR Test
Inverse variance weighted
MR Egger
Simple mode
Weighted median
Weighted mode
0.6
0.3
0.0
−0.3
−0.6
0.05
0.10
0.15
SNP effect on Urate levels || id:ebi−a−GCST90025965
lignant neoplasm of bronchus and lung (all cancers excluded) || id:finn−b−C3_BRONCHUS_LUNG_EXA

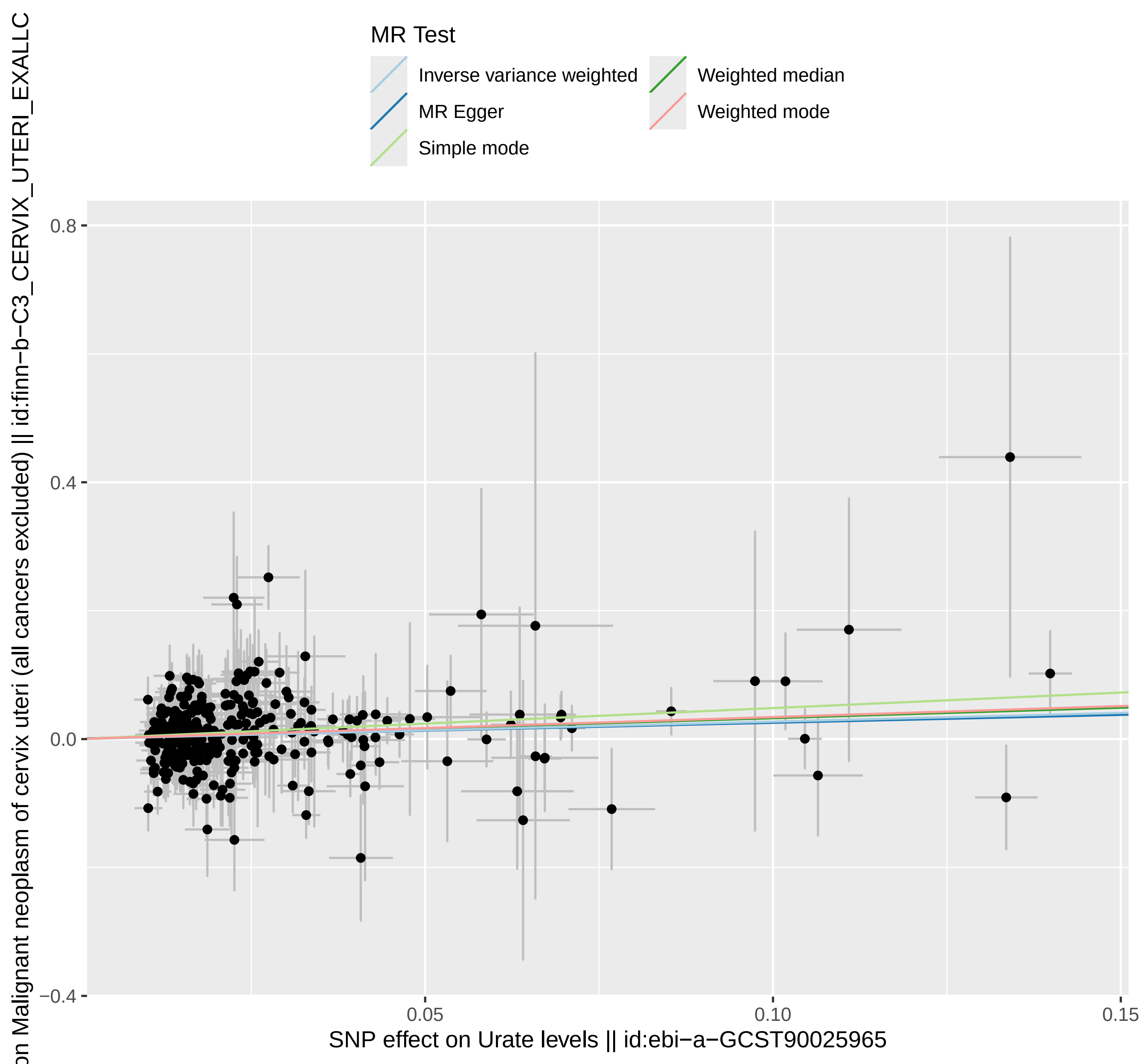

MR Test
Inverse variance weighted
MR Egger
Simple mode
Weighted median
Weighted mode
on Malignant neoplasm of cervix uteri (all cancers excluded) || id:finn-b-C3_CERVIX_UTERI_EXALLC
0.8
0.4
0.0
−0.4
0.05
0.10
0.15
SNP effect on Urate levels || id:ebi−a−GCST90025965

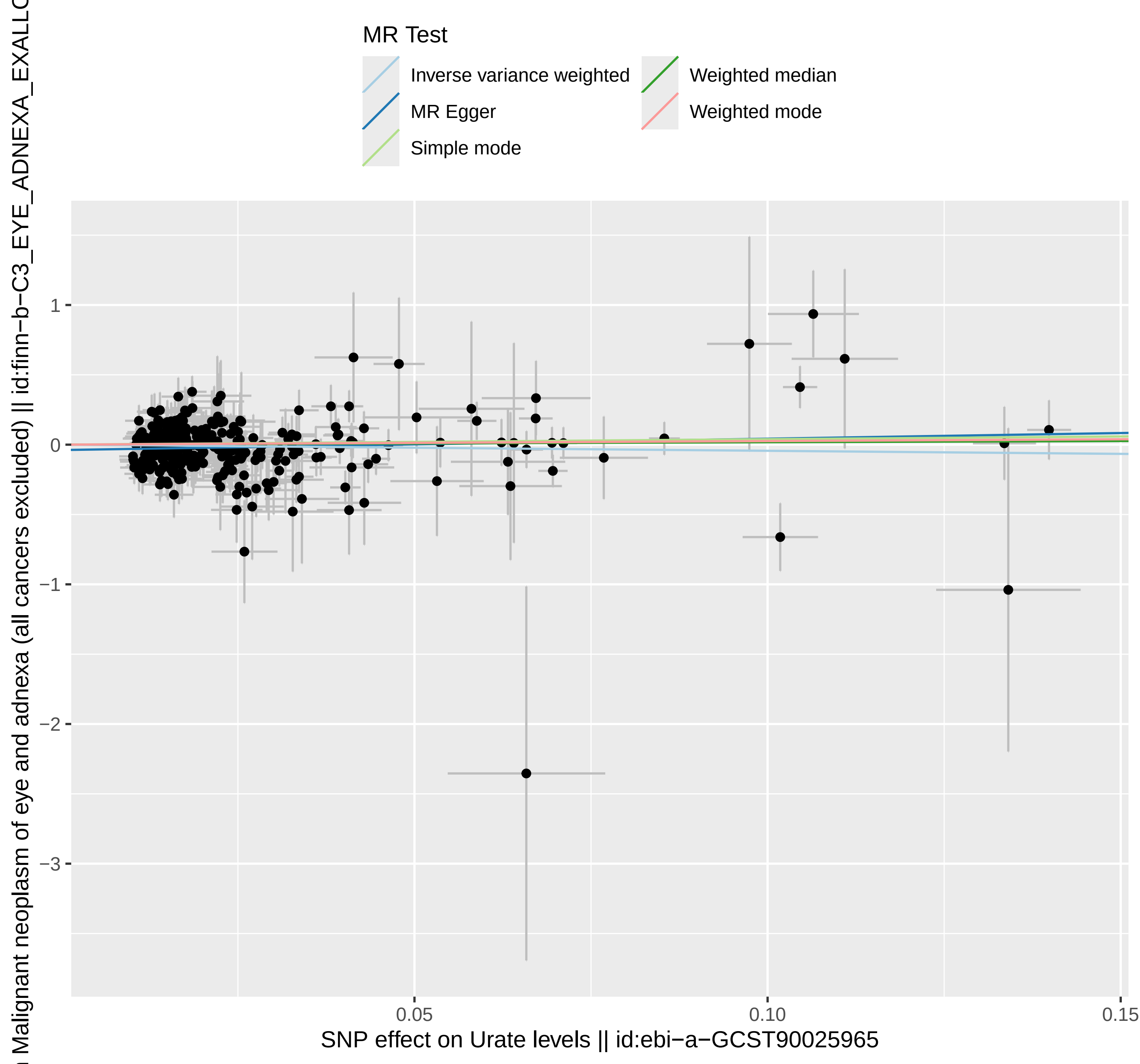

MR Test
Inverse variance weighted
MR Egger
Simple mode
Weighted median
Weighted mode
1
0
−1
−2
−3
0.05
0.10
0.15
SNP effect on Urate levels || id:ebi−a−GCST90025965
Malignant neoplasm of eye and adnexa (all cancers excluded) || id:finn−b−C3_EYE_ADNEXA_EXALLC

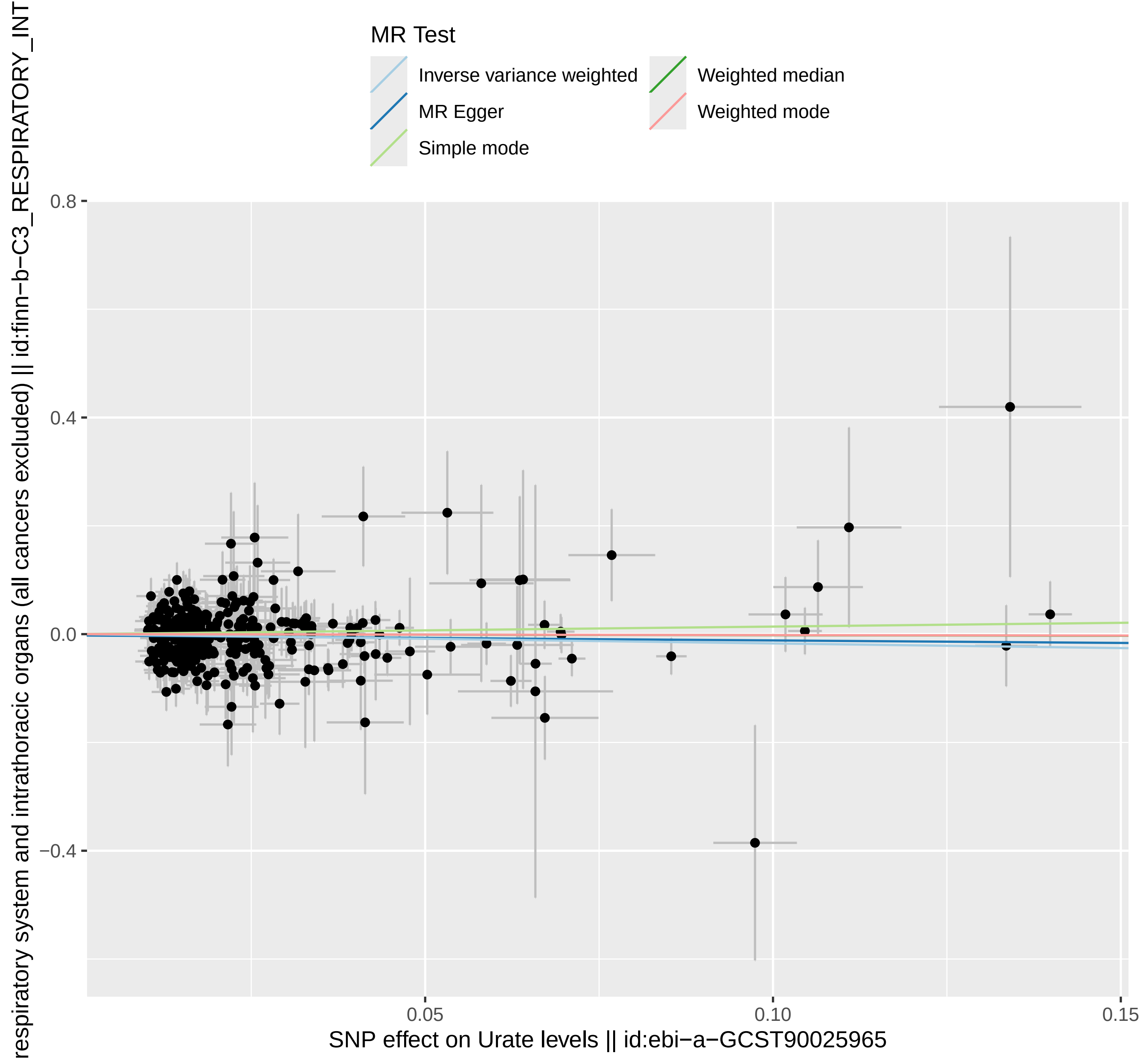
MR Test
Inverse variance weighted
MR Egger
Simple mode
Weighted median
Weighted mode
0.8
0.4
0.0
−0.4
0.05
0.10
0.15
SNP effect on Urate levels || id:ebi−a−GCST90025965
respiratory system and intrathoracic organs (all cancers excluded) || id:finn−b−C3_RESPIRATORY_INT

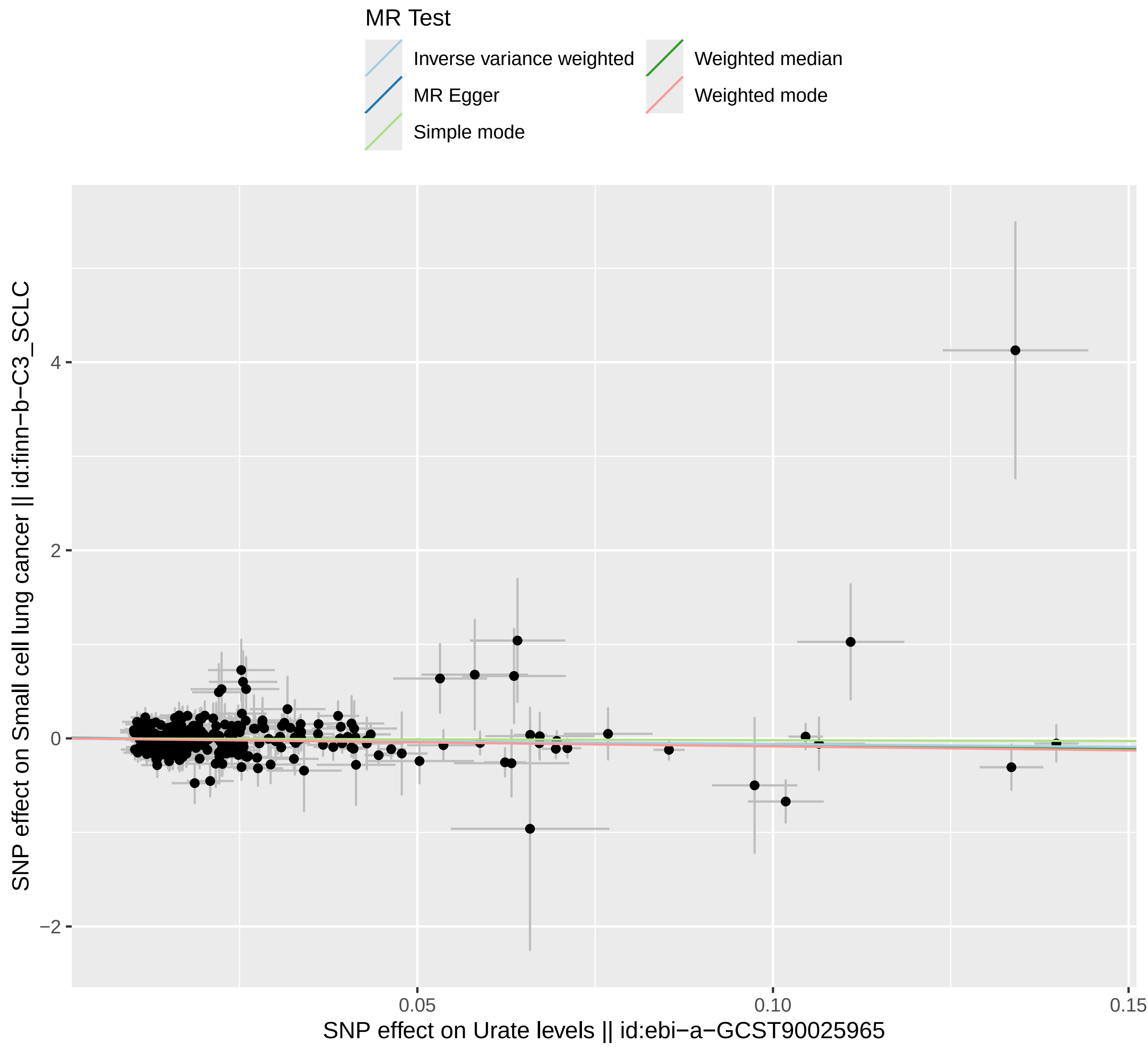
MR Test
Inverse variance weighted
MR Egger
Simple mode
Weighted median
Weighted mode
SNP effect on Small cell lung cancer || id:finn−b−C3_SCLC
SNP effect on Urate levels || id:ebi−a−GCST90025965
4
2
0
−2
0.05
0.10
0.15

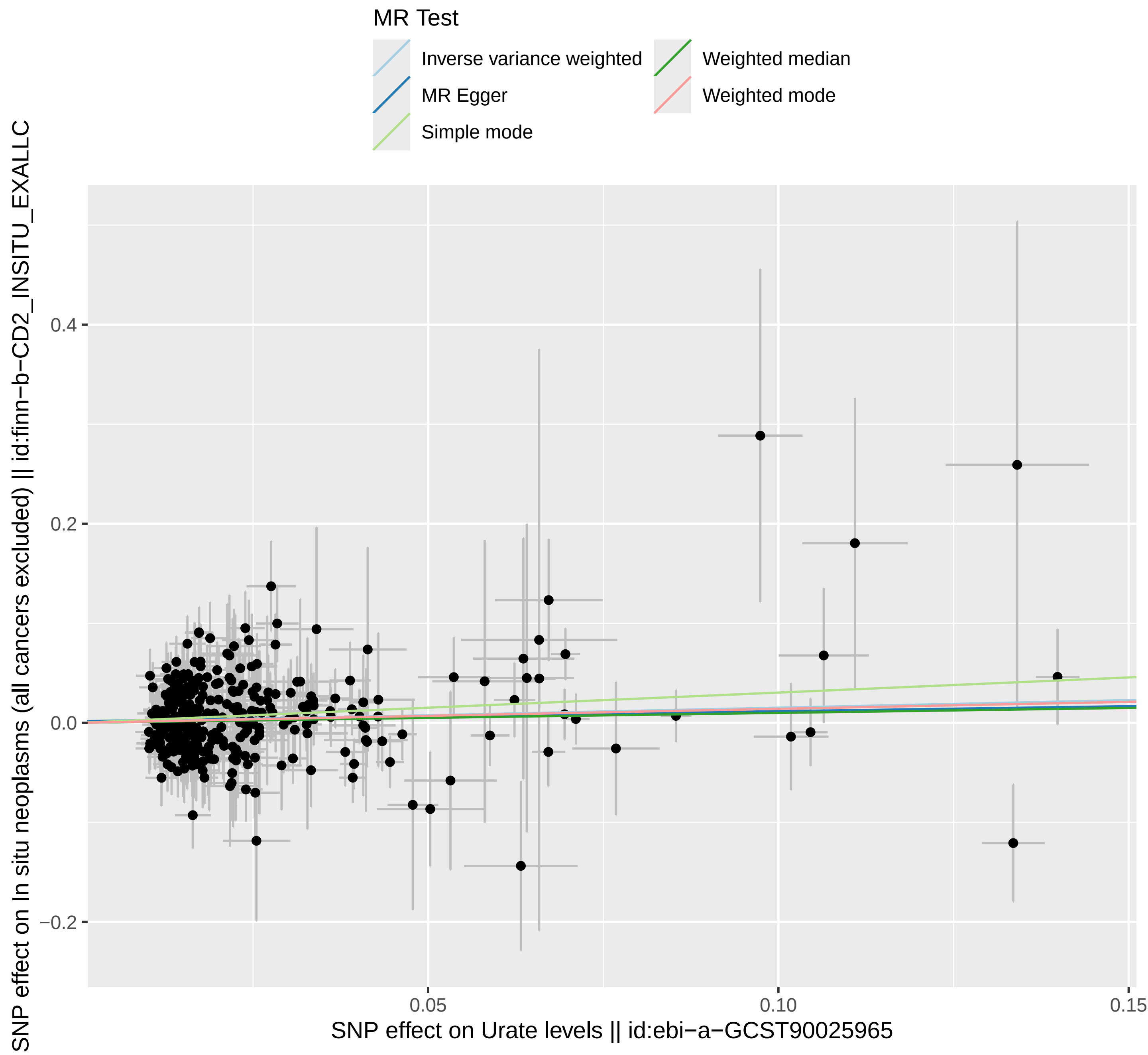
MR Test
Inverse variance weighted
MR Egger
Simple mode
Weighted median
Weighted mode
0.4
0.2
0.0
−0.2
0.05
0.10
0.15
SNP effect on Urate levels || id:ebi−a−GCST90025965
SNP effect on In situ neoplasms (all cancers excluded) || id:finn−b−CD2_INSITU_EXALLC

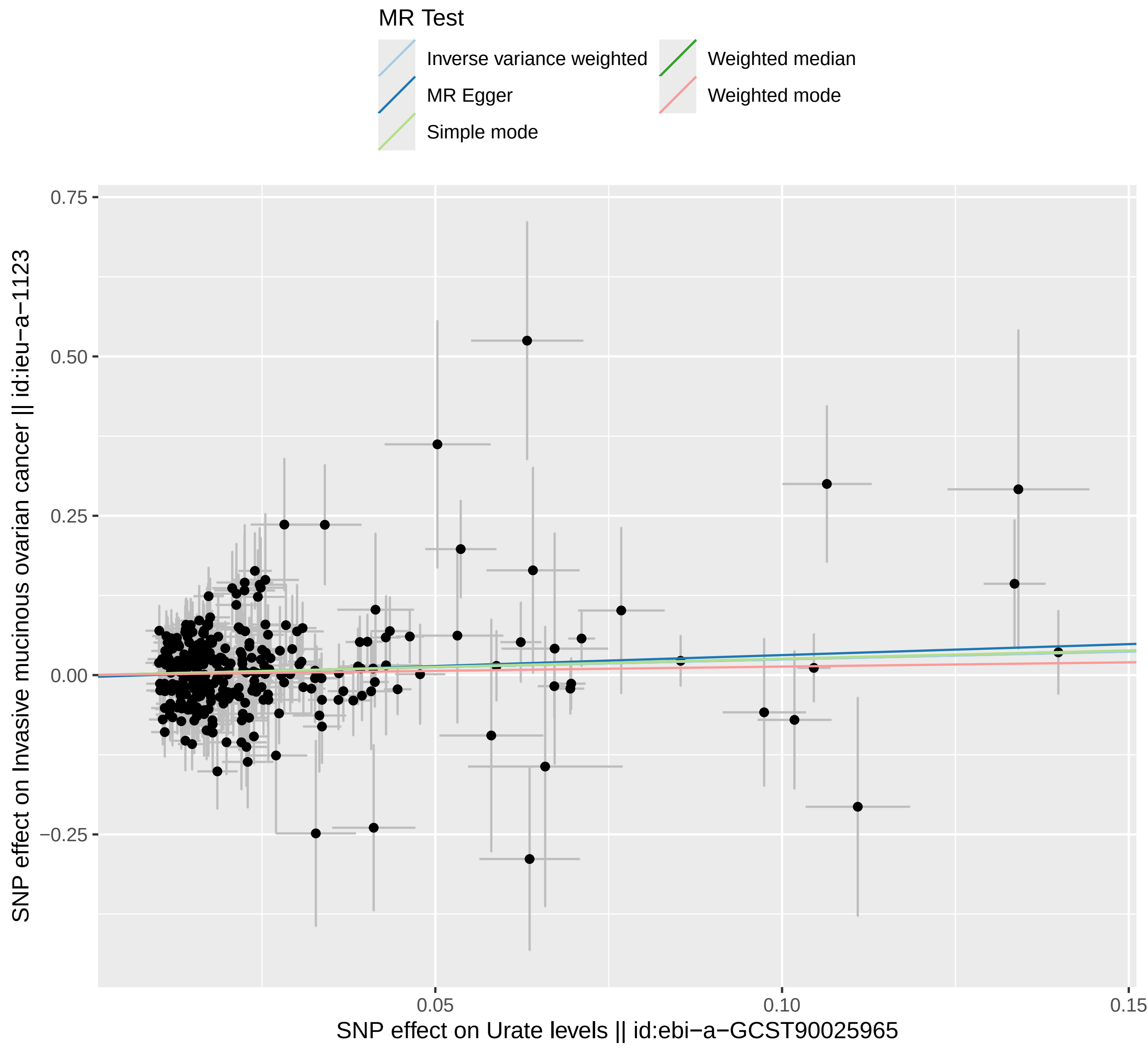
MR Test
Inverse variance weighted
MR Egger
Simple mode
Weighted median
Weighted mode
0.75
0.50
0.25
0.00
−0.25
0.05
0.10
0.15
SNP effect on Urate levels || id:ebi−a−GCST90025965
SNP effect on Invasive mucinous ovarian cancer || id:ieu−a−1123

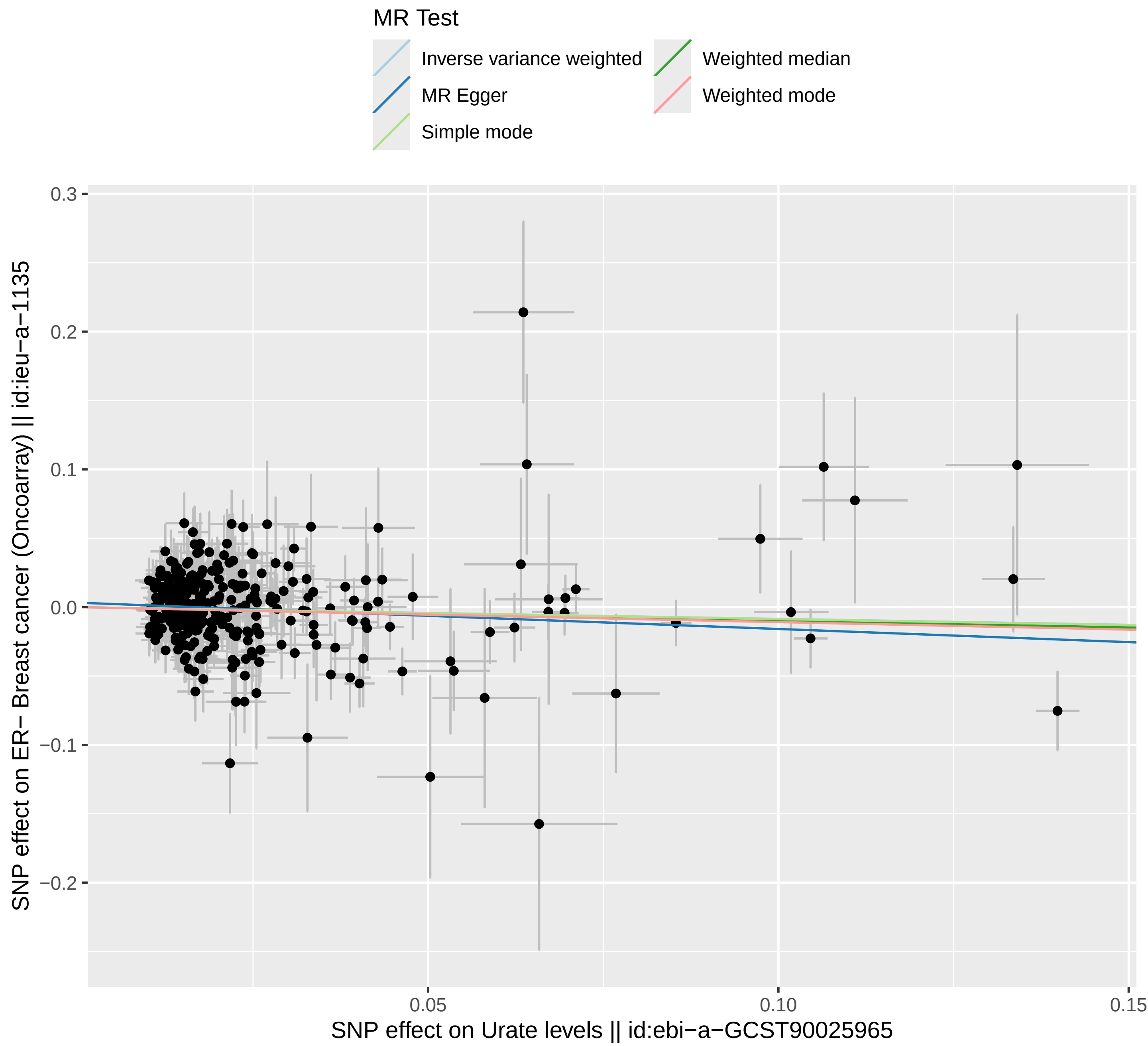

MR Test
Inverse variance weighted
MR Egger
Simple mode
Weighted median
Weighted mode
0.3
0.2
0.1
0.0
−0.1
−0.2
0.05
0.10
0.15
SNP effect on ER− Breast cancer (Oncoarray) || id:ieu−a−1135
SNP effect on Urate levels || id:ebi−a−GCST90025965

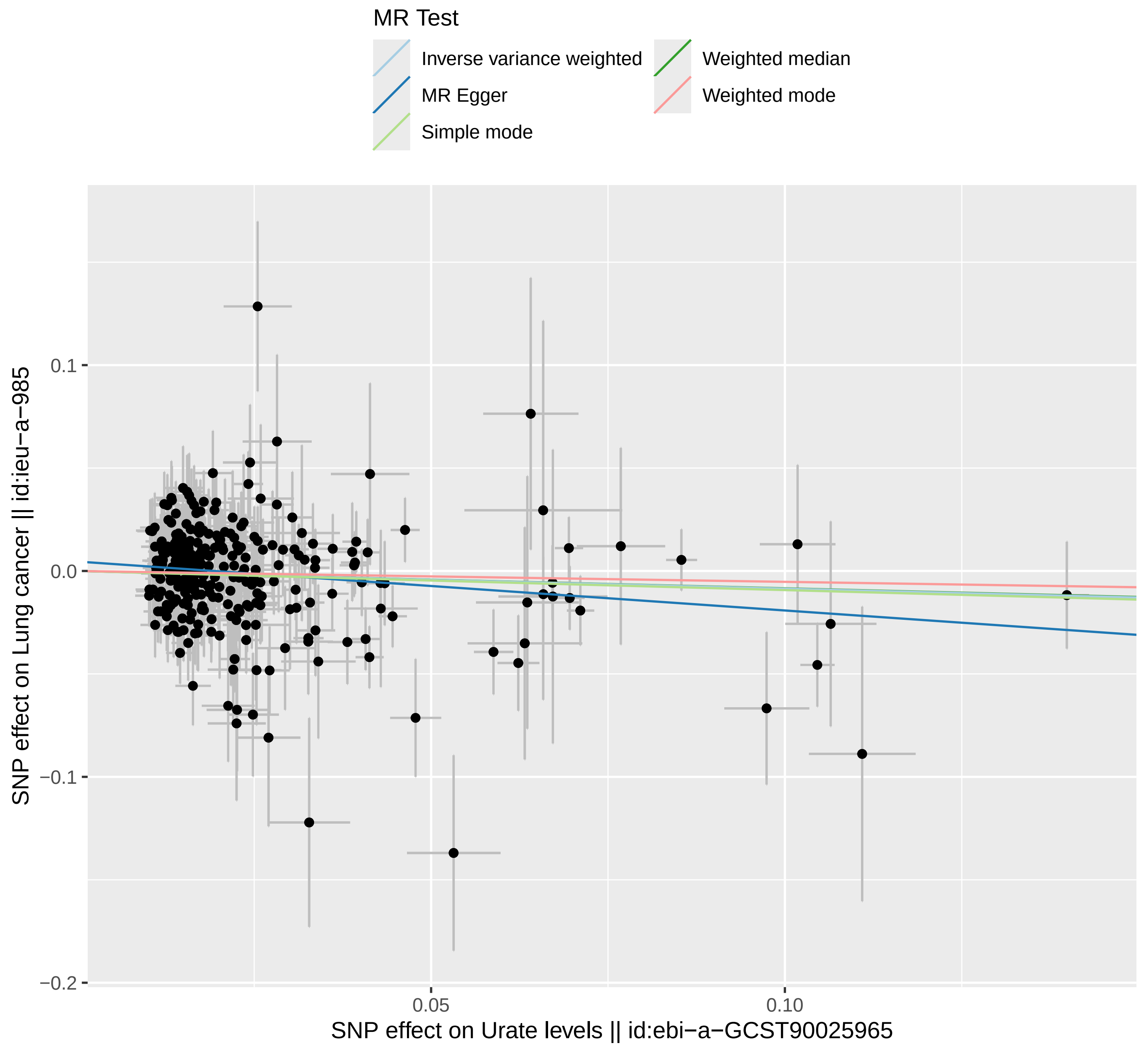
MR Test
Inverse variance weighted
MR Egger
Simple mode
Weighted median
Weighted mode
0.1
0.0
−0.1
−0.2
0.05
0.10
SNP effect on Lung cancer || id:ieu−a−985
SNP effect on Urate levels || id:ebi−a−GCST90025965

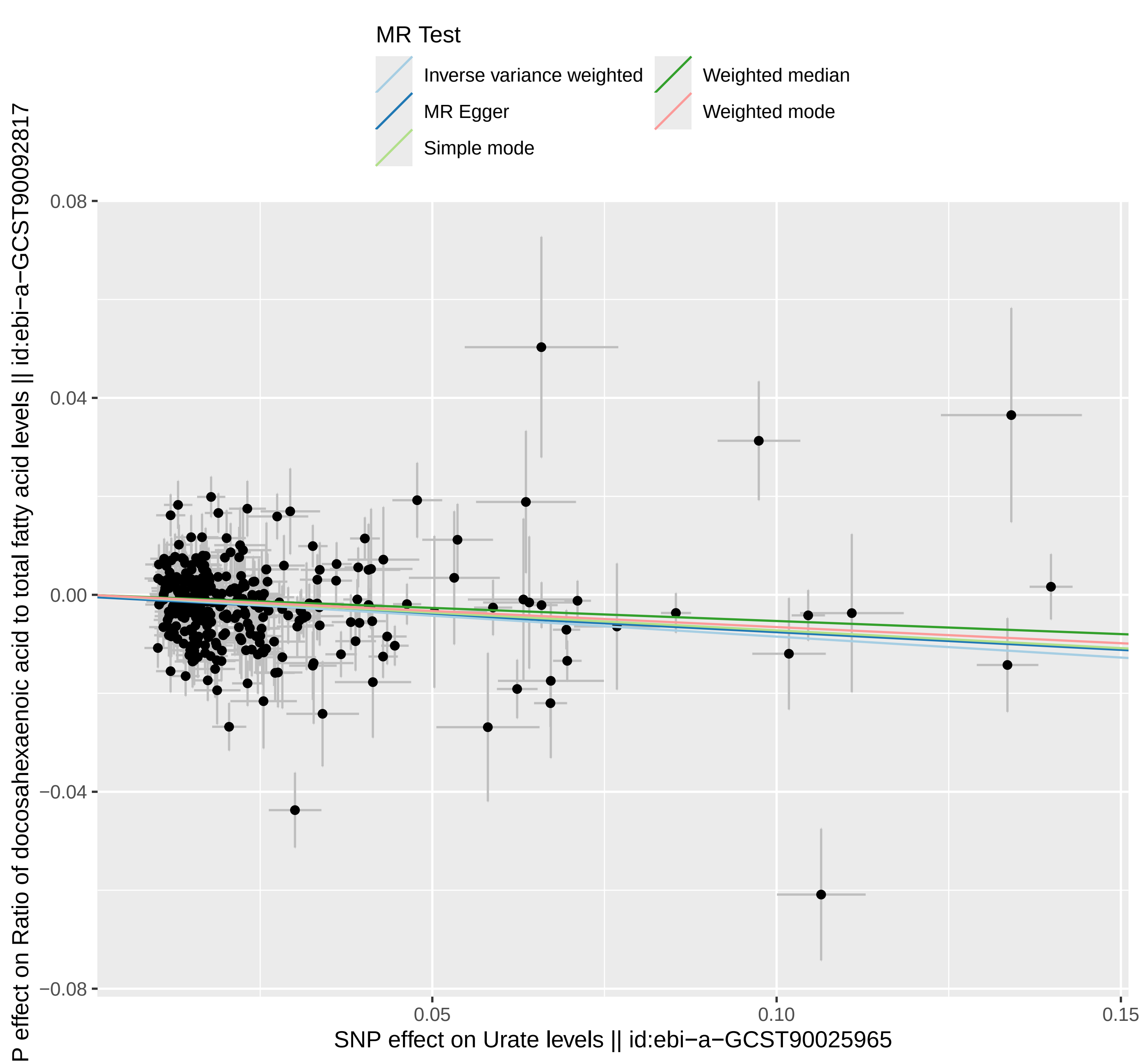
MR Test
Inverse variance weighted
MR Egger
Simple mode
Weighted median
Weighted mode
0.08
0.04
0.00
−0.04
−0.08
0.05
0.10
0.15
P effect on Ratio of docosahexaenoic acid to total fatty acid levels || id:ebi−a−GCST90092817
SNP effect on Urate levels || id:ebi−a−GCST90025965

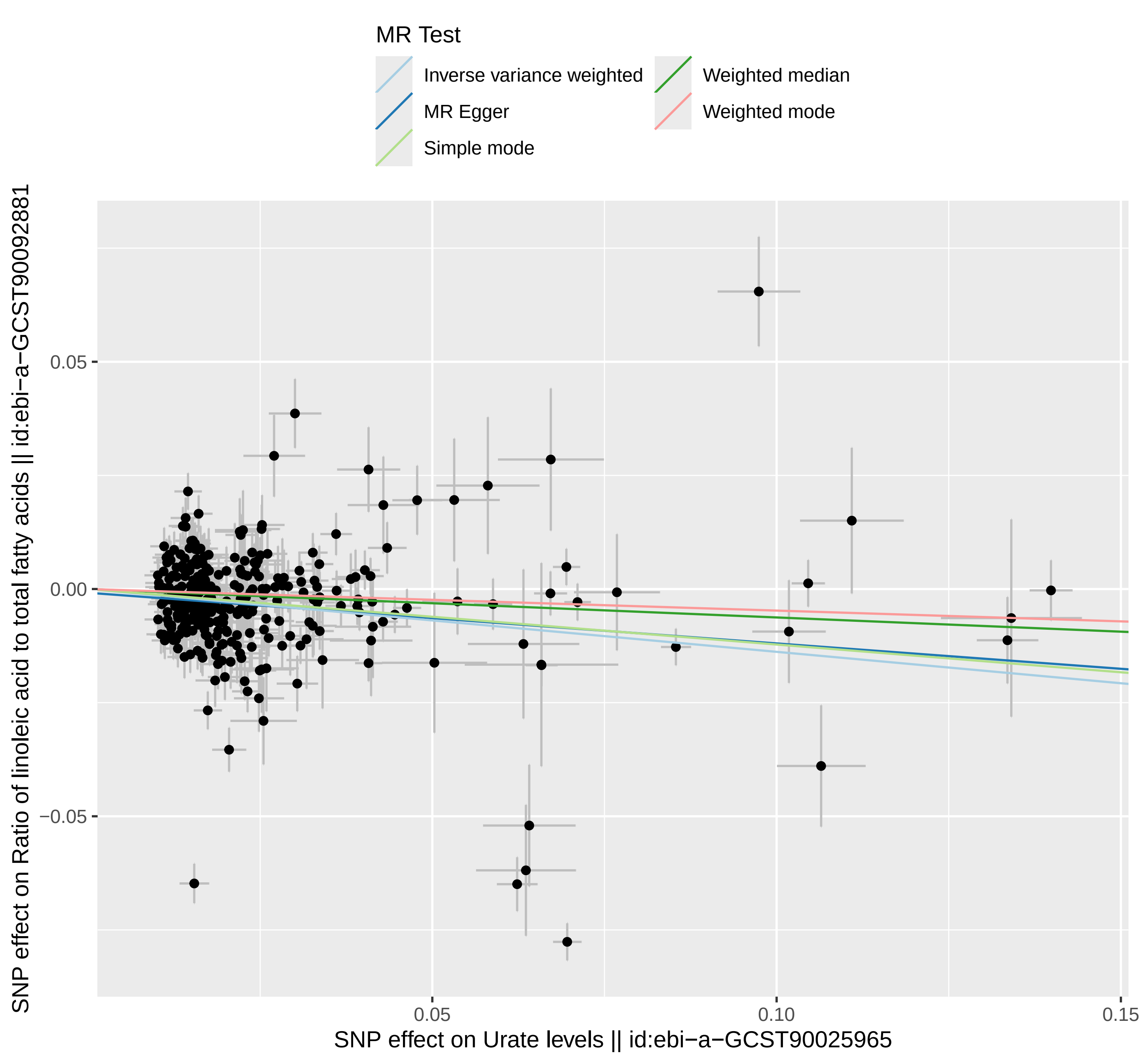

MR Test
Inverse variance weighted
MR Egger
Simple mode
Weighted median
Weighted mode
SNP effect on Ratio of linoleic acid to total fatty acids || id:ebi−a−GCST90092881
0.05
0.00
−0.05
0.05
0.10
0.15
SNP effect on Urate levels || id:ebi−a−GCST90025965

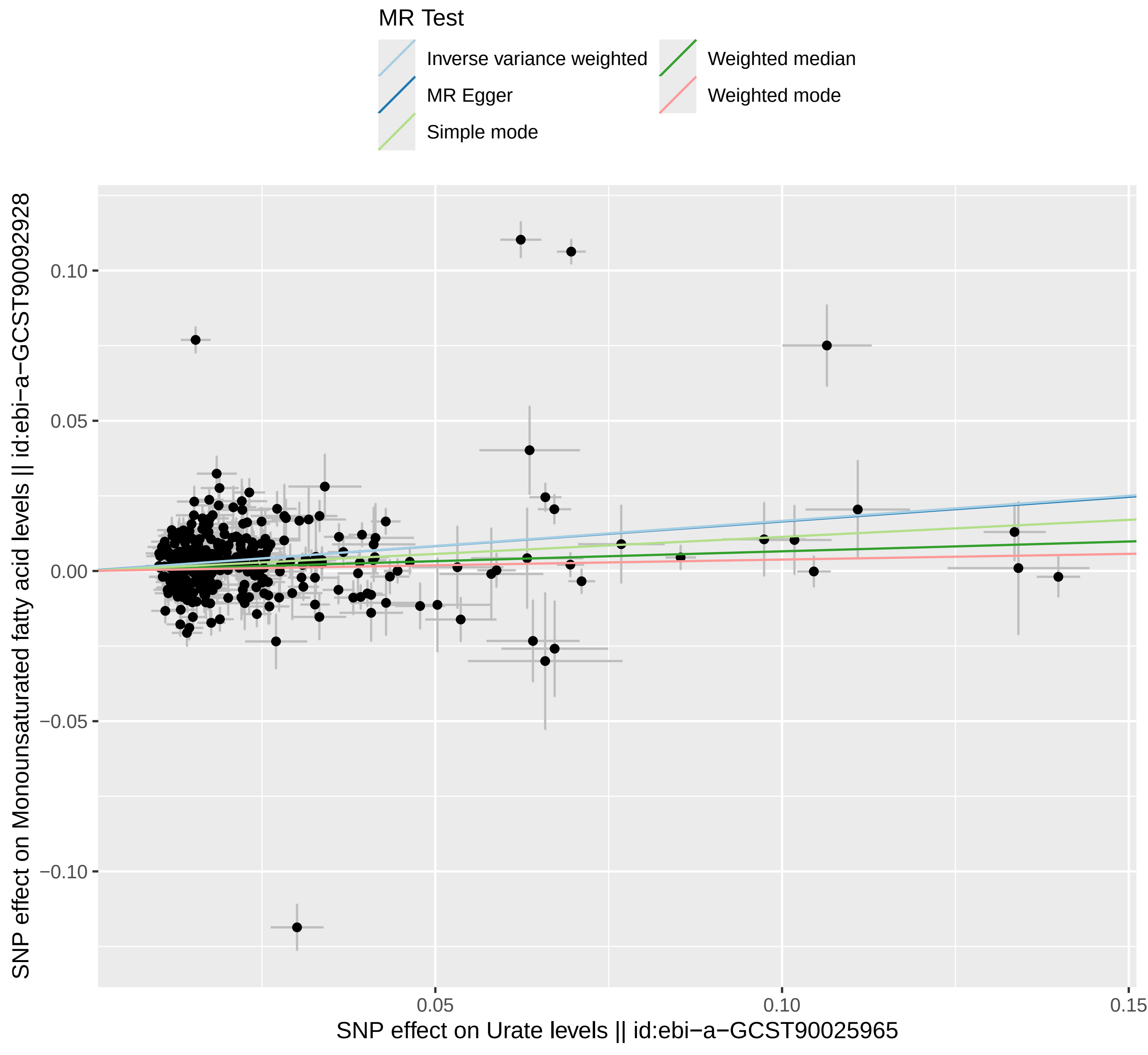

MR Test
Inverse variance weighted
MR Egger
Simple mode
Weighted median
Weighted mode
0.10
0.05
0.00
−0.05
−0.10
0.05
0.10
0.15
SNP effect on Monounsaturated fatty acid levels || id:ebi−a−GCST90092928
SNP effect on Urate levels || id:ebi−a−GCST90025965

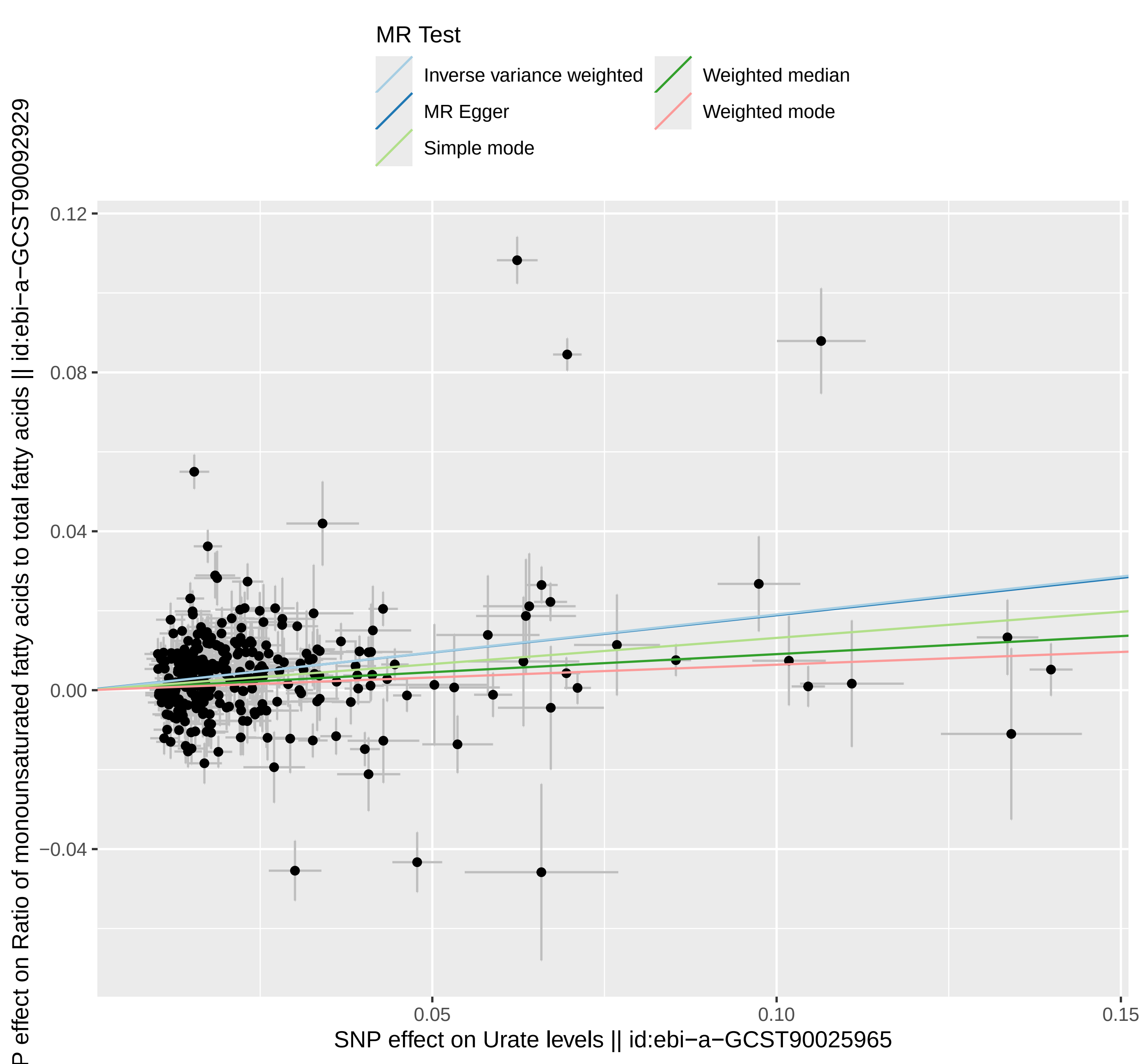
MR Test
Inverse variance weighted
MR Egger
Simple mode
Weighted median
Weighted mode
0.12
0.08
0.04
0.00
−0.04
0.05
0.10
0.15
SNP effect on Urate levels || id:ebi−a−GCST90025965
P effect on Ratio of monounsaturated fatty acids to total fatty acids || id:ebi−a−GCST90092929

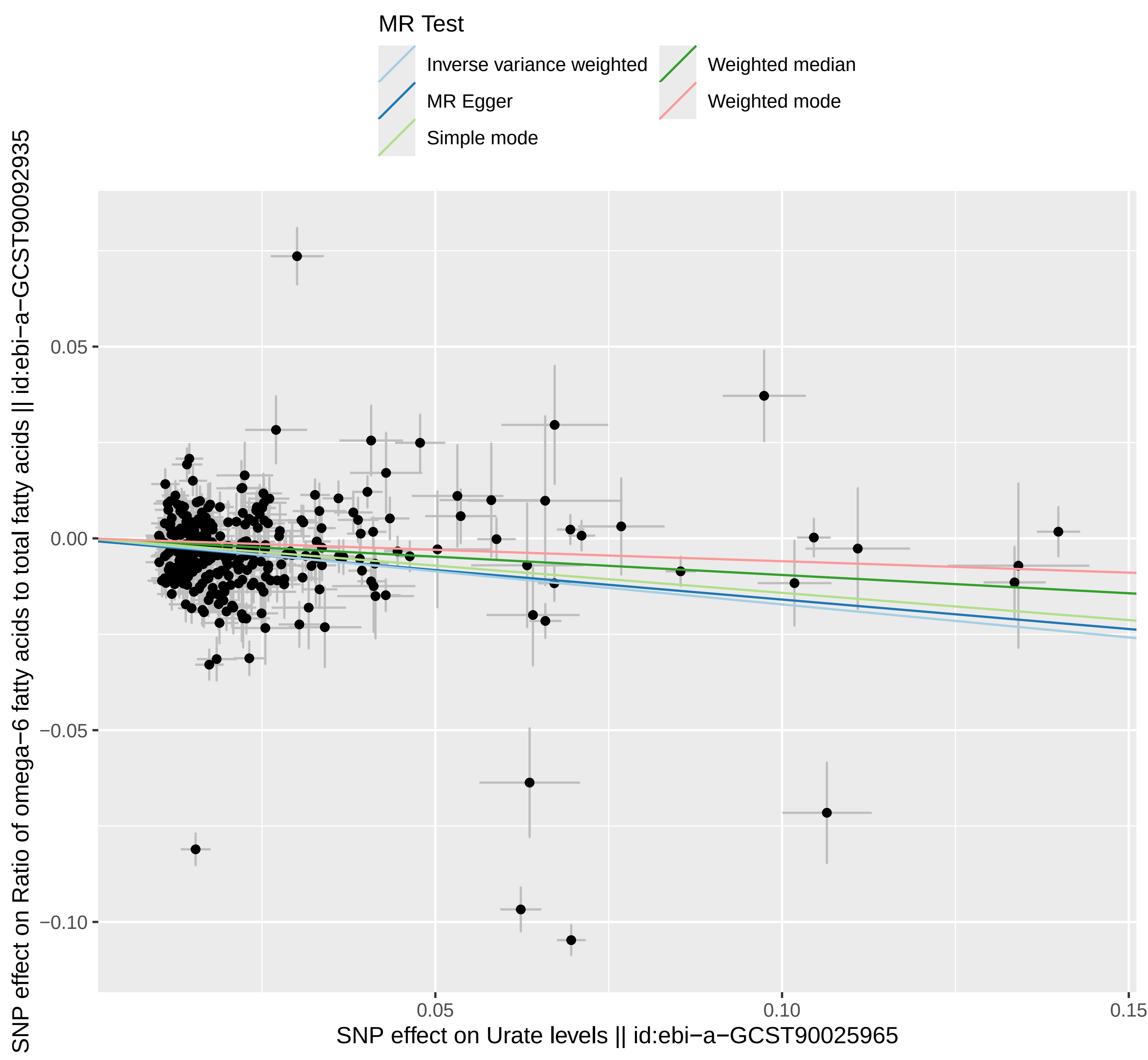

MR Test
Inverse variance weighted
MR Egger
Simple mode
Weighted median
Weighted mode
0.05
0.00
−0.05
−0.10
0.05
0.10
0.15
SNP effect on Ratio of omega−6 fatty acids to total fatty acids || id:ebi−a−GCST90092935
SNP effect on Urate levels || id:ebi−a−GCST90025965

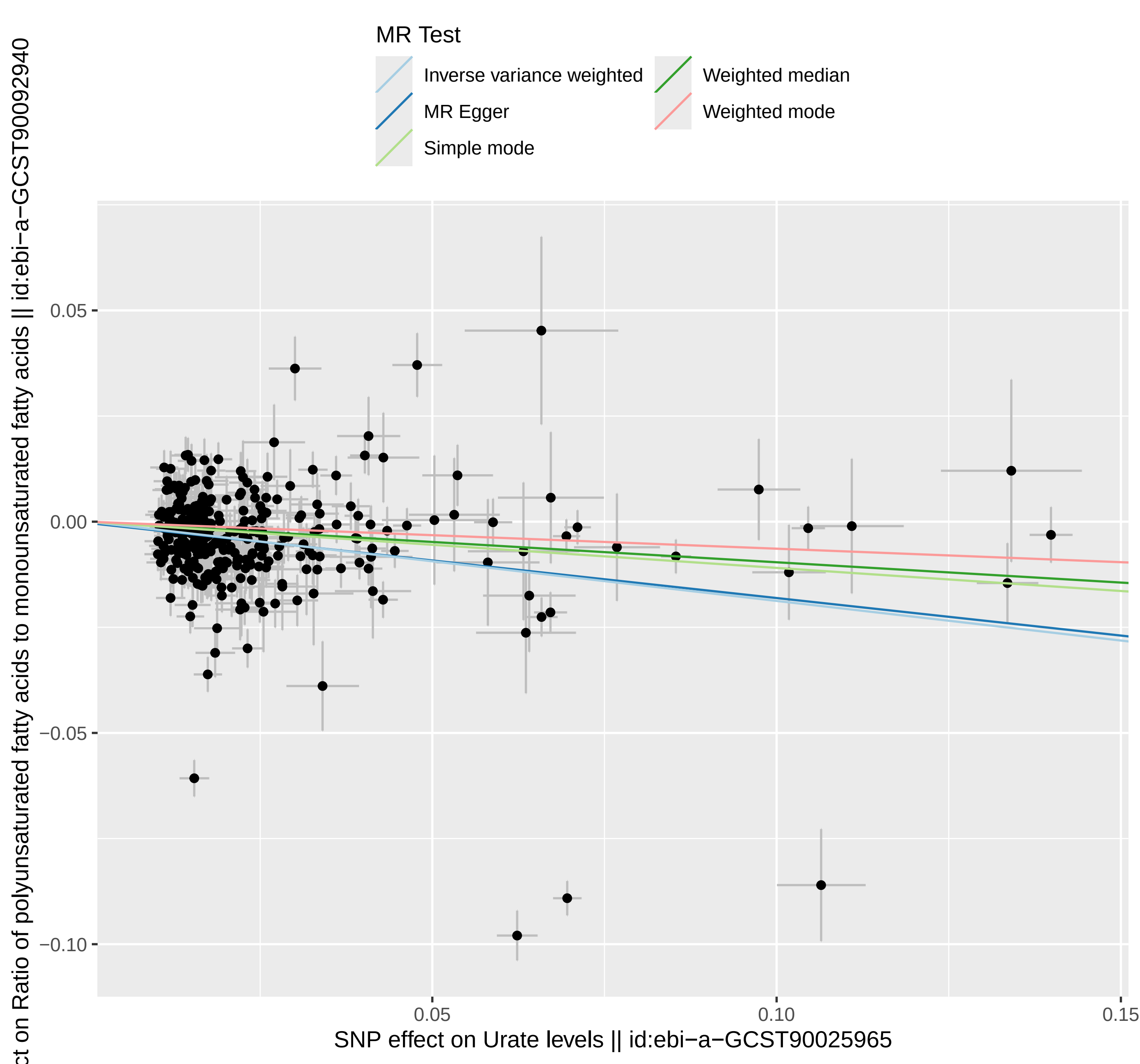
MR Test
Inverse variance weighted
MR Egger
Simple mode
Weighted median
Weighted mode
0.05
0.00
−0.05
−0.10
0.05
0.10
0.15
SNP effect on Urate levels || id:ebi−a−GCST90025965
ct on Ratio of polyunsaturated fatty acids to monounsaturated fatty acids || id:ebi−a−GCST90092940

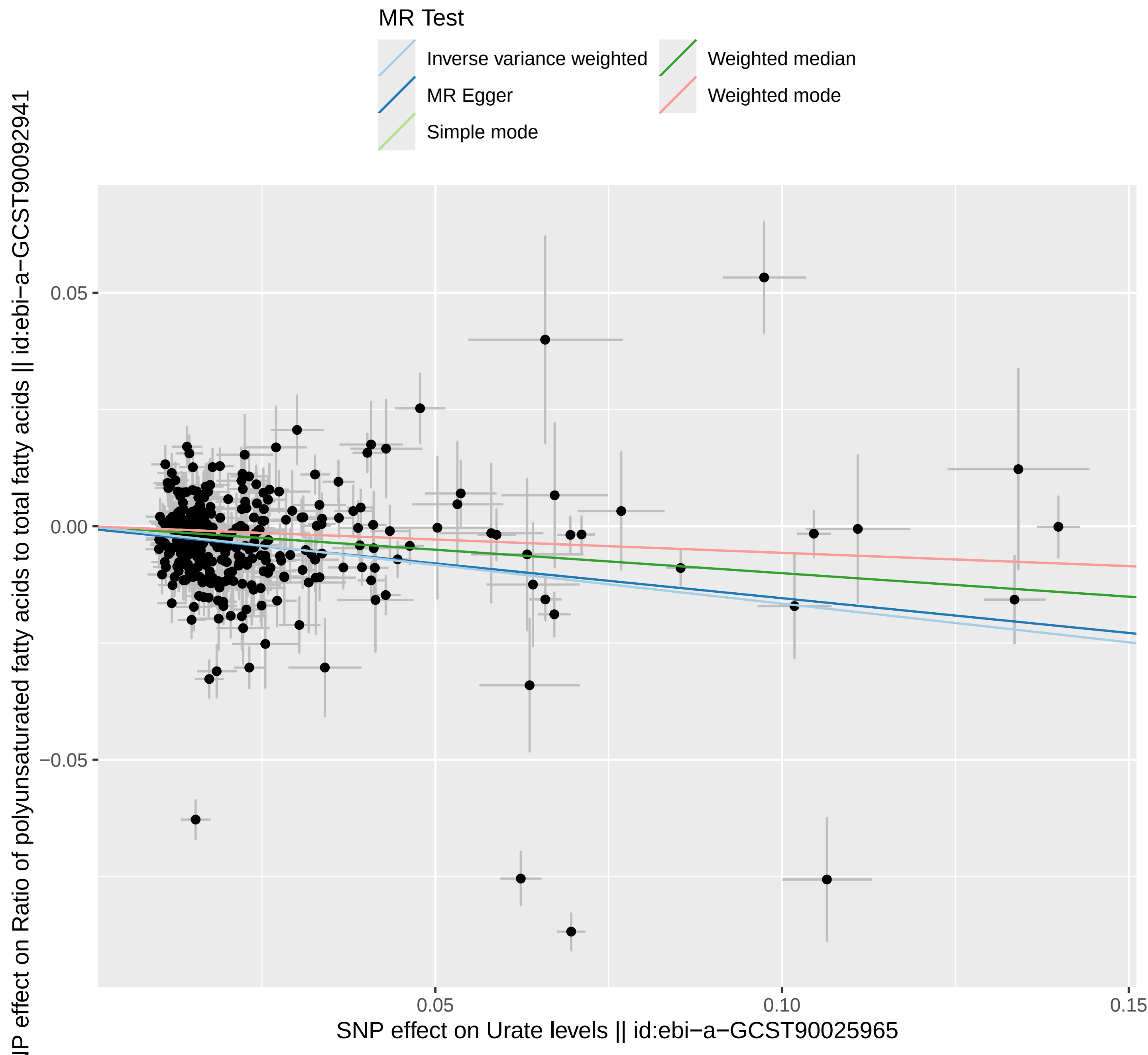

MR Test
Inverse variance weighted
MR Egger
Simple mode
Weighted median
Weighted mode
0.05
0.00
−0.05
0.05
0.10
0.15
SNP effect on Urate levels || id:ebi−a−GCST90025965
P effect on Ratio of polyunsaturated fatty acids to total fatty acids || id:ebi−a−GCST90092941

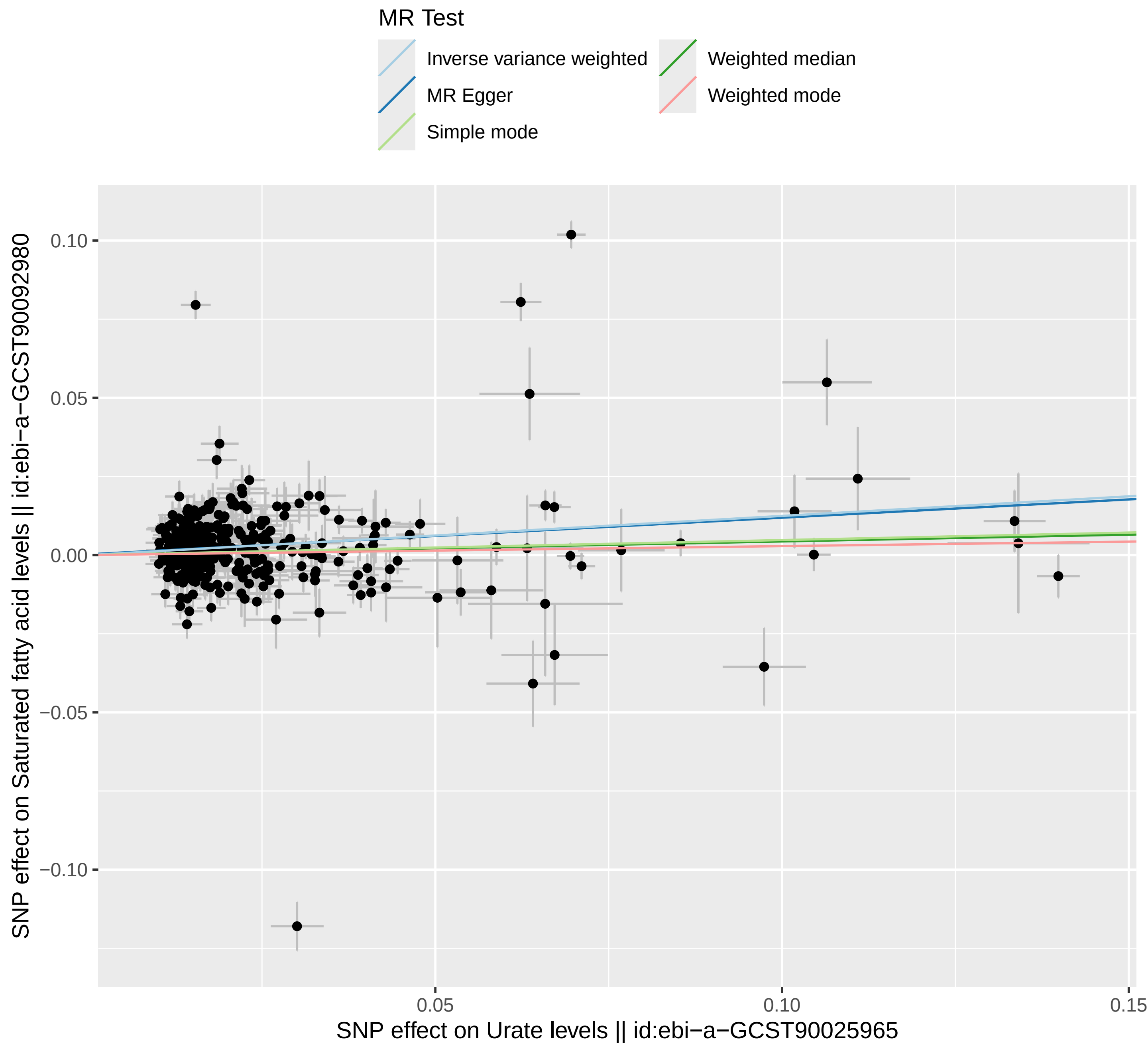
MR Test
Inverse variance weighted
MR Egger
Simple mode
Weighted median
Weighted mode
0.10
0.05
0.00
−0.05
−0.10
SNP effect on Saturated fatty acid levels || id:ebi−a−GCST90092980
0.05
0.10
0.15
SNP effect on Urate levels || id:ebi−a−GCST90025965

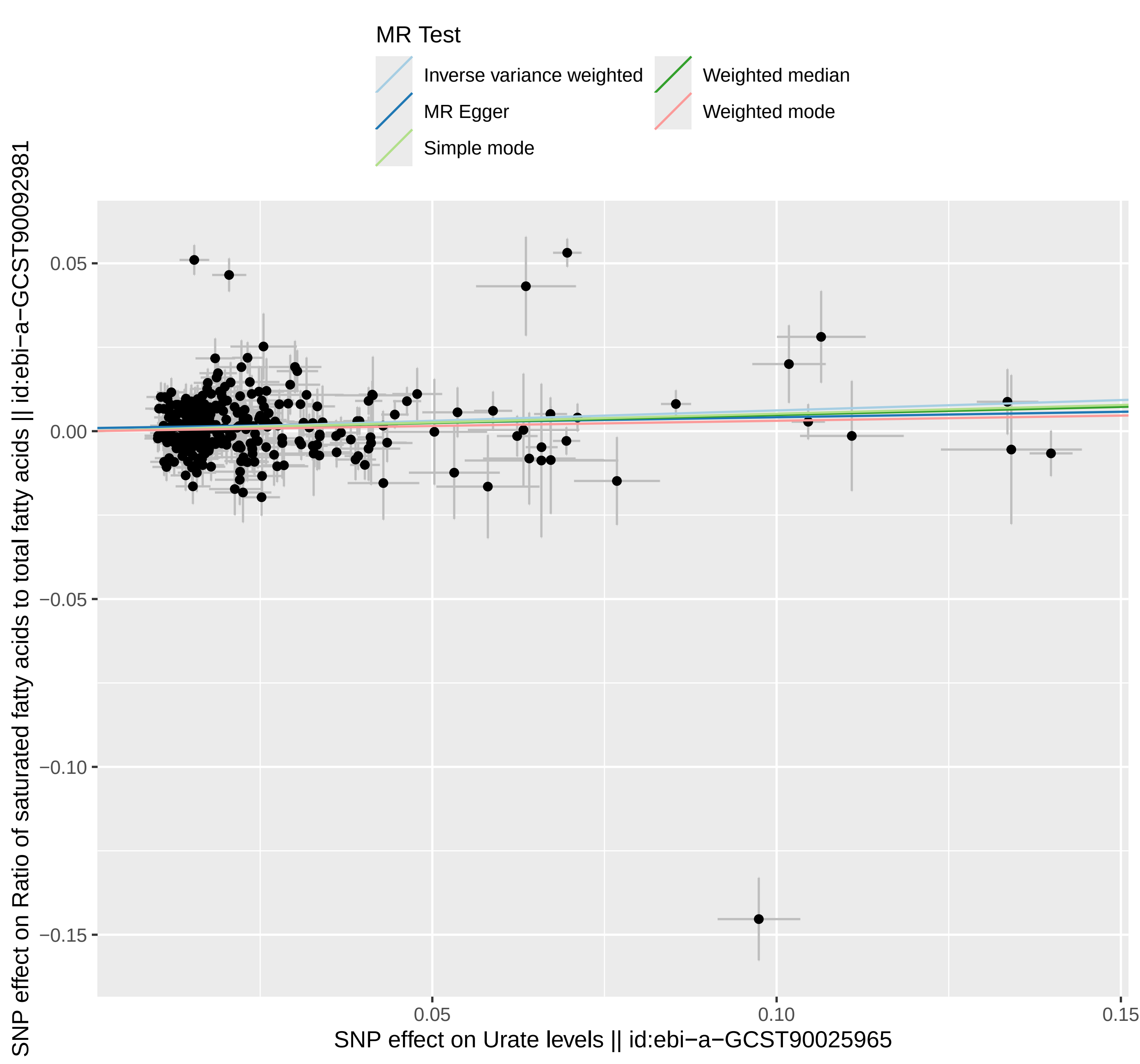

MR Test
Inverse variance weighted
MR Egger
Simple mode
Weighted median
Weighted mode
0.05
0.00
−0.05
−0.10
−0.15
0.05
0.10
0.15
SNP effect on Ratio of saturated fatty acids to total fatty acids || id:ebi−a−GCST90092981
SNP effect on Urate levels || id:ebi−a−GCST90025965

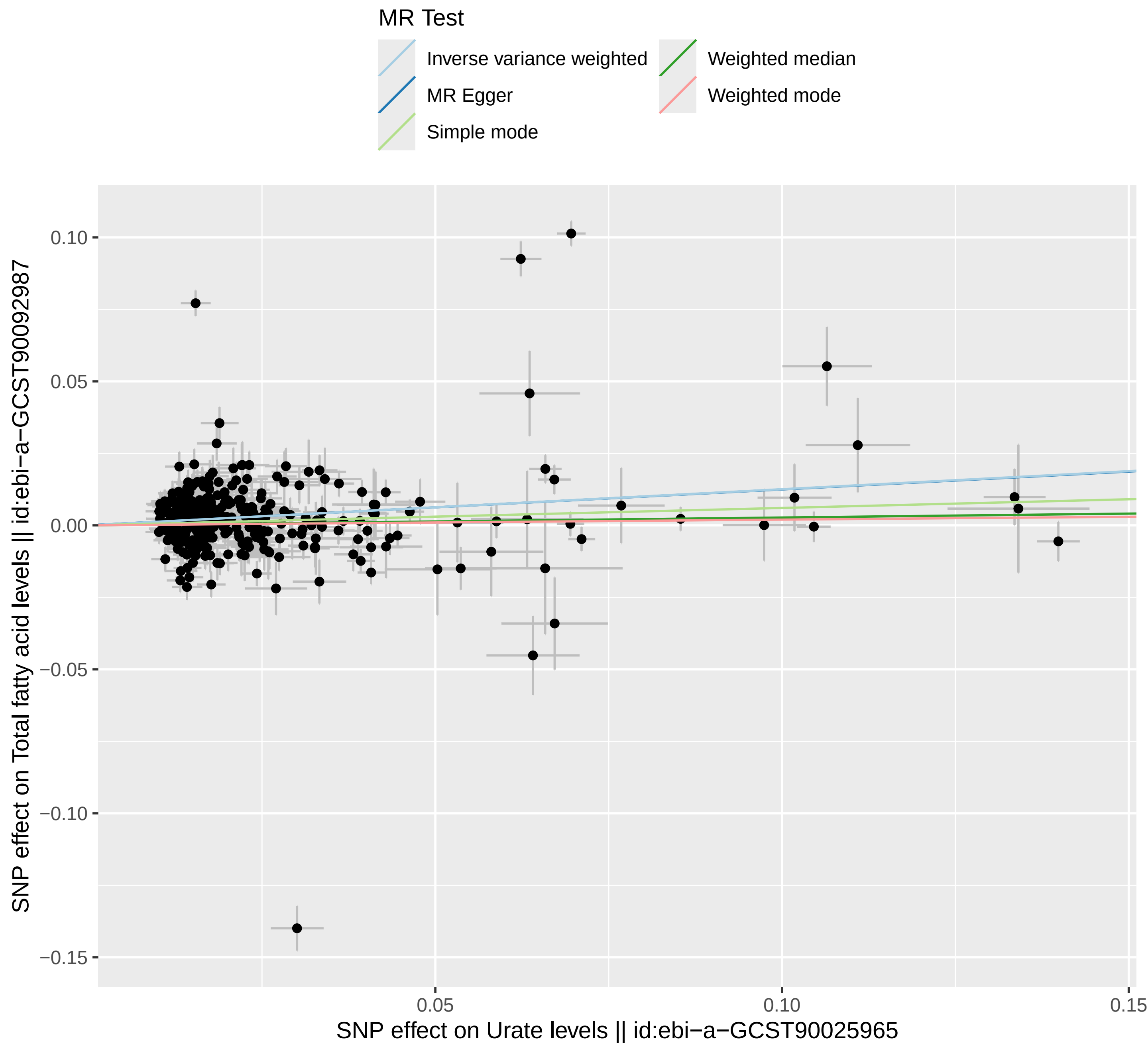

MR Test
Inverse variance weighted
MR Egger
Simple mode
Weighted median
Weighted mode
0.10
0.05
0.00
−0.05
−0.10
−0.15
0.05
0.10
0.15
SNP effect on Total fatty acid levels || id:ebi−a−GCST90092987
SNP effect on Urate levels || id:ebi−a−GCST90025965

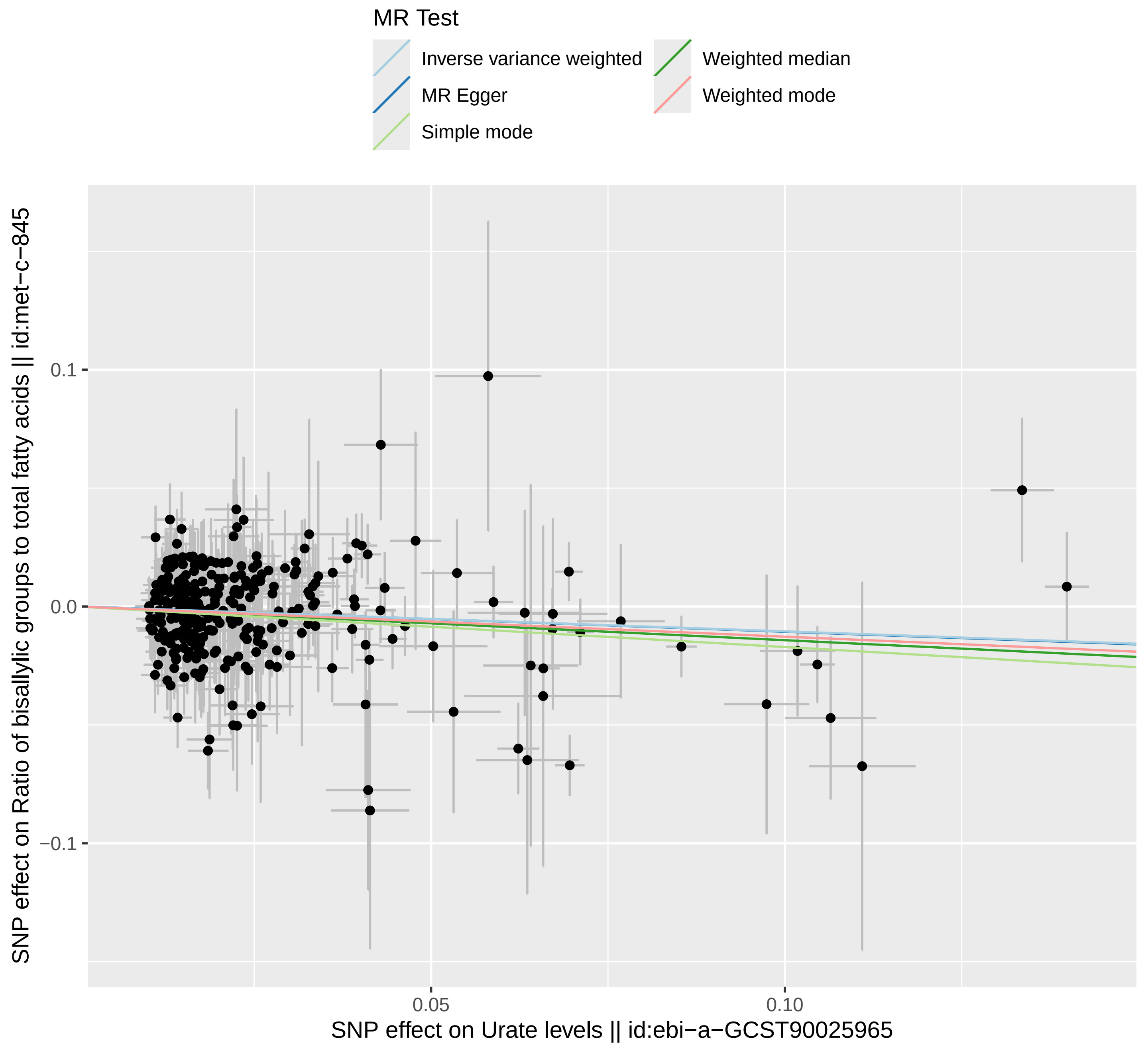
MR Test
Inverse variance weighted
MR Egger
Simple mode
Weighted median
Weighted mode
SNP effect on Ratio of bisallylic groups to total fatty acids || id:met−c−845
0.1
0.0
−0.1
0.05
0.10
SNP effect on Urate levels || id:ebi−a−GCST90025965

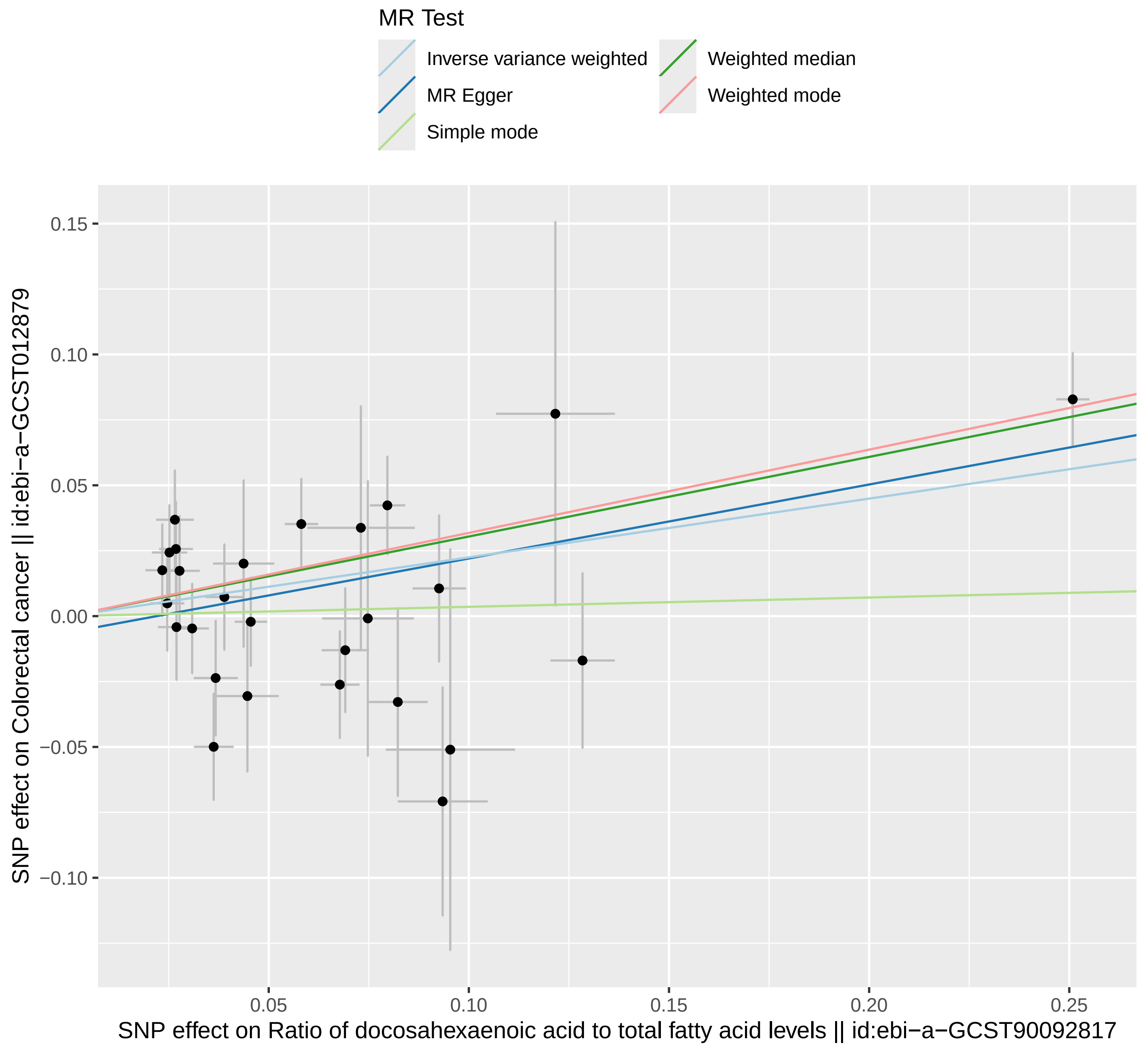
MR Test
Inverse variance weighted
MR Egger
Simple mode
Weighted median
Weighted mode
0.15
0.10
0.05
0.00
−0.05
−0.10
SNP effect on Colorectal cancer || id:ebi−a−GCST012879
0.05
0.10
0.15
0.20
0.25
SNP effect on Ratio of docosahexaenoic acid to total fatty acid levels || id:ebi−a−GCST90092817

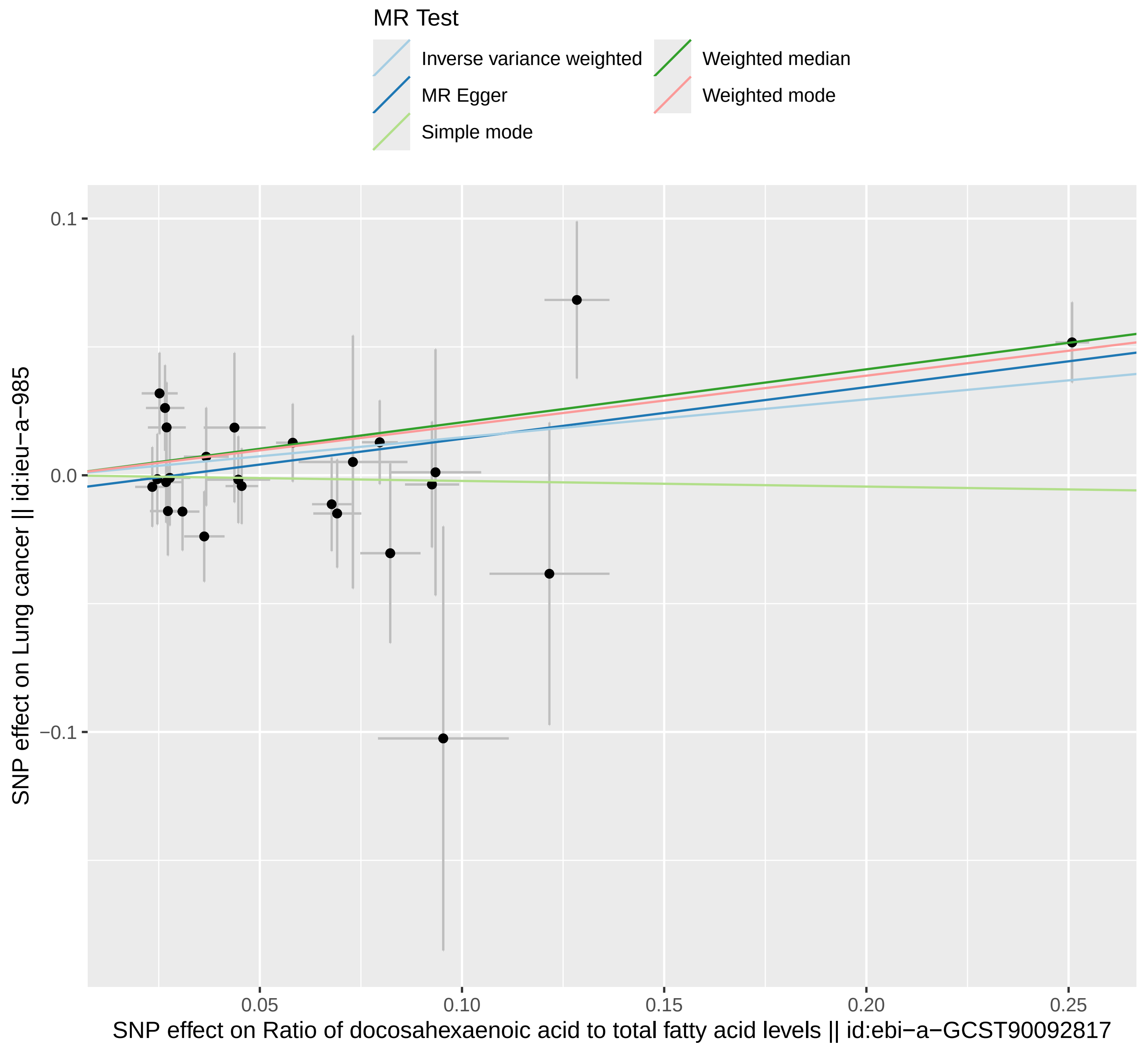

MR Test
Inverse variance weighted
MR Egger
Simple mode
Weighted median
Weighted mode
0.1
0.0
−0.1
SNP effect on Lung cancer || id:ieu−a−985
0.05
0.10
0.15
0.20
0.25
SNP effect on Ratio of docosahexaenoic acid to total fatty acid levels || id:ebi−a−GCST90092817

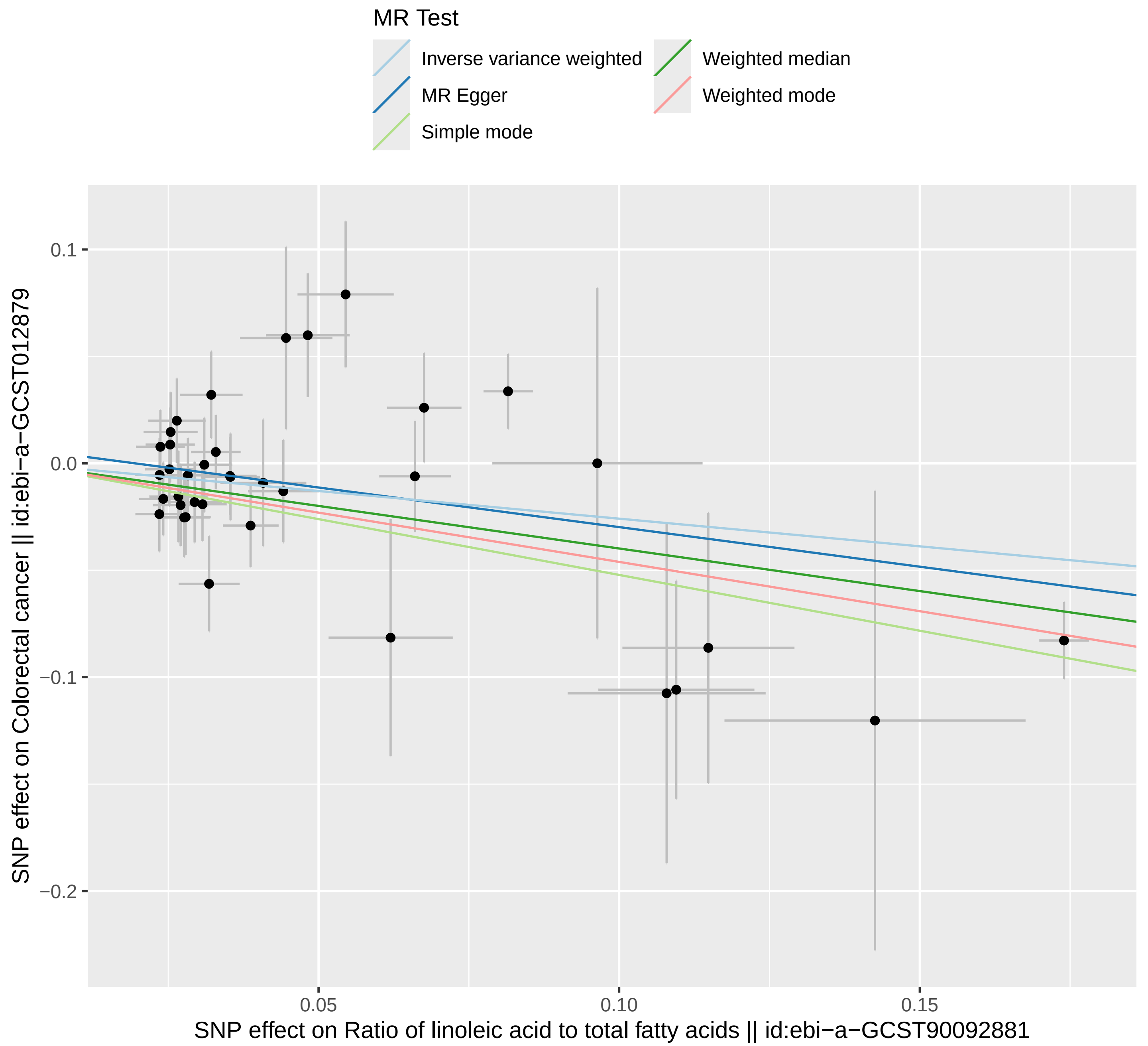

MR Test
Inverse variance weighted
MR Egger
Simple mode
Weighted median
Weighted mode
0.1
0.0
−0.1
−0.2
SNP effect on Colorectal cancer || id:ebi−a−GCST012879
0.05
0.10
0.15
SNP effect on Ratio of linoleic acid to total fatty acids || id:ebi−a−GCST90092881

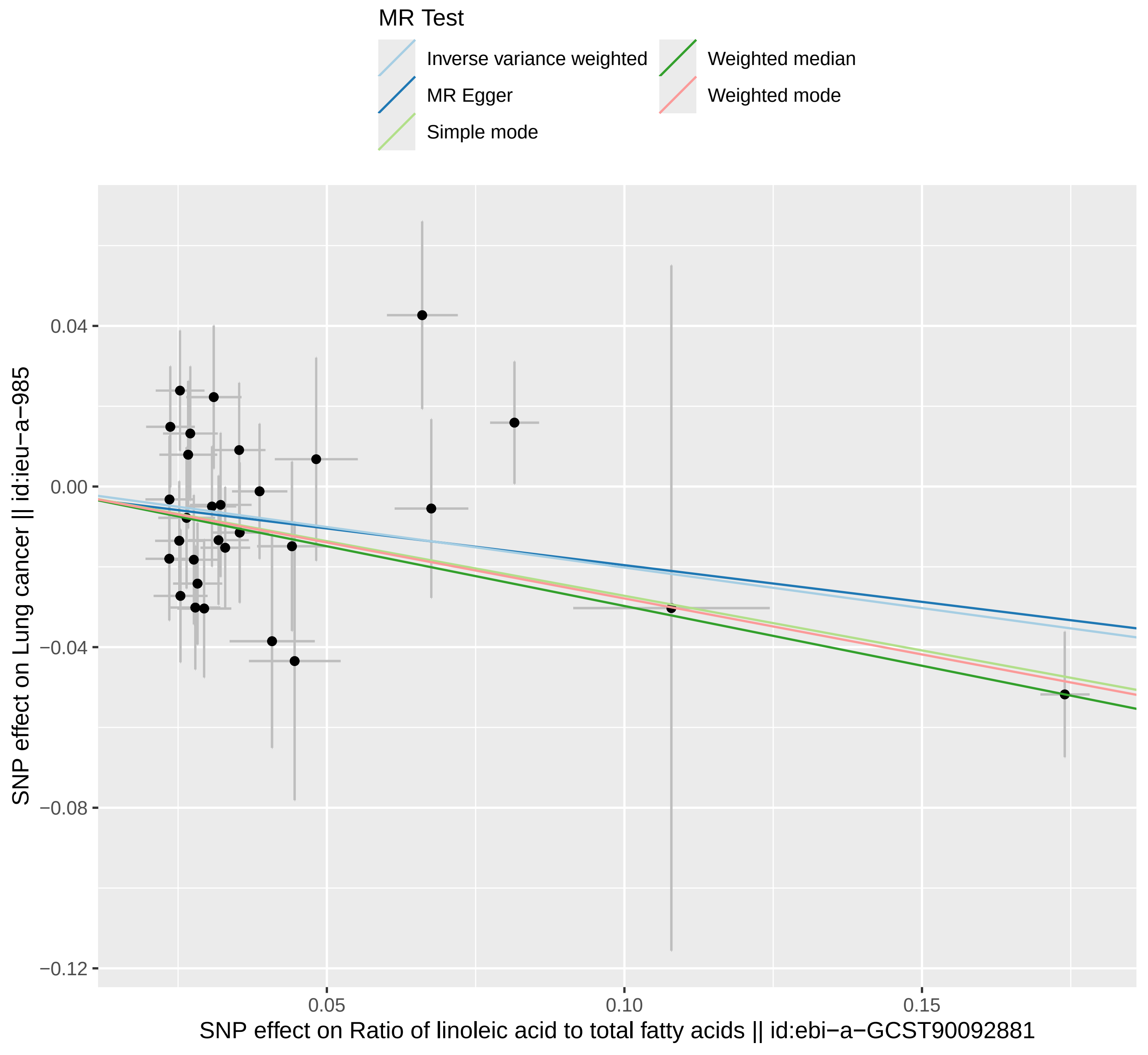

MR Test
Inverse variance weighted
MR Egger
Simple mode
Weighted median
Weighted mode
0.04
0.00
−0.04
−0.08
−0.12
0.05
0.10
0.15
SNP effect on Lung cancer || id:ieu−a−985
SNP effect on Ratio of linoleic acid to total fatty acids || id:ebi−a−GCST90092881

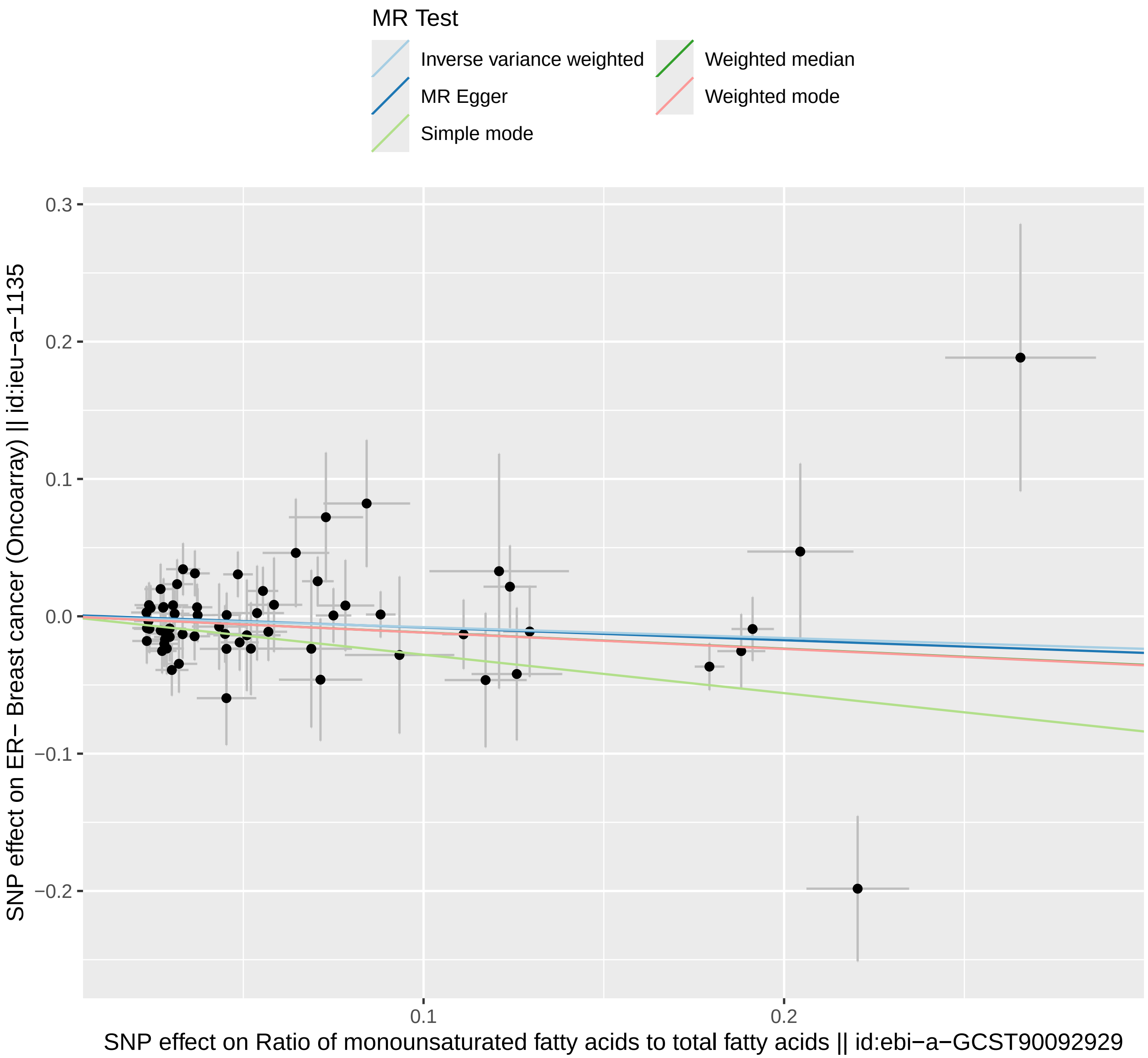
MR Test
Inverse variance weighted
MR Egger
Simple mode
Weighted median
Weighted mode
0.3
0.2
0.1
0.0
−0.1
−0.2
SNP effect on ER− Breast cancer (Oncoarray) || id:ieu−a−1135
0.1
0.2
SNP effect on Ratio of monounsaturated fatty acids to total fatty acids || id:ebi−a−GCST90092929

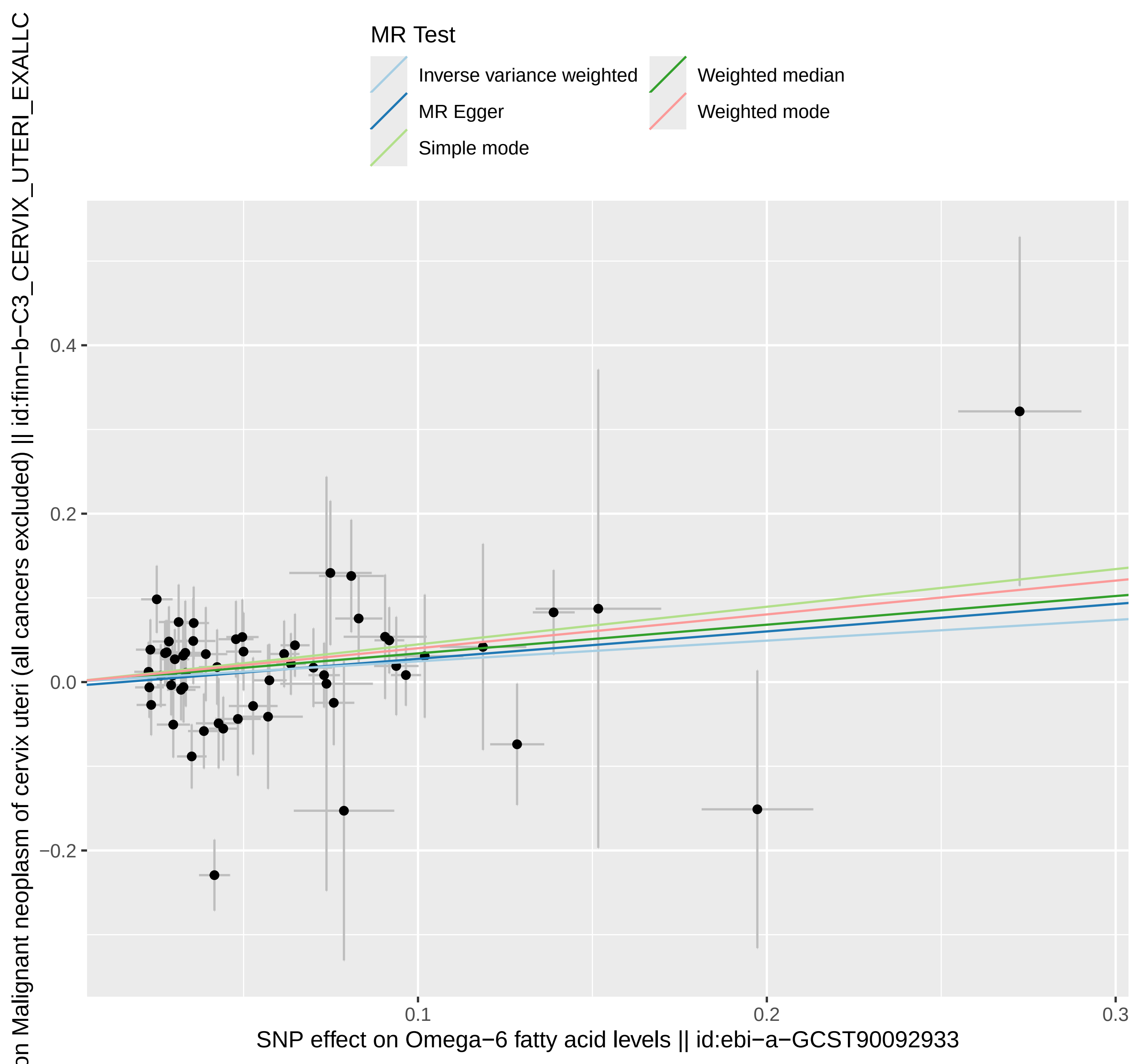

MR Test
Inverse variance weighted
MR Egger
Simple mode
Weighted median
Weighted mode
0.4
0.2
0.0
−0.2
0.1
0.2
0.3
SNP effect on Omega−6 fatty acid levels || id:ebi−a−GCST90092933
on Malignant neoplasm of cervix uteri (all cancers excluded) || id:finn−b−C3_CERVIX_UTERI_EXALLC

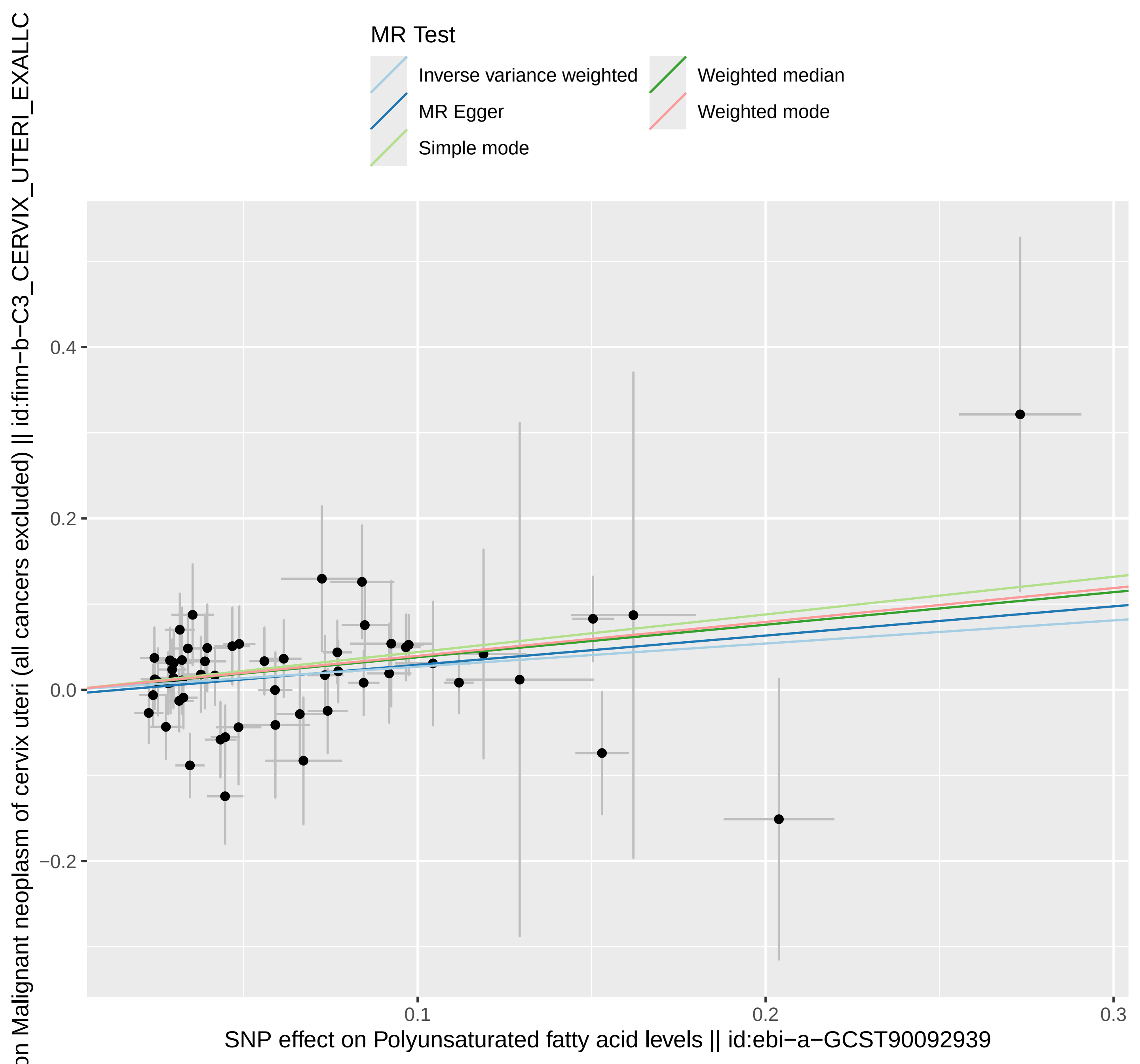

MR Test
Inverse variance weighted
MR Egger
Simple mode
Weighted median
Weighted mode
0.4
0.2
0.0
−0.2
0.1
0.2
0.3
SNP effect on Polyunsaturated fatty acid levels || id:ebi−a−GCST90092939
on Malignant neoplasm of cervix uteri (all cancers excluded) || id:finn−b−C3_CERVIX_UTERI_EXALLC

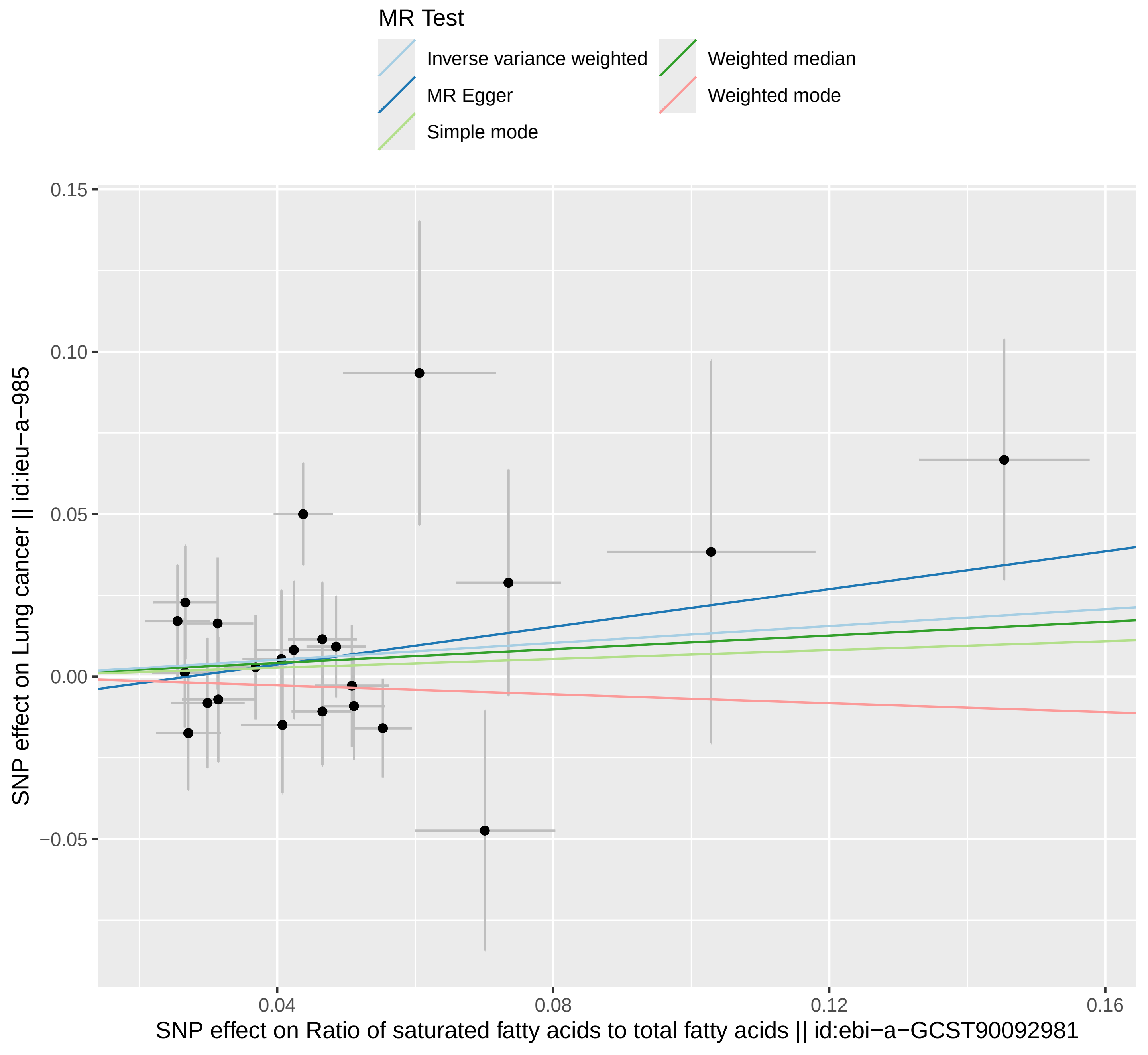

MR Test
Inverse variance weighted
MR Egger
Simple mode
Weighted median
Weighted mode
0.15
0.10
0.05
0.00
−0.05
0.04
0.08
0.12
0.16
SNP effect on Lung cancer || id:ieu−a−985
SNP effect on Ratio of saturated fatty acids to total fatty acids || id:ebi−a−GCST90092981

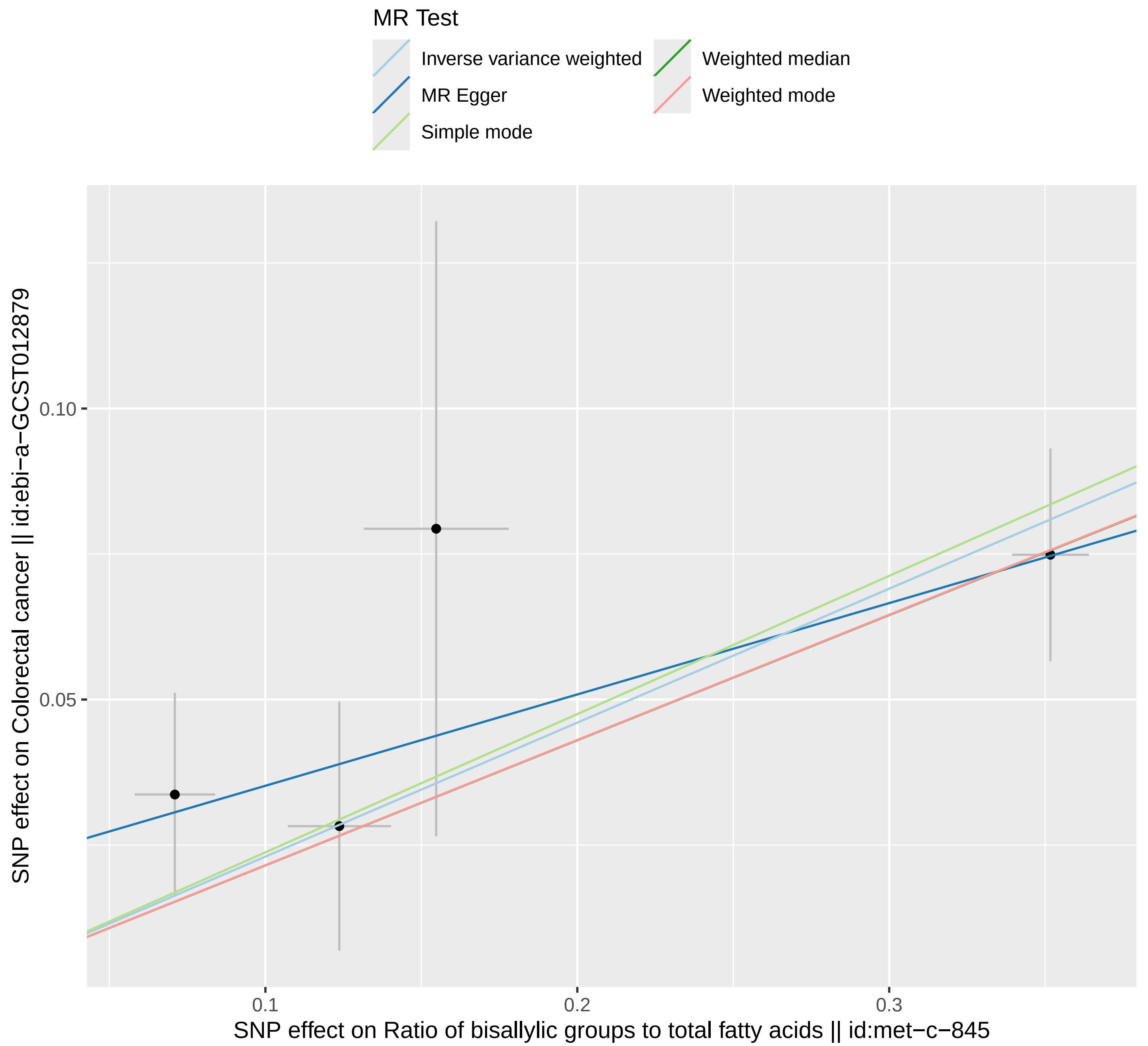
MR Test
Inverse variance weighted
MR Egger
Simple mode
Weighted median
Weighted mode
0.10
0.05
0.1
0.2
0.3
SNP effect on Colorectal cancer || id:ebi−a−GCST012879
SNP effect on Ratio of bisallylic groups to total fatty acids || id:met−c−845
